# Aerobic Lineage of the Oxidative Stress Response Protein Rubrerythrin Emerged in an Ancient Microaerobic, (Hyper)Thermophilic Environment

**DOI:** 10.3389/fmicb.2016.01822

**Published:** 2016-11-18

**Authors:** Juan P. Cardenas, Raquel Quatrini, David S. Holmes

**Affiliations:** ^1^Center for Bioinformatics and Genome Biology, Fundacion Ciencia & VidaSantiago, Chile; ^2^Facultad de Ciencias Biologicas, Universidad Andres BelloSantiago, Chile; ^3^Laboratory of Microbial Ecophysiology, Fundación Ciencia & VidaSantiago, Chile

**Keywords:** rubrerythrin, evolution, phylogeny, comparative genomics, microaerophilic, hyperthermophiles, GOE, cyanobacteria

## Abstract

Rubrerythrins (RBRs) are non-heme di-iron proteins belonging to the ferritin-like superfamily. They are involved in oxidative stress defense as peroxide scavengers in a wide range of organisms. The vast majority of RBRs, including classical forms of this protein, contain a C-terminal rubredoxin-like domain involved in electron transport that is used during catalysis in anaerobic conditions. Rubredoxin is an ancient and large protein family of short length (<100 residues) that contains a Fe-S center involved in electron transfer. However, functional forms of the enzyme lacking the rubredoxin-like domain have been reported (e.g., sulerythrin and ferriperoxin). In this study, phylogenomic evidence is presented that suggests that a complete lineage of rubrerythrins, lacking the rubredoxin-like domain, arose in an ancient microaerobic and (hyper)thermophilic environments in the ancestors of the Archaea Thermoproteales and Sulfolobales. This lineage (termed the “aerobic-type” lineage) subsequently evolved to become adapted to environments with progressively lower temperatures and higher oxygen concentrations via the acquisition of two co-localized genes, termed DUF3501 and RFO, encoding a conserved protein of unknown function and a predicted Fe-S oxidoreductase, respectively. Proposed Horizontal Gene Transfer events from these archaeal ancestors to Bacteria expanded the opportunities for further evolution of this RBR including adaption to lower temperatures. The second lineage (termed the cyanobacterial lineage) is proposed to have evolved in cyanobacterial ancestors, maybe in direct response to the production of oxygen via oxygenic photosynthesis during the Great Oxygen Event (GOE). It is hypothesized that both lineages of RBR emerged in a largely anaerobic world with “whiffs” of oxygen and that their subsequent independent evolutionary trajectories allowed microorganisms to transition from this anaerobic world to an aerobic one.

## Introduction

The ability to combat oxidative stress is a widespread feature found in most organisms including many obligate anaerobic and microaerophilic organisms. The Great Oxygenation Event (GOE), that has been hypothesized to occur approximately 2.3 billion years ago (Lyons et al., 2014), would likely have initiated an adaptation process to attenuate the threatening exposure to increased levels of reactive oxygen species (ROS). The development of efficient ROS scavenging mechanisms would have facilitated the co-evolution of redox proteins that could take advantage of the energetically advantageous use of oxygen as a terminal electron acceptor.

Reactive oxygen species are partially reduced oxygen compounds that are produced as byproducts of oxygen reduction and, in many conditions, their exacerbated production can lead to severe’ stress and even cellular death (Imlay, 2003). Several mechanisms have evolved to mitigate the oxidative stress caused by ROS including direct mechanisms and indirect scavenging of ROS (Farr and Kogoma, 1991; Maaty et al., 2009; Zuber, 2009). The most stable ROS is hydrogen peroxide (H_2_O_2_) and a variety of enzymes have evolved to remove it, such as catalases, peroxiredoxins and other peroxidases (Mishra and Imlay, 2012). Among the H_2_O_2_–scavenging enzymes, one variant with an important role in stress survival in several microorganisms, is rubrerythrin.

Rubrerythrin (RBR) is a member of the Ferritin-like superfamily (FLSF), consisting of proteins with a variety of different functions such as iron storage and/or iron detoxification (ferritins, Dps proteins, bacterioferritins), ubiquinone biosynthesis (COQ7 proteins), and radical scavenging (Mn-catalases, RBRs; Andrews, 2010). These proteins possess a four-helical bundle, configured in two anti-parallel helix pairs, forming a di-iron center, mediated by the coordination of (at least) six highly conserved residues among the four-helix structure (Andrews, 2010). In RBR, the di-iron center is responsible for the reduction of H_2_O_2_ and organic hydroperoxide (Dillard et al., 2011). The role of RBR in the reduction of H_2_O_2_ has been experimentally verified in several different organisms including aerobes (Sato et al., 2012), cyanobacteria (Zhao et al., 2007), and also obligate anaerobes (Weinberg et al., 2004). The classic RBR, found in the obligate anaerobe *Desulfovibrio vulgaris* contains, in addition to the FLSF domain, a C-terminal domain related to the rubredoxin family, putatively involved in electron transfer during catalysis (Andrews, 2010). This feature is conserved in most representatives of this family (Iyer et al., 2005), including a version of RBR with its rubredoxin domain in the opposite orientation with respect to the classic version called “reverse RBR” (Andrews, 2010). This suggests that the short rubredoxin-like domain has an important role in RBR function.

Despite the strong conservation of the rubredoxin-like domain in RBR proteins, there are also functional versions of this enzyme without the rubredoxin-like domain such as sulerythrin and ferriperoxin from *Sulfolobus tokodaii* (Wakagi, 2003) and *Hydrogenobacter thermophilus* (Sato et al., 2012). This one-domain RBR, composed only of the FLSF domain, was found to be widely distributed in microbial organisms, raising questions about how it evolved and whether the lack of the C-terminal rubredoxin-family domain had functional implications for the catalysis of H_2_O_2_. However, in obligate anaerobes, a functional and physical association between RBR and other proteins was demonstrated, suggesting that some other proteins might supply the missing catalytic function to the one-domain RBR (Weinberg et al., 2004; Le Fourn et al., 2011).

In this report, we have undertaken a phylogenomic analysis of RBRs using a number of techniques including conventional phylogenetic approaches, sequence similarity networks and comparisons of genomic neighborhoods. The objective was to derive a plausible trajectory of the evolution of RBRs and, if possible, to link this trajectory with postulated changes of temperature and atmospheric oxygen concentrations during the early stages of the earth over 3 billion years ago.

## Materials and Methods

### Compilation of RBR Sequences

Rubrerythrin sequences were obtained from NCBI non-redundant (NR) database using a two-step filter: first, the NR database was analyzed using *HMMsearch* (*HMMer* version 3.0) against *PF02915* (PFAM domain for Rubrerythrin, *E*-value < 10^-6^) followed by a RPS-BLAST search against COG, recovering all proteins with significant similarity to COG1592 (*E*-value < 10^-10^); all sequences with lengths less than 100 residues and/or with COG coverage values less than 70% were discarded. **Supplementary Table S1** lists the sequences found using this strategy.

### Sequence Similarity Network

The sequence similarity network elaboration was as described by Atkinson et al. (2009). A total of 4527 RBR sequences found by the aforementioned method were clustered using CD-HIT software (Fu et al., 2012), resulting in 2631 representatives comprising groups defined by 90% identity. These filtered sequences were analyzed in a BLASTp-all-versus-all round (no-filter, default parameters) with a threshold *E*-value of 10^-35^. The pairwise bit scores were used as measure of distance for the network, visualized in Cytoscape 3.0.2 using *Organic layout*.

### Phylogenetic Analyses

Protein sequences were aligned using MAFFT (Katoh and Standley, 2014) with the L-INS strategy. The phylogenetic analyses were performed using either maximum likelihood (ML) or Bayesian inferences (BI). For ML, the trees were elaborated in PhyML, version 3.1 (Guindon et al., 2009) with different sets of parameters for each case. For BI, the trees were elaborated in MrBayes version 3.2.6 (Huelsenbeck and Ronquist, 2001), with different parameters for each case (see “Results”). The selected substitution models for both ML and BI analyses were selected using ModelGenerator (Keane et al., 2006).

For the “aerobic-type” RBR tree, the alignment containing 127 ungapped and unambiguous sites for 335 taxa was analyzed by MrBayes with five substitution rate categories and gamma-distributed rate variation, using LG as the prior model. Bayesian analysis was run for five million generations (in two independent runs, using four chains and a heating parameter of 0.1); trees were saved every 200 generations and posterior probabilities calculated after discarding the first 33% of trees.

For the DUF3501 and the rubrerythrin-associated Fe-S oxidoreductase (RFO) protein families (see below), the trees were constructed using PhyML. For DUF3501, the alignment containing 118 ungapped and unambiguous sites for 258 taxa was used; the ML-based tree was performed using the LG model with four substitution rate categories and gamma-distributed rate variation, with a proportion of invariable sites. The tree topology search was performed by the combination of NNIs and SPRs strategies and the approximate likelihood ratio was computed by the SH-like branch support test (Anisimova et al., 2011). The RFO tree was computed using similar parameters, from an alignment containing 289 ungapped and unambiguous sites for 206 taxa. Additionally, a tree of the “cyanobacterial group” RBR orthologs was computed from an alignment containing 289 ungapped and unambiguous sites for 206 taxa, using the same configuration used for the two latter phylogenetic analyses.

### Other Analyses

Genomic contexts for the genes encoding rubrerythrin were obtained from IMG-JGI (Markowitz et al., 2012), using a set of 4461 complete prokaryotic genomes. Protein domain analyses were carried out using InterProScan (Quevillon et al., 2005). Co-localized genes with RBR were searched in the complete prokaryotic genome collection using BLAST (Altschul et al., 1997).

## Results and Discussion

### Identification of Different Rubrerythrin Groups

In order to study the evolution of RBR forms, the first step was the preparation of a trustworthy set of these proteins. The search of putative RBR homologs was made using a dual criterion selection that combines the sensibility of Hidden Markov Model (HMM) based search (PF02915) with a confirmative application of RPS-BLAST against the COG1592 family (corresponding to the RBR family). This double filter allowed a discrimination to be made between RBRs and other members of the FLSF, including members of the Mg^+2^-protoporphyrin IX monomethyl ester cyclase subfamily, which were also included in the HMM profile for PF02915.

A sequence similarity network approach was implemented in order to identify subfamilies inside the RBR family. Similarity networks are acceptable approximations for the study of huge protein families, although they are not a replacement for phylogenetic studies (Atkinson et al., 2009). The use of a given *E*-value (or score) threshold in an “all-vs-all” BLAST permits the recovery of clusters that can be visualized in a network fashion, where each node is an homolog or family member, and each edge is the measure of the pairwise score obtained by BLAST. The distances between different node groups are inversely proportional to the value of their pairwise scores: higher BLAST pairwise scores between multiple sequences promote their clustering, and vice versa.

**FIGURE 1:**
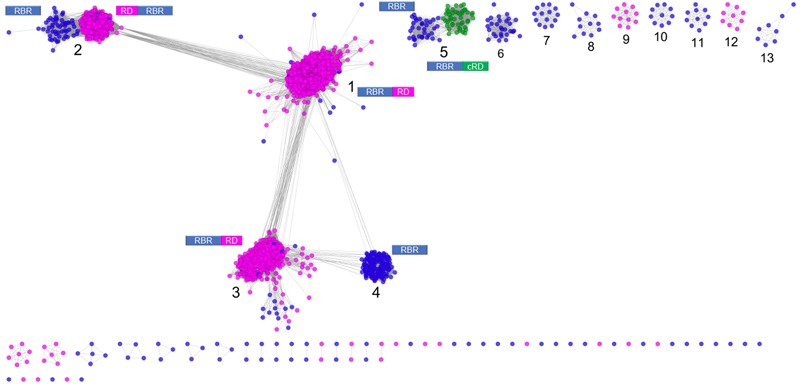
**Sequence similarity network for RBR.** The network was made from 2631 representative sequences (filtered by >90% identity) applying score data relationships in All-versus-All BLASTp (*E*-value < 10–45). Topology was generated in Cytoscape 3.0.2 using the *Prefuse forced directed* layout. The main clusters were assigned with numbers (see “Text” for more details). The domain architectures of the different forms of RBR are shown. Node colors indicate the type of rubredoxin-like domain where Magenta: “short-spaced” rubredoxin-like domain; Green: “long-spaced” rubredoxin-like domain; Blue: without any rubredoxin-like domain. Abbreviations: RBR, rubrerythrin; RD, rubredoxin-like domain; cRD, cyanobacterial rubredoxin-like domain.

**Figure 1** shows a sequence similarity network for the RBR orthologs retrieved from the NR database following the strategy outlined above. From this network (comprising 2631 representative genes), a set of node clusters or groups can be inferred. Four large groups (1–4) connected in a major network were recovered using an *E*-value threshold (*E* < 10^-45^). In addition, there are nine minor isolated groups (5–13), and several smaller groups with disconnected nodes, indicating that, at the BLAST *E*-value used, those sequences have no similarity with other members of the RBR protein set. Group 5 is included with groups 1–4 in subsequent analyses. Groups 1–5 comprise ∼93% of the sequences.

Group 1 (**Figure 1**) corresponds to the principal group of canonical rubrerythrins, and is comprised almost completely (98.7 % of 1336 sequences) by members with the rubredoxin-like domain with a relatively short spacing between the two C-x-x-C motifs (10–14 residues, we term “short-spaced”). Taxonomic information (**Supplementary Figure S1**) indicates that this group is dominated by members of *Clostridia* (44.5%) and δ/𝜀*-Proteobacteria* classes (11.0%) and the *Bacteroidetes* phylum (10.2%). Comparison with PDB database showed that the classical RBR protein from *D. vulgaris* (Swissprot accession P24931, PDB entry 1B71) is a member of this group. Therefore, we propose that group 1 is termed the “Classical RBR” group. The group is populated predominately by obligate anaerobic microorganisms.

Group 2 (**Figure 1**) consists of two sub-clusters: the first (85.3% of the total sequences) exhibits the rubredoxin domain as do group 1 rubredoxins, but unlike group 1 this rubredoxin domain is located in the N terminal of the protein as previously observed in *Clostridium acetobutylicum* (Riebe et al., 2009). The other sub-cluster (14.7% of the total sequences) does not have a rubredoxin domain. Group 2 was dominated obligate anaerobes such as members of the *Clostridia* class (64.5 %) and *Bacteroidetes* phylum (10.5%; see **Supplementary Figure S1**). We propose that group 2 be termed the “Reverse-type” RBR group.

Group 3 (**Figure 1**) is mainly composed of RBR with the C-terminal rubredoxin-like domain and, according to taxonomic information, is dominated by members of δ*/*𝜀-*Proteobacteria* (25.97%) and *Clostridia* (20.7%) classes and the *Euryarchaeota* phylum (17.31%). Comparison with PDB showed that a previously crystallized RBR from *Pyrococcus furiosus* belongs to this group (Uniprot accession Q9UWP7, PDB entry 1NNQ); this RBR contains canonical hydrogen peroxide reductase activity (Dillard et al., 2011). We propose that this group is termed the “Classical group B.” Like group 1, both the “reverse” (group 2) and the “classical-B” types of RBR (group 3) are enriched in taxonomic groups dominated by obligate anaerobes.

Group 4 (**Figure 1**) is composed exclusively of genes lacking the rubredoxin-like domain. This group is dominated by members of α/β/γ-Proteobacteria (61.1%), Actinobacteria (9.0%), and Crenarchaeota (including members of Sulfolobales and Thermoproteales orders, 8.3%), as well as bacterial members from Aquificae (including the ferriperoxin from *H. thermophilus*), Nitrospirae and some few members of Firmicutes, Deltaproteobacteria, and archaeal members of Thermoplasmatales (from the Euryarchaeota phylum). Unlike the three aforementioned groups, members of group 4 are dispersed in taxonomic groups dominated by (facultative) aerobic organisms. Sulerythrin from *S. tokodaii* (Uniprot accession F9VPE5; PDB entry 1J30) and a rubrerythrin from *Burkholderia pseudomallei* (Uniprot accession Q3JK2; PDB entry 4DI0) have three dimensional structures in the PDB. The presence of RBRs without the rubredoxin-like domain is distributed in different sequence clusters of this network, suggesting that these proteins arose independently more than once in evolution. The detection of a well-defined group composed only of members without the aforementioned domain, and additionally, that those homologs are mostly associated with aerobic organisms, strongly suggest that this group is a complete and distinctive evolutionary lineage with common properties and we term it the “aerobic-type” group of RBR.

Group 5 (**Figure 1**) contains two well-compartmentalized sub-clusters; one of them (comprising 41.8% of the sequences) lacks the rubredoxin-like domain. Members of the other sub-cluster (comprising the 58.2% of the sequences) is distinguished by having a C-terminal rubredoxin-like domain, with a longer spacing between the cysteines motifs (27–32 residues) compared with the C-terminal domain of the classical RBR (10–14 residues). Taxonomically, the group 5 is dominated by members of Cyanobacteria (59.5%) and α/β/γ-Proteobacteria (36.7%). The “long-spaced” rubredoxin-like domain is present in almost all cyanobacterial RBRs and has a different evolutionary origin from the other RBRs. Due to the prevalence of cyanobacterial RBR genes in group 5, we term this the “Cyanobacterial-type” RBR. Given their evolutionary properties, the phylogenetic analysis of this group, and the origin of the “long” rubredoxin domain will be covered below.

### Phylogenetic and Genomic Analyses of the “Aerobic-Type” RBR

**FIGURE 2:**
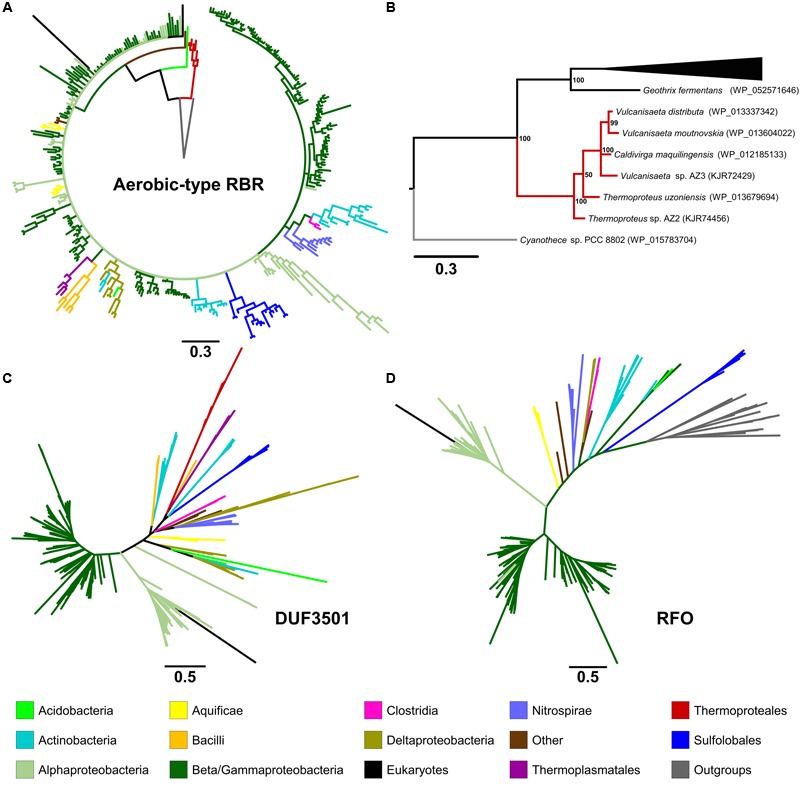
**Phylogenetic trees constructed in this study.** The circular tree for representative members of the “aerobic-type” RBR **(A)** and **(B)** with a phylogram representation of the closest clade to the root of the tree **(B)**. The figure also includes unrooted versions of the trees made from the protein sequences of DUF3501 **(C)** and RFO **(D)**. For the tree of the “aerobic-type” RBR group, the sequence of a RBR from *Cyanothece* sp. PCC 8802 (a cyanobacterial RBR) was used as an outgroup taxon, whereas the tree for RFO used the sequence of GlpC from *Escherichia coli* as outgroup. For each case, the line length represents the respective value for phylogenetic distance. Organism names and more detailed trees are displayed in **Supplementary Figures S2**–**S5**.

In order to analyze the evolutionary history of this proposed lineage, a phylogenetic tree was constructed using BI (**Figure 2A** in summary and **Supplementary Figure S2** in detail). The “aerobic-type” RBR phylogenetic tree (using an appropriate outgroup) showed that the closest clade to the root of the group comprises a set of orthologs from archaeal members (**Figure 2B** in summary and **Supplementary Figure S2** in detail) from the *Vulcanisaeta, Caldivirga*, and *Thermoproteus* genera of the Thermoproteales order (Itoh et al., 1999; Mavromatis et al., 2010; Gumerov et al., 2011; Mardanov et al., 2011). All of the extant members of this group are hyperthermophiles and are slightly acidophilic (pH 3.0–6.5). They grow only in anaerobic or microaerobic conditions. For example, a well-supported clade contains orthologs from members of Actinobacteria (species from *Acidithrix, Ferrimicrobium*, and other related groups), Nitrospirae (species from *Leptospirillum* and *Nitrospira*), and Clostridia (*Sulfobacillus*), associated with extremely acidic (pH < 3) environments (Cardenas et al., 2016). Another well-supported clade contains only orthologs from members of the Sulfolobales order (from *Sulfolobus, Metallosphaera*, and *Acidianus* genera). It is interesting to note that this clade is paraphyletic with respect to other clades containing other orthologs from Archaea (for example, the clades containing orthologs from Thermoproteales or Thermoplasmatales). This suggests that the evolutionary history of this lineage of RBR involved multiple different horizontal transferences within archaeal organisms. Another noticeable observation inferable from this tree is the expansion of this RBR lineage inside in the Alpha-, Beta- and Gammaproteobacteria. Most importantly, the phylogenetic tree of RBR suggests that the most ancient group (the closest to the root) is composed of members from the Thermoproteales; this is consistent with the idea that this form of RBR evolved in a microaerobic and thermophilic environment.

### Gene Context Analyses of Genes/Domains Predicted to Be Related to “Aerobic-Type” RBR Function

**FIGURE 3:**
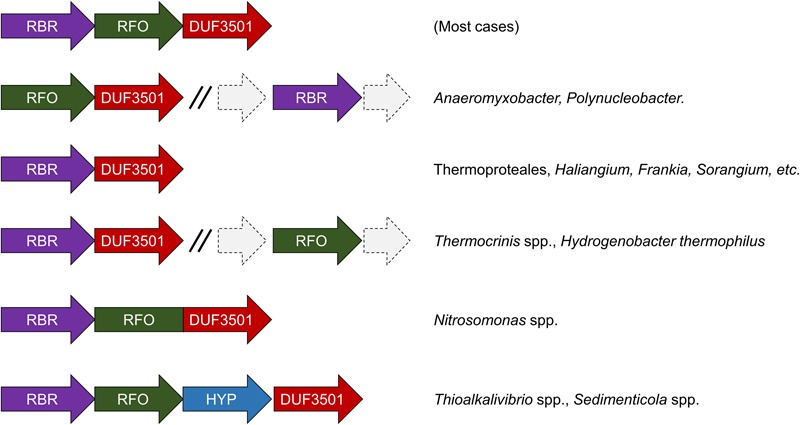
**Genomic neighborhoods of “aerobic” RBR in sequenced genomes.** RBR, aerobic-type rubrerythrin; DUF3501, conserved protein of unknown function; RRO, Fe-S oxidoreductase; HYP, hypothetical protein.

We have undertaken an analysis of the gene contexts of the “aerobic-type” of RBR in order to shed light on its evolution. A comparative visualization of RBR gene neighborhoods among complete genomes was carried out using the IMG-JGI database (Markowitz et al., 2012). The gene neighborhood comparison (**Figure 3**) showed that the majority of the RBR genes from the “aerobic-type” were co-localized in a conserved gene cluster with a gene encoding a protein of unknown function (DUF3501, pfam12007), and in several other cases, also co-localized with a gene predicted to encode a member of the COG0247 family (*Fe-S oxidoreductase*). The role of the DUF3501 protein is unknown. However, the function of the COG0247 family protein has been suggested to be related to electron transfer inside protein complexes, since it contains a conserved CCG domain (pfam02754) and a cysteine rich domain. The latter is present in several oxidoreductase complexes related to energy metabolism under anaerobic conditions such as subunit GlpC in the anaerobic glycerol-3-phosphate dehydrogenase (Cole et al., 1988) and subunits of the HdrD/E from the heterodisulfide reductase complex found in methanogens (Kunkel et al., 1997). We propose that this COG0247 family protein member associated with RBR be termed RFO (Rubrerythrin-associated Fe-S Oxidoreductase). In some instances, DUF3501- and RFO are fused into one gene (e.g., *Nitrosomonas* spp., **Figure 3**). In other organisms (*Thermocrinis* spp. and *H. thermophilus*), the RFO-encoding gene is separated from the other two genes. It is interesting to note that other members of the deeply rooted Aquificae phylum (such as *Hydrogenobaculum*) have the more common three-gene arrangement. This difference in gene cluster structure among members of the same phylum may be a reflection of the antiquity of the events that resulted in these different gene arrangements.

Additional comparisons suggest that the genes encoding DUF3501 and RFO are not only strongly co-localized with the “aerobic-type” RBR, but also are co-occurrent. An examination of a set of 4461 completed prokaryotic genomes from the IMG-JGI database showed that DUF3501 and RFO genes are not associated with RBR in only one organism (*Burkholderia cepacia* AMMD) and only in one other genome (*Azoarcus* sp. CIB) a DUF3501-encoding gene was detected without the other two, RFO and RBR (Supplementary Table S2). The exclusive co-occurrence of RBR – DUF3501 was found in 25 organisms, and the triple co-occurrence of RBR, DUF3501, and RFO was detected in 185 genomes. Interestingly, the co-occurrence of RBR – DUF3501 was detected in the members of Thermoproteales, suggesting that this association could be as ancient as the last common ancestor of this group, supporting the contention that the origin of “aerobic-type” RBR lineage, occurred in a high-temperature and microaerobic environment.

### Phylogenetic Analysis of DUF3501 and RFO Families

In order to investigate the possible co-evolution of the “aerobic-type” RBR with the DUF3501 and RFO genes, phylogenetic trees for DUF3501 (**Figure 2C** in summary and **Supplementary Figure S3** in detail) and RFO (**Figure 2D** in summary and **Supplementary Figure S4** in detail) were constructed. These trees are consistent with the late expansion of the respective protein families into the Alpha-, Beta- and Gammaproteobacteria, and with the observed expansion of the co-localizing “aerobic-type” RBR. The case of DUF3501 also showed the non-monophyletic association between the archaeal orthologs from the three different orders (Thermoproteales, Sulfolobales, and Thermoplasmatales), seen previously in the RBR tree. In the case of the RFO tree, BLAST searches of orthologs for this phylogenetic tree showed that the most closely related proteins are GlpCs that encode the subunit C of the anaerobic glycerol-3-phosphate dehydrogenase (involved in glycerol degradation under anaerobic conditions). The application of GlpC as an outgroup showed that the most ancient group corresponds to Sulfolobales, an archaeal order composed of aerobic thermoacidophiles (Huber and Stetter, 2015), suggesting that both GlpC as well as the “aerobic-type” RBR, arose in high-temperature aerobic environments. Both phylogenetic trees for DUF3501 and RFO supporting the contention of the co-evolution of DUF3501 and RFO with the “aerobic-type” RBR.

### The “Cyanobacterial Group”: A Parallel Adaptation of Rubrerythrin to an Aerobic Environment

Information obtained from phylogenomic analyses of the “aerobic-type” RBR and its co-occurrent genes suggests an evolutionary trajectory driven by the adaptation to aerobic environments, initiated from an (hyper)thermophilic ancestor. However, another group in the RBR sequence similarity network was also detected (the “cyanobacterial group,” group 5 from **Figure 1**) that was not connected to the other four groups and was composed mostly of members from Cyanobacteria (phototrophic oxygen-producers) and α/β/γ-Proteobacteria (facultative anaerobes), raising the question about how this protein group evolved. A phylogenetic tree for members of the “cyanobacterial RBR” group was made using ML (**Supplementary Figure S5**). This analysis showed two well-defined clades: a clade containing the cyanobacterial protein and a clade containing the proteobacterial orthologs. The members of the cyanobacterial clade contained a fusion of the FLSF of RBR with a “long-spaced” rubredoxin domain (data not shown). Based on the well-defined phylogenetic bifurcation and the coherent pattern of inheritance, the most parsimonious explanation is that the original RBR ancestor for this group was a one-domain RBR (as the “aerobic-type” RBR is), but in Cyanobacteria, the protein was fused with the “long-spaced” rubredoxin domain. We hypothesize that this adaptation had a relationship with the exposure to oxygen associated with the development of oxygenic photosynthesis in Cyanobacterial ancestor during GOE.

### Evolution of the “Aerobic” and “Cyanobacterial” Rubrerythrins in the Early Earth: A Model

**FIGURE 4:**
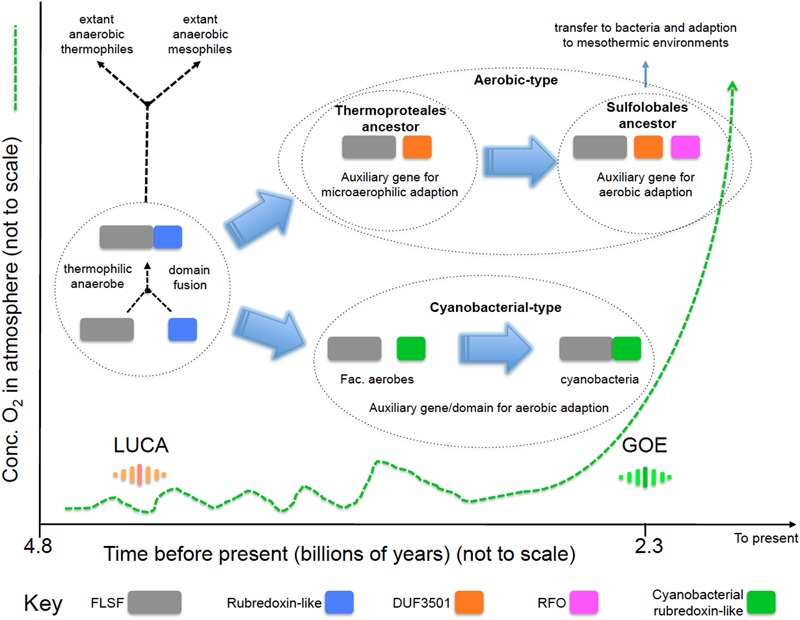
**Proposed model of the evolution of the “aerobic-type” RBR and the “cyanobacterial group” RBRs.** Phylogenomic evidence suggests that the “aerobic-type” RBR emerged in microaerophilic and hyperthermophilic organisms and evolved into a form adapted to aerobic environments via the acquisition of two co-localizing genes, termed DUF3501 and RFO. In parallel, the “cyanobacterial group” of RBR evolved from (facultative) anaerobes undergoing a separate adaptation process in Cyanobacteria when atmospheric oxygen levels increased. The small fluctuations in oxygen before GOE represent “whiffs” of oxygen. Abbreviations: FLSF, ferritin-like superfamily; LUCA, the Last Universal Common Ancestor; GOE, Great Oxygenation Event; DUF, Domain of Unknown Function; RFO, Rubrerythrin-associated Fe-S Oxidoreductase.

We propose a model (**Figure 4**) for the evolution of the “aerobic” and “cyanobacterial” rubrerythrins that is consistent with evidence provided by network information, phylogenetic trees and gene/domain contexts of the different forms of rubrerythrins. It is suggested that the “classical” form of rubrerythrin arose by gene fusion of a FLSF domain and a rubredoxin-like domain in a hot and anaerobic environment. This form is predicted to have existed in LUCA (Weiss et al., 2016). Extant obligate thermophilic and mesophilic anaerobes retain this form today [e.g., *Thermotoga maritima* (Lakhal et al., 2011) and *D. vulgaris* (Lumppio et al., 1997), respectively].

Two separate lines of evolution then occurred. In the first, that is postulated to have occurred in the ancestors of the thermophilc Archaeal group Thermoproteales, we hypothesize that the loss of the rubredoxin-like domain was compensated for by the acquisition of DUF3051. This association is proposed to have provided protection in microaerobic environments that might have arisen before GOE due to exposure to low levels of oxygen. These low levels and perhaps transitory increments of oxygen could have originated from biotic or abiotic reactions, such as enzymatic ROS-detoxifying reactions or water UV photolysis (Martin and Sousa, 2016). It is probable that during those “oxygen whiffs,” many proteins would be the object of selection pressure to survive in oxygen that could force them to lose or gain new features such as protein domains (Nasir et al., 2014) or physiological activities. In addition, the ancient hot environment of the Archaean eon could helped to accelerate the evolutionary rate of change of these proteins, as postulated to be occurring in current extreme environments (Li et al., 2014). Subsequently, an additional gene, RFO, was added that provided full function in aerobic environments perhaps resulting from GOE. This step is proposed to have occurred in the ancestors of the thermophilc Archaea Sulfolobales. This triple gene form was passed by HGT to the Bacteria and underwent further adaption to work in mesothermic conditions (e.g., <40°C). It is not known why the additional DUF3051 and RFO genes are not found fused as domains to the FLSF domain but rather they remain as conserved co-localizing but separate genes. Perhaps this architecture provides more opportunities for differential expression of the genes.

The second line, involved the replacement of the rubredoxin-like domain with a separately evolved cyanobacterial rubredoxin-like domain. It is hypothesized that this promoted the function of RBR in high levels of oxygen resulting from oxygenic photosynthesis that is thought to have evolved in the cyanobacterial lineage about the time of GOE.

Oxidative stress is an ancient and widespread phenomenon: several of the extant obligate anaerobes exhibit mechanisms to combat the presence of ROS (Jenney et al., 1999). One of those mechanisms is the use of RBR. This protein has been suggested to be an ancient protein (Andrews, 2010) predicted to be present in LUCA that was itself hypothesized to be an obligate anaerobe and thermophile (Martin and Sousa, 2016). The proposed antiquity of RBR makes it an interesting protein to be analyzed, establishing a case study of the effect of the arise of oxygen in the adaption and origin of new protein (sub)families. It is interesting to note that the vast majority of the known RBRs have the “short-spaced” rubredoxin-like domain, even in a “reverse” form (**Figure 1**). This strongly suggest that, regardless that the very last common ancestor of all rubrerythrins lacked this rubredoxin domain, this fusion was implemented very early in evolution, and maybe even in different independent events (e.g., generating the “classic” and “reverse” RBR separately), and those successful, two-domain RBRs have been inherited by extant aerobic microorganisms.

There is phylogenomic evidence of ancient, massive gene birth events. Large-scale phylogenetic reconstruction of more than 3,000 gene families predicted a massive event of birth and loss of gene families occurring approximately 3.3–2.9 × 10^9^ years ago (David and Alm, 2011), coinciding with or perhaps preceding, with the estimated time of GOE (Bekker, 2014). Another study (using a similar strategy) suggests that aerobic metabolism appeared about 2.9 × 10^9^ years ago (Wang et al., 2011). In that context, it is probable that this “aerobic-type” lineage of RBR, as well as their co-occurrent genes, could arise before GOE or just as GOE was starting. Regarding the case of the “cyanobacterial group” of RBR, the evidence provided by the sequence similarity network (group 5, **Figure 1**) suggests that this group of RBRs has a different evolutionary pathway from the “aerobic-form.”

It is interesting to note that the 3-genes form of RBR was most likely to have evolved in the ancestors of two archaeal orders: Thermoproteales and Sulfolobales. Both those two taxonomic orders belong to the Crenarchaeota phylum, and have a proposed ancient point of divergence (Gribaldo and Brochier-Armanet, 2006). Whereas DUF3501 may have arisen in temperatures over 85° C (temperature associated with the lifestyle of extant members of Thermoproteales), the RFO gene could have arisen in temperatures between 60–80°C (associated with the lifestyle of extant Sulfolobales). We hypothesize that the acquisition of DUF3501 was part of an adaptation for function in combined microaerobic and hyperthermic conditions, whereas RFO evolving in direct response to increasing oxygen presence. One observation, consistent with this supposition, is that the RFO gene is not found in microaerobic organisms, such as members of Thermoproteales and some members of Deltaproteobacteria.

We suggest that, since RFO belongs to a group of proteins potentially involved in electron transfer, its linkage with the “aerobic-type” RBR can compensate for the loss of the rubredoxin-like domain, a component of the anaerobic RBRs that has a proposed role in electron transfer during catalysis (Kurtz, 2006). However, this also raises another question: how is the catalysis of electron transfer in the RBR form found in Thermoproteales, where DUF3501 but not RFO is present? This question remains to be experimentally addressed.

The proposed origin and evolution of the “aerobic-type” RBR is consistent with the hypothesis that early life evolved in a hot, anaerobic environment (Martin and Sousa, 2016). It also supports the idea that oxidative stress mechanisms could have been present before GOE. It also provides an exquisite opportunity to study the evolutionary trajectory of proteins as they adapt to increasing oxygen levels and to econiches with lower temperatures.

## Author Contributions

JC, RQ, and DH conceived the project. JC designed and carried out the experiments. All authors analyzed the data. JC wrote the first draft of the manuscript. All authors contributed to other drafts of the manuscript. All authors read and approved the final manuscript.

## Conflict of Interest Statement

The authors declare that the research was conducted in the absence of any commercial or financial relationships that could be construed as a potential conflict of interest.

## Supplementary Material

FIGURE S1 **Phylogenetic distribution of rubrerythrin (RBR) types belonging to groups 1–5 (see “Text” of article).** Sequences were retrieved from the network shown in **Figure 1** and were sorted by taxonomic origin (Phylum or Class) and counted.

FIGURE S2 **Phylogenetic distribution of the “aerobic” group of RBRs.** The tree was elaborated as specified in “Material and Methods.” Each taxon is tagged with an NCBI Accession number shown in parentheses.

FIGURE S3 **Phylogenetic distribution of members of the DUF3501 protein family.** The tree was elaborated as specified in “Material and Methods.” The branch support value is symbolized by the color of each node circle (color gradient legend shown). This tree is unrooted. Each taxon is tagged with an NCBI Accession number shown in parentheses.

FIGURE S4 **Phylogenetic distribution of members of the RFO protein family.** The tree was elaborated as specified in “Material and Methods.” The branch support value is symbolized by the color of each node circle (color gradient legend shown). This tree was rooted using the sequence of GlpC from *Escherichia coli* as an outgroup. Each taxon is tagged with an NCBI Accession number shown in parentheses.

FIGURE S5 **Phylogenetic distribution of members of the “cyanobacterial group” of RBRs.** The tree was elaborated as specified in “Material and Methods.” The branch support value is symbolized by the color of each node circle (color gradient legend shown). In the base of each great clade, the domain fusion/separation is specified. This tree was rooted using the sequence of an “aerobic-type” RBR derived from *Vulcanisaeta distributa* as an outgroup. Each taxon is tagged with an NCBI Accession number shown in parentheses. Abbreviation: RB, rubredoxin.

TABLE S1 **List of rubrerythrin (RBR) sequences found in NR database.** The table contains the NCBI accessions, protein length, and description for each sequence.

TABLE S2 **List of candidate aerobic rubrerythrins, DUF3501 and RFO in prokaryotic finished genomes from IMG-JGI database.** This table includes a Venn diagram displaying the co-occurrence of these genes.
