# Supplementary figures and images for: Aerobic Lineage of the Oxidative Stress Response Protein Rubrerythrin Emerged in an Ancient Microaerobic, (Hyper)Thermophilic Environment
Source: Front Microbiol. 2016 Nov 18;7:1822. doi: 10.3389/fmicb.2016.01822 (PMC5114695; doi:10.3389/fmicb.2016.01822)

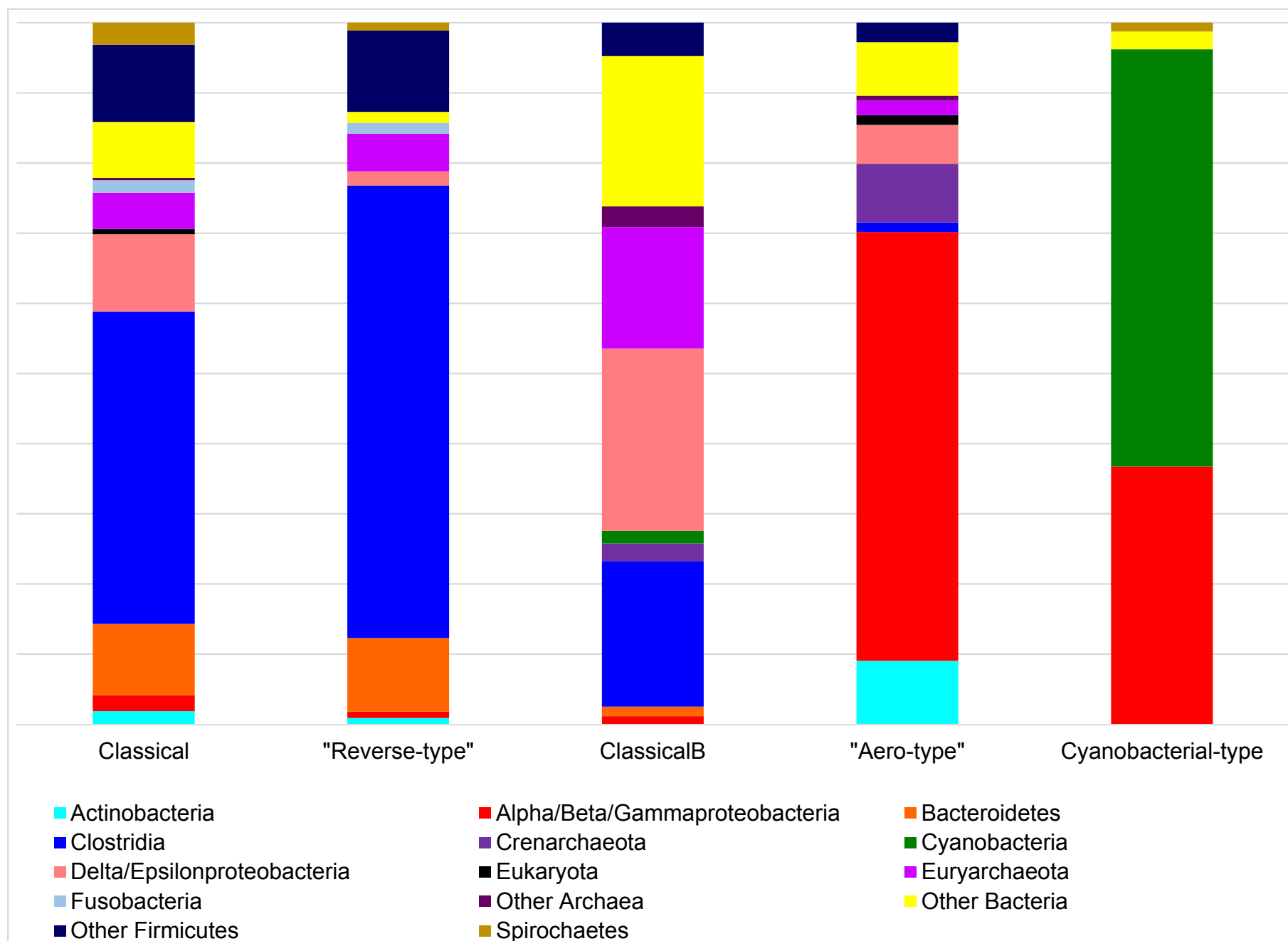

Supplement: FIGURE S1 — Phylogenetic distribution of rubrerythrin (RBR) types belonging to groups 1–5 (see “Text” of article). Sequences were retrieved from the network shown in Figure 1 and were sorted by taxonomic origin (Phylum or Class) and counted. [file Figure_S1.pdf]

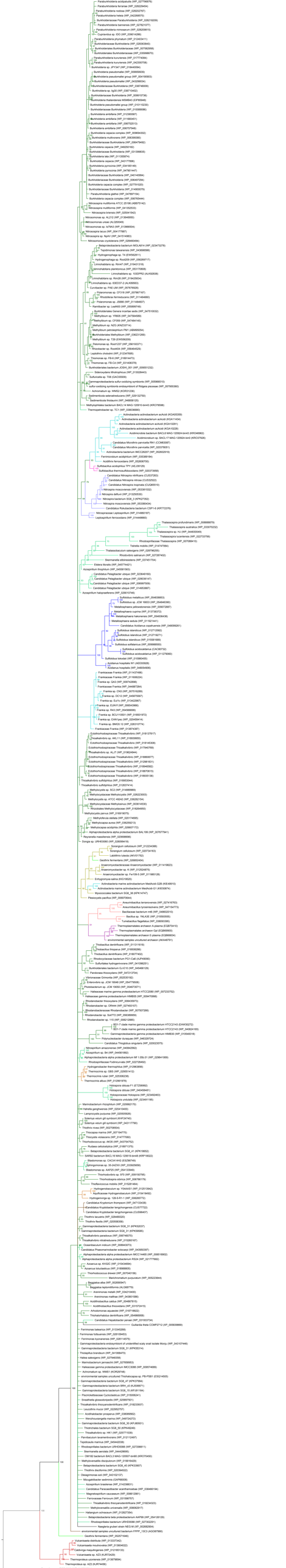

Supplement: FIGURE S2 — Phylogenetic distribution of the “aerobic” group of RBRs. The tree was elaborated as specified in “Material and Methods.” Each taxon is tagged with an NCBI Accession number shown in parentheses. [file Figure_S2.pdf]

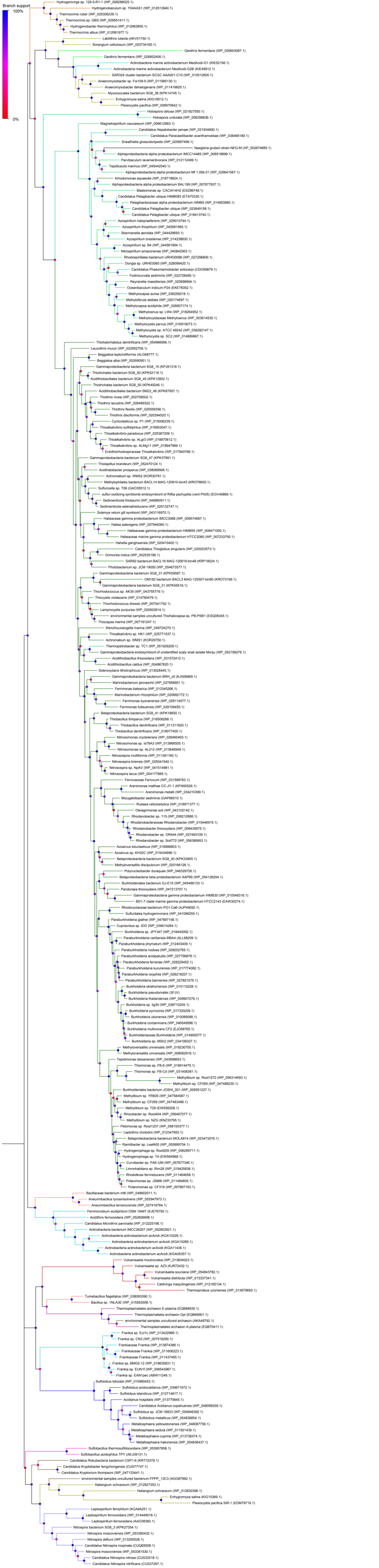

Supplement: FIGURE S3 — Phylogenetic distribution of members of the DUF3501 protein family. The tree was elaborated as specified in “Material and Methods.” The branch support value is symbolized by the color of each node circle (color gradient legend shown). This tree is unrooted. Each taxon is tagged with an NCBI Accession number shown in parentheses. [file Figure_S3.pdf]

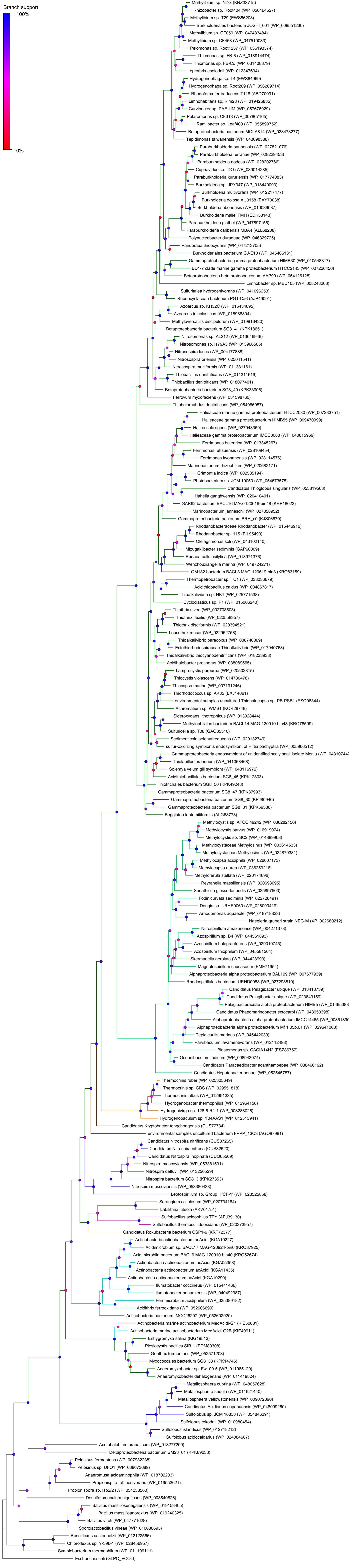

Supplement: FIGURE S4 — Phylogenetic distribution of members of the RFO protein family. The tree was elaborated as specified in “Material and Methods.” The branch support value is symbolized by the color of each node circle (color gradient legend shown). This tree was rooted using the sequence of GlpC from Escherichia coli as an outgroup. Each taxon is tagged with an NCBI Accession number shown in parentheses. [file Figure_S4.pdf]

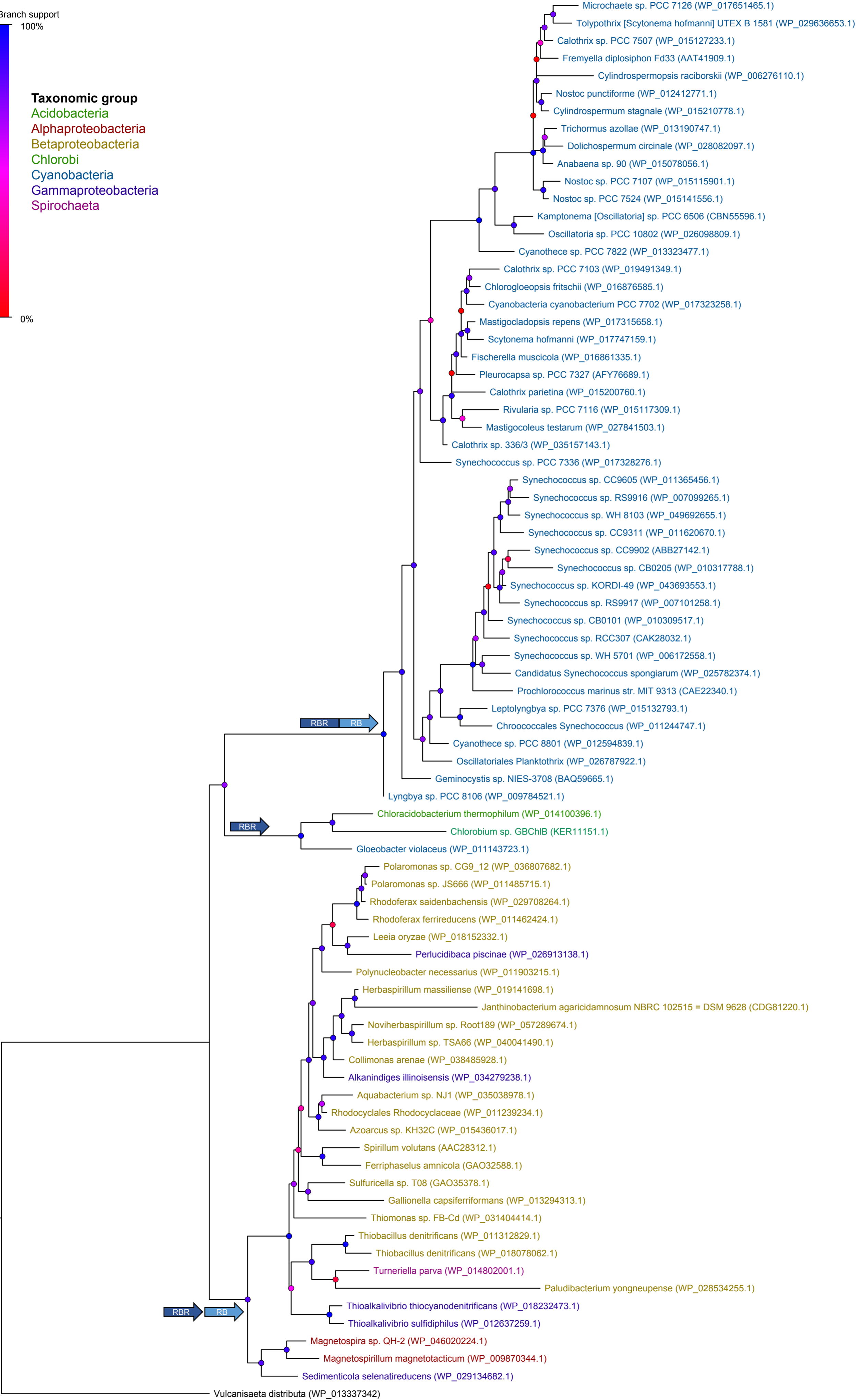

Supplement: FIGURE S5 — Phylogenetic distribution of members of the “cyanobacterial group” of RBRs. The tree was elaborated as specified in “Material and Methods.” The branch support value is symbolized by the color of each node circle (color gradient legend shown). In the base of each great clade, the domain fusion/separation is specified. This tree was rooted using the sequence of an “aerobic-type” RBR derived from Vulcanisaeta distributa as an outgroup. Each taxon is tagged with an NCBI Accession number shown in parentheses. Abbreviation: RB, rubredoxin. [file Figure_S5.pdf]
