# Supplementary material for: Aerobic Lineage of the Oxidative Stress Response Protein Rubrerythrin Emerged in an Ancient Microaerobic, (Hyper)Thermophilic Environment
Source: Front Microbiol. 2016 Nov 18;7:1822. doi: 10.3389/fmicb.2016.01822 (PMC5114695; doi:10.3389/fmicb.2016.01822)
Supplement: TABLE S1 — List of rubrerythrin (RBR) sequences found in NR database. The table contains the NCBI accessions, protein length, and description for each sequence. [file Table_S1.pdf]

ACCESSION	LENGTH	DESCRIPTION
WP_052399336.1	1081	hypothetical protein [Candidatus Methanoplasma termitum]
WP_024614503.1	741	iron hydrogenase [Clostridium sp. Ade.TY]
WP_009004329.1	721	iron hydrogenase [Clostridium sp. D5]
WP_047774150.1	721	iron hydrogenase [[Eubacterium] fissicatena]
WP_049728956.1	720	iron hydrogenase [Dorea sp. D27]
WP_006442921.1	719	iron hydrogenase [[Clostridium] hylemonae]
WP_050642365.1	717	iron hydrogenase [Eubacterium sp. SB2]
WP_055153265.1	717	iron hydrogenase [[Eubacterium] contortum]
WP_004607406.1	708	iron hydrogenase [[Clostridium] scindens]
WP_025641998.1	708	iron hydrogenase [[Clostridium] scindens]
WP_009248230.1	708	iron hydrogenase [Lachnospiraceae bacterium 5_1_57FAA]
WP_008982139.1	696	iron hydrogenase [Ruminococcaceae bacterium D16]
WP_004460591.1	696	iron hydrogenase [Clostridium perfringens]
WP_004459025.1	696	iron hydrogenase [Clostridium perfringens]
WP_003480816.1	696	iron hydrogenase [Clostridium perfringens]
WP_011590552.1	696	iron hydrogenase [Clostridium perfringens]
WP_003460372.1	696	iron hydrogenase [Clostridium perfringens]
ALG48395.1	696	"(Fe) hydrogenase, cytoplasmic, one subunit form[Clostridium perfringens]"
WP_003451814.1	696	iron hydrogenase [Clostridium perfringens]
WP_003461974.1	696	iron hydrogenase [Clostridium perfringens]
WP_004457171.1	696	iron hydrogenase [Clostridium perfringens]
WP_003477508.1	696	iron hydrogenase [Clostridium perfringens]
WP_049040149.1	696	iron hydrogenase [Clostridium perfringens]
WP_057257832.1	696	iron hydrogenase [Clostridium perfringens]
ABG85712.1	696	[Fe] hydrogenase [Clostridium perfringens SM101]
CEP99940.1	694	[Fe] hydrogenase [[[Clostridium] sordellii]
CEP79688.1	694	[Fe] hydrogenase [[Clostridium] sordellii]
CEN77532.1	694	[Fe] hydrogenase [[Clostridium] sordellii]
CEO09741.1	694	[Fe] hydrogenase [[Clostridium] sordellii]
WP_057538354.1	694	iron hydrogenase [[Clostridium] sordellii]
WP_057557298.1	694	iron hydrogenase [[Clostridium] sordellii]
WP_057544475.1	694	iron hydrogenase [[Clostridium] sordellii]
WP_053984510.1	692	iron hydrogenase [Lachnospiraceae bacterium mt14]
CEP90568.1	691	[Fe] hydrogenase [[Clostridium] sordellii]
WP_057561389.1	690	iron hydrogenase [[Clostridium] sordellii]
WP_057543543.1	690	iron hydrogenase [[Clostridium] sordellii]
WP_057546480.1	690	iron hydrogenase [[Clostridium] sordellii]
WP_054630111.1	690	iron hydrogenase [[Clostridium] sordellii]
WP_057567825.1	690	iron hydrogenase [[Clostridium] sordellii]
WP_057570429.1	690	iron hydrogenase [[Clostridium] sordellii]
CEK34946.1	690	"[Fe] hydrogenase,Iron hydrogenase 1,Iron only hydrogenase large subunit, C-terminal domain,[FeFe] hydrogenase, group A,Iron only hydrogenase large subunit, C-terminal domain

[[[Clostridium]sordellii]"
CEP42209.1   690   [Fe] hydrogenase [[[Clostridium] sordellii]
WP_057569238.1 690   iron hydrogenase [[Clostridium] sordellii]
WP_057579217.1 690   iron hydrogenase [[Clostridium] sordellii]
WP_057563930.1 690   iron hydrogenase [[Clostridium] sordellii]
WP_057541720.1 690   iron hydrogenase [[Clostridium] sordellii]
WP_057583545.1 690   iron hydrogenase [[Clostridium] sordellii]
WP_057535698.1 690   iron hydrogenase [[Clostridium] sordellii]
WP_057549039.1 690   iron hydrogenase [[Clostridium] sordellii]
WP_057540539.1 690   iron hydrogenase [[Clostridium] sordellii]
WP_021128815.1 690   [FeFe] hydrogenase group A family
protein[[Clostridium] sordellii]
WP_057550056.1 690   iron hydrogenase [[Clostridium] sordellii]
WP_057585955.1 690   iron hydrogenase [[Clostridium] sordellii]
WP_057566798.1 690   iron hydrogenase [[Clostridium] sordellii]
WP_057575739.1 690   iron hydrogenase [[Clostridium] sordellii]
WP_057554456.1 690   iron hydrogenase [[Clostridium] sordellii]
WP_035117908.1 686   iron hydrogenase [Clostridium sp. NCR]
WP_024620034.1 686   iron hydrogenase [[Clostridium] bifermentans]
WP_021431890.1 686   [FeFe] hydrogenase group A family
protein[[Clostridium] bifermentans]
WP_021429872.1 686   [FeFe] hydrogenase group A family
protein[[Clostridium] bifermentans]
WP_053832585.1 686   iron hydrogenase [Candidatus Dorea
massiliensis]
WP_027639472.1 685   iron hydrogenase [Clostridium cadaveris]
WP_040565074.1 683   iron hydrogenase [Phascolarctobacterium
succinatutens]
CDD10809.1   683   rubredoxin [Phascolarctobacterium
succinatutens CAG:287]
WP_005436344.1 683   iron hydrogenase [Sutterella wadsworthensis]
EEG52537.1   683   rubredoxin [[[Clostridium asparagiforme] DSM
15981]
WP_016148395.1 682   [FeFe] hydrogenase group A [Butyricicoccus
pullicaecorum]
CDE76601.1   682   hydrogenase [Sutterella sp. CAG:521]
WP_003500375.1 682   MULTISPECIES: iron hydrogenase [Clostridiales]
WP_044902140.1 682   iron hydrogenase [[Clostridium] symbiosum]
KSV59732.1   681   iron hydrogenase [Acetivibrio ethanolgignens]
WP_023050767.1 681   hypothetical protein [Cetobacterium somerae]
WP_005981915.1 680   iron hydrogenase [Fusobacterium ulcerans]
WP_008695770.1 680   iron hydrogenase [Fusobacterium ulcerans]
WP_033139544.1 679   iron hydrogenase [Blautia producta]
WP_027128738.1 679   iron hydrogenase [Fusobacterium perfoetens]
WP_041137825.1 678   iron hydrogenase [Clostridiaceae bacterium
GM1]
WP_005884626.1 678   iron hydrogenase [Fusobacterium mortiferum]
ERI74480.1   677   rubredoxin [[[Clostridium] symbiosum ATCC
14940]
WP_040412269.1 675   iron hydrogenase [[Clostridium] asparagiforme]

WP_024736187.1 675   iron hydrogenase [Clostridiales bacterium VE202-15]
WP_009288921.1 650   iron hydrogenase [Anaerostipes sp. 3_2_56FAA]
CDC38750.1   650   iron only hydrogenase large subunit domain-containing protein [Anaerostipes sp. CAG:276]
WP_006566021.1 650   iron hydrogenase [Anaerostipes caccae]
EFY04859.1   641   rubredoxin [Phascolarctobacterium succinatutens YIT 12067]
WP_009988159.1 447   rubrerythrin [Ruminococcus flavefaciens]
WP_028521319.1 446   rubrerythrin [Ruminococcus flavefaciens]
WP_028518472.1 445   rubrerythrin [Ruminococcus flavefaciens]
WP_024859961.1 443   rubrerythrin [Ruminococcus flavefaciens]
KJU84227.1   440   Metallophosphoesterase domain protein [Candidatus Magnetobacterium bavaricum]
ENZ02401.1   435   reverse rubrerythrin-1 [Clostridium colicanis 209318]
WP_033141937.1 424   hypothetical protein [Blautia producta]
WP_029469677.1 424   hypothetical protein [Clostridiales bacterium VE202-06]
WP_018595037.1 424   hypothetical protein [Blautia producta]
WP_044937201.1 421   hypothetical protein [Blautia schinkii]
WP_052567099.1 419   hypothetical protein [Candidatus Magnetobacterium casensis]
WP_016297126.1 419   hypothetical protein [Lachnospiraceae bacterium M18-1]
WP_004070369.1 419   hypothetical protein [Clostridium sp. ASF502]
WP_002601872.1 419   hypothetical protein [Hungatella hathewayi]
WP_051364555.1 419   hypothetical protein [Hungatella hathewayi]
ABZ08910.1   416   putative integral membrane protein DUF125 [uncultured marine microorganism HF4000_APKG5H11]
CCY53975.1   399   rubredoxin [Coprococcus sp. CAG:782]
CDD68526.1   398   putative uncharacterized protein [Firmicutes bacterium CAG:475]
ADL05267.1   396   Rubrerythrin [[[Clostridium] saccharolyticum WM1]
WP_026648213.1 393   rubrerythrin [Blautia wexlerae]
WP_055225691.1 393   rubrerythrin [Fusicatenibacter saccharivorans]
WP_055221665.1 393   rubrerythrin [Fusicatenibacter saccharivorans]
WP_055267390.1 393   rubrerythrin [Fusicatenibacter saccharivorans]
WP_055215535.1 393   rubrerythrin [Dorea longicatena]
WP_008402258.1 393   rubrerythrin [Clostridium sp. L2-50]
WP_055238331.1 393   rubrerythrin [Agathobacter rectalis]
WP_055650114.1 393   rubrerythrin [Hungatella hathewayi]
WP_028087396.1 393   rubrerythrin [Dorea longicatena]
WP_006428685.1 393   rubrerythrin [Dorea longicatena]
WP_023920583.1 393   hypothetical protein [Ruminococcus lactaris]
WP_055288773.1 393   rubrerythrin [Eubacterium ramulus]
WP_055061830.1 393   rubrerythrin [Agathobacter rectalis]
WP_015516632.1 393   rubrerythrin [Agathobacter rectalis]
CDD47747.1   393   rubredoxin [Firmicutes bacterium CAG:534]

WP_055151679.1	393	rubrerythrin [Blautia wexlerae]
WP_019161200.1	393	MULTISPECIES: rubrerythrin [Clostridiales]
CCX90872.1	393	rubredoxin [Succinatimonas sp. CAG:777]
WP_055282054.1	393	rubrerythrin [Dorea longicatena]
CCY58482.1	393	rubredoxin [Clostridium sp. CAG:632]
WP_005805542.1	392	MULTISPECIES: rubrerythrin [Candidatus Arthromitus]
CDF12809.1	392	rubrerythrin [Eubacterium sp. CAG:581]
ACL20268.1	392	Rubrerythrin [Desulfitobacterium hafniense DCB-2]
CDX01070.1	392	Rubrerythrin-1 [Desulfitobacterium hafniense]
CDB99452.1	391	rubredoxin [Firmicutes bacterium CAG:41]
AHF25366.1	388	rubrerythrin [uncultured bacterium Contig1625]
AHF25446.1	387	rubrerythrin [uncultured bacterium Contig1586]
CCY04765.1	381	rubrerythrin [Faecalibacterium sp. CAG:1138]
CDD68787.1	380	rubrerythrin [Firmicutes bacterium CAG:475]
EHQ91664.1	350	rubrerythrin [Desulfosporosinus youngiae DSM 17734]
ABD06030.1	345	conserved hypothetical protein [Rhodopseudomonas palustris HaA2]
EPZ55216.1	338	rubrerythrin family protein [[[Clostridium] sordellii ATCC 9714]
ALO98679.1	335	Rubrerythrin [Streptomyces hygroscopicus subsp. limoneus]
WP_021739596.1	331	"rubredoxin, partial [Eubacterium ramulus]"
ABJ08081.1	327	Rubrerythrin [Rhodopseudomonas palustris BisA53]
AFV12528.1	326	nigerythrin [Thermacetogenium phaeum DSM 12270]
WP_014498402.1	323	membrane protein [Bradyrhizobium japonicum]
WP_038957397.1	323	rubrerythrin [Bradyrhizobium japonicum]
WP_014439516.1	323	membrane protein [Bradyrhizobium sp. S23321]
WP_012046937.1	323	membrane protein [Bradyrhizobium sp. BTAi1]
WP_028157258.1	323	rubrerythrin [Bradyrhizobium japonicum]
WP_057746251.1	323	rubrerythrin [Bradyrhizobium manausense]
WP_011474509.1	323	membrane protein [Rhodopseudomonas palustris]
WP_039156005.1	323	rubrerythrin [Bradyrhizobium japonicum]
WP_008136776.1	323	membrane protein [Bradyrhizobium sp. YR681]
WP_027562058.1	323	MULTISPECIES: rubrerythrin [Bradyrhizobium]
WP_024510873.1	323	rubrerythrin [Bradyrhizobium sp. ARR65]
WP_008960072.1	323	MULTISPECIES: membrane protein [Bradyrhizobium]
WP_006611314.1	323	membrane protein [Bradyrhizobium sp. ORS 285]
WP_009027621.1	323	membrane protein [Bradyrhizobium sp. ORS 375]
WP_024338942.1	323	MULTISPECIES: rubrerythrin [Bradyrhizobium]
WP_028149248.1	323	rubrerythrin [Bradyrhizobium japonicum]
WP_038933625.1	323	rubrerythrin [Bradyrhizobium japonicum]
WP_006019946.1	323	hypothetical protein [Afipia broomeae]
WP_015669745.1	323	hypothetical protein [Bradyrhizobium oligotrophicum]

WP_022723851.1	323	membrane protein [Rhodopseudomonas sp. B29]
WP_025033669.1	323	rubrerythrin [Bradyrhizobium sp. DOA9]
WP_027549594.1	323	rubrerythrin [Bradyrhizobium sp. Cp5.3]
WP_008974041.1	323	membrane protein [Bradyrhizobium sp. STM 3843]
WP_011090619.1	323	MULTISPECIES: membrane protein [Bradyrhizobium]
WP_027276389.1	323	rubrerythrin [Rhodopseudomonas palustris]
WP_027543398.1	323	rubrerythrin [Bradyrhizobium sp. WSM2254]
WP_027574981.1	323	rubrerythrin [Bradyrhizobium sp. WSM1743]
WP_011504284.1	323	membrane protein [Rhodopseudomonas palustris]
WP_045005549.1	323	rubrerythrin [Bradyrhizobium sp. LTSP857]
WP_045010919.1	323	rubrerythrin [Bradyrhizobium sp. LTSP849]
WP_028339122.1	323	rubrerythrin [Bradyrhizobium elkanii]
WP_038384833.1	323	rubrerythrin [Bradyrhizobium elkanii]
WP_007599587.1	323	membrane protein [Bradyrhizobium sp. WSM1253]
WP_027570114.1	323	rubrerythrin [Bradyrhizobium sp. URHA0013]
WP_029586581.1	323	rubrerythrin [Bradyrhizobium sp. URHD0069]
WP_007605213.1	323	membrane protein [Bradyrhizobium sp. WSM471]
WP_018457571.1	323	membrane protein [Bradyrhizobium sp. WSM4349]
WP_027515352.1	323	rubrerythrin [Bradyrhizobium sp. WSM1417]
WP_041798024.1	323	rubrerythrin [Rhodopseudomonas palustris]
WP_041801009.1	323	rubrerythrin [Rhodopseudomonas palustris]
WP_018321384.1	323	membrane protein [Bradyrhizobium sp. WSM2793]
WP_028140637.1	323	MULTISPECIES: rubrerythrin [Bradyrhizobium]
WP_027530715.1	323	rubrerythrin [Bradyrhizobium sp. WSM3983]
EHL05695.1	319	rubredoxin [Desulfitobacterium hafniense DP7]
EFG76409.1	314	Rubrerythrin [Mycobacterium parascrofulaceum ATCC BAA-614]
AKG46799.1	312	rubrerythrin [Streptomyces xiamenensis]
CCY71294.1	309	rubredoxin [Clostridium sp. CAG:921]
EIA23552.1	303	"Rubrerythrin, partial [Candidatus Arthromitus sp. SFB-3]"
KPK11130.1	303	hypothetical protein AMJ56_06655 [Anaerolineae bacterium SG8_19]
ETK32968.1	303	hypothetical protein MPTA5024_26900 [Microbispora sp.ATCC PTA-5024]
WP_026561174.1	303	hypothetical protein [Bacillus sp. J37]
ABC76073.1	291	rubrerythrin and thioesterase superfamily protein[Syntrophus aciditrophicus SB]
KPK65072.1	289	hypothetical protein AMK73_03970 [Planctomycetes bacterium SM23_32]
WP_030511192.1	275	hypothetical protein [Microbispora rosea]
WP_030918203.1	272	membrane protein [Streptosporangium amethystogenes]
KLO41234.1	267	hypothetical protein ABW17_14945 [Mycobacterium nebraskense]
CQD10735.1	267	Nigerythrin [Mycobacterium europaeum]
CAE22340.1	266	Rubredoxin:Rubrerythrin:Rubredoxin-type Fe(Cys)4 protein[Prochlorococcus marinus str. MIT 9313]
WP_055586632.1	266	hypothetical protein [Streptomyces

griseoplanus]

WP_037933470.1	266	membrane protein [Streptomyces toyocaensis]
WP_032914808.1	266	MULTISPECIES: membrane protein [Streptomyces]
WP_043909348.1	266	membrane protein [Kitasatospora griseola]
WP_028424074.1	266	membrane protein [Streptomyces sp. GXT6]
WP_053801956.1	266	hypothetical protein [Streptomyces rimosus]
WP_037708137.1	266	membrane protein [Streptomyces sp. AA1529]
WP_051102123.1	266	hypothetical protein [Streptomyces sp. BoleA5]
WP_058083617.1	266	rubrerythrin family protein [Streptomyces hygroscopicus]
WP_030636543.1	266	membrane protein [Streptomyces rimosus]
WP_037786638.1	266	membrane protein [Streptomyces sp. CNT318]
WP_055557719.1	266	hypothetical protein [Streptomyces sp. NBRC 110028]
WP_055627176.1	266	hypothetical protein [Streptomyces cyanoalbus]
WP_030285917.1	266	membrane protein [Streptomyces sp. NRRL B-5680]
WP_030556304.1	266	membrane protein [Streptomyces aureofaciens]
WP_043178704.1	266	MULTISPECIES: membrane protein [Streptomyces]
WP_033349765.1	266	membrane protein [Streptomyces aureofaciens]
WP_039653703.1	266	MULTISPECIES: membrane protein [Streptomyces]
WP_053634931.1	266	hypothetical protein [Streptomyces sp. XY152]
WP_030827252.1	266	MULTISPECIES: membrane protein [Streptomyces]
WP_014677291.1	266	rubrerythrin [Streptomyces hygroscopicus]
WP_037813870.1	266	membrane protein [Streptomyces sp. MspMP-M5]
WP_037753187.1	265	membrane protein [Streptomyces sp. CNQ-525]
WP_040908576.1	265	membrane protein [Streptomyces griseoflavus]
WP_040912249.1	265	membrane protein [Streptomyces vitaminophilus]
WP_037953041.1	265	membrane protein [Streptomyces sp. PRh5]
WP_015759834.1	263	MULTISPECIES: rubrerythrin [Eggerthella]
WP_009305720.1	263	rubrerythrin [Eggerthella sp. 1_3_56FAA]
WP_037969685.1	263	membrane protein [Streptosporangium amethystogenes]
WP_033409825.1	263	membrane protein [Nonomuraea coxensis]
AKT90500.1	261	rubrerythrin [Campylobacter ureolyticus RIGS 9880]
WP_009849329.1	261	rubrerythrin [Mariprofundus ferrooxydans]
WP_026195361.1	261	rubrerythrin [Mariprofundus ferrooxydans]
EET59585.1	256	Rubrerythrin [Marvinbryantia formatexigens DSM 14469]
WP_013978659.1	255	rubrerythrin [Eggerthella sp. YY7918]
EGC88409.1	254	rubredoxin [Eggerthella sp. HGA1]
CBL03851.1	254	Rubrerythrin [Gordonibacter pamelaeae 7-10-1-b]
AAT41909.1	251	putative rubrerythrin [Fremyella diplosiphon Fd33]
CUQ68960.1	247	NADH peroxidase [[Ruminococcus] torques]
CUQ69941.1	247	NADH peroxidase [[Ruminococcus] torques]
ETI88971.1	247	Rubrerythrin [Clostridium butyricum DORA_1]
CBN55596.1	245	rubrerythrin [[[Oscillatoria] sp. PCC 6506]

ERI68923.1   245   Rubrerythrin [Clostridium sp. KLE 1755]
EFD01225.1   245   Rubrerythrin [[Clostridium] hathewayi DSM 13479][Hungatella hathewayi DSM 13479]
CAK28032.1   244   Rubrerythrin [Synechococcus sp. RCC307]
ABB27142.1   243   rubrerythrin [Synechococcus sp. CC9902]
AFY76689.1   243   rubrerythrin [Pleurocapsa sp. PCC 7327]
WP_025782374.1 242   rubrerythrin [Candidatus Synechococcus spongiarum]
KKZ10095.1   242   rubrerythrin [Candidatus Synechococcus spongiarum 15L]
KKZ12851.1   242   rubrerythrin [Candidatus Synechococcus spongiarum SP3]
KGG31229.1   241   Rubrerythrin [Prochlorococcus sp. MIT 0703]
WP_028089445.1 240   rubrerythrin [Dolichospermum circinale]
WP_028082097.1 240   rubrerythrin [Dolichospermum circinale]
EDP22910.1   240   rubredoxin [Faecalibacterium prausnitzii M21/2]
WP_015117309.1 240   rubrerythrin [Rivularia sp. PCC 7116]
WP_027841503.1 240   rubrerythrin [Mastigocoleus testarum]
WP_051931171.1 240   hypothetical protein [Gillisia sp. Hel_I_29]
EGQ12609.1   239   rubrerythrin [Prevotella pallens ATCC 700821]
WP_003517169.1 239   rubrerythrin [Ruminiclostridium thermocellum]
WP_011244747.1 238   MULTISPECIES: rubrerythrin [Synechococcus]
WP_010995347.1 237   rubrerythrin [Nostoc sp. PCC 7120]
WP_013190747.1 237   rubrerythrin [Trichormus azollae]
WP_015078056.1 237   rubrerythrin [Anabaena sp. 90]
WP_015141556.1 237   rubrerythrin [Nostoc sp. PCC 7524]
ABA24022.1   237   Rubrerythrin [Anabaena variabilis ATCC 29413]
BAT52915.1   237   Rubrerythrin [Nostoc sp. NIES-3756]
WP_015115901.1 237   rubredoxin-type Fe(Cys)4 protein [Nostoc sp. PCC 7107]
WP_012412771.1 237   rubrerythrin [Nostoc punctiforme]
ALF55025.1   237   rubrerythrin [Nostoc piscinale CENA21]
WP_039202628.1 237   rubrerythrin [Aphanizomenon flos-aquae]
WP_053540696.1 237   rubrerythrin [Anabaena sp. wa102]
WP_016949313.1 237   rubrerythrin [Anabaena sp. PCC 7108]
WP_015216721.1 237   Rubredoxin-type Fe(Cys)4 protein [Anabaena cylindrica]
WP_027404836.1 237   rubrerythrin [Aphanizomenon flos-aquae]
WP_034834755.1 237   rubrerythrin [Clostridium perfringens]
WP_045867661.1 237   rubrerythrin [Tolypothrix sp. PCC 7601]
WP_006276110.1 237   rubrerythrin [Cylindrospermopsis raciborskii]
WP_057176961.1 237   rubrerythrin [Cylindrospermopsis sp. CR12]
WP_015127233.1 237   rubredoxin-type Fe(Cys)4 protein [Calothrix sp. PCC 7507]
WP_015210778.1 237   rubrerythrin [Cylindrospermum stagnale]
WP_029636653.1 237   rubrerythrin [[Scytonema hofmanni] UTEX B 1581]
WP_039743686.1 237   rubrerythrin [Hassallia byssoidea]
WP_049692655.1 237   rubrerythrin [Synechococcus sp. WH 8103]

WP_011129222.1	237	rubrerythrin [Synechococcus sp. WH 8102]
WP_017323258.1	237	rubrerythrin [cyanobacterium PCC 7702]
WP_026722006.1	237	rubrerythrin [Fischerella sp. PCC 9431]
WP_015200760.1	237	rubredoxin-type Fe(Cys)4 protein [Calothrix parietina]
WP_016861335.1	237	rubrerythrin [Fischerella muscicola]
WP_038552364.1	237	rubrerythrin [Synechococcus sp. KORDI-52]
WP_044450913.1	237	rubrerythrin [Mastigocladus laminosus]
WP_017311057.1	237	rubrerythrin [Fischerella sp. PCC 9339]
WP_026103677.1	237	MULTISPECIES: rubrerythrin [Kamptonema]
WP_026734515.1	237	rubrerythrin [Fischerella sp. PCC 9605]
WP_038083884.1	237	rubrerythrin [Tolypothrix bouteillei]
WP_010309517.1	237	rubrerythrin [Synechococcus sp. CB0101]
WP_019491349.1	237	hypothetical protein [Calothrix sp. PCC 7103]
WP_053456909.1	237	rubrerythrin [Hapalosiphon sp. MRB220]
WP_016876585.1	237	rubrerythrin [Chlorogloeopsis fritschii]
WP_012594839.1	237	rubrerythrin [Cyanothece sp. PCC 8801]
WP_006854148.1	237	rubrerythrin [Synechococcus sp. WH 8016]
WP_016865209.1	237	rubrerythrin [Fischerella muscicola]
WP_011620670.1	237	rubrerythrin [Synechococcus sp. CC9311]
WP_007099265.1	237	rubrerythrin [Synechococcus sp. RS9916]
WP_026787922.1	237	MULTISPECIES: rubrerythrin [Planktothrix]
WP_035157143.1	237	rubrerythrin [Calothrix sp. 336/3]
WP_048347479.1	237	rubrerythrin [Synechococcus sp. WH 8020]
WP_007101258.1	237	rubrerythrin [Synechococcus sp. RS9917]
WP_042154811.1	237	rubrerythrin [Planktothrix agardhii]
WP_043693553.1	237	rubrerythrin [Synechococcus sp. KORDI-49]
WP_048869271.1	237	rubrerythrin [Scytonema tolypothrichoides]
WP_017747159.1	237	rubrerythrin [Scytonema hofmanni]
WP_041040163.1	237	rubrerythrin [Tolypothrix campylonemoides]
WP_015783704.1	237	rubrerythrin [Cyanothece sp. PCC 8802]
WP_026796619.1	237	rubrerythrin [Planktothrix prolifica]
WP_011365456.1	237	rubrerythrin [Synechococcus sp. CC9605]
WP_041425218.1	237	rubrerythrin [Synechococcus sp. CC9902]
WP_017315658.1	237	rubrerythrin [Mastigocladopsis repens]
WP_006172558.1	237	rubrerythrin [Synechococcus sp. WH 5701]
WP_041392872.1	237	rubrerythrin [Pleurocapsa minor]
WP_026098809.1	237	rubrerythrin [Oscillatoria sp. PCC 10802]
WP_009788687.1	237	rubrerythrin [Synechococcus sp. BL107]
WP_013323477.1	237	rubrerythrin [Cyanothece sp. PCC 7822]
WP_017651465.1	236	rubrerythrin [Microchaete sp. PCC 7126]
BAQ59665.1	236	rubredoxin [Geminocystis sp. NIES-3708]
WP_043736828.1	236	rubrerythrin [Synechococcus sp. RCC307]
WP_051621223.1	236	hypothetical protein [Leeuwenhoekiella sp. Hel_I_48]
WP_051605384.1	236	hypothetical protein [Sediminibacter sp. Hel_I_10]
WP_017720143.1	236	hypothetical protein [Oscillatoria sp. PCC 10802]
ABM79614.1	235	Rubredoxin:Rubrerythrin:Rubredoxin-type

Fe(Cys)4 protein[Prochlorococcus marinus str. MIT 9303]
WP_036914792.1 235   MULTISPECIES: rubrerythrin [Prochlorococcus]
WP_041385290.1 235   rubrerythrin [Prochlorococcus marinus]
GAP40024.1   234   rubrerythrin [Anaerolineaceae bacterium TC1]
EAT04986.1   234   Rubrerythrin:Rubredoxin-type Fe(Cys)4 protein
[delta proteobacterium MLMS-1]
WP_010423737.1 234   rubrerythrin [Anaerophaga thermohalophila]
ACV62525.1   233   Rubrerythrin [Desulfotomaculum acetoxidans DSM
771]
ERI81721.1   233   rubrerythrin [Bacteroides pyogenes F0041]
WP_009784521.1 233   rubrerythrin [Lyngbya sp. PCC 8106]
WP_046282288.1 233   rubrerythrin [Limnoraphis robusta]
WP_015132793.1 233   rubredoxin-type Fe(Cys)4 protein [Leptolyngbya
sp.PCC 7376]
WP_023065408.1 233   rubredoxin family protein [Lyngbya aestuarii]
WP_017328276.1 232   rubrerythrin [Synechococcus sp. PCC 7336]
WP_046279991.1 232   rubrerythrin [Limnoraphis robusta]
WP_009787579.1 232   rubrerythrin [Lyngbya sp. PCC 8106]
CCX93121.1   232   putative uncharacterized protein [Firmicutes
bacterium CAG:110]
WP_010317788.1 231   rubrerythrin [Synechococcus sp. CB0205]
WP_006099524.1 231   rubrerythrin [Coleofasciculus chthonoplastes]
WP_013324595.1 230   rubrerythrin [Cyanothece sp. PCC 7822]
WP_015956576.1 228   rubrerythrin [Cyanothece sp. PCC 7424]
ABC78317.1   227   rubrerythrin [Syntrophus aciditrophicus SB]
ERI80233.1   226   "rubredoxin, partial [, partial [[Clostridium]
symbiosum ATCC 14940]"
WP_031580558.1 226   rubrerythrin [Lachnospiraceae bacterium
AC2028]
WP_015142224.1 226   rubrerythrin [Pleurocapsa minor]
CUO67413.1   225   NADH peroxidase [Coprococcus eutactus]
EDS19954.1   225   rubredoxin [Erysipelatoclostridium ramosum DSM
1402]
CDE34494.1   223   rubrerythrin [Prevotella stercorea CAG:629]
CDD70945.1   223   putative uncharacterized protein [Sutterella
sp. CAG:397]
EDK21662.1   223   non-haem iron protein [Campylobacter jejuni
subsp. jejuni CG8486]
WP_051868405.1 223   hypothetical protein [Vibrio sp. ER1A]
WP_006070235.1 223   non-haem iron protein [Vibrio shilonii]
AHF23960.1   222   rubrerythrin [uncultured bacterium Contig19]
EKN30018.1   222   rubrerythrin [Parabacteroides distasonis
CL09T03C24]
KMW41811.1   222   rubrerythrin [Parabacteroides sp. D26]
CUN30362.1   222   NADH peroxidase [Parabacteroides distasonis]
KFA95419.1   222   hypothetical protein HW45_26730 [Vibrio sp.
ER1A]
CDD59157.1   221   rubredoxin [Clostridium sp. CAG:43]
WP_012798333.1 221   rubrerythrin [Slackia heliotrinireducens]
ERJ13641.1   221   L-aspartate oxidase protein [Haloplasma

contractile SSD-17B]
WP_011459535.1 220 rubrerythrin [Desulfitobacterium hafniense]
WP_041709234.1 220 rubrerythrin [[Clostridium] saccharolyticum]
WP_026184035.1 220 rubrerythrin [Desulfitobacterium hafniense]
WP_026198800.1 220 rubrerythrin [Desulfitobacterium hafniense]
WP_035214361.1 220 rubrerythrin [Desulfitobacterium hafniense]
WP_035130631.1 220 rubrerythrin [Clostridium sulfidigenes]
WP_031516882.1 220 rubrerythrin [Desulfotomaculum alkaliphilum]
WP_015359392.1 220 MULTISPECIES: rubrerythrin [Ruminiclostridium]
WP_031583610.1 220 rubrerythrin [Lachnospiraceae bacterium P6A3]
WP_031390765.1 219 rubrerythrin [Clostridium sp. KNHs209]
WP_008340642.1 219 rubrerythrin [Sulfurimonas gotlandica]
WP_021286986.1 219 non-heme iron protein [Sulfurimonas sp.
AST-10]
KIM02820.1 219 enolase [Sulfurovum sp. AS07-7]
WP_013553152.1 219 rubrerythrin [Nitratifractor salsuginis]
WP_024954170.1 219 rubrerythrin [Sulfurospirillum arcachonense]
WP_011980131.1 219 rubrerythrin [Sulfurovum sp. NBC37-1]
WP_046551573.1 219 rubrerythrin [Sulfurovum lithotrophicum]
WP_014473823.1 219 rubrerythrin [Arcobacter sp. L]
WP_046550517.1 218 rubrerythrin [Sulfurovum lithotrophicum]
WP_012083640.1 218 rubrerythrin [Sulfurovum sp. NBC37-1]
WP_013327203.1 218 rubrerythrin [Sulfurimonas autotrophica]
WP_051283750.1 218 hypothetical protein [Desulforegula
conservatrix]
WP_025071778.1 217 rubrerythrin [Prevotella timonensis]
WP_004376597.1 217 rubrerythrin [Prevotella oris]
WP_004372594.1 217 rubrerythrin [Prevotella oris]
WP_008566079.1 217 rubrerythrin [Prevotella maculosa]
WP_004348733.1 217 rubrerythrin [Prevotella buccalis]
WP_036873262.1 217 rubrerythrin [Prevotella buccalis]
WP_004351576.1 217 rubrerythrin [Prevotella denticola]
WP_013671782.1 217 rubrerythrin [Prevotella denticola]
WP_023058523.1 217 MULTISPECIES: rubrerythrin [Prevotella]
WP_025068209.1 217 rubrerythrin [Prevotella denticola]
WP_008125168.1 217 rubrerythrin [Prevotella timonensis]
WP_025837601.1 217 rubrerythrin [Prevotella scopos]
WP_023983663.1 217 hypothetical protein [Prevotella oralis]
WP_004369792.1 217 rubrerythrin [Prevotella oralis]
WP_021671773.1 217 rubredoxin [Prevotella sp. F0091]
WP_004379319.1 217 rubrerythrin [Prevotella oulorum]
WP_025071093.1 217 rubrerythrin [Prevotella oulorum]
WP_008448005.1 217 rubrerythrin [Prevotella amnii]
WP_007367068.1 217 rubrerythrin [Prevotella multiformis]
WP_004381741.1 217 rubrerythrin [Prevotella veroralis]
WP_025078590.1 217 rubrerythrin [Prevotella fusca]
WP_028898802.1 217 rubrerythrin [Prevotella sp. HJM029]
WP_036895507.1 217 rubrerythrin [Prevotella sp. oral taxon 306]
WP_013265577.1 217 rubrerythrin [Prevotella melaninogenica]
WP_004364910.1 217 hypothetical protein [Prevotella nigrescens]

WP_036865562.1	217	rubrerythrin [Prevotella melaninogenica]
WP_008823798.1	217	rubrerythrin [Prevotella histicola]
WP_004359194.1	217	rubrerythrin [Prevotella melaninogenica]
WP_036896745.1	217	rubrerythrin [Prevotella sp. S7 MS 2]
WP_040595923.1	217	rubrerythrin [Prevotella pallens]
WP_036871467.1	217	rubrerythrin [Prevotella histicola]
WP_004355069.1	217	rubrerythrin [Prevotella disiens]
WP_036922198.1	217	rubrerythrin [Prevotella sp. ICM33]
WP_025876516.1	217	rubrerythrin [Prevotella corporis]
WP_036895325.1	217	rubrerythrin [Prevotella sp. S7-1-8]
WP_005843208.1	217	MULTISPECIES: rubrerythrin [Prevotellaceae]
WP_025792895.1	217	rubrerythrin [Prevotella histicola]
WP_024998603.1	217	rubrerythrin [Prevotella falsenii]
WP_009010238.1	217	rubrerythrin [Prevotella sp. C561]
WP_004338012.1	217	MULTISPECIES: rubrerythrin [Prevotella]
WP_036864207.1	217	rubrerythrin [Prevotella bivia]
WP_028905728.1	217	rubrerythrin [Prevotella intermedia]
WP_013135919.1	217	rubrerythrin [Arcobacter nitrofigilis]
WP_045168271.1	217	rubrerythrin [Prevotella intermedia]
WP_044047427.1	217	rubrerythrin [Prevotella intermedia]
WP_029408521.1	217	rubrerythrin [Thiomicrospira sp. Milos-T2]
WP_028896004.1	217	rubrerythrin [Prevotella sp. HUN102]
WP_007175273.1	217	rubrerythrin [Prevotella bergensis]
EFW03993.1	217	rubrerythrin [Coprobacillus sp. 29_1]
WP_044418237.1	217	rubrerythrin [Arcobacter anaerophilus]
EST48653.1	217	Rubrerythrin 1 [Spironucleus salmonicida]
EBA40034.1	217	rubredoxin [Collinsella aerofaciens ATCC 25986]
WP_021825613.1	216	rubrerythrin [Prevotella salivae]
WP_007133984.1	216	rubrerythrin [Prevotella salivae]
ETJ98907.1	216	rubrerythrin [Eubacterium nodatum ATCC 33099]
CDC41645.1	216	putative uncharacterized protein [Firmicutes bacterium CAG:449]
KHG34230.1	216	enolase [Sulfurospirillum sp. MES]
WP_012857090.1	216	rubrerythrin [Sulfurospirillum deleyianum]
WP_041961684.1	216	rubrerythrin [Sulfurospirillum cavolei]
WP_041957740.1	216	rubrerythrin [Sulfurospirillum arsenophilum]
WP_025345025.1	216	rubrerythrin [Sulfurospirillum multivorans]
KFL33618.1	216	enolase [Sulfurospirillum sp. SCADC]
WP_002917833.1	216	rubrerythrin [Campylobacter jejuni]
WP_057044055.1	216	enolase [Campylobacter jejuni]
WP_014769547.1	216	iron-binding protein [Sulfurospirillum barnesii]
CCJ35014.1	216	rubrerythrin [Methanoculleus bourgensis MS2]
WP_019892954.1	216	hypothetical protein [Allobaculum stercoricanis]
EDR46845.1	216	Rubrerythrin [Dorea formicigenerans ATCC 27755]
WP_002898833.1	215	non-heme iron protein [Campylobacter jejuni]
EFB90103.1	215	Rubrerythrin [Pyramidobacter piscolens W5455]

WP_044598121.1	215	desulforubrerythrin [Campylobacter peloridis]
WP_034286987.1	215	rubrerythrin [Helicobacter sp. MIT 11-5569]
WP_012660833.1	215	desulforubrerythrin [Campylobacter lari]
WP_039662379.1	215	desulforubrerythrin [Campylobacter subantarcticus]
WP_039666391.1	215	desulforubrerythrin [Campylobacter sp. RM16704]
KGL36644.1	215	enolase [Helicobacter sp. MIT 05-5294]
WP_026943897.1	215	rubrerythrin [Helicobacter rodentium]
WP_012108207.1	215	rubrerythrin [Campylobacter hominis]
WP_011139226.1	215	non-heme iron protein [Wolinella succinogenes]
WP_002787434.1	215	non-heme iron protein [Campylobacter coli]
WP_020974054.1	215	rubrerythrin [Campylobacter coli]
WP_038831292.1	215	rubrerythrin [Campylobacter coli]
WP_038839309.1	215	rubrerythrin [Campylobacter coli]
WP_002860858.1	215	non-hem iron protein [Campylobacter jejuni]
WP_038832622.1	215	rubrerythrin [Campylobacter coli]
WP_038836225.1	215	rubrerythrin [Campylobacter coli]
WP_014516877.1	215	rubrerythrin [Campylobacter jejuni]
WP_013459707.1	215	rubrerythrin [Sulfuricurvum kujiense]
WP_002853081.1	215	rubrerythrin [Campylobacter jejuni]
WP_038855424.1	215	rubrerythrin [Campylobacter coli]
WP_002884670.1	215	MULTISPECIES: non-heme iron protein [Campylobacter]
WP_002779391.1	215	MULTISPECIES: rubrerythrin [Campylobacter]
WP_002855562.1	215	MULTISPECIES: rubrerythrin [Campylobacter]
WP_041159967.1	215	rubrerythrin [Campylobacter jejuni]
WP_044600797.1	215	rubrerythrin [Campylobacter coli]
WP_052801959.1	215	rubrerythrin [Campylobacter jejuni]
WP_002867501.1	215	non-heme iron protein [Campylobacter jejuni]
WP_002868315.1	215	non-heme iron protein [Campylobacter jejuni]
WP_032586422.1	215	rubrerythrin [Campylobacter jejuni]
WP_038851732.1	215	rubrerythrin [Campylobacter coli]
WP_042963519.1	215	rubrerythrin [Campylobacter coli]
WP_057037556.1	215	enolase [Campylobacter coli]
WP_013977947.1	215	rubrerythrin [Clostridium sp. SY8519]
WP_002846240.1	215	rubrerythrin [Campylobacter coli]
WP_052794530.1	215	rubrerythrin [Campylobacter jejuni]
WP_057043868.1	215	enolase [Campylobacter coli]
WP_057037298.1	215	enolase [Campylobacter coli]
WP_015654007.1	215	hypothetical protein [uncultured Sulfuricurvum sp.RIFRC-1]
KIM11624.1	215	enolase [Sulfuricurvum sp. PC08-66]
WP_002831541.1	215	non-heme iron protein [Campylobacter coli]
WP_021357971.1	215	non-heme iron protein [Campylobacter jejuni]
WP_002809654.1	215	non-heme iron protein [Campylobacter coli]
WP_006802716.1	215	rubrerythrin [Helicobacter winghamensis]
WP_004276640.1	215	rubrerythrin [Campylobacter upsaliensis]
WP_004276092.1	215	rubrerythrin [Campylobacter upsaliensis]
WP_052797076.1	215	rubrerythrin [Campylobacter jejuni]

WP_057030697.1	215	enolase [Campylobacter coli]
WP_027304384.1	215	rubrerythrin [Campylobacter upsaliensis]
WP_052783378.1	215	rubrerythrin [Campylobacter coli]
WP_057031322.1	215	enolase [Campylobacter coli]
WP_039648902.1	215	desulforubrerythrin [Campylobacter insulaenigrae]
WP_039664942.1	215	desulforubrerythrin [Campylobacter volucris]
WP_025802608.1	215	rubrerythrin [Campylobacter corcagiensis]
WP_033915596.1	214	rubrerythrin [Campylobacter sputorum]
WP_050335670.1	214	rubrerythrin [Campylobacter ureolyticus]
WP_018712896.1	214	non-heme iron protein [Campylobacter ureolyticus]
WP_024962411.1	214	rubrerythrin [Campylobacter ureolyticus]
WP_016646523.1	214	iron-binding protein [Campylobacter ureolyticus]
WP_021091887.1	214	rubrerythrin [Campylobacter concisus]
WP_021092534.1	214	rubrerythrin [Campylobacter concisus]
WP_052791412.1	214	rubrerythrin [Campylobacter jejuni]
WP_058001507.1	214	rubrerythrin family protein [Campylobacter coli]
WP_012140636.1	214	rubrerythrin [Campylobacter concisus]
WP_002940712.1	214	MULTISPECIES: rubrerythrin [Campylobacter]
WP_034970165.1	214	rubrerythrin [Campylobacter mucosalis]
WP_054197314.1	214	rubrerythrin [Campylobacter concisus]
WP_005870535.1	214	rubrerythrin [Campylobacter gracilis]
EKD64303.1	213	rubrerythrin [uncultured bacterium]
EEG76070.1	213	Rubrerythrin [Dethiobacter alkaliphilus AHT 1]
WP_022749680.1	212	rubrerythrin [Lachnobacterium bovis]
EDP25422.1	211	rubredoxin [Coprococcus eutactus ATCC 27759]
EEI86353.1	211	Rubrerythrin [Anaerococcus lactolyticus ATCC 51172]
WP_019001923.1	211	rubrerythrin [Succinimonas amylolytica]
CUN03067.1	211	NADH peroxidase [Turicibacter sanguinis]
EFB31112.1	211	rubredoxin [Prevotella oris F0302]
CDA87843.1	210	putative uncharacterized protein [Clostridium sp.CAG:230]
WP_055657808.1	210	rubrerythrin [Hungatella hathewayi]
EKD31566.1	210	rubrerythrin [uncultured bacterium]
EEC95977.1	210	rubredoxin [Parabacteroides johnsonii DSM 18315]
EEA91206.1	210	rubredoxin [Collinsella stercoris DSM 13279]
EEP43546.1	210	rubredoxin [Collinsella intestinalis DSM 13280]
WP_031492602.1	210	hypothetical protein [Succinivibrio dextrinosolvens]
ABW19864.1	209	Rubrerythrin [Alkaliphilus oremlandii OhILAs]
WP_005609372.1	209	rubrerythrin [Ruminococcus lactaris]
WP_038374926.1	209	rubrerythrin [Brachyspira alvinipulli]
WP_051731102.1	209	hypothetical protein [Mucinivorans hirudinis]
WP_051566185.1	208	hypothetical protein [Acidobacteriaceae

bacterium URHE0068]
WP_055072040.1 208   rubrerythrin [Clostridium sp. ND2]
WP_002571948.1 208   rubrerythrin [[Clostridium] bolteae]
WP_002569884.1 208   rubrerythrin [[Clostridium] bolteae]
WP_002589915.1 208   rubrerythrin [[Clostridium] clostridioforme]
WP_002578225.1 208   rubrerythrin [[Clostridium] bolteae]
CDB30714.1   208   rubrerythrin [Firmicutes bacterium CAG:137]
KLU71190.1   208   hypothetical protein RHS_2969 [Robinsoniella sp. RHS]
WP_051538928.1 208   hypothetical protein [Prolixibacter bellariivorans]
XP_001580748.1 207   Rubrerythrin family protein [Trichomonas vaginalis G3]
WP_057991118.1 207   rubrerythrin family protein [Campylobacter jejuni]
WP_016440945.1 207   hypothetical protein [Coprococcus sp. HPP0048]
WP_008974797.1 207   rubrerythrin [Lachnospiraceae bacterium 6_1_37FAA]
WP_009262492.1 207   rubrerythrin [Lachnospiraceae bacterium 9_1_43BFAA]
WP_016438290.1 207   hypothetical protein [Coprococcus sp. HPP0074]
WP_050606346.1 206   rubrerythrin [Clostridium sp. mt5]
EEK16578.1   206   Rubrerythrin [Porphyromonas uenonis 60-3]
WP_016303516.1 206   hypothetical protein [Lachnospiraceae bacterium A2]
WP_009982723.1 206   rubrerythrin [Ruminococcus flavefaciens]
WP_008395793.1 206   rubrerythrin [Clostridium sp. M62/1]
CDF02813.1   206   rubrerythrin [Ruminococcus sp. CAG:624]
EID33055.1   205   rubrerythrin [Prevotella sp. oral taxon 306 str. F0472]
EGQ11584.1   205   rubrerythrin [Prevotella nigrescens ATCC 33563]
ERJ76704.1   205   rubredoxin [Prevotella disiens JCM 6334 = ATCC 29426]
KJJ86208.1   205   rubrerythrin [Prevotella intermedia ZT]
AFJ07599.1   205   rubrerythrin [Prevotella intermedia 17]
WP_051316903.1 205   rubrerythrin [Haloplasma contractile]
CDD39911.1   205   rubrerythrin [Clostridium sp. CAG:299]
WP_028520318.1 205   rubrerythrin [Ruminococcus flavefaciens]
WP_024859293.1 205   rubrerythrin [Ruminococcus flavefaciens]
WP_028515988.1 205   rubrerythrin [Ruminococcus flavefaciens]
WP_028516484.1 205   rubrerythrin [Ruminococcus flavefaciens]
WP_012527187.1 205   rubrerythrin [Anaeromyxobacter sp. K]
WP_015934432.1 205   rubrerythrin [Anaeromyxobacter dehalogenans]
Q58144.1   204   RecName: Full=Putative rubrerythrin
EGV50649.1   204   rubrerythrin [endosymbiont of Riftia pachyptila (vent Ph05)]
WP_045725046.1 204   rubrerythrin [Clostridium baratii]
WP_054200618.1 204   rubrerythrin [Clostridium baratii]
WP_055208133.1 204   rubrerythrin [Clostridium baratii]

WP_039312367.1 204   rubrerythrin [Clostridium baratii]
GAP73452.1   204   rubrerythrin [Candidatus Symbiothrix
dinenymphae]
WP_024614067.1 204   rubrerythrin [Clostridium sp. Ade.TY]
WP_040215138.1 204   rubrerythrin [Clostridium polynesiense]
KRN50268.1   204   rubrerythrin [Kandleria vitulina DSM 20405]
WP_002586991.1 204   rubrerythrin [[Clostridium] clostridioforme]
WP_027641755.1 204   hypothetical protein [[Clostridium]
clostridioforme]
WP_016322753.1 204   hypothetical protein [Oscillibacter sp. 1-3]
WP_016217411.1 204   hypothetical protein [Dorea sp. 5-2]
CDD59022.1   204   rubrerythrin [Eggerthella sp. CAG:298]
WP_033124300.1 204   rubrerythrin [Eubacterium sp. ER2]
EHL88164.1   204   reverse rubrerythrin-2 [Tannerella sp.
6_1_58FAA_CT1]
CDD89853.1   204   reverse rubrerythrin-2 [Tannerella sp. CAG:51]
WP_037299025.1 204   rubrerythrin [Ruminococcus flavefaciens]
WP_019678584.1 204   hypothetical protein [Ruminococcus
flavefaciens]
WP_047109763.1 204   rubrerythrin [Brachyspira hyodysenteriae]
WP_048593458.1 204   rubrerythrin [Brachyspira suanatina]
WP_014488838.1 204   rubrerythrin [Brachyspira intermedia]
WP_047114597.1 204   rubrerythrin [Brachyspira hyodysenteriae]
WP_044555390.1 204   rubrerythrin [Brachyspira hyodysenteriae]
WP_020064315.1 204   rubrerythrin [Brachyspira hyodysenteriae]
WP_008723643.1 204   rubrerythrin [Brachyspira hampsonii]
WP_047113621.1 204   rubrerythrin [Brachyspira hyodysenteriae]
ELV06636.1   204   rubrerythrin [Brachyspira hampsonii 30599]
WP_011422143.1 204   rubrerythrin [Anaeromyxobacter dehalogenans]
GAO04568.1   204   nigerythrin [Anaeromyxobacter sp. PSR-1]
ABR50261.1   203   Rubrerythrin [Alkaliphilus metalliredigens
QYMF]
WP_038672312.1 203   rubrerythrin [Ruminococcus bicirculans]
CDC67814.1   203   putative rubrerythrin [Ruminococcus sp. CAG:
57]
WP_025544888.1 203   hypothetical protein [Oscillospiraceae
bacterium VE202-24]
WP_016224705.1 203   hypothetical protein [Lachnospiraceae
bacterium 3-2]
WP_021751014.1 203   MULTISPECIES: rubrerythrin [Oscillibacter]
WP_029504115.1 203   hypothetical protein [Lachnoclostridium
phytofermentans]
WP_003503522.1 203   MULTISPECIES: rubrerythrin [Clostridiales]
WP_004605896.1 203   rubrerythrin [[Clostridium] scindens]
WP_048621396.1 203   rubrerythrin [Clostridium sp. BR72]
WP_025642608.1 203   hypothetical protein [[Clostridium] scindens]
WP_024731971.1 203   hypothetical protein [Clostridiales bacterium
VE202-14]
WP_049730133.1 203   rubrerythrin [Dorea sp. D27]
WP_006444442.1 203   rubrerythrin [[Clostridium] hylemonae]

WP_015558524.1 203 rubrerythrin [Ruminococcus champanellensis]
WP_049673858.1 203 rubrerythrin [Desulfocarbo indianensis]
WP_013256906.1 203 rubrerythrin [Desulfarculus baarsii]
WP_042432513.1 203 nigerythrin [Senegalimassilia anaerobia]
WP_054043891.1 203 hypothetical protein [bacterium 336/3]
WP_041747605.1 203 rubrerythrin [Brachyspira pilosicoli]
WP_015274941.1 203 rubrerythrin [Brachyspira pilosicoli]
CDD52635.1 203 rubrerythrin [Ruminococcus sp. CAG:379]
EFR40504.1 202 Rubrerythrin [Selenomonas sp. oral taxon 137 str. F0430]
BAC57530.1 202 Rrc [Campylobacter jejuni]
WP_052543956.1 202 rubrerythrin [Desulfotomaculum gibsoniae]
WP_044945822.1 202 rubrerythrin [Blautia schinkii]
CDE06272.1 202 rubrerythrin [Prevotella sp. CAG:485]
WP_016291146.1 202 hypothetical protein [Lachnospiraceae bacterium 28-4]
WP_055160665.1 202 rubrerythrin [[Ruminococcus] torques]
WP_055172634.1 202 rubrerythrin [[Ruminococcus] torques]
CBL25408.1 202 Rubrerythrin [Ruminococcus torques L2-14]
WP_054753190.1 202 rubrerythrin [Ruminococcus faecis]
WP_020436936.1 202 rubrerythrin [[Ruminococcus] torques]
WP_055146791.1 202 rubrerythrin [[Ruminococcus] torques]
WP_040015518.1 202 rubrerythrin [Dorea formicigenerans]
EXM38959.1 202 hypothetical protein RASY3_11645 [Ruminococcus albus SY3]
WP_005340028.1 202 MULTISPECIES: rubrerythrin [Dorea]
WP_016213283.1 202 hypothetical protein [Eubacterium sp. 14-2]
WP_022740807.1 202 nigerythrin [Adlercreutzia equolifaciens]
WP_035067608.1 202 nigerythrin [Desulfovibrio termitidis]
WP_035129934.1 202 rubrerythrin [Clostridium sulfidigenes]
1YUX 202 "Chain A, Mixed Valant State Of Nigerythrin"
WP_010937330.1 202 nigerythrin [Desulfovibrio vulgaris]
WP_016309632.1 202 MULTISPECIES: hypothetical protein [Enterorhabdus]
AAC45480.1 202 nigerythrin [Desulfovibrio vulgaris str. Hildenborough]
WP_012613203.1 202 rubrerythrin [Desulfovibrio vulgaris]
WP_011369063.1 202 rubrerythrin [Desulfovibrio alaskensis]
WP_007525431.1 202 rubrerythrin family protein [Desulfovibrio sp. A2]
WP_039690873.1 202 nigerythrin [Coriobacteriaceae bacterium 68-1-3]
WP_048594774.1 202 rubrerythrin [Brachyspira suanatina]
WP_013115023.1 202 rubrerythrin [Brachyspira murdochii]
WP_014487499.1 202 rubrerythrin [Brachyspira intermedia]
WP_008724750.1 202 rubrerythrin [Brachyspira hampsonii]
WP_012670342.1 202 rubrerythrin [Brachyspira hyodysenteriae]
ELV04650.1 202 rubrerythrin [Brachyspira hampsonii 30599]
WP_015539117.1 202 rubrerythrin [Gordonibacter pamelaeae]
WP_047113502.1 202 rubrerythrin [Brachyspira hyodysenteriae]

KPJ58394.1	201	Rubrerythrin-2 [Planctomycetes bacterium DG_23]
CDB14991.1	201	rubrerythrin [Clostridium sp. CAG:221]
WP_006783684.1	201	MULTISPECIES: rubrerythrin [Turicibacter]
WP_024853333.1	201	hypothetical protein [[Ruminococcus] gnavus]
CCZ67318.1	201	putative uncharacterized protein [Ruminococcus gnavus CAG:126]
WP_009245414.1	201	rubrerythrin [[Ruminococcus] gnavus]
WP_004843897.1	201	rubrerythrin [[Ruminococcus] gnavus]
WP_004614768.1	201	rubrerythrin [Tyzzerella nexilis]
WP_053985749.1	201	rubrerythrin [Lachnospiraceae bacterium mt14]
WP_050641724.1	201	MULTISPECIES: rubrerythrin [Clostridiales]
WP_025654824.1	201	hypothetical protein [Clostridiales bacterium VE202-21]
WP_009001958.1	201	MULTISPECIES: rubrerythrin [Clostridiales]
WP_048920693.1	201	hypothetical protein [Rufibacter sp. DG31D]
WP_047111403.1	201	rubrerythrin [Brachyspira hyodysenteriae]
WP_047101416.1	201	rubrerythrin [Brachyspira hyodysenteriae]
WP_047117049.1	201	rubrerythrin [Brachyspira hyodysenteriae]
WP_028330639.1	201	rubrerythrin [Brachyspira alvinipulli]
XP_004254521.1	201	hypothetical protein EIN_411030 [Entamoeba invadens IP1]
ERI92238.1	200	rubrerythrin [Clostridiales bacterium oral taxon 876 str.F0540]
WP_003543373.1	200	MULTISPECIES: rubrerythrin [Desulfotomaculum]
ETP71079.1	200	"rubrerythrin, partial [Lachnospiraceae bacterium JC7]"
Q9AGG3.1	200	RecName: Full=Rubrerythrin; Short=Rr
WP_006599645.1	200	rubrerythrin [Pseudoramibacter alactolyticus]
WP_055277651.1	200	rubrerythrin [Turicibacter sanguinis]
WP_055242455.1	200	rubrerythrin [Turicibacter sanguinis]
WP_013360273.1	200	rubrerythrin [[Clostridium] sticklandii]
CQB88637.1	200	Rubrerythrin [Chlamydia trachomatis]
WP_051454291.1	200	rubrerythrin [Ruminococcus albus]
WP_051506512.1	200	rubrerythrin [Ruminococcus albus]
WP_031390589.1	200	hypothetical protein [Clostridium sp. KNHs209]
WP_018027689.1	200	hypothetical protein [Porphyromonas somerae]
WP_002851039.1	200	rubrerythrin [Ruminococcus albus]
WP_013496944.1	200	rubrerythrin [Ruminococcus albus]
WP_009268121.1	200	rubrerythrin [Lachnospiraceae bacterium 1_4_56FAA]
WP_016296345.1	200	hypothetical protein [Lachnospiraceae bacterium M18-1]
WP_009608073.1	200	rubredoxin [Eggerthella sp. HGA1]
CDB01269.1	200	putative uncharacterized protein [Lachnospiraceae bacterium CAG:215]
WP_050623094.1	200	rubrerythrin [Clostridium sp. GD3]
EPZ59452.1	200	rubrerythrin family protein [[[Clostridium] sordellii ATCC 9714]
WP_009306007.1	200	MULTISPECIES: rubrerythrin [Eggerthella]

WP_039249747.1	200	rubrerythrin [Clostridium novyi]
WP_011721332.1	200	MULTISPECIES: rubrerythrin [Clostridium]
WP_004073587.1	200	hypothetical protein [Clostridium sp. ASF502]
WP_013980654.1	200	rubrerythrin [Eggerthella sp. YY7918]
WP_039252283.1	200	rubrerythrin [Clostridium novyi]
WP_003364011.1	200	rubrerythrin [Clostridium botulinum]
ADC70114.1	199	Rubrerythrin [Methanocaldococcus sp. FS406-22]
WP_025434443.1	199	rubrerythrin [Eubacterium acidaminophilum]
WP_038266725.1	199	rubrerythrin [[Clostridium] litorale]
WP_013008273.1	199	rubrerythrin [Deferribacter desulfuricans]
WP_038352032.1	199	rubrerythrin [Eubacterium limosum]
WP_013382441.1	199	rubrerythrin [Eubacterium limosum]
WP_018359254.1	199	hypothetical protein [Porphyromonas levii]
WP_008721830.1	199	rubrerythrin [Clostridiales bacterium 1_7_47FAA]
WP_007869001.1	199	rubrerythrin [[Clostridium] citroniae]
WP_045091470.1	199	rubrerythrin [Clostridium sp. FS41]
WP_007709123.1	199	rubrerythrin [[Clostridium] asparagiforme]
WP_054333910.1	199	rubrerythrin [Clostridia bacterium UC5.1-2E3]
WP_054348724.1	199	rubrerythrin [Clostridia bacterium UC5.1-1D2]
WP_027293056.1	199	MULTISPECIES: hypothetical protein [Robinsoniella]
WP_016288045.1	199	hypothetical protein [Lachnospiraceae bacterium 3-1]
WP_028505770.1	199	hypothetical protein [Ruminococcus sp. FC2018]
EKQ54176.1	198	rubrerythrin [Methanobacterium sp. Maddingley MBC34]
AEH60041.1	198	Rubrerythrin [Methanosalsum zhilinae DSM 4017]
CDD62483.1	198	rubrerythrin [Clostridium sp. CAG:505]
CDC30243.1	198	rubrerythrin [Firmicutes bacterium CAG:466]
CRZ33564.1	198	Rubrerythrin-1 [Herbinix hemicellulosilytica]
WP_015360285.1	198	rubrerythrin-1 [[Clostridium] stercorarium]
WP_034836484.1	198	rubrerythrin [[Clostridium] cellulosi]
WP_013886953.1	198	rubrerythrin [Flexistipes sinusarabici]
EFY07444.1	198	rubredoxin [Succinatimonas hippei YIT 12066]
WP_050741716.1	198	rubrerythrin [Acetobacterium bakii]
WP_014356061.1	198	rubrerythrin [Acetobacterium woodii]
WP_026394585.1	198	rubrerythrin [Acetobacterium dehalogenans]
CCX91110.1	198	rubrerythrin [Firmicutes bacterium CAG:110]
WP_009252885.1	198	MULTISPECIES: rubrerythrin [Clostridiales]
WP_033144017.1	198	rubrerythrin [Blautia producta]
WP_054353691.1	198	rubrerythrin [Clostridia bacterium UC5.1-1D4]
WP_042271465.1	198	rubrerythrin [Clostridium dakarense]
WP_018593700.1	198	hypothetical protein [Blautia producta]
WP_025490235.1	198	hypothetical protein [Clostridium sp. KLE 1755]
WP_027398117.1	198	hypothetical protein [Anaerovorax odorimutans]
WP_041139637.1	198	rubrerythrin [Clostridiaceae bacterium GM1]
WP_050638332.1	198	rubrerythrin [Candidatus Stoquefichus sp. SB1]
WP_042401121.1	198	MULTISPECIES: rubrerythrin [Clostridium]

WP_028528106.1 198   hypothetical protein [Ruminococcus gauvreauii]
CDA73742.1   198   putative rubrerythrin [Ruminococcus sp. CAG:
579]
WP_008788339.1 198   MULTISPECIES: rubrerythrin [Coprobacillus]
WP_032117422.1 198   rubrerythrin [Clostridium sp. CL-2]
WP_016226753.1 198   hypothetical protein [Lachnospiraceae
bacterium 10-1]
WP_028043282.1 198   hypothetical protein [Candidatus Stoquefichus
massiliensis]
WP_055277254.1 198   rubrerythrin [Clostridium disporicum]
WP_057570376.1 198   rubrerythrin [[Clostridium] sordellii]
CCX40948.1   198   putative uncharacterized protein [Firmicutes
bacterium CAG:102]
WP_033117454.1 198   rubrerythrin [Intestinimonas
butyriciproducens]
CDD09509.1   198   rubrerythrin [Clostridium sp. CAG:349]
CCY04538.1   198   rubrerythrin [Faecalibacterium sp. CAG:1138]
AAB85259.1   197   rubrerythrin [Methanothermobacter
thermautotrophicus str.Delta H]
WP_014256395.1 197   rubrerythrin [[Clostridium] clariflavum]
WP_026883794.1 197   rubrerythrin [Clostridium akagii]
WP_010244176.1 197   rubrerythrin [Acetivibrio cellulolyticus]
WP_014903702.1 197   rubrerythrin [Desulfosporosinus meridiei]
EEP28846.1   197   Rubrerythrin [Shuttleworthia satelles DSM
14600]
WP_054325313.1 197   rubrerythrin [Coprobacillus cateniformis]
WP_034602489.1 197   rubrerythrin [Desulfosporosinus sp. HMP52]
WP_036935694.1 197   rubrerythrin [Pseudobacteroides
cellulosolvens]
WP_041271342.1 197   rubrerythrin [Desulfovibrio aespoeensis]
KJS47068.1   197   rubrerythrin [Peptococcaceae bacterium
BRH_c23]
WP_054873765.1 197   rubrerythrin [Oxobacter pfennigii]
WP_015049881.1 197   rubrerythrin [Thermacetogenium phaeum]
WP_004463395.1 197   rubrerythrin [Ruminiclostridium thermocellum]
WP_007288125.1 197   rubrerythrin [Thermosinus carboxydivorans]
WP_054260686.1 197   rubrerythrin [Propionispora sp. Iso2/2]
WP_013379042.1 197   rubrerythrin [Eubacterium limosum]
WP_044565138.1 197   rubrerythrin [Anaerococcus provenciensis]
CUH93398.1   197   Rubrerythrin [Herbinix sp. SD1D]
WP_037326024.1 197   rubrerythrin [Anaerococcus lactolyticus]
WP_027634524.1 197   rubrerythrin [Clostridium hydrogeniformans]
WP_008901603.1 197   MULTISPECIES: rubrerythrin [Peptoniphilus]
WP_045575486.1 197   rubrerythrin [Desulfosporosinus sp. I2]
WP_047389476.1 197   rubrerythrin [Firmicutes bacterium ZOR0006]
WP_013010221.1 197   rubrerythrin [Denitrovibrio acetiphilus]
WP_027390348.1 197   rubrerythrin [Chrysiogenes arsenatis]
WP_005957221.1 197   rubrerythrin [Peptoniphilus harei]
WP_046496496.1 197   rubrerythrin [Syntrophomonas zehnderi]
WP_054252877.1 197   rubrerythrin [Clostridiales bacterium SIT13]

WP_004813891.1 197   rubrerythrin [Anaerococcus hydrogenalis]
WP_034427919.1 197   MULTISPECIES: rubrerythrin [Firmicutes]
WP_004817609.1 197   rubrerythrin [Anaerococcus hydrogenalis]
WP_010293906.1 197   rubrerythrin [Clostridium senegalense]
WP_023055254.1 197   rubrerythrin [Peptoniphilus sp. BV3C26]
WP_013505708.1 197   rubrerythrin [Desulfurispirillum indicum]
WP_040398215.1 197   rubrerythrin [Anaerococcus senegalensis]
WP_012993274.1 197   rubrerythrin [Mageeibacillus indolicus]
WP_004839517.1 197   rubrerythrin [Anaerococcus vaginalis]
WP_019116854.1 197   rubrerythrin [Anaerococcus sp. PH9]
WP_049690310.1 197   rubrerythrin [Anaerococcus sp. SB3]
WP_019118774.1 197   rubrerythrin [Anaerococcus obesiensis]
WP_018589524.1 197   hypothetical protein [Terrisporobacter glycolicus]
WP_040660142.1 197   rubrerythrin [Oscillibacter ruminantium]
CUH92540.1   197   hypothetical protein SD1D_0993 [Herbinix sp. SD1D]
CCZ33753.1   197   putative uncharacterized protein [Firmicutes bacterium CAG:646]
WP_021844373.1 197   hypothetical protein [Blautia hydrogenotrophica]
WP_026900308.1 197   hypothetical protein [Peptostreptococcaceae bacterium VA2]
WP_054631293.1 197   rubrerythrin [[Clostridium] sordellii]
CEP42082.1   197   rubrerythrin [[[Clostridium] sordellii]
WP_050617663.1 197   rubrerythrin [Intestinimonas sp. GD2]
WP_055267809.1 197   rubrerythrin [Clostridium disporicum]
WP_005947299.1 197   rubrerythrin [Blautia hydrogenotrophica]
CEP99809.1   197   rubrerythrin [[[Clostridium] sordellii]
WP_057579303.1 197   rubrerythrin [[Clostridium] sordellii]
CEP90437.1   197   rubrerythrin [[Clostridium] sordellii]
WP_021128688.1 197   rubrerythrin-1 [[Clostridium] sordellii]
CEP44329.1   197   rubrerythrin [[[Clostridium] sordellii]
WP_025234284.1 197   hypothetical protein [Clostridium sp. ASBs410]
WP_007491149.1 197   MULTISPECIES: rubrerythrin [Clostridiales]
WP_054790870.1 197   rubrerythrin [[Clostridium] sphenoides]
WP_044941228.1 197   rubrerythrin [Flavonifractor plautii]
WP_039890473.1 197   rubrerythrin [Hungatella hathewayi]
WP_038280027.1 197   rubrerythrin [[Clostridium] celerecrescens]
WP_013273542.1 197   rubrerythrin [[Clostridium] saccharolyticum]
CCZ62820.1   197   rubrerythrin [Clostridium hathewayi CAG:224]
WP_024294370.1 197   MULTISPECIES: hypothetical protein[Lachnoclostridium]
WP_002601930.1 197   rubrerythrin [Hungatella hathewayi]
AGY52891.1   197   Reverse rubrerythrin-1 [Bacteroidales bacterium CF]
CDE72958.1   197   rubrerythrin [Acidaminococcus sp. CAG:917]
WP_007785315.1 197   rubrerythrin [Desulfosporosinus youngiae]
WP_007938359.1 197   MULTISPECIES: rubrerythrin [Pelosinus]
CDB29423.1   197   putative uncharacterized protein [Firmicutes

bacterium CAG:137]
WP_026882448.1 196   rubrerythrin [Clostridium akagii]
WP_015425079.1 196   rubrerythrin [Candidatus Cloacimonas acidaminovorans]
WP_052221581.1 196   rubrerythrin [Clostridium homopropionicum]
EFW30727.1   196   Rubrerythrin [Selenomonas artemidis F0399]
WP_006489955.1 196   Rubrerythrin-1 [Mesotoga infera]
WP_047754207.1 196   rubrerythrin [Kosmotoga pacifica]
WP_014731759.1 196   rubrerythrin [Mesotoga prima]
WP_024614127.1 196   rubrerythrin [Clostridium sp. Ade.TY]
WP_012160644.1 196   rubrerythrin [Alkaliphilus oremlandii]
WP_039249338.1 196   rubrerythrin [Clostridium novyi]
WP_011721161.1 196   MULTISPECIES: rubrerythrin [Clostridium]
WP_002599047.1 196   hypothetical protein [Clostridium colicanis]
WP_008517480.1 196   rubrerythrin [Dethiobacter alkaliphilus]
WP_039227137.1 196   rubrerythrin [Clostridium novyi]
WP_003375224.1 196   MULTISPECIES: rubrerythrin [Clostridium]
WP_009525212.1 196   rubrerythrin [Peptostreptococcaceae bacterium ACC19a]
WP_004834635.1 196   rubrerythrin [Anaerococcus prevotii]
WP_009527933.1 196   MULTISPECIES: rubrerythrin [unclassified Peptostreptococcaceae (miscellaneous)]
WP_012861712.1 196   rubrerythrin [Sebaldella termitidis]
WP_013724406.1 196   rubrerythrin [Clostridium botulinum]
WP_039277127.1 196   rubrerythrin [Clostridium botulinum]
WP_039257555.1 196   rubrerythrin [Clostridium botulinum]
WP_019279371.1 196   rubrerythrin [Clostridium botulinum]
WP_004836638.1 196   rubrerythrin [Anaerococcus tetradius]
WP_039216895.1 196   rubrerythrin [Clostridium novyi]
WP_039255809.1 196   rubrerythrin [Clostridium novyi]
WP_002599578.1 196   rubrerythrin [Clostridium colicanis]
WP_015778574.1 196   rubrerythrin [Anaerococcus prevotii]
WP_003367634.1 196   rubrerythrin [Clostridium botulinum]
WP_007062895.1 196   rubrerythrin [Clostridium carboxidivorans]
WP_007784838.1 196   rubrerythrin [Desulfosporosinus youngiae]
WP_034438336.1 196   rubrerythrin [Candidatus Clostridium anorexicamassiliense]
WP_027633091.1 196   rubrerythrin [Clostridium hydrogeniformans]
WP_055665415.1 196   rubrerythrin [Clostridiaceae bacterium mt10]
WP_011196452.1 196   rubrerythrin [Symbiobacterium thermophilum]
WP_039679875.1 196   rubrerythrin [Terrisporobacter othiniensis]
WP_041240130.1 196   rubrerythrin [Gordonibacter pamelaeae]
ACR79556.1   196   Rubrerythrin [Kosmotoga olearia TBF 19.5.1]
WP_040191566.1 196   rubrerythrin [Clostridium sp. CL-6]
WP_005213790.1 196   rubrerythrin [Clostridium celatum]
WP_040197927.1 196   hypothetical protein [Candidatus Soleaferrea massiliensis]
WP_012423951.1 196   rubrerythrin [Clostridium botulinum]
WP_012449659.1 196   rubrerythrin [Clostridium botulinum]
WP_041081723.1 196   rubrerythrin [Clostridium botulinum]

WP_003369352.1 196   rubrerythrin [Clostridium botulinum]
WP_021875252.1 196   rubrerythrin [Clostridium chauvoei]
WP_048571041.1 196   rubrerythrin [Clostridium cylindrosporum]
WP_005213308.1 196   rubredoxin [Clostridium celatum]
WP_017352850.1 196   rubrerythrin [Clostridium botulinum]
WP_002580745.1 196   MULTISPECIES: rubrerythrin [Clostridium]
WP_003425793.1 196   rubrerythrin [Clostridium butyricum]
WP_027636665.1 196   hypothetical protein [Clostridium butyricum]
WP_035764252.1 196   rubrerythrin [Clostridium butyricum]
CDD68406.1   196   rubrerythrin family protein [Firmicutes bacterium CAG:475]
WP_055233448.1 196   MULTISPECIES: rubrerythrin [Firmicutes]
WP_022000839.1 196   rubredoxin [Coprobacillus sp. 8_1_38FAA]
CDE28633.1   196   rubredoxin [Catenibacterium sp. CAG:290]
WP_006504690.1 196   rubrerythrin [Catenibacterium mitsuokai]
WP_027089181.1 196   rubrerythrin [[Clostridium] saccharogumia]
WP_024721137.1 196   rubrerythrin [Clostridiales bacterium VE202-01]
CDB87714.1   196   rubredoxin [Firmicutes bacterium CAG:170]
CDD64621.1   196   putative uncharacterized protein [Firmicutes bacterium CAG:882]
WP_004853916.1 196   rubrerythrin [Coprococcus eutactus]
WP_055224152.1 196   rubrerythrin [Coprococcus eutactus]
WP_015534780.1 196   rubrerythrin [Coprococcus eutactus]
WP_010965865.1 195   Rubrerythrin-1 [Clostridium acetobutylicum]
WP_034581317.1 195   rubrerythrin [Clostridium acetobutylicum]
WP_011973045.1 195   rubrerythrin [Methanococcus aeolicus]
WP_035147352.1 195   rubrerythrin [Clostridium tetanomorphum]
WP_011100747.1 195   rubrerythrin [Clostridium tetani]
WP_029452095.1 195   rubrerythrin [Clostridium algidicarnis]
WP_035307029.1 195   rubrerythrin [Clostridium sp. HMP27]
WP_034570737.1 195   rubrerythrin [Clostridiales bacterium oral taxon 876]
WP_053242994.1 195   rubrerythrin [Clostridium sp. DMHC 10]
WP_023439639.1 195   rubrerythrin [Clostridium tetani]
AEG18329.1   195   Rubrerythrin [Methanobacterium paludis]
WP_048081127.1 195   MULTISPECIES: rubrerythrin [Methanobacterium]
WP_004819573.1 195   rubrerythrin [Peptoniphilus indolicus]
WP_008787282.1 195   MULTISPECIES: rubrerythrin [Coprobacillus]
WP_035289291.1 195   rubrerythrin [Clostridium sp. KNHs214]
WP_027632838.1 195   rubrerythrin [Clostridium hydrogeniformans]
WP_028044495.1 195   rubrerythrin [Candidatus Stoquefichus massiliensis]
WP_050637831.1 195   rubrerythrin [Candidatus Stoquefichus sp. SB1]
WP_017415060.1 195   rubrerythrin [Clostridium tunisiense]
WP_003397458.1 195   MULTISPECIES: rubrerythrin [Clostridium]
WP_003486830.1 195   MULTISPECIES: rubrerythrin [Clostridium]
WP_041350334.1 195   rubrerythrin [Clostridium botulinum]
WP_012705447.1 195   rubrerythrin [Clostridium botulinum]
WP_003358705.1 195   MULTISPECIES: rubrerythrin [Clostridium]

WP_032122930.1	195	rubrerythrin [Clostridium sp. LF2]
WP_010234282.1	195	rubrerythrin [Clostridium arbusti]
AJD25952.1	195	rubrerythrin-1 [Clostridium botulinum CDC_297]
WP_040215197.1	195	rubrerythrin [Clostridium polynesiense]
WP_053943152.1	195	rubrerythrin [Kallipyga sp. GM4]
WP_003448599.1	195	rubrerythrin [Clostridium perfringens]
WP_021284195.1	195	rubrerythrin [Clostridium sp. BL8]
WP_011009633.1	195	rubrerythrin [Clostridium perfringens]
WP_027634907.1	195	rubrerythrin [Clostridium butyricum]
WP_010074167.1	195	rubrerythrin [Clostridium cellulovorans]
ETI88045.1	195	Rubrerythrin [Clostridium butyricum DORA_1]
WP_019134176.1	195	hypothetical protein [Kallipyga massiliensis]
WP_023388643.1	195	rubrerythrin [Youngiibacter fragilis]
ABG86250.1	195	rubrerythrin [Clostridium perfringens SM101]
WP_002581773.1	195	MULTISPECIES: rubrerythrin [Clostridium]
WP_040107968.1	195	MULTISPECIES: rubrerythrin [Clostridium]
WP_008676349.1	195	rubrerythrin [Clostridium sp. 7_2_43FAA]
WP_013867741.1	195	rubrerythrin [Methanothermococcus okinawensis]
WP_053469386.1	195	MULTISPECIES: rubrerythrin [Clostridium]
WP_003495500.1	195	MULTISPECIES: rubrerythrin [Clostridium]
WP_005366636.1	195	rubrerythrin [[Eubacterium] yurii]
WP_032123266.1	195	rubrerythrin [Clostridium sp. LF2]
WP_027626538.1	195	rubrerythrin [Clostridium lundense]
WP_048202130.1	195	rubrerythrin [Methanocaldococcus sp. JH146]
WP_003467787.1	195	rubrerythrin [Clostridium perfringens]
WP_011967638.1	195	rubrerythrin [Clostridium beijerinckii]
CAA63429.1	195	rubrerythrin [Clostridium perfringens]
WP_040193860.1	195	rubrerythrin [Clostridium sp. CL-6]
KKP69144.1	195	Rubrerythrin [Parcubacteria (Moranbacteria) bacterium GW2011_GWE1_35_17]
WP_048197508.1	195	rubrerythrin [Methanocaldococcus sp. FS406-22]
EKE04656.1	195	hypothetical protein ACD_20C00033G0006 [uncultured bacterium]
WP_017209758.1	195	MULTISPECIES: rubrerythrin [Clostridium]
WP_007045226.1	195	rubrerythrin [Methanotorris formicicus]
EKE21981.1	195	rubrerythrin [uncultured bacterium]
WP_026886601.1	195	rubrerythrin [Clostridium beijerinckii]
KKS26051.1	195	Rubrerythrin [Parcubacteria bacterium GW2011_GWA2_42_11]
WP_015732516.1	195	rubrerythrin [Methanocaldococcus vulcanius]
WP_021802994.1	195	rubrerythrin [Clostridium intestinale]
WP_039312478.1	195	rubrerythrin [Clostridium baratii]
KKP57063.1	195	Rubrerythrin [Parcubacteria (Moranbacteria) bacterium GW2011_GWF1_34_10]
WP_055070005.1	195	rubrerythrin [Clostridium sp. ND2]
WP_051639689.1	195	rubrerythrin [Succinivibrio dextrinosolvens]
WP_045725099.1	195	rubrerythrin [Clostridium baratii]
WP_054200576.1	195	rubrerythrin [Clostridium baratii]
WP_022852296.1	195	rubrerythrin [Geovibrio sp. L21-Ace-BES]
KPJ85585.1	195	rubrerythrin [Spirochaetes bacterium DG_61]

WP_018107070.1	195	hypothetical protein [Porphyromonas bennonis]
WP_045510404.1	195	rubrerythrin [bacterium UASB14]
WP_016208086.1	195	rubrerythrin [Clostridium sartagoforme]
WP_047000398.1	195	rubrerythrin [Clostridium sp. C8]
WP_055208206.1	195	rubrerythrin [Clostridium baratii]
WP_025748647.1	195	rubrerythrin [Caldicoprobacter oshimai]
WP_039635033.1	195	rubrerythrin [Clostridium argentinense]
WP_044036036.1	195	rubrerythrin [Clostridium bornimense]
WP_017413669.1	195	hypothetical protein [Clostridium tunisiense]
WP_009169364.1	195	rubrerythrin [Clostridium sp. DL-VIII]
WP_053241008.1	195	rubrerythrin [Clostridium sp. DMHC 10]
AFV80078.1	195	rubrerythrin 2 [Spironucleus salmonicida]
KJS50719.1	195	rubrerythrin [Peptococcaceae bacterium BRH_c23]
WP_004622163.1	195	rubrerythrin [[Clostridium] papyrosolvens]
WP_040191462.1	195	rubrerythrin [Clostridium sp. CL-6]
WP_020816181.1	195	rubrerythrin [[Clostridium] papyrosolvens]
EST48649.1	195	Rubrerythrin 1 [Spironucleus salmonicida]
AFV80077.1	195	rubrerythrin 1 [Spironucleus salmonicida]
WP_003441864.1	195	hypothetical protein [Clostridium pasteurianum]
WP_041893607.1	195	rubrerythrin [Clostridium beijerinckii]
WP_055078836.1	195	rubrerythrin [Peptoniphilaceae bacterium SIT14]
WP_022749627.1	195	rubrerythrin [Clostridium saccharobutylicum]
WP_045574133.1	195	rubrerythrin [Desulfosporosinus sp. I2]
WP_055070012.1	195	rubrerythrin [Clostridium sp. ND2]
WP_006716294.1	195	rubrerythrin [Desulfitobacterium metallireducens]
WP_021282507.1	195	hypothetical protein [Clostridium sp. BL8]
WP_014182750.1	195	rubrerythrin [Desulfosporosinus orientis]
WP_007778297.1	195	rubrerythrin [Desulfosporosinus youngiae]
WP_034598280.1	195	rubrerythrin [Desulfosporosinus sp. HMP52]
WP_003426696.1	195	MULTISPECIES: rubrerythrin [Peptoclostridium]
WP_021385797.1	195	rubrerythrin-1 [Peptoclostridium difficile]
WP_003422762.1	195	rubrerythrin [Peptoclostridium difficile]
WP_021397258.1	195	rubrerythrin-1 [Peptoclostridium difficile]
WP_054274819.1	195	rubrerythrin [Peptoclostridium difficile]
WP_054277428.1	195	rubrerythrin [Peptoclostridium difficile]
WP_054271427.1	195	rubrerythrin [Peptoclostridium difficile]
WP_014901196.1	195	rubrerythrin [Desulfosporosinus meridiei]
CDB16699.1	195	rubrerythrin [Clostridium sp. CAG:221]
WP_009622261.1	195	rubrerythrin [Desulfosporosinus sp. OT]
CCX43137.1	195	rubrerythrin [Prevotella sp. CAG:1031]
WP_049177092.1	195	rubrerythrin [Clostridium botulinum]
WP_027639619.1	195	hypothetical protein [Clostridium cadaveris]
EDS06280.1	195	Rubrerythrin [[[Clostridium] scindens ATCC 35704]
CDC20813.1	195	putative uncharacterized protein [Eubacterium sp.CAG:274]

CDB75290.1	195	rubrerythrin [Clostridium sp. CAG:265]
ADK32062.1	195	rubrerythrin [Brachyspira pilosicoli 95/1000]
WP_028246002.1	195	rubrerythrin [Pseudobutyrivibrio ruminis]
WP_044974021.1	195	rubrerythrin [Ruminococcus sp. HUN007]
WP_012741214.1	195	MULTISPECIES: rubrerythrin [Clostridiales]
CDE45008.1	195	rubredoxin [Clostridium sp. CAG:411]
WP_007885721.1	195	rubrerythrin [Roseburia inulinivorans]
WP_055168373.1	195	rubrerythrin [Roseburia inulinivorans]
WP_050623394.1	195	rubrerythrin [Clostridium sp. GD3]
WP_021923073.1	195	hypothetical protein [Roseburia inulinivorans]
CCY43566.1	195	putative uncharacterized protein [Firmicutes bacterium CAG:124]
CDA24614.1	195	putative uncharacterized protein [Roseburia sp. CAG:197]
CBK75078.1	195	Rubrerythrin [Butyrivibrio fibrisolvens 16/4]
CDA87883.1	195	putative uncharacterized protein [Clostridium sp.CAG:230]
CCZ08565.1	195	rubredoxin [Clostridium sp. CAG:127]
WP_031543913.1	195	rubrerythrin [Lachnospiraceae bacterium AC2014]
CCZ55488.1	195	rubrerythrin [Clostridium sp. CAG:75]
WP_033153398.1	195	rubrerythrin [Pseudobutyrivibrio ruminis]
WP_044937091.1	195	rubrerythrin [Pseudobutyrivibrio sp. LB2011]
WP_028242037.1	195	rubrerythrin [Pseudobutyrivibrio ruminis]
WP_033119453.1	195	rubrerythrin [Oscillibacter sp. ER4]
CCZ46887.1	195	rubredoxin [Firmicutes bacterium CAG:129]
WP_028234759.1	195	rubrerythrin [Pseudobutyrivibrio sp. MD2005]
WP_048060893.1	194	rubrerythrin [Methanothermobacter thermautotrophicus]
WP_048175476.1	194	rubrerythrin [Methanothermobacter sp. CaT2]
WP_013295965.1	194	rubrerythrin [Methanothermobacter marburgensis]
WP_053954905.1	194	rubrerythrin [Clostridiaceae bacterium mt12]
WP_018086033.1	194	rubrerythrin [Desulfurispora thermophila]
WP_023991981.1	194	rubrerythrin [Methanobacterium sp. MB1]
WP_003536067.1	194	MULTISPECIES: rubrerythrin [Erysipelotrichaceae]
WP_035379593.1	194	rubrerythrin [Fervidicella metallireducens]
WP_004609596.1	194	rubrerythrin [[Clostridium] spiroforme]
WP_027309207.1	194	rubrerythrin [Caloramator sp. ALD01]
WP_021169478.1	194	MULTISPECIES: rubrerythrin-1 [Sporomusa]
WP_013036546.1	194	rubrerythrin [Methanohalophilus mahii]
CEL24973.1	194	putative rubrerythrin [Methanobacterium formicicum]
WP_048073111.1	194	rubrerythrin [Methanobacterium formicicum]
WP_012035722.1	194	rubrerythrin [Methanocella arvoryzae]
WP_012102369.1	194	rubrerythrin [Clostridium kluyveri]
WP_008909893.1	194	rubrerythrin [Caloramator australicus]
WP_048187977.1	194	rubrerythrin [Methanobacterium paludis]
WP_012104101.1	194	rubrerythrin [Clostridium kluyveri]

WP_014406992.1	194	rubrerythrin [Methanocella conradii]
WP_004029775.1	194	rubrerythrin [Methanobacterium formicicum]
WP_050354595.1	194	rubrerythrin [[Clostridium] purinilyticum]
WP_057978474.1	194	rubrerythrin [Caloramator mitchellensis]
WP_053962820.1	194	rubrerythrin [Clostridiales bacterium mt11]
KPJ70890.1	194	rubrerythrin [Microgenomates bacterium DG_75]
WP_012068175.1	194	rubrerythrin [Methanococcus maripaludis]
BAI60400.1	194	rubrerythrin [Methanocella paludicola SANAE]
WP_011868731.1	194	rubrerythrin [Methanococcus maripaludis]
WP_012193057.1	194	rubrerythrin [Methanococcus maripaludis]
WP_012291219.1	194	rubrerythrin [Finegoldia magna]
CDE27133.1	194	rubrerythrin [Catenibacterium sp. CAG:290]
WP_006504321.1	194	rubrerythrin [Catenibacterium mitsuokai]
WP_002838770.1	194	rubrerythrin [Finegoldia magna]
WP_055233608.1	194	MULTISPECIES: rubrerythrin [Firmicutes]
WP_042273934.1	194	rubrerythrin [Clostridium dakarense]
WP_002835979.1	194	rubrerythrin [Finegoldia magna]
WP_034864345.1	194	rubrerythrin [[Clostridium] saccharogumia]
KPQ45173.1	194	rubrerythrin [Candidatus Methanoperedens sp. BLZ1]
WP_055665096.1	194	rubrerythrin [Clostridiaceae bacterium mt10]
WP_004801402.1	194	hypothetical protein [Eggerthia catenaformis]
WP_045439238.1	194	rubrerythrin [bacterium UASB270]
WP_021676331.1	194	rubrerythrin [Peptostreptococcaceae bacterium oral taxon 113]
WP_018666542.1	194	rubrerythrin [Thermobrachium celere]
WP_020001332.1	194	rubrerythrin [Desulfovibrio desulfuricans]
WP_045078374.1	194	rubrerythrin [Peptoniphilus sp. 1-1]
KKK44578.1	194	Rubrerythrin-1 [Lokiarchaeum sp. GC14_75]
XP_004260064.1	194	hypothetical protein EIN_056780 [Entamoeba invadens IP1]
WP_017751342.1	194	rubrerythrin [Clostridium tyrobutyricum]
WP_027361186.1	194	rubrerythrin [Desulfovibrio acrylicus]
WP_039656829.1	194	rubrerythrin [Clostridium tyrobutyricum]
WP_015791604.1	194	rubrerythrin [Methanocaldococcus fervens]
KQC06946.1	194	rubrerythrin [Candidatus Cloacimonas sp. SDB]
WP_042678921.1	194	rubrerythrin [Anaerosalibacter sp. ND1]
WP_012066371.1	194	rubrerythrin [Methanococcus vannielii]
XP_001583405.1	194	rubrerythrin [Trichomonas vaginalis G3]
EKD24960.1	194	rubrerythrin [uncultured bacterium (gcode 4)]
WP_016647447.1	194	hypothetical protein [Campylobacter ureolyticus]
CDC20612.1	194	putative uncharacterized protein [Clostridium sp.CAG:306]
WP_018713367.1	194	hypothetical protein [Campylobacter ureolyticus]
WP_024962021.1	194	rubrerythrin [Campylobacter ureolyticus]
WP_014314692.1	194	rubrerythrin [Clostridium sp. BNL1100]
WP_023790756.1	194	rubrerythrin [candidate division SR1 bacterium RAAC1_SR1_1]

WP_029069983.1 194   rubrerythrin [Kandleria vitulina]
WP_014792350.1 194   MULTISPECIES: rubrerythrin
[Desulfitobacterium]
WP_005810244.1 194   rubrerythrin [Desulfitobacterium hafniense]
WP_015942738.1 194   rubrerythrin [Desulfitobacterium hafniense]
EEG74188.1   194   rubredoxin [[[Clostridium] hylemonae DSM
15053]
WP_015924425.1 194   rubrerythrin [[Clostridium] cellulolyticum]
WP_024833214.1 194   rubrerythrin [[Clostridium] josui]
WP_027623852.1 194   rubrerythrin [Clostridium lundense]
WP_015260947.1 194   rubrerythrin [Desulfitobacterium
dichloroeliminans]
WP_014966319.1 194   rubrerythrin [Gottschalkia acidurici]
WP_007049444.1 194   hypothetical protein [Anaerofustis
stercorihominis]
WP_033162047.1 194   rubrerythrin [Sharpea azabuensis]
WP_008864613.1 194   rubrerythrin [Parasutterella
excrementihominis]
WP_008811183.1 194   rubrerythrin [Burkholderiales bacterium
1_1_47]
CDA42539.1   194   rubrerythrin [Proteobacteria bacterium CAG:
139]
XP_001303688.1 194   Rubrerythrin family protein [Trichomonas
vaginalis G3]
WP_042294422.1 194   rubrerythrin [Candidatus Arthromitus sp.SFB-
mouse-NL]
CAG36385.1   194   hypothetical protein DP1656 [Desulfotalea
psychrophila LSv54]
WP_014094445.1 194   rubrerythrin [Candidatus Arthromitus sp. SFB-
rat-Yit]
WP_024722097.1 194   hypothetical protein [Clostridiales bacterium
VE202-01]
WP_005806718.1 194   rubrerythrin [Candidatus Arthromitus sp. SFB-
mouse]
WP_027090097.1 194   hypothetical protein [[Clostridium]
saccharogumia]
WP_022444130.1 194   rubrerythrin [Sutterellaceae bacterium ND3]
WP_004608819.1 194   hypothetical protein [[Clostridium]
spiroforme]
WP_003538076.1 194   MULTISPECIES: hypothetical
protein[Erysipelotrichaceae]
XP_001303689.1 194   Rubrerythrin family protein [Trichomonas
vaginalis G3]
CDE71254.1   194   rubrerythrin [Subdoligranulum sp. CAG:314]
CCZ28117.1   194   putative uncharacterized protein [Firmicutes
bacterium CAG:194]
CDF46757.1   194   putative uncharacterized protein [Roseburia
sp. CAG:100]
CCX86847.1   194   putative uncharacterized protein [Clostridium
sp.CAG:590]

CDA89391.1   194   putative uncharacterized protein [Firmicutes bacterium CAG:238]
WP_016300088.1 194   hypothetical protein [Lachnospiraceae bacterium COE1]
WP_048190906.1 193   rubrerythrin [Methanobacterium sp. SMA-27]
WP_013195453.1 193   rubrerythrin [Methanohalobium evestigatum]
WP_013644532.1 193   rubrerythrin [Methanobacterium lacus]
WP_048189918.1 193   rubrerythrin [Methanobacterium sp. SMA-27]
WP_048815420.1 193   rubrerythrin [Methanosalsum zhilinae]
WP_013683342.1 193   rubrerythrin [Archaeoglobus veneficus]
WP_011170577.1 193   rubrerythrin [Methanococcus maripaludis]
WP_013999024.1 193   rubrerythrin [Methanococcus maripaludis]
WP_048187053.1 193   rubrerythrin [Methanococcus voltae]
WP_048570523.1 193   rubrerythrin [Clostridium cylindrosporum]
WP_036608664.1 193   rubrerythrin [Oribacterium sp. P6A1]
WP_037564949.1 193   rubrerythrin [Spirochaeta sp. JC202]
WP_035117647.1 193   rubrerythrin [Clostridium sp. NCR]
WP_019213869.1 193   hypothetical protein [Fenollaria massiliensis]
KHO53696.1   193   Rubrerythrin [archaeon GW2011_AR13]
GAK35308.1   193   rubrerythrin [Bacteroides graminisolvens DSM 19988 = JCM 15093]
WP_014259880.1 193   rubrerythrin [Desulfovibrio africanus]
WP_032118181.1 193   rubrerythrin [Clostridium sp. CL-2]
WP_055263379.1 193   rubrerythrin [Clostridium disporicum]
WP_005987301.1 193   rubrerythrin [Desulfovibrio africanus]
WP_055206382.1 193   rubrerythrin [Faecalibacterium prausnitzii]
WP_055432986.1 193   rubrerythrin [Candidatus Symbiothrix dinenymphae]
WP_013100292.1 193   rubrerythrin [Methanocaldococcus infernus]
WP_019246297.1 193   hypothetical protein [Candidatus Alistipes marseilloanorexicus]
WP_021428250.1 193   MULTISPECIES: rubrerythrin-1 [Clostridiales]
WP_021431612.1 193   MULTISPECIES: rubrerythrin-1 [Clostridiales]
WP_034548053.1 193   rubrerythrin [Tissierellia bacterium S7-1-4]
WP_057556863.1 193   rubrerythrin [[Clostridium] sordellii]
WP_021127477.1 193   rubrerythrin-1 [[Clostridium] sordellii]
WP_057538964.1 193   rubrerythrin [[Clostridium] sordellii]
CEN80436.1   193   Rbr1 [[Clostridium] sordellii]
WP_004627444.1 193   rubrerythrin [[Clostridium] termitidis]
WP_027631231.1 193   rubrerythrin [[Clostridium] cellobioparum]
EFE92461.1   193   Rubrerythrin [Oribacterium sp. oral taxon 078 str. F0262]
WP_004331485.1 193   rubrerythrin [Porphyromonas asaccharolytica]
WP_013760174.1 193   rubrerythrin [Porphyromonas asaccharolytica]
WP_039865730.1 193   rubrerythrin [Porphyromonas uenonis]
WP_025882873.1 193   rubrerythrin [Porphyromonas uenonis]
CCZ95241.1   193   rubrerythrin [Corallococcus sp. CAG:1435]
WP_008982391.1 193   rubrerythrin [Ruminococcaceae bacterium D16]
WP_027717144.1 192   rubrerythrin [Desulfovirgula thermocuniculi]
KPL03376.1   192   rubrerythrin [Planctomycetes bacterium

SM23_65]
WP_015751640.1 192   rubrerythrin [Desulfohalobium retbaense]
WP_041267599.1 192   rubrerythrin [Geobacter daltonii]
WP_054693833.1 192   rubrerythrin [Geobacter toluenoxydans]
WP_049765069.1 192   rubrerythrin [Syntrophothermus lipocalidus]
WP_006002602.1 192   rubrerythrin [Desulfuromonas acetoxidans]
CDE59974.1   192   rubrerythrin [Fusobacterium sp. CAG:439]
CDE88976.1   192   rubrerythrin [Clostridium sp. CAG:729]
WP_046860043.1 192   rubrerythrin [Sedimenticola sp. SIP-G1]
WP_053951611.1 192   rubrerythrin [Candidatus Thioglobus sp. EF1]
WP_041064865.1 192   rubrerythrin [Thiolapillus brandeum]
WP_020448427.1 192   rubrerythrin [Candidatus Methanomassiliicoccus
intestinalis]
WP_015905989.1 192   rubrerythrin [Desulfobacterium autotrophicum]
WP_004328033.1 192   rubrerythrin [Alistipes putredinis]
WP_006475597.1 192   MULTISPECIES: rubrerythrin [sulfur-oxidizing
symbionts]
CCY62996.1   192   putative uncharacterized protein [Clostridium
sp.CAG:967]
WP_054648405.1 192   rubrerythrin [Desulfovibrio brasiliensis]
KQC11140.1   192   rubrerythrin [Methanolinea sp. SDB]
WP_029132193.1 192   rubrerythrin [Sedimenticola selenatireducens]
KPK64549.1   192   rubrerythrin [candidate division WOR_3
bacterium SM23_42]
ADI35996.1   192   Rubrerythrin [Methanococcus voltae A3]
WP_028576899.1 192   rubrerythrin [Desulfomicrobium escambiense]
EEZ80762.1   192   rubrerythrin [uncultured SUP05 cluster
bacterium]
WP_055205701.1 192   rubrerythrin [Faecalibacterium prausnitzii]
WP_013611354.1 192   rubrerythrin [Odoribacter splanchnicus]
WP_045031825.1 192   rubrerythrin [Draconibacterium sp. JN14CK-3]
WP_034595380.1 192   rubrerythrin [Clostridiales bacterium
VE202-01]
CCX80871.1   192   putative uncharacterized protein [Clostridium
sp.CAG:715]
WP_011965064.1 192   rubrerythrin [Bacteroides vulgatus]
WP_055098652.1 192   rubrerythrin [Porphyromonadaceae bacterium
GM3]
CDC96489.1   192   rubrerythrin [Alistipes sp. CAG:268]
KPL14761.1   192   rubrerythrin [candidate division WOR_3
bacterium SM1_77]
WP_009135412.1 192   rubrerythrin [Odoribacter laneus]
WP_021930938.1 192   rubrerythrin [Coprobacter secundus]
WP_022663807.1 192   rubrerythrin [Desulfospira joergensenii]
WP_037464848.1 192   rubrerythrin [Smithella sp. F21]
WP_040625472.1 192   rubrerythrin [Smithella sp. ME-1]
WP_054721122.1 192   rubrerythrin [Marinifilum fragile]
CDB15163.1   192   rubrerythrin [Clostridium sp. CAG:221]
WP_005844074.1 192   MULTISPECIES: rubrerythrin [Bacteroides]
WP_041502387.1 192   rubrerythrin [Sanguibacteroides justesenii]

WP_038657166.1 192   rubrerythrin [Mucinivorans hirudinis]
WP_026207370.1 192   rubrerythrin [Butyricimonas synergistica]
CDD83474.1   192   uncharacterized protein BN666_02754
[Bacteroides sp.CAG:462]
WP_037378405.1 192   MULTISPECIES: rubrerythrin [Smithella]
WP_008624744.1 192   rubrerythrin [Paraprevotella xylaniphila]
WP_005945048.1 192   hypothetical protein [Bacteroides
massiliensis]
CDB75950.1   192   rubrerythrin [Clostridium sp. CAG:265]
KFO68809.1   192   rubrerythrin [Smithella sp. SCADC]
WP_012469852.1 192   rubrerythrin [Geobacter lovleyi]
WP_009860442.1 192   MULTISPECIES: rubrerythrin [Parabacteroides]
CCX62970.1   192   rubrerythrin [Bacteroides sp. CAG:598]
WP_022601499.1 192   rubrerythrin [Coprobacter fastidiosus]
WP_005825226.1 192   MULTISPECIES: rubrerythrin [Bacteria]
WP_009134041.1 192   rubrerythrin [Alistipes indistinctus]
CCZ47033.1   192   rubrerythrin [Bacteroides sp. CAG:661]
WP_013218628.1 192   rubrerythrin [Dehalogenimonas
lykanthroporepellens]
KPK01195.1   192   rubrerythrin [Nitrospira bacterium SG8_35_4]
WP_058018644.1 192   rubrerythrin [Porphyromonas gingivalis]
WP_009317760.1 192   rubrerythrin [Tannerella sp. 6_1_58FAA_CT1]
WP_006743866.1 192   rubrerythrin [Bacteroides coprosuis]
WP_012457408.1 192   rubrerythrin [Porphyromonas gingivalis]
WP_009125993.1 192   rubrerythrin [Bacteroides fluxus]
WP_008619344.1 192   rubrerythrin [Paraprevotella clara]
WP_021665268.1 192   rubrerythrin [Porphyromonas gingivalis]
WP_010955948.1 192   rubrerythrin [Porphyromonas gingivalis]
WP_004291818.1 192   rubrerythrin [Bacteroides eggerthii]
WP_026292353.1 192   rubrerythrin [Porphyromonas gulae]
WP_039418376.1 192   rubrerythrin [Porphyromonas gulae]
WP_004583836.1 192   rubrerythrin [Porphyromonas gingivalis]
WP_018665624.1 192   rubrerythrin [Bacteroides gallinarum]
WP_016660952.1 192   hypothetical protein [Bacteroides stercoris]
CDB10421.1   192   rubrerythrin [Bacteroides sp. CAG:633]
WP_024995507.1 192   rubrerythrin [Bacteroides graminisolvens]
WP_039422388.1 192   rubrerythrin [Porphyromonas gulae]
WP_039437874.1 192   rubrerythrin [Porphyromonas gulae]
WP_004333850.1 192   rubrerythrin [Porphyromonas endodontalis]
WP_022546262.1 192   rubrerythrin [Bacteroidales bacterium CF]
WP_023847048.1 192   rubrerythrin [Porphyromonas gingivalis]
WP_024992900.1 192   rubrerythrin [Bacteroides
paurosaccharolyticus]
WP_039429863.1 192   rubrerythrin [Porphyromonas sp. COT-052
OH4946]
WP_004312048.1 192   MULTISPECIES: rubrerythrin [Bacteroides]
CCY92353.1   192   rubrerythrin [Bacteroides sp. CAG:1076]
CDA76752.1   192   rubrerythrin [Bacteroides sp. CAG:530]
CDB98559.1   192   rubrerythrin [Bacteroides sp. CAG:443]
CDE99170.1   192   rubrerythrin [Clostridium sp. CAG:813]

WP_021677129.1	192	rubrerythrin [Porphyromonas gingivalis]
WP_013618245.1	192	rubrerythrin [Bacteroides salanitronis]
WP_045090513.1	192	MULTISPECIES: rubrerythrin [Bacteria]
WP_010664166.1	192	rubrerythrin [Marinilabilia salmonicolor]
WP_039435611.1	192	rubrerythrin [Porphyromonas gulae]
WP_005655746.1	192	rubrerythrin [Bacteroides stercoris]
CCZ10751.1	192	putative uncharacterized protein [Odoribacter sp.CAG:788]
WP_004302842.1	192	MULTISPECIES: rubrerythrin [Bacteroides]
WP_025076001.1	192	rubrerythrin [Bacteroides faecichinchillae]
WP_027200986.1	192	rubrerythrin [Butyricimonas virosa]
WP_039440090.1	192	rubrerythrin [Porphyromonas gulae]
WP_005783570.1	192	MULTISPECIES: rubrerythrin [Bacteroides]
WP_005676804.1	192	MULTISPECIES: rubrerythrin [Bacteroides]
WP_013546351.1	192	rubrerythrin [Bacteroides helcogenes]
WP_009121994.1	192	rubrerythrin [Bacteroides clarus]
WP_010535931.1	192	MULTISPECIES: rubrerythrin [Bacteroides]
CDD25246.1	192	rubrerythrin [Alistipes sp. CAG:29]
WP_025278600.1	192	rubrerythrin [Barnesiella viscericola]
WP_015546108.1	192	rubrerythrin [Alistipes shahii]
WP_018695974.1	192	MULTISPECIES: rubrerythrin [Alistipes]
WP_008762794.1	192	MULTISPECIES: rubrerythrin [Bacteroides]
WP_007570715.1	192	rubrerythrin [Bacteroides coprocola]
WP_040296285.1	192	rubrerythrin [Barnesiella intestinihominis]
WP_055201914.1	192	rubrerythrin [Alistipes finegoldii]
WP_015775618.1	192	rubrerythrin [Desulfomicrobium baculatum]
WP_022138411.1	192	hypothetical protein [Bacteroides acidifaciens]
CDE61019.1	192	rubrerythrin [Parabacteroides sp. CAG:409]
WP_009598975.1	192	MULTISPECIES: rubrerythrin [Alistipes]
WP_010259681.1	192	rubrerythrin [Alistipes timonensis]
WP_019150345.1	192	rubrerythrin [Alistipes senegalensis]
WP_002558465.1	192	MULTISPECIES: rubrerythrin [Bacteroides]
WP_032558772.1	192	rubrerythrin [Bacteroides fragilis]
WP_005782823.1	192	MULTISPECIES: rubrerythrin [Bacteroides]
WP_007211450.1	192	rubrerythrin [Bacteroides cellulosilyticus]
WP_043116341.1	192	rubrerythrin [Solemya velum gill symbiont]
WP_042366356.1	192	rubrerythrin [Bacteroidaceae bacterium MS4]
WP_007479583.1	192	rubrerythrin [Bacteroides salyersiae]
WP_045213164.1	192	rubrerythrin [Desulfonatronovibrio magnus]
KJR41924.1	192	rubrerythrin [Candidatus Magnetoovum chiemensis]
WP_007216470.1	192	MULTISPECIES: rubrerythrin [Bacteroides]
WP_055279612.1	192	rubrerythrin [Bacteroides finegoldii]
WP_004298806.1	192	MULTISPECIES: rubrerythrin [Bacteroides]
WP_044534913.1	192	rubrerythrin [Bacteroides intestinalis]
WP_007661066.1	192	rubrerythrin [Bacteroides intestinalis]
WP_012498715.1	192	rubrerythrin [Chloroherpeton thalassium]
WP_034542215.1	192	rubrerythrin [Bacteroides pyogenes]
WP_008139895.1	192	rubrerythrin [Bacteroides coprophilus]

WP_050701661.1 192   rubrerythrin [Dysgonomonas sp. BGC7]
WP_009130031.1 192   hypothetical protein [Bacteroides oleiciplenus]
WP_027325083.1 192   rubrerythrin [Bacteroides pyogenes]
WP_025831397.1 192   rubrerythrin [Bacteroides stercorirosoris]
WP_026473415.1 192   rubrerythrin [Alkaliflexus imshenetskii]
WP_029904633.1 192   rubrerythrin [Prevotella sp. 10(H)]
KSV18978.1   192   rubrerythrin [Dehalococcoides mccartyi]
WP_007559642.1 192   rubrerythrin [Bacteroides plebeius]
WP_006799462.1 192   rubrerythrin [Dysgonomonas gadei]
CDC55687.1   192   rubrerythrin [Bacteroides coprophilus CAG:333]
WP_006843541.1 192   rubrerythrin [Dysgonomonas mossii]
CDD52131.1   192   putative uncharacterized protein [Bacteroides sp.CAG:875]
WP_018360727.1 192   rubrerythrin [Porphyromonas macacae]
WP_018711445.1 192   rubrerythrin [Bacteroides barnesiae]
WP_028573838.1 192   rubrerythrin [Desulfonatronovibrio hydrogenovorans]
WP_036872789.1 192   rubrerythrin [Porphyromonas macacae]
WP_026627457.1 192   rubrerythrin [Dysgonomonas capnocytophagoides]
WP_036850539.1 192   rubrerythrin [Porphyromonas macacae]
WP_018124528.1 192   hypothetical protein [Desulfovibrio oxyclinae]
WP_019245360.1 192   rubrerythrin [Candidatus Alistipes marseilloanorexicus]
WP_023050095.1 192   hypothetical protein [Cetobacterium somerae]
WP_015423282.1 192   rubrerythrin [uncultured Termite group 1 bacterium]
EKD31048.1   192   rubrerythrin [uncultured bacterium]
WP_050710037.1 192   rubrerythrin [Dysgonomonas sp. HGC4]
WP_028729997.1 192   MULTISPECIES: rubrerythrin [Parabacteroides]
WP_005639590.1 192   rubrerythrin [Parabacteroides merdae]
WP_025863722.1 192   rubrerythrin [Prolixibacter bellariivorans]
WP_018109392.1 192   rubrerythrin [Bacteroides propionicifaciens]
CDE93045.1   192   rubrerythrin [Fusobacterium sp. CAG:815]
WP_047381627.1 192   MULTISPECIES: rubrerythrin [Cetobacterium]
WP_005860325.1 192   MULTISPECIES: rubrerythrin [Bacteroidales]
WP_008777696.1 192   MULTISPECIES: rubrerythrin [Bacteroidales]
WP_014587372.1 192   rubrerythrin [Methanosaeta harundinacea]
WP_025843304.1 192   rubrerythrin [Porphyromonas gingivicanis]
WP_052571249.1 192   rubrerythrin [Endomicrobium proavitum]
WP_008146779.1 192   rubrerythrin [Parabacteroides johnsonii]
EKQ56997.1   192   rubrerythrin [Clostridium sp. Maddingley MBC34-26]
KKR66677.1   192   Rubrerythrin [Microgenomates bacterium GW2011_GWA2_40_6]
EFB76574.1   192   rubredoxin [Subdoligranulum variabile DSM 15176]
GAP42074.1   192   Rubrerythrin [Bacteroidales bacterium TBC1]
WP_052537642.1 192   hypothetical protein [Clostridium sp. ATCC 29733]

ADK30736.1   192   rubrerythrin [Brachyspira pilosicoli 95/1000]
CDE82529.1   192   rubredoxin domain/rubrerythrin domain
protein[Clostridium sp. CAG:273]
WP_027291458.1 192   hypothetical protein [Rikenella microfusus]
WP_005035118.1 191   rubrerythrin [Holophaga foetida]
WP_048092148.1 191   rubrerythrin [Geoglobus acetivorans]
KKR04288.1   191   Rubrerythrin [Parcubacteria bacterium
GW2011_GWC2_39_14]
CBL28015.1   191   Rubrerythrin [Fretibacterium fastidiosum]
WP_048096694.1 191   rubrerythrin [Geoglobus ahangari]
WP_040199203.1 191   rubrerythrin [Geoalkalibacter subterraneus]
ACM21370.1   191   rubrerythrin [Geobacter daltonii FRC-32]
WP_028579509.1 191   rubrerythrin [Desulfobulbus japonicus]
KPJ48653.1   191   rubrerythrin [candidate division Zixibacteria
bacterium DG_27]
WP_019129500.1 191   rubrerythrin [Alistipes obesi]
WP_013899040.1 191   rubrerythrin [Methanosalsum zhilinae]
WP_011392800.1 191   rubrerythrin [Moorella thermoacetica]
WP_025323826.1 191   rubrerythrin [Deferrisoma camini]
WP_025774782.1 191   rubrerythrin [Moorella thermoacetica]
KPK68154.1   191   rubrerythrin [candidate division WOR_3
bacterium SM23_60]
WP_054936809.1 191   rubrerythrin [Moorella glycerini]
WP_013195540.1 191   rubrerythrin [Methanohalobium evestigatum]
WP_010454459.1 191   rubrerythrin [Succinivibrionaceae bacterium
WG-1]
WP_008862772.1 191   rubrerythrin [Barnesiella intestinihominis]
WP_011735840.1 191   rubrerythrin [Pelobacter propionicus]
WP_013313282.1 191   rubrerythrin [Spirochaeta thermophila]
WP_013969266.1 191   rubrerythrin [Treponema caldarium]
WP_040096808.1 191   rubrerythrin [Geoalkalibacter ferrihydriticus]
EFV17709.1   191   rubrerythrin [Lachnospiraceae bacterium
5_1_63FAA]
WP_014812030.1 191   rubrerythrin [Desulfomonile tiedjei]
WP_011500366.1 191   rubrerythrin [Methanococcoides burtonii]
WP_040334017.1 191   rubrerythrin [Candidatus Magnetobacterium
casensis]
KPJ53066.1   191   rubrerythrin [candidate division TA06
bacterium DG_24]
KJU82019.1   191   rubrerythrin [Candidatus Magnetobacterium
bavaricum]
WP_014624115.1 191   rubrerythrin [Spirochaeta thermophila]
WP_014955594.1 191   rubrerythrin [Desulfobacula toluolica]
KJJ85427.1   191   rubrerythrin [Candidatus Omnitrophus sp.
SKK-01]
WP_002672707.1 191   MULTISPECIES: hypothetical protein [Treponema]
WP_027938838.1 191   rubrerythrin [Anaerobiospirillum
succiniciproducens]
WP_013718127.1 191   rubrerythrin [Methanosaeta concilii]
WP_020880762.1 191   rubrerythrin [Desulfovibrio sp. X2]

WP_035241542.1	191	rubrerythrin [Desulfobacter vibrioformis]
WP_012414192.1	191	rubrerythrin [Elusimicrobium minutum]
EGC78877.1	191	rubrerythrin [Treponema denticola F0402]
WP_002681498.1	191	rubrerythrin [Treponema denticola]
WP_012765686.1	191	rubrerythrin [Desulfovibrio salexigens]
WP_049676848.1	191	rubrerythrin [Desulfocarbo indianensis]
WP_013258857.1	191	rubrerythrin [Desulfarculus baarsii]
WP_020589335.1	191	rubrerythrin [Desulfobacter curvatus]
KPJ76254.1	191	rubrerythrin [Deltaproteobacteria bacterium SG8_13]
WP_027985332.1	191	rubrerythrin [delta proteobacterium PSCGC 5451]
CBX27097.1	191	Rubrerythrin [uncultured Desulfobacterium sp.]
WP_015708567.1	191	rubrerythrin [Treponema primitia]
WP_053183603.1	191	rubrerythrin [Sunxiuqinia dokdonensis]
WP_027471821.1	191	rubrerythrin [Saccharicrinis fermentans]
WP_027354731.1	191	rubrerythrin [Desulfosarcina sp. BuS5]
WP_015947133.1	191	rubrerythrin [Desulfatibacillum alkenivorans]
WP_028313476.1	191	rubrerythrin [Desulfatibacillum aliphaticivorans]
WP_038060158.1	191	rubrerythrin [Thermodesulfobacterium commune]
WP_044824218.1	191	Rubrerythrin-2 [Clostridium aceticum]
WP_054700786.1	191	rubrerythrin [Desulfosarcina cetonica]
WP_005950880.1	191	rubrerythrin [Fusobacterium varium]
WP_002700510.1	191	rubrerythrin [Treponema phagedenis]
WP_010688638.1	191	hypothetical protein [Treponema denticola]
WP_015403152.1	191	rubrerythrin [Desulfocapsa sulfexigens]
CCY34689.1	191	rubrerythrin [Alistipes sp. CAG:831]
WP_022855442.1	191	rubrerythrin [Thermodesulfobacterium thermophilum]
KPA16419.1	191	rubrerythrin [Candidatus Magnetomorum sp. HK-1]
WP_010255723.1	191	rubrerythrin [Treponema primitia]
WP_025278832.1	191	rubrerythrin [Barnesiella viscericola]
WP_027179014.1	191	rubrerythrin [Desulfovibrio bastinii]
WP_028841102.1	191	rubrerythrin [Thermodesulfobacterium hveragerdense]
WP_013386504.1	191	rubrerythrin [Ilyobacter polytropus]
WP_004070586.1	191	rubrerythrin [Desulfobacter postgatei]
WP_005976226.1	191	rubrerythrin [Fusobacterium ulcerans]
WP_009532534.1	191	rubrerythrin [Stomatobaculum longum]
EKO38102.1	191	rubrerythrin [Desulfovibrio magneticus str. Maddingley MBC34]
WP_020886358.1	191	rubrerythrin [Desulfovibrio alkalitolerans]
WP_020965332.1	191	rubrerythrin [Treponema pedis]
WP_027190264.1	191	rubrerythrin [Desulfovibrio putealis]
WP_012421095.1	191	rubrerythrin [Akkermansia muciniphila]
WP_009318349.1	191	MULTISPECIES: rubrerythrin [Porphyromonadaceae]
WP_031931504.1	191	rubrerythrin [Akkermansia muciniphila]

WP_040868532.1 191 rubrerythrin [delta proteobacterium MLMS-1]
KPL05062.1 191 rubrerythrin [candidate division Zixibacteria bacterium SM1_73]
CDD93241.1 191 rubrerythrin [Akkermansia sp. CAG:344]
WP_011367236.1 191 rubrerythrin [Desulfovibrio alaskensis]
CDN30853.1 191 Rubrerythrin [Mucinivorans hirudinis]
WP_040909165.1 191 rubrerythrin [Succinatimonas hippei]
WP_012750412.1 191 rubrerythrin [Desulfovibrio magneticus]
CDA21257.1 191 rubrerythrin [Bacteroides sp. CAG:144]
WP_027181940.1 191 rubrerythrin [Desulfovibrio alaskensis]
CDD90020.1 191 putative uncharacterized protein [Tannerella sp. CAG:51]
WP_031451152.1 191 rubrerythrin [Desulfobacula sp. TS]
WP_013277969.1 191 rubrerythrin [Acetohalobium arabaticum]
WP_015403748.1 191 rubrerythrin [Desulfocapsa sulfexigens]
WP_041585684.1 191 rubrerythrin [Syntrophus aciditrophicus]
CCZ21913.1 191 rubrerythrin [Acetobacter sp. CAG:977]
WP_027365788.1 191 rubrerythrin [Desulfotomaculum alcoholivorax]
GAP68371.1 191 rubrerythrin [Bacteroidales bacterium 6E]
WP_024823535.1 191 rubrerythrin [Desulfovibrio magneticus]
WP_027357987.1 191 rubrerythrin [Desulforegula conservatrix]
WP_027720491.1 191 rubrerythrin [Desulfovibrio zosterae]
WP_044348917.1 191 rubrerythrin [Desulfarculus sp. SPR]
WP_007526116.1 191 rubrerythrin [Desulfovibrio sp. A2]
ESQ12708.1 191 hypothetical protein N839_07900 [uncultured Desulfofustis sp. PB-SRB1]
WP_028323298.1 191 rubrerythrin [Desulfatirhabdium butyrativorans]
WP_054033821.1 191 rubrerythrin [Desulfatitalea tepidiphila]
WP_022637374.1 191 rubrerythrin [Chitinivibrio alkaliphilus]
WP_024269093.1 191 rubrerythrin [Salinispira pacifica]
WP_010940353.1 191 rubrerythrin [Desulfovibrio vulgaris]
WP_011187439.1 191 rubrerythrin [Desulfotalea psychrophila]
WP_037546401.1 191 rubrerythrin [Spirochaeta sp. JC230]
WP_013446093.1 191 rubrerythrin [Paludibacter propionicigenes]
WP_027185132.1 191 rubrerythrin [Desulfovibrio inopinatus]
WP_043631798.1 191 rubrerythrin [Desulfovibrio sp. TomC]
WP_027369402.1 191 rubrerythrin [Desulfovermiculus halophilus]
KPK26950.1 191 rubrerythrin [Desulfobacterales bacterium SG8_35_2]
WP_015335533.1 191 rubrerythrin [Desulfovibrio hydrothermalis]
WP_008685449.1 191 MULTISPECIES: rubrerythrin [Desulfovibrio]
CCX94114.1 191 putative uncharacterized protein [Bacteroides sp. CAG:20]
WP_028584150.1 191 rubrerythrin [Desulfobulbus mediterraneus]
WP_015946000.1 191 rubrerythrin [Desulfovibrio vulgaris]
WP_012176028.1 191 rubrerythrin [Desulfococcus oleovorans]
EJZ61940.1 191 hypothetical protein HMPREF9448_02617 [Barnesiella intestinihominis YIT 11860]
WP_031386421.1 191 rubrerythrin [Desulfonatronum thiodismutans]

WP_031481624.1 191   rubrerythrin [Desulfovibrio frigidus]
WP_035068052.1 191   rubrerythrin [Desulfovibrio termitidis]
WP_011526779.1 191   rubrerythrin [Lawsonia intracellularis]
WP_029459530.1 191   rubrerythrin [Desulfovibrio alcoholivorans]
WP_004514275.1 191   rubrerythrin [Geobacter metallireducens]
WP_028573294.1 191   rubrerythrin [Desulfonatronum lacustre]
WP_028587657.1 191   rubrerythrin [Desulfocurvus vexinensis]
WP_015402607.1 191   rubrerythrin [Desulfocapsa sulfexigens]
CDA21674.1   191   putative uncharacterized protein [Bacteroides sp.CAG:144]
WP_046737350.1 191   rubrerythrin [Dehalogenimonas sp. WBC-2]
WP_037561765.1 191   rubrerythrin [Spirochaeta sp. JC202]
WP_009181658.1 191   rubrerythrin [Desulfovibrio sp. FW1012B]
WP_054029823.1 191   rubrerythrin [Desulfatitalea tepidiphila]
WP_006006079.1 191   rubrerythrin [Desulfovibrio piger]
WP_028877737.1 191   rubrerythrin [Terasakiella pusilla]
WP_005990785.1 191   rubrerythrin [Desulfovibrio fructosivorans]
WP_009105908.1 191   rubrerythrin [Desulfovibrio sp. U5L]
WP_006966864.1 191   MULTISPECIES: rubrerythrin [Desulfotignum]
WP_027291782.1 191   rubrerythrin [Rikenella microfusus]
WP_008870214.1 191   rubrerythrin [Desulfonatronospira thiodismutans]
WP_031579836.1 191   rubrerythrin [Ruminobacter sp. RM87]
WP_013253211.1 191   rubrerythrin [Spirochaeta smaragdinae]
WP_012176061.1 191   rubrerythrin [Desulfococcus oleovorans]
EKD65848.1   191   rubrerythrin [uncultured bacterium (gcode 4)]
WP_027192012.1 191   rubrerythrin [Desulfovibrio putealis]
KPK25014.1   191   rubrerythrin [Dehalococcoidia bacterium SG8_51_3]
XP_001320125.1 191   rubrerythrin [Trichomonas vaginalis G3]
WP_020611480.1 191   rubrerythrin [Spirochaeta bajacaliforniensis]
WP_012624269.1 191   rubrerythrin [Desulfovibrio desulfuricans]
WP_052644722.1 191   rubrerythrin [haloalkaliphilic bacterium ACht6-1]
KGO35505.1   191   rubrerythrin [Desulfobulbus sp. Tol-SR]
WP_028895282.1 191   rubrerythrin [Syntrophorhabdus aromaticivorans]
WP_022659514.1 191   rubrerythrin [Desulfovibrio desulfuricans]
WP_045221761.1 191   rubrerythrin [Desulfonatronum thioautotrophicum]
WP_014162393.1 191   rubrerythrin [Thermovirga lienii]
WP_011700050.1 191   rubrerythrin [Syntrophobacter fumaroxidans]
WP_028315440.1 191   rubrerythrin [Desulfatibacillum aliphaticivorans]
WP_015948579.1 191   rubrerythrin [Desulfatibacillum alkenivorans]
WP_006967833.1 191   rubrerythrin [Desulfotignum phosphitoxidans]
WP_022062916.1 191   rubrerythrin [Alistipes inops]
WP_024333274.1 191   rubrerythrin [Desulfotignum balticum]
WP_012662982.1 191   rubrerythrin [Desulfobacterium autotrophicum]
WP_013164260.1 191   rubrerythrin [Desulfurivibrio alkaliphilus]

ESQ09050.1 191 hypothetical protein N839_14080 [uncultured Desulfofustis sp. PB-SRB1]
KJR97003.1 191 rubrerythrin [Desulfobulbaceae bacterium BRH_c16a]
WP_015326477.1 191 rubrerythrin [Halobacteroides halobius]
KKK45799.1 191 Rubrerythrin-2 [Lokiarchaeum sp. GC14_75]
KQC13697.1 191 rubrerythrin [Methanosaeta sp. SDB]
WP_018247234.1 191 rubrerythrin [Orenia marismortui]
WP_022664902.1 191 rubrerythrin [Desulfospira joergensenii]
WP_054696138.1 191 rubrerythrin [Desulfosarcina cetonica]
WP_014955780.1 191 rubrerythrin [Desulfobacula toluolica]
WP_028972836.1 191 rubrerythrin [Spirochaeta cellobiosiphila]
KKT53703.1 191 rubrerythrin [Parcubacteria bacterium GW2011_GWC2_44_22]
CCX45138.1 191 putative uncharacterized protein [Prevotella sp.CAG:1031]
WP_047446982.1 191 rubrerythrin [Alistipes sp. ZOR0009]
WP_027340637.1 191 rubrerythrin [Halonatronum saccharophilum]
WP_005028319.1 191 MULTISPECIES: rubrerythrin [Bilophila]
WP_021931732.1 191 hypothetical protein [Coprobacter secundus]
KKK40908.1 191 Rubrerythrin-2 [Lokiarchaeum sp. GC14_75]
WP_051564869.1 191 hypothetical protein [Acidobacteriaceae bacterium URHE0068]
WP_044036735.1 191 rubrerythrin [Clostridium bornimense]
EKE29180.1 191 rubrerythrin [uncultured bacterium (gcode 4)]
WP_031450116.1 191 rubrerythrin [Desulfobacula sp. TS]
WP_027870716.1 191 reverse rubrerythrin-1 [[Eubacterium] cellulosolvens]
WP_004603579.1 191 rubrerythrin [[Eubacterium] cellulosolvens]
GAP44537.1 191 Rubrerythrin [Bacteroidales bacterium TBC1]
EIC01069.1 191 Rubrerythrin [Treponema saccharophilum DSM 2985]
CDE60057.1 191 rubrerythrin [Fusobacterium sp. CAG:439]
WP_033162550.1 191 reverse rubrerythrin-1 [Sharpea azabuensis]
EFY05962.1 191 rubredoxin [Phascolarctobacterium succinatutens YIT 12067]
CEP00741.1 191 hypothetical protein PBRA_001795 [Plasmodiophora brassicae]
AFL65981.1 190 Rubrerythrin [Desulfurococcus fermentans DSM 16532]
WP_054835868.1 190 rubrerythrin [Methanobrevibacter arboriphilus]
WP_042704224.1 190 rubrerythrin [Methanobrevibacter arboriphilus]
CDE85206.1 190 rubrerythrin [Coraliomargarita sp. CAG:312]
KPJ66132.1 190 rubrerythrin [Coxiella sp. DG_40]
KPK67860.1 190 rubrerythrin [candidate division TA06 bacterium SM23_40]
KPL19145.1 190 rubrerythrin [candidate division Zixibacteria bacterium SM23_81]
WP_013755819.1 190 rubrerythrin [Thermodesulfobium narugense]
WP_013683737.1 190 rubrerythrin [Archaeoglobus veneficus]

WP_010878334.1 190 rubrerythrin [Archaeoglobus fulgidus]
WP_048095334.1 190 rubrerythrin [Archaeoglobus fulgidus]
WP_041971718.1 190 rubrerythrin [Geobacter sp. OR-1]
CDE45142.1 190 putative uncharacterized protein [Clostridium sp.CAG:768]
AGI85811.1 190 Rubrerythrin [Candidatus Methanomethylophilus alvus Mx1201]
WP_020676503.1 190 rubrerythrin [Geopsychrobacter electrodiphilus]
WP_011448328.1 190 rubrerythrin [Methanospirillum hungatei]
KPK41364.1 190 rubrerythrin [Phycisphaerae bacterium SG8_4]
KPK35817.1 190 rubrerythrin [Phycisphaerae bacterium SG8_4]
KQC04006.1 190 rubrerythrin [Methanoculleus sp. SDB]
ADU61823.1 190 Rubrerythrin [Desulfovibrio aespoeensis Aspo-2]
WP_038470795.1 190 rubrerythrin [Mollicutes bacterium HR1]
WP_015284420.1 190 rubrerythrin [Methanoregula formicica]
WP_011342107.1 190 rubrerythrin [Pelobacter carbinolicus]
WP_048093937.1 190 rubrerythrin [Candidatus Methanoperedens nitroreducens]
WP_014322391.1 190 rubrerythrin [Desulfovibrio desulfuricans]
WP_035055537.1 190 rubrerythrin [Desulfuromonas sp. TF]
WP_006423105.1 190 rubrerythrin [delta proteobacterium NaphS2]
KKQ10940.1 190 Rubrerythrin [candidate division WS6 bacterium GW2011_GWC2_36_7]
WP_022854187.1 190 rubrerythrin [Thermodesulfatator atlanticus]
WP_053549946.1 190 rubrerythrin [Desulfuromonas sp. WTL]
WP_039646436.1 190 rubrerythrin [Geobacter soli]
KON33521.1 190 rubrerythrin [miscellaneous Crenarchaeota group-6 archaeon AD8-1]
KPJ60919.1 190 rubrerythrin [Latescibacteria bacterium DG_63]
WP_022661277.1 190 rubrerythrin [Desulfovibrio longus]
WP_010943440.1 190 rubrerythrin [Geobacter sulfurreducens]
WP_027175670.1 190 rubrerythrin [Desulfovibrio aminophilus]
KPK74746.1 190 rubrerythrin [Phycisphaerae bacterium SM23_30]
WP_018336532.1 190 rubrerythrin [Butyricimonas synergistica]
KKP92913.1 190 Rubrerythrin [candidate division WS6 bacterium GW2011_GWC1_36_11]
WP_013611958.1 190 rubrerythrin [Odoribacter splanchnicus]
CDB08865.1 190 rubrerythrin [Odoribacter splanchnicus CAG:14]
WP_011939152.1 190 rubrerythrin [Geobacter uraniireducens]
WP_027201120.1 190 rubrerythrin [Butyricimonas virosa]
WP_012940032.1 190 rubrerythrin [Archaeoglobus profundus]
WP_041721174.1 190 Rubrerythrin-2 [Alkaliphilus metalliredigens]
CCZ09086.1 190 rubrerythrin [Odoribacter sp. CAG:788]
CCZ51085.1 190 rubrerythrin [Acinetobacter sp. CAG:196]
WP_015758189.1 190 rubrerythrin [Desulfotomaculum acetoxidans]
WP_015414944.1 190 rubrerythrin [Desulfovibrio piezophilus]
1RYT 190 "Chain A, Rubrerythrin"
KFZ27103.1 190 Rubrerythrin [Mollicutes bacterium HR2]

WP_009136958.1 190   rubrerythrin [Odoribacter laneus]
KPK62857.1   190   rubrerythrin [Planctomycetes bacterium
SM23_32]
WP_028854998.1 190   rubrerythrin [Psychrilyobacter atlanticus]
EHO63412.1   190   hypothetical protein HMPREF9453_00429
[Dialister succinatiphilus YIT 11850]
WP_014552427.1 190   rubrerythrin [Halanaerobium praevalens]
CCY24521.1   190   putative uncharacterized protein [Brachyspira
sp.CAG:484]
WP_050637578.1 190   rubrerythrin [Candidatus Stoquefichus sp. SB1]
WP_049755933.1 190   rubrerythrin [Desulfotomaculum reducens]
WP_027188758.1 190   rubrerythrin [Desulfovibrio cuneatus]
WP_041503793.1 190   rubrerythrin [Sanguibacteroides justesenii]
WP_039743409.1 190   rubrerythrin [Geobacter pickeringii]
AGL02745.1   190   rubrerythrin [Desulfotomaculum gibsoniae DSM
7213]
WP_013908170.1 190   rubrerythrin [Thermodesulfatator indicus]
WP_048193200.1 190   rubrerythrin [Methanococcoides methylutens]
WP_048204774.1 190   rubrerythrin [Methanococcoides methylutens]
CDA49315.1   190   putative uncharacterized protein [Dialister
sp. CAG:486]
WP_012107850.1 190   rubrerythrin [Methanoregula boonei]
WP_023937807.1 190   rubrerythrin [Porphyromonas crevioricanis]
CCZ54726.1   190   rubrerythrin [Dialister invisus CAG:218]
EEW96766.1   190   Rubrerythrin [Dialister invisus DSM 15470]
KGO34590.1   190   rubrerythrin [Desulfobulbus sp. Tol-SR]
KKP69391.1   190   Rubrerythrin [candidate division CPR3
bacterium GW2011_GWF2_35_18]
WP_006873306.1 190   rubrerythrin [Anaerotruncus colihominis]
WP_055245195.1 190   rubrerythrin [Anaerotruncus colihominis]
WP_021875007.1 190   Rubredoxin/rubrerythrin [Clostridium chauvoei]
WP_021875005.1 190   Rubredoxin/rubrerythrin [Clostridium chauvoei]
CCX66113.1   190   putative uncharacterized protein [Firmicutes
bacterium CAG:791]
WP_042684508.1 189   rubrerythrin [Methermicoccus shengliensis]
WP_013799585.1 189   rubrerythrin [Methanotorris igneus]
WP_004591527.1 189   rubrerythrin [Methanocaldococcus villosus]
WP_018154272.1 189   rubrerythrin [Methanothermococcus
thermolithotrophicus]
EGC92084.1   189   rubrerythrin [Turicibacter sp. HGF1]
EFF64972.1   189   rubrerythrin [Turicibacter sanguinis PC909]
CUM95984.1   189   NADH peroxidase [Turicibacter sanguinis]
WP_011406382.1 189   rubrerythrin [Methanosphaera stadtmanae]
XP_004185684.1 189   hypothetical protein EIN_296480 [Entamoeba
invadens IP1]
XP_001734442.1 189   hypothetical protein [Entamoeba dispar SAW760]
XP_652131.1   189   rubrerythrin [Entamoeba histolytica HM-1:IMSS]
WP_049750090.1 189   rubrerythrin [Syntrophomonas wolfei]
EDS12607.1   189   Rubrerythrin [Anaerotruncus colihominis DSM
17241]

EWS56209.1	189	NADH peroxidase [Methylibium sp. T29]
WP_034439356.1	189	rubrerythrin [Candidatus Clostridium anorexicamassiliense]
WP_040659440.1	189	rubrerythrin [Oscillibacter ruminantium]
EHI60849.1	189	"hypothetical protein HMPREF9473_01226, partial[[Clostridium] hathewayi WAL-18680], partial [Hungatella hathewayi WAL-18680]"
WP_052647262.1	189	"hypothetical protein, partial [Parabacteroides distasonis]"
CDA80893.1	189	putative uncharacterized protein [Firmicutes bacterium CAG:176]
CCX74805.1	189	putative uncharacterized protein [Firmicutes bacterium CAG:83]
WP_014115985.1	189	hypothetical protein [Oscillibacter valericigenes]
WP_040660762.1	189	reverse rubrerythrin-1 [Oscillibacter ruminantium]
WP_003866202.1	189	hypothetical protein [Holdemanella biformis]
CDA34599.1	189	putative uncharacterized protein [Firmicutes bacterium CAG:536]
CRH84074.1	189	NADH peroxidase [Chlamydia trachomatis]
CCX72147.1	189	putative uncharacterized protein [Firmicutes bacterium CAG:555]
WP_023438056.1	188	rubrerythrin [Clostridium tetani]
WP_011099417.1	188	rubrerythrin [Clostridium tetani]
WP_035768032.1	188	rubrerythrin [Butyrivibrio sp. NC2002]
ESQ26743.1	188	Rubrerythrin [uncultured Acidilobus sp. JCHS]
WP_029320843.1	188	rubrerythrin [Butyrivibrio sp. AE3004]
WP_022760481.1	188	MULTISPECIES: rubrerythrin [Butyrivibrio]
WP_026495011.1	188	MULTISPECIES: rubrerythrin [Butyrivibrio]
AFZ71258.1	188	rubrerythrin [Caldisphaera lagunensis DSM 15908]
WP_012618309.1	188	rubrerythrin [Methanosphaerula palustris]
WP_010878335.1	188	rubrerythrin [Archaeoglobus fulgidus]
EEA83634.1	188	Rubrerythrin [Tyzzerella nexilis DSM 1787]
WP_055275627.1	188	rubrerythrin [Turicibacter sanguinis]
WP_035773736.1	188	rubrerythrin [Butyrivibrio sp. VCD2006]
WP_055244722.1	188	rubrerythrin [Turicibacter sanguinis]
WP_055304873.1	188	rubrerythrin [Turicibacter sanguinis]
ERL05449.1	188	rubredoxin [Mitsuokella sp. oral taxon 131 str. W9106]
KKR31355.1	188	Rubrerythrin [Parcubacteria (Falkowbacteria) bacterium GW2011_GWF2_39_8]
KJS29564.1	188	rubrerythrin [Desulfatitalea sp. BRH_c12]
WP_012940031.1	188	rubrerythrin [Archaeoglobus profundus]
WP_039630034.1	188	rubrerythrin [Clostridium argentinense]
ACU40891.1	188	rubrerythrin [Clostridium perfringens SM101]
CDC73778.1	188	putative uncharacterized protein [Oscillibacter sp.CAG:155]
EEU96748.1	188	rubredoxin [Faecalibacterium prausnitzii

A2-165]
WP_021751391.1 188   MULTISPECIES: rubredoxin [Oscillibacter]
CDB44589.1   188   putative uncharacterized protein [Firmicutes bacterium CAG:240]
EFG95225.1   187   rubredoxin [Fusobacterium nucleatum subsp. nucleatum ATCC 23726]
WP_027110477.1 187   rubrerythrin [Lachnospiraceae bacterium NC2008]
WP_027111240.1 187   rubrerythrin [Lachnospiraceae bacterium NK4A136]
WP_023353752.1 187   rubredoxin [Catonella morbi]
WP_031549026.1 187   rubrerythrin [Oribacterium sp. FC2011]
WP_044913568.1 187   rubrerythrin [Butyrivibrio sp. WCE2006]
WP_046496118.1 187   rubrerythrin [Syntrophomonas zehnderi]
WP_026509644.1 187   rubrerythrin [Butyrivibrio sp. LC3010]
WP_026658409.1 187   rubrerythrin [Butyrivibrio sp. AC2005]
WP_029200279.1 187   rubrerythrin [Oribacterium sp. NK2B42]
WP_022778708.1 187   rubrerythrin [Butyrivibrio sp. AE3009]
WP_026488607.1 187   rubrerythrin [Butyrivibrio sp. XBB1001]
WP_026662055.1 187   rubrerythrin [Butyrivibrio proteoclasticus]
WP_022772842.1 187   rubrerythrin [Butyrivibrio sp. AE2015]
WP_026653756.1 187   rubrerythrin [Butyrivibrio proteoclasticus]
WP_029232359.1 187   rubrerythrin [Butyrivibrio sp. VCB2006]
WP_013279767.1 187   rubrerythrin [Butyrivibrio proteoclasticus]
EFW24763.1   187   Rubrerythrin [Solobacterium moorei F0204]
WP_012281292.1 187   rubrerythrin [Heliobacterium modesticaldum]
WP_026493443.1 187   MULTISPECIES: rubrerythrin [Butyrivibrio]
WP_019133211.1 187   hypothetical protein [Peptoniphilus obesi]
EPZ61933.1   187   rubrerythrin-1 [[[Clostridium] sordellii ATCC 9714]
WP_044942626.1 187   rubrerythrin [Flavonifractor plautii]
WP_007490931.1 187   MULTISPECIES: rubrerythrin [Clostridiales]
WP_009261316.1 187   rubrerythrin [Flavonifractor plautii]
CCY01957.1   187   rubredoxin/rubrerythrin [Prevotella sp. CAG:924]
AIF26914.1   187   hypothetical protein [uncultured bacterium fosmid pJB154B8_contig II]
WP_044995284.1 187   reverse rubrerythrin-1 [[Clostridium] scindens]
WP_004340983.1 187   MULTISPECIES: rubrerythrin [Prevotella]
KON46559.1   187   rubrerythrin [Mariprofundus ferrooxydans]
WP_027444999.1 187   rubrerythrin [Prevotella baroniae]
WP_021589504.1 187   rubrerythrin domain protein [Prevotella baroniae]
EEC58371.1   187   rubredoxin [[Bacteroides] pectinophilus ATCC 43243]
ADM28143.1   186   Rubrerythrin [Ignisphaera aggregans DSM 17230]
WP_012309164.1 186   rubrerythrin [Candidatus Korarchaeum cryptofilum]
WP_011838347.1 186   rubrerythrin [Staphylothermus marinus]

WP_012159692.1 186   rubrerythrin [Alkaliphilus oremlandii]
WP_013130119.1 186   rubrerythrin [Thermosphaera aggregans]
WP_014256015.1 186   rubrerythrin [[Clostridium] clariflavum]
WP_055408571.1 186   Rubrerythrin-2 [Pyrodictium delaneyi]
WP_042666848.1 186   Rubrerythrin-2 [Desulfurococcus amylolyticus]
WP_044824245.1 186   Rubrerythrin-2 [Clostridium aceticum]
WP_048815811.1 186   Rubrerythrin-2 [Desulfurococcus fermentans]
WP_041606071.1 186   Rubrerythrin-2 [Halothermothrix orenii]
WP_012065401.1 186   rubrerythrin [Alkaliphilus metalliredigens]
WP_027116448.1 186   rubrerythrin [Lachnospiraceae bacterium P6B14]
WP_006599316.1 186   rubrerythrin [Pseudoramibacter alactolyticus]
WP_009104226.1 186   rubrerythrin [Treponema sp. JC4]
WP_027728770.1 186   rubrerythrin [Treponema sp. C6A8]
EGQ74171.1   186   rubrerythrin [Fusobacterium nucleatum subsp.
animalis ATCC 51191]
WP_004097601.1 186   rubrerythrin [Acetonema longum]
ESE31097.1   186   rubrerythrin [Eubacterium brachy ATCC 33089]
WP_022933159.1 186   rubrerythrin [Treponema bryantii]
KKQ31386.1   186   rubrerythrin [Microgenomates (Shapirobacteria)
bacterium GW2011_GWF2_37_20]
CDA56662.1   186   rubredoxin/rubrerythrin [Prevotella sp. CAG:
604]
CDD20342.1   186   rubredoxin/rubrerythrin [Prevotella sp. CAG:
732]
EEU51305.1   186   rubredoxin [Parabacteroides sp. D13]
WP_049728516.1 186   hypothetical protein [Dorea sp. D27]
WP_009141576.1 186   rubrerythrin [Collinsella tanakaei]
WP_008021780.1 186   rubrerythrin [Bacteroides xylanisolvens]
WP_027326221.1 186   rubrerythrin [Bacteroides pyogenes]
WP_004297333.1 186   MULTISPECIES: rubrerythrin [Bacteroides]
WP_004315957.1 186   MULTISPECIES: rubrerythrin [Bacteroides]
WP_010537504.1 186   MULTISPECIES: rubrerythrin [Bacteroides]
WP_040360524.1 186   hypothetical protein [Collinsella stercoris]
WP_044161048.1 186   rubrerythrin [Bacteroides reticulotermitis]
WP_008760531.1 186   MULTISPECIES: rubrerythrin [Bacteroides]
WP_039910730.1 186   hypothetical protein [Collinsella
intestinalis]
WP_027449708.1 186   rubrerythrin [Prevotella brevis]
WP_027452720.1 186   rubrerythrin [Prevotella albensis]
CCZ11705.1   186   rubredoxin [Prevotella sp. CAG:1092]
WP_007902767.1 186   rubrerythrin [Prevotella stercorea]
CDB04522.1   186   rubredoxin [Prevotella sp. CAG:520]
CDC27299.1   186   rubredoxin/rubrerythrin [Prevotella sp. CAG:
386]
CDA65509.1   186   rubredoxin/rubrerythrin [Prevotella copri CAG:
164]
WP_006846830.1 186   rubrerythrin [Prevotella copri]
CDA94435.1   186   rubrerythrin [Prevotella sp. CAG:1320]
CDD77519.1   186   putative uncharacterized protein
[Cryptobacterium sp.CAG:338]

WP_019239019.1	186	hypothetical protein [Collinsella sp. GD3]
CCY06130.1	186	putative uncharacterized protein [Eggerthella sp.CAG:1427]
WP_019129086.1	186	hypothetical protein [Enorma massiliensis]
CCY36432.1	186	rubrerythrin [Alistipes sp. CAG:831]
WP_022094328.1	186	MULTISPECIES: rubredoxin [Collinsella]
EDS75528.1	186	rubredoxin [[[Clostridium] spiroforme DSM 1552]
WP_011753266.1	185	rubrerythrin [Thermofilum pendens]
WP_026477307.1	185	Rubrerythrin-2 [Alkaliphilus transvaalensis]
WP_034445569.1	185	rubrerythrin [Butyrivibrio sp. AE2032]
WP_026514505.1	185	rubrerythrin [Butyrivibrio sp. LB2008]
ACL70656.1	185	Rubrerythrin [Halothermothrix orenii H 168]
WP_022770355.1	185	MULTISPECIES: rubrerythrin [Butyrivibrio]
WP_018703050.1	185	MULTISPECIES: hypothetical protein [Veillonellaceae]
WP_022765800.1	185	rubrerythrin [Butyrivibrio sp. XPD2006]
WP_026516526.1	185	rubrerythrin [Butyrivibrio sp. MC2021]
WP_024865436.1	185	rubrerythrin [Butyrivibrio sp. FCS014]
WP_026668081.1	185	MULTISPECIES: rubrerythrin [Butyrivibrio]
WP_026656696.1	185	rubrerythrin [Butyrivibrio sp. AE3003]
WP_009009477.1	185	"hypothetical protein, partial [Coprobacillus sp. D7]"
WP_004602127.1	185	rubrerythrin [[Eubacterium] cellulosolvens]
EEG35028.1	185	Rubrerythrin [[[Eubacterium] hallii DSM 3353]
WP_041719340.1	185	Rubrerythrin-2 [Alkaliphilus oremlandii]
WP_034327790.1	185	Rubrerythrin-2 [Alkaliphilus transvaalensis]
WP_005490414.1	185	rubrerythrin [Halanaerobium saccharolyticum]
EFE27957.2	185	rubredoxin [Filifactor alocis ATCC 35896]
KKP97689.1	185	Rubrerythrin [Parcubacteria bacterium GW2011_GWC2_36_17]
WP_035658380.1	185	rubrerythrin [Lachnospiraceae bacterium AC2031]
WP_055258443.1	185	rubrerythrin [Sarcina ventriculi]
AKM82576.1	185	rubrerythrin [Berkelbacteria bacterium GW2011_GWE1_39_12]
KKS39928.1	185	Rubrerythrin [Parcubacteria (Kuenenbacteria) bacterium GW2011_GWA2_42_15]
EKD23022.1	185	rubrerythrin [uncultured bacterium]
WP_054347887.1	185	rubrerythrin [Clostridia bacterium UC5.1-1D2]
WP_009532810.1	185	hypothetical protein [Stomatobaculum longum]
CDN32110.1	185	Rubrerythrin [Mucinivorans hirudinis]
WP_028903793.1	185	MULTISPECIES: rubrerythrin [Prevotella]
WP_013064684.1	185	rubrerythrin [Prevotella ruminicola]
WP_013251201.1	185	rubrerythrin [Olsenella uli]
WP_033150891.1	185	rubrerythrin [Prevotella sp. RM4]
CDB71723.1	185	reverse rubrerythrin-1 [Bacteroides cellulosilyticus CAG:158]
WP_007215010.1	185	MULTISPECIES: rubrerythrin [Bacteroides]
CCX52675.1	185	rubredoxin/rubrerythrin domain protein

[Alistipes sp.CAG:514]
WP_036912480.1 185   rubrerythrin [Prevotella sp. FD3004]
WP_042368037.1 185   rubrerythrin [Bacteroidaceae bacterium MS4]
WP_005677773.1 185   rubrerythrin [Bacteroides caccae]
WP_005654904.1 185   rubrerythrin [Bacteroides stercoris]
WP_007759170.1 185   MULTISPECIES: rubrerythrin [Bacteroides]
CCX60638.1   185   rubredoxin [Bacteroides sp. CAG:598]
WP_007754478.1 185   rubrerythrin [Bacteroides finegoldii]
WP_005836026.1 185   MULTISPECIES: rubrerythrin [Bacteria]
WP_057281797.1 185   rubrerythrin [Bacteroides uniformis]
WP_005826622.1 185   MULTISPECIES: rubrerythrin [Bacteroides]
WP_009123967.1 185   rubrerythrin [Bacteroides fluxus]
WP_002561768.1 185   MULTISPECIES: rubrerythrin [Bacteroides]
WP_018668172.1 185   rubrerythrin [Bacteroides gallinarum]
WP_004289806.1 185   rubrerythrin [Bacteroides eggerthii]
WP_009120582.1 185   rubrerythrin [Bacteroides clarus]
WP_009131168.1 185   MULTISPECIES: reverse rubrerythrin-1
[Bacteroides]
WP_025074650.1 185   rubrerythrin [Bacteroides faecichinchillae]
WP_005923828.1 185   rubrerythrin [Bacteroides salyersiae]
WP_028913103.1 185   rubrerythrin [Prevotella sp. MA2016]
WP_024996773.1 185   rubrerythrin [Bacteroides graminisolvens]
WP_005802932.1 185   MULTISPECIES: rubrerythrin [Bacteroides]
WP_005780311.1 185   MULTISPECIES: rubrerythrin [Bacteroides]
WP_014546175.1 185   rubredoxin domain/rubrerythrin domain
protein[Fibrobacter succinogenes]
WP_006744184.1 185   rubrerythrin [Bacteroides coprosuis]
CCZ15422.1   185   rubredoxin [Prevotella sp. CAG:487]
WP_028910195.1 185   rubrerythrin [Prevotella sp. AGR2160]
WP_005883686.1 185   hypothetical protein [Fusobacterium
mortiferum]
CDD00247.1   185   rubredoxin/rubrerythrin [Prevotella sp. CAG:
474]
WP_018464624.1 185   rubrerythrin [Prevotella paludivivens]
WP_040221130.1 185   hypothetical protein [Collinsella sp. MS5]
WP_018108963.1 185   rubrerythrin [Bacteroides propionicifaciens]
WP_006281395.1 185   rubrerythrin [Prevotella bryantii]
CDD66986.1   185   putative uncharacterized protein [Eggerthella
sp.CAG:368]
WP_004376384.1 185   rubrerythrin [Prevotella oris]
WP_026285948.1 185   rubrerythrin [Prevotella oris]
WP_010966300.1 184   Rubrerythrin-2 [Clostridium acetobutylicum]
WP_013404091.1 184   rubrerythrin [Caldicellulosiruptor
hydrothermalis]
WP_003515904.1 184   rubrerythrin [Ruminiclostridium thermocellum]
WP_013431128.1 184   MULTISPECIES: rubrerythrin
[Caldicellulosiruptor]
WP_004623811.1 184   rubrerythrin [[Clostridium] termitidis]
CUH93674.1   184   Rubrerythrin-2 [Herbinix sp. SD1D]
WP_045173788.1 184   Rubrerythrin-2 [Caldicellulosiruptor sp.

		Wai35.B1]
WP_008675976.1	184	rubrerythrin [Clostridium sp. 7_2_43FAA]
WP_027630854.1	184	Rubrerythrin-2 [[Clostridium] cellobioparum]
WP_013239243.1	184	MULTISPECIES: rubrerythrin [Clostridium]
WP_038322710.1	184	Rubrerythrin-2 [bacterium MS4]
WP_038290335.1	184	Rubrerythrin-2 [[Clostridium] straminisolvens]
WP_021653591.1	184	rubredoxin [Clostridiales bacterium oral taxon 876]
WP_035152429.1	184	Rubrerythrin-2 [Clostridium tetanomorphum]
WP_016207820.1	184	MULTISPECIES: rubrerythrin [Clostridium]
WP_045168441.1	184	Rubrerythrin-2 [Caldicellulosiruptor sp. Rt8.B8]
CRZ33232.1	184	Rubrerythrin-2 [Herbinix hemicellulosilytica]
WP_011916036.1	184	rubrerythrin [Caldicellulosiruptor saccharolyticus]
WP_035309429.1	184	Rubrerythrin-2 [Clostridium sp. HMP27]
WP_010073584.1	184	rubrerythrin [Clostridium cellulovorans]
WP_026881658.1	184	Rubrerythrin-2 [Clostridium akagii]
WP_014805963.1	184	rubrerythrin [Anaerobaculum mobile]
WP_003446704.1	184	rubrerythrin [Clostridium pasteurianum]
WP_008518515.1	184	rubrerythrin [Dethiobacter alkaliphilus]
WP_013048395.1	184	rubrerythrin [Aminobacterium colombiense]
WP_012201128.1	184	rubrerythrin [Lachnoclostridium phytofermentans]
WP_013276547.1	184	rubrerythrin [Thermosediminibacter oceani]
WP_054874105.1	184	Rubrerythrin-2 [Oxobacter pfennigii]
AFH42831.1	184	Rubrerythrin [Fervidicoccus fontis Kam940]
WP_010239140.1	184	rubrerythrin [Clostridium arbusti]
WP_021804113.1	184	rubrerythrin [Clostridium intestinale]
WP_015616990.1	184	rubrerythrin [Clostridium pasteurianum]
WP_017895636.1	184	rubrerythrin [Clostridium tyrobutyricum]
WP_027423162.1	184	rubrerythrin [Lachnospiraceae bacterium AC3007]
WP_017751338.1	184	rubrerythrin [Clostridium tyrobutyricum]
WP_014119866.1	184	rubrerythrin [Oscillibacter valericigenes]
WP_040664245.1	184	Rubrerythrin-2 [Oscillibacter ruminantium]
WP_009201996.1	184	rubrerythrin [Anaerobaculum hydrogeniformans]
WP_008522232.1	184	MULTISPECIES: rubrerythrin [Jonquetella]
WP_033167689.1	184	Rubrerythrin-2 [Clostridium sp. KNHs205]
WP_029503652.1	184	Rubrerythrin-2 [Lachnoclostridium phytofermentans]
CCY46459.1	184	rubrerythrin [Firmicutes bacterium CAG:822]
WP_019678004.1	184	rubrerythrin [Ruminococcus flavefaciens]
CDB35436.1	184	rubrerythrin [Phascolarctobacterium sp. CAG: 266]
CDC42288.1	184	rubrerythrin [Firmicutes bacterium CAG:449]
EDP11788.1	184	Rubrerythrin [[[Eubacterium] dolichum DSM 3991]
WP_011523538.1	184	rubrerythrin [Candidatus Koribacter versatilis]

WP_040910258.1	184	rubrerythrin [Subdoligranulum sp. 4_3_54A2FAA]
CDB45409.1	184	rubrerythrin [Phascolarctobacterium sp. CAG:207]
WP_014017857.1	184	rubrerythrin [Candidatus Arthromitus sp. SFB-mouse]
WP_005806942.1	184	MULTISPECIES: rubrerythrin [Candidatus Arthromitus]
WP_014094331.1	184	rubrerythrin [Candidatus Arthromitus sp. SFB-rat-Yit]
WP_006419397.1	184	rubredoxin [delta proteobacterium NaphS2]
CDA21116.1	184	putative uncharacterized protein [Bacteroides sp.CAG:144]
WP_004035663.1	184	hypothetical protein [Clostridium sp. ASF356]
WP_021930436.1	184	reverse rubrerythrin-2 [Porphyromonadaceae bacterium GM3]
WP_044229076.1	184	rubrerythrin [Coprobacter secundus]
WP_010260269.1	184	MULTISPECIES: rubrerythrin [Alistipes]
CCZ97951.1	184	reverse rubrerythrin-2 [Alistipes sp. CAG:157]
WP_027290989.1	184	rubrerythrin [Rikenella microfusus]
WP_031258901.1	184	MULTISPECIES: rubrerythrin [Porphyromonadaceae]
WP_038657626.1	184	rubrerythrin [Mucinivorans hirudinis]
AEB74876.1	184	Rubrerythrin [Clostridium botulinum BKT015925]
WP_035473905.1	184	rubrerythrin [Alistipes inops]
CDE58647.1	184	putative uncharacterized protein [Parabacteroides sp.CAG:409]
CCZ96731.1	184	rubrerythrin [Alistipes sp. CAG:53]
WP_009596576.1	184	MULTISPECIES: rubrerythrin [Alistipes]
CDC99125.1	184	rubrerythrin [Alistipes sp. CAG:268]
WP_025310114.1	184	rubrerythrin [Bacteroidales bacterium CF]
WP_018697267.1	184	rubrerythrin [Alistipes onderdonkii]
WP_019130021.1	184	rubrerythrin [Alistipes obesi]
CDA45640.1	184	rubredoxin [Prevotella sp. CAG:5226]
WP_032134423.1	184	rubrerythrin [Alistipes sp. AL-1]
WP_013548401.1	184	rubrerythrin [Bacteroides helcogenes]
CDB94176.1	184	putative uncharacterized protein [Firmicutes bacterium CAG:41]
CDE63134.1	184	rubrerythrin [Alistipes putredinis CAG:67]
WP_040293562.1	184	rubrerythrin [Alistipes putredinis]
CDE04162.1	184	putative uncharacterized protein [Anaerotruncus sp.CAG:390]
CDB09591.1	184	rubredoxin [Bacteroides sp. CAG:633]
WP_055202973.1	184	rubrerythrin [Alistipes finegoldii]
CCY92490.1	184	rubredoxin [Bacteroides sp. CAG:1076]
WP_007567666.1	184	rubrerythrin [Bacteroides coprocola]
WP_018709987.1	184	rubrerythrin [Bacteroides barnesiae]
CDE07193.1	184	rubrerythrin [Prevotella sp. CAG:485]
WP_025077384.1	184	rubrerythrin [Prevotella fusca]
WP_036877180.1	184	rubrerythrin [Prevotella oryzae]
ACN83568.1	184	rubrerythrin fusion protein [Brachyspira

hyodysenteriae WA1]
CDD04333.1   184   rubredoxin [Prevotella sp. CAG:592]
WP_042519110.1 184   MULTISPECIES: rubrerythrin [Prevotella]
WP_044073929.1 184   rubrerythrin [Prevotella sp. P5-60]
EEG30728.1   184   rubredoxin [[[Clostridium] methylpentosum DSM 5476]
CDD15263.1   184   rubredoxin/rubrerythrin domain protein [Alistipes sp.CAG:435]
WP_007286844.1 184   MULTISPECIES: hypothetical protein [Clostridiales]
CDC61839.1   184   rubrerythrin [Clostridium sp. CAG:448]
WP_018592506.1 184   hypothetical protein [Terrisporobacter glycolicus]
CCY73368.1   184   rubrerythrin [Eubacterium sp. CAG:115]
WP_010168870.1 184   rubrerythrin [Epulopiscium sp. 'N.t. morphotype B']
CCY19454.1   184   putative uncharacterized protein [Eubacterium sp.CAG:786]
WP_052887015.1 183   Rubrerythrin-2 [Thermofilum carboxyditrophus]
WP_020961864.1 183   hypothetical protein [Thermofilum sp. 1910b]
KPQ42122.1   183   rubrerythrin [Candidatus Methanoperedens sp. BLZ1]
WP_025322072.1 183   Rubrerythrin-2 [Deferrisoma camini]
WP_006316469.1 183   rubrerythrin [Caldisalinibacter kiritimatiensis]
WP_052220911.1 183   Rubrerythrin-2 [Clostridium homopropionicum]
WP_035163879.1 183   Rubrerythrin-2 [Caloranaerobacter azorensis]
WP_054870936.1 183   Rubrerythrin-2 [Caloranaerobacter sp. TR13]
KON30053.1   183   hypothetical protein AC482_04865 [miscellaneous Crenarchaeota group-15 archaeon DG-45]
CBL33253.1   183   Rubrerythrin [[[Eubacterium] siraeum V10Sc8a]
KSV60220.1   183   rubrerythrin [Acetivibrio ethanolgignens]
WP_031472891.1 183   rubrerythrin [[Clostridium] aminophilum]
WP_027426529.1 183   rubrerythrin [Lachnospiraceae bacterium NC2004]
EAQ71904.2   183   rubrerythrin [Campylobacter jejuni subsp. jejuni 81-176]
AAW34508.1   183   rubrerythrin [Campylobacter jejuni RM1221]
EOS61828.1   183   hypothetical protein C815_00416 [Firmicutes bacterium M10-2]
BAI62440.1   183   rubrerythrin [Methanocella paludicola SANAE]
WP_027097568.1 183   reverse rubrerythrin-1 [Clostridium paraputrificum]
WP_049753292.1 183   rubrerythrin [Kosmotoga olearia]
WP_049176964.1 183   reverse rubrerythrin-1 [Clostridium botulinum]
WP_027637324.1 183   rubrerythrin [Clostridium cadaveris]
WP_048569485.1 183   reverse rubrerythrin-1 [Clostridium cylindrosporum]
WP_017826780.1 183   rubrerythrin [Clostridium botulinum]
WP_041277804.1 183   reverse rubrerythrin-1 [Desulfotalea

psychrophila]
WP_029911665.1 183   reverse rubrerythrin-1 [Pelobacter selenIigenes]
WP_035379119.1 183   reverse rubrerythrin-1 [Fervidicella metallireducens]
CDE81538.1   183   rubredoxin/rubrerythrin [Ruminococcus sp. CAG:353]
WP_055431978.1 183   rubrerythrin [Candidatus Symbiothrix dinenymphae]
WP_011721187.1 183   MULTISPECIES: rubrerythrin [Clostridium]
WP_007652517.1 183   MULTISPECIES: rubrerythrin [Parabacteroides]
WP_019245200.1 183   rubrerythrin [Candidatus Alistipes marseilloanorexicus]
WP_055202452.1 183   rubrerythrin [Faecalibacterium prausnitzii]
WP_048927455.1 183   rubrerythrin [Parabacteroides sp. D26]
WP_005856013.1 183   MULTISPECIES: rubrerythrin [Bacteroidales]
WP_021282221.1 183   reverse rubrerythrin-1 [Clostridium sp. BL8]
CDE10813.1   183   fAD-dependent pyridine nucleotide-disulfide oxidoreductase [Clostridium sp. CAG:354]
AHF11704.1   183   rubrerythrin [Barnesiella viscericola DSM 18177]
WP_008622918.1 183   MULTISPECIES: rubrerythrin [Paraprevotella]
WP_017413519.1 183   reverse rubrerythrin-1 [Clostridium tunisiense]
WP_041503634.1 183   rubrerythrin [Sanguibacteroides justesenii]
WP_044053933.1 183   rubrerythrin [Alistipes shahii]
CCZ06310.1   183   reverse rubrerythrin-1 [Odoribacter sp. CAG:788]
WP_013611024.1 183   rubrerythrin [Odoribacter splanchnicus]
WP_009136061.1 183   rubrerythrin [Odoribacter laneus]
CDE87858.1   183   putative rubrerythrin [Prevotella sp. CAG:891]
WP_012573190.1 183   rubrerythrin [Candidatus Azobacteroides pseudotrichonymphae]
WP_005640236.1 183   MULTISPECIES: rubrerythrin [Parabacteroides]
CCX48806.1   183   rubrerythrin [Bacteroides sp. CAG:927]
CDE83258.1   183   rubrerythrin [Coraliomargarita sp. CAG:312]
WP_007562386.1 183   MULTISPECIES: rubrerythrin [Bacteroides]
CDD50588.1   183   putative uncharacterized protein [Bacteroides sp.CAG:875]
WP_013617000.1 183   rubrerythrin [Bacteroides salanitronis]
WP_027634469.1 183   reverse rubrerythrin-1 [Clostridium hydrogeniformans]
CCZ71337.1   183   rubrerythrin [Bacteroides sp. CAG:702]
CDE56697.1   183   rubredoxin [Prevotella sp. CAG:873]
WP_031532031.1 183   MULTISPECIES: rubrerythrin [Bacteroides]
WP_005846285.1 183   MULTISPECIES: rubrerythrin [Bacteroides]
WP_024994080.1 183   rubrerythrin [Bacteroides paurosaccharolyticus]
WP_005940397.1 183   hypothetical protein [Bacteroides massiliensis]

WP_016276007.1	183	MULTISPECIES: hypothetical protein [Bacteroides]
WP_042274929.1	183	reverse rubrerythrin-1 [Clostridium dakarense]
CDC18181.1	183	rubrerythrin [Clostridium sp. CAG:306]
CDE42050.1	183	rubrerythrin [Prevotella sp. CAG:279]
CDA77590.1	183	putative uncharacterized protein [Bacteroides sp.CAG:530]
WP_042275143.1	183	reverse rubrerythrin-1 [Clostridium dakarense]
WP_039678051.1	183	reverse rubrerythrin-1 [Terrisporobacter othiniensis]
WP_028306644.1	183	reverse rubrerythrin-1 [Desulfitibacter alkalitolerans]
WP_040197811.1	183	reverse rubrerythrin-1 [Candidatus Soleaferrea massiliensis]
WP_018592409.1	183	reverse rubrerythrin-1 [Terrisporobacter glycolicus]
WP_026900898.1	183	reverse rubrerythrin-1 [Peptostreptococcaceae bacterium VA2]
CDD91123.1	183	rubrerythrin [Coprobacillus sp. CAG:826]
WP_028856722.1	183	reverse rubrerythrin-1 [Psychrilyobacter atlanticus]
WP_028856911.1	183	reverse rubrerythrin-1 [Psychrilyobacter atlanticus]
WP_013387972.1	183	rubrerythrin [Ilyobacter polytropus]
WP_028856296.1	183	reverse rubrerythrin-1 [Psychrilyobacter atlanticus]
WP_039680914.1	183	reverse rubrerythrin-1 [Terrisporobacter othiniensis]
CCC72339.1	183	rubredoxin [Megasphaera elsdenii DSM 20460]
WP_019678632.1	183	reverse rubrerythrin-1 [Ruminococcus flavefaciens]
CDF05419.1	183	rubredoxin [Megasphaera elsdenii CAG:570]
CCZ45039.1	183	uncharacterized protein BN702_01098 [Bacteroides sp.CAG:545]
CDF00458.1	183	rubrerythrin [Ruminococcus sp. CAG:624]
CBK98310.1	183	Rubrerythrin [Faecalibacterium prausnitzii L2-6]
CDA93412.1	183	rubrerythrin [Bacteroides sp. CAG:709]
WP_035394078.1	183	reverse rubrerythrin-1 [Faecalibacterium prausnitzii]
WP_005926148.1	183	hypothetical protein [Faecalibacterium prausnitzii]
CDC64421.1	183	rubredoxin [Bacteroides sp. CAG:770]
WP_055187975.1	183	reverse rubrerythrin-1 [Faecalibacterium prausnitzii]
EEX69070.1	183	rubredoxin [Mitsuokella multacida DSM 20544]
WP_009984629.1	183	rubrerythrin [Ruminococcus flavefaciens]
WP_005947058.1	183	hypothetical protein [Fusobacterium varium]
WP_005980926.1	183	hypothetical protein [Fusobacterium ulcerans]
WP_022287215.1	183	rubredoxin [Ruminococcus bicirculans]

WP_028520467.1	183	reverse rubrerythrin-1 [Ruminococcus flavefaciens]
CDC11322.1	183	putative uncharacterized protein [Clostridium sp.CAG:413]
WP_024859667.1	183	reverse rubrerythrin-1 [Ruminococcus flavefaciens]
WP_005352561.1	183	hypothetical protein [[Eubacterium] siraeum]
WP_055142355.1	183	reverse rubrerythrin-1 [[Eubacterium] siraeum]
CDC49388.1	183	rubrerythrin [Eubacterium siraeum CAG:80]
CBK97321.1	183	Rubrerythrin [[[Eubacterium] siraeum 70/3]
WP_031560318.1	183	reverse rubrerythrin-1 [Ruminococcus flavefaciens]
WP_028516601.1	183	reverse rubrerythrin-1 [Ruminococcus flavefaciens]
CDD09842.1	183	putative uncharacterized protein [Clostridium sp.CAG:349]
CDC78241.1	183	putative uncharacterized protein [Clostridium sp.CAG:964]
CDA72344.1	183	rubredoxin [Ruminococcus sp. CAG:579]
WP_028515630.1	183	reverse rubrerythrin-1 [Ruminococcus flavefaciens]
CCX43055.1	183	rubredoxin [Prevotella sp. CAG:1031]
WP_012034775.1	183	rubrerythrin [Methanocella arvoryzae]
AKB33522.1	182	Rubrerythrin [Methanosarcina siciliae HI350]
WP_048181075.1	182	Rubrerythrin-2 [Methanosarcina siciliae]
WP_015412365.1	182	rubrerythrin [Methanosarcina mazei]
WP_048159363.1	182	Rubrerythrin-2 [Methanosarcina sp. WWM596]
WP_048041388.1	182	Rubrerythrin-2 [Methanosarcina mazei]
WP_011034120.1	182	rubrerythrin [Methanosarcina mazei]
WP_048173422.1	182	Rubrerythrin-2 [Methanosarcina siciliae]
WP_011021181.1	182	rubrerythrin [Methanosarcina acetivorans]
WP_048043868.1	182	MULTISPECIES: Rubrerythrin-2 [Methanosarcina]
WP_048124758.1	182	Rubrerythrin-2 [Methanosarcina lacustris]
WP_048128827.1	182	Rubrerythrin-2 [Methanosarcina sp. WH1]
WP_048038848.1	182	Rubrerythrin-2 [Methanosarcina mazei]
WP_048119122.1	182	Rubrerythrin-2 [Methanosarcina barkeri]
WP_048166844.1	182	Rubrerythrin-2 [Methanosarcina thermophila]
WP_054299261.1	182	Rubrerythrin-2 [Methanosarcina sp. E03.2]
WP_011306877.1	182	rubrerythrin [Methanosarcina barkeri]
WP_048121642.1	182	Rubrerythrin-2 [Methanosarcina vacuolata]
WP_048092966.1	182	Rubrerythrin-2 [Candidatus Methanoperedens nitroreducens]
WP_048156958.1	182	Rubrerythrin-2 [Methanosarcina sp. Kolksee]
WP_048141397.1	182	Rubrerythrin-2 [Methanosarcina horonobensis]
WP_048107351.1	182	Rubrerythrin-2 [Methanosarcina barkeri]
WP_011308471.1	182	rubrerythrin [Methanosarcina barkeri]
WP_048117216.1	182	Rubrerythrin-2 [Methanosarcina barkeri]
WP_042678437.1	182	Rubrerythrin-2 [Anaerosalibacter sp. ND1]
WP_034601978.1	182	Rubrerythrin-2 [Clostridiisalibacter paucivorans]

WP_052563077.1 182   Rubrerythrin-2 [Candidatus Brocadia sinica]
WP_007221336.1 182   rubrerythrin [Candidatus Jettenia caeni]
CAJ75126.1   182   similar to rubrerythrin [Candidatus Kuenenia stuttgartiensis]
ESQ20412.1   182   Rubrerythrin [uncultured Acidilobus sp. MG]
WP_034822175.1 182   rubrerythrin [[Eubacterium] nodatum]
WP_044908687.1 182   rubrerythrin [Lachnospiraceae bacterium MC2017]
WP_027113898.1 182   rubrerythrin [Lachnospiraceae bacterium NK4A144]
WP_054251899.1 182   Rubrerythrin-2 [Clostridiales bacterium SIT13]
WP_036931399.1 182   rubrerythrin [Proteocatella sphenisci]
WP_007788586.1 182   rubrerythrin [Peptostreptococcus stomatis]
WP_034924990.1 182   rubrerythrin [Erysipelotrichaceae bacterium NK3D112]
EGG85349.1   182   hypothetical protein HMPREF1025_01753 [Lachnospiraceae bacterium 3_1_46FAA]
WP_036763491.1 182   rubrerythrin [Peptostreptococcus sp. MV1]
WP_040627466.1 182   rubrerythrin [Solobacterium moorei]
CCX55312.1   182   rubrerythrin [Veillonella sp. CAG:933]
CDD01112.1   182   rubrerythrin [Prevotella sp. CAG:474]
WP_006555959.1 182   hypothetical protein [Veillonella ratti]
WP_007158000.1 182   rubrerythrin [Oribacterium sinus]
WP_009533907.1 182   rubrerythrin [Oribacterium parvum]
WP_002843693.1 182   rubrerythrin [Peptostreptococcus anaerobius]
WP_012448784.1 182   rubrerythrin [Natranaerobius thermophilus]
WP_009428683.1 182   MULTISPECIES: rubrerythrin [Oribacterium]
WP_037403892.1 182   rubrerythrin [Solobacterium moorei]
WP_005840266.1 182   rubrerythrin [Mitsuokella multacida]
WP_009352954.1 182   rubrerythrin [Veillonella sp. oral taxon 780]
WP_040636889.1 182   rubrerythrin [Mitsuokella sp. oral taxon 131]
KJS11395.1   182   rubrerythrin [Peptococcaceae bacterium BRH_c8a]
WP_013787581.1 182   rubrerythrin [Thermoanaerobacterium xylanolyticum]
EDS10832.1   182   rubredoxin [Anaerotruncus colihominis DSM 17241]
WP_045410680.1 182   Rubrerythrin-2 [Thermoanaerobacterium saccharolyticum]
WP_014759338.1 182   rubrerythrin [Thermoanaerobacterium aotearoense]
WP_014968330.1 182   rubrerythrin [Gottschalkia acidurici]
WP_055160953.1 182   rubrerythrin [Mitsuokella jalaludinii]
WP_036378317.1 182   rubrerythrin [Mitsuokella jalaludinii]
WP_028254497.1 182   rubrerythrin [Veillonella magna]
WP_015312142.1 182   rubrerythrin [Thermoanaerobacterium thermosaccharolyticum]
WP_013298486.1 182   rubrerythrin [Thermoanaerobacterium thermosaccharolyticum]
KQM09791.1   182   rubrerythrin [Methanomassiliicoccales archaeon

RumEn M2]
WP_036826810.1 182   rubrerythrin [Porphyromonadaceae bacterium COT-184 OH4590]
WP_007221337.1 182   rubrerythrin [Candidatus Jettenia caeni]
WP_026022299.1 182   rubrerythrin [Clostridium senegalense]
ABC77889.1   182   rubrerythrin [Syntrophus aciditrophicus SB]
WP_005375473.1 182   rubrerythrin [Veillonella atypica]
CDD97408.1   182   rubrerythrin [Akkermansia sp. CAG:344]
WP_031930584.1 182   hypothetical protein [Akkermansia muciniphila]
WP_012419913.1 182   rubrerythrin [Akkermansia muciniphila]
WP_021773695.1 182   rubrerythrin [Oribacterium sp. oral taxon 078]
WP_040652863.1 182   rubrerythrin [Oribacterium sp. oral taxon 078]
WP_053551244.1 182   reverse rubrerythrin-1 [Desulfuromonas sp. WTL]
WP_006000948.1 182   rubrerythrin [Desulfuromonas acetoxidans]
WP_020678183.1 182   reverse rubrerythrin-1 [Geopsychrobacter electrodiphilus]
WP_036936366.1 182   reverse rubrerythrin-1 [Pseudobacteroides cellulosolvens]
WP_002598340.1 182   hypothetical protein [Clostridium colicanis]
WP_039635209.1 182   reverse rubrerythrin-1 [Clostridium argentinense]
WP_046494822.1 182   reverse rubrerythrin-1 [Syntrophomonas zehnderi]
WP_026939382.1 182   reverse rubrerythrin-1 [Holophaga foetida]
WP_014825481.1 182   rubrerythrin [Desulfosporosinus acidiphilus]
WP_013840449.1 182   rubrerythrin [Desulfotomaculum ruminis]
WP_026883500.1 182   reverse rubrerythrin-1 [Clostridium akagii]
WP_047811093.1 182   reverse rubrerythrin-1 [Desulfosporosinus acididurans]
WP_026901415.1 182   reverse rubrerythrin-1 [Peptostreptococcaceae bacterium VA2]
WP_010249727.1 182   rubrerythrin [Acetivibrio cellulolyticus]
WP_021770447.1 182   rubredoxin [Mitsuokella sp. oral taxon 131]
WP_013505958.1 182   rubrerythrin [Desulfurispirillum indicum]
WP_027389513.1 182   reverse rubrerythrin-1 [Chrysiogenes arsenatis]
WP_027308166.1 182   reverse rubrerythrin-1 [Caloramator sp. ALD01]
WP_027406964.1 182   reverse rubrerythrin-1 [Anaerovibrio sp. RM50]
WP_047382460.1 182   MULTISPECIES: reverse rubrerythrin-1 [Cetobacterium]
WP_026896255.1 182   reverse rubrerythrin-1 [Clostridiisalibacter paucivorans]
WP_023051559.1 182   rubrerythrin [Cetobacterium somerae]
WP_009134550.1 182   rubrerythrin [Alistipes indistinctus]
WP_027397212.1 182   reverse rubrerythrin-1 [Anaerovibrio lipolyticus]
WP_039210555.1 182   reverse rubrerythrin-1 [Anaerovibrio lipolyticus]
WP_003367602.1 182   rubrerythrin [Clostridium botulinum]

WP_039255828.1 182   reverse rubrerythrin-1 [Clostridium novyi]
WP_028511003.1 182   reverse rubrerythrin-1 [Ruminococcus sp. NK3A76]
WP_018336370.1 182   rubrerythrin [Butyricimonas synergistica]
WP_008677212.1 182   reverse rubrerythrin-1 [Clostridium sp. 7_2_43FAA]
WP_025277476.1 182   rubrerythrin [Barnesiella viscericola]
WP_008862038.1 182   rubrerythrin [Barnesiella intestinihominis]
WP_027202454.1 182   rubrerythrin [Butyricimonas virosa]
WP_008680413.1 182   reverse rubrerythrin-1 [Clostridium sp. 7_2_43FAA]
WP_029169419.1 182   reverse rubrerythrin-1 [Clostridium botulinum]
KLU66640.1   182   reverse rubrerythrin-1 [Desulfosporosinus acididurans]
WP_012103749.1 182   hypothetical protein [Clostridium kluyveri]
WP_038291063.1 182   reverse rubrerythrin-1 [[Clostridium]straminisolvens]
WP_035135384.1 182   reverse rubrerythrin-1 [Clostridium sulfidigenes]
WP_040329673.1 182   reverse rubrerythrin-1 [Candidatus Clostridium anorexicamassiliense]
WP_044035896.1 182   reverse rubrerythrin-1 [Clostridium bornimense]
WP_044035895.1 182   reverse rubrerythrin-1 [Clostridium bornimense]
AFM40740.1   182   rubrerythrin [Desulfosporosinus acidiphilus SJ4]
WP_041687854.1 182   reverse rubrerythrin-1 [[Eubacterium] eligens]
WP_003376185.1 182   rubrerythrin [Clostridium sp. K25]
WP_039258096.1 182   reverse rubrerythrin-1 [Clostridium botulinum]
WP_039216866.1 182   MULTISPECIES: reverse rubrerythrin-1 [Clostridium]
WP_041138126.1 182   reverse rubrerythrin-1 [Clostridiaceae bacterium GM1]
WP_052221559.1 182   reverse rubrerythrin-1 [Clostridium homopropionicum]
KPA16744.1   182   rubredoxin/rubrerythrin [Candidatus Magnetomorum sp.HK-1]
WP_025437095.1 182   reverse rubrerythrin-1 [Eubacterium acidaminophilum]
WP_053289670.1 182   reverse rubrerythrin-1 [Clostridium botulinum]
WP_010293799.1 182   rubrerythrin [Clostridium senegalense]
CCY16543.1   182   rubrerythrin [Prevotella sp. CAG:755]
CDF21346.1   182   reverse rubrerythrin-2 [Prevotella sp. CAG: 617]
WP_038263140.1 182   reverse rubrerythrin-1 [[Clostridium] litorale]
WP_026900654.1 182   reverse rubrerythrin-1 [Peptostreptococcaceae bacterium VA2]
WP_011461089.1 182   hypothetical protein [Desulfitobacterium

hafniense]
WP_021169717.1 182   MULTISPECIES: reverse rubrerythrin-1 [Sporomusa]
WP_018660703.1 182   rubrerythrin [Thermobrachium celere]
WP_006193450.1 182   MULTISPECIES: rubredoxin/rubrerythrin [Selenomonas]
WP_004092112.1 182   rubredoxin/rubrerythrin domain protein [Acetonema longum]
WP_007392899.1 182   rubredoxin [Megasphaera sp. UPII 135-E]
WP_034420202.1 182   reverse rubrerythrin-1 [Clostridiales bacterium DRI-13]
WP_012282616.1 182   rubredoxin/rubrerythrin domain protein[Heliobacterium modesticaldum]
WP_009347310.1 182   rubrerythrin [Alloprevotella rava]
WP_023053896.1 182   rubrerythrin [Megasphaera sp. BV3C16-1]
WP_027128888.1 182   reverse rubrerythrin-1 [Fusobacterium perfoetens]
WP_012937701.1 182   rubrerythrin [Acidaminococcus fermentans]
WP_027894728.1 182   reverse rubrerythrin-1 [Megasphaera elsdenii]
CDA62669.1   182   reverse rubrerythrin-1 [Clostridium sp. CAG:169]
WP_012938354.1 182   rubrerythrin [Acidaminococcus fermentans]
WP_020311478.1 182   MULTISPECIES: rubredoxin [Megasphaera]
CCX41174.1   182   rubredoxin [Clostridium sp. CAG:1024]
CCX55191.1   182   putative uncharacterized protein [Bacteroides sp.CAG:1060]
WP_044504562.1 182   reverse rubrerythrin-1 [Megasphaera massiliensis]
WP_048515280.1 182   reverse rubrerythrin-1 [Megasphaera cerevisiae]
WP_010166197.1 182   rubrerythrin [Epulopiscium sp. 'N.t. morphotype B']
WP_038672522.1 182   reverse rubrerythrin-1 [Pelosinus sp. UFO1]
WP_006943235.1 182   rubredoxin [Megasphaera micronuciformis]
WP_055175846.1 182   reverse rubrerythrin-1 [Lachnospira pectinoschiza]
CDE20207.1   182   putative uncharacterized protein [Eubacterium sp.CAG:841]
WP_013657958.1 182   rubrerythrin [Cellulosilyticum lentocellum]
WP_054330887.1 182   reverse rubrerythrin-1 [Clostridia bacterium UC5.1-2F7]
WP_055216971.1 182   reverse rubrerythrin-1 [[Eubacterium] eligens]
WP_002847084.1 182   rubredoxin [Ruminococcus albus]
WP_055286907.1 182   reverse rubrerythrin-1 [[Eubacterium] eligens]
CDD34454.1   182   rubrerythrin domain protein [Roseburia sp. CAG:309]
WP_016317990.1 182   reverse rubrerythrin-1 [Anaerotruncus sp. G3(2012)]
WP_024857963.1 182   reverse rubrerythrin-1 [Ruminococcus albus]
WP_013976877.1 182   hypothetical protein [Clostridium sp. SY8519]

CCY12599.1	182	rubredoxin [Eubacterium sp. CAG:146]
WP_009015448.1	182	MULTISPECIES: rubredoxin/ rubrerythrin[Acidaminococcus]
WP_027938397.1	182	reverse rubrerythrin-1 [Anaeroarcus burkinensis]
WP_013499606.1	182	rubrerythrin [Ruminococcus albus]
WP_018702663.1	182	reverse rubrerythrin-1 [Anaeromusa acidaminophila]
CCZ20923.1	182	putative uncharacterized protein [Ruminococcus sp.CAG:724]
WP_029474072.1	182	reverse rubrerythrin-1 [Clostridiales bacterium VE202-08]
CDB41006.1	182	putative uncharacterized protein [Ruminococcus sp.CAG:177]
WP_022938418.1	182	reverse rubrerythrin-1 [Dielma fastidiosa]
CDA79519.1	182	reverse rubrerythrin-1 [Clostridium sp. CAG: 242]
CCY04419.1	182	putative uncharacterized protein [Faecalibacterium sp.CAG:1138]
CCZ83557.1	182	putative uncharacterized protein [Ruminococcus sp.CAG:254]
CDE11924.1	182	rubrerythrin [Ruminococcus sp. CAG:330]
WP_021683652.1	182	rubredoxin [Ruminococcus callidus]
KPJ60469.1	181	Rubrerythrin-2 [Latescibacteria bacterium DG_63]
WP_026888973.1	181	Rubrerythrin-2 [Clostridium beijerinckii]
WP_042678685.1	181	Rubrerythrin-2 [Anaerosalibacter sp. ND1]
EKD27216.1	181	nigerythrin [uncultured bacterium]
CDA90916.1	181	rubredoxin [Firmicutes bacterium CAG:238]
WP_027204281.1	181	rubrerythrin [Butyrivibrio fibrisolvens]
WP_027217530.1	181	rubrerythrin [Butyrivibrio fibrisolvens]
WP_046444360.1	181	rubrerythrin [Catabacter hongkongensis]
CCY24708.1	181	putative uncharacterized protein [Brachyspira sp.CAG:484]
WP_004033725.1	181	rubrerythrin [Methanobrevibacter smithii]
WP_034232510.1	181	rubrerythrin [Lachnospiraceae bacterium AC2029]
WP_019265530.1	181	rubrerythrin [Methanobrevibacter smithii]
WP_009643717.1	181	MULTISPECIES: rubrerythrin [Mogibacterium]
CCZ19813.1	181	rubrerythrin [Ruminococcus sp. CAG:724]
WP_035433052.1	181	rubrerythrin [Atopobium parvulum]
WP_035428857.1	181	rubrerythrin [Atopobium sp. ICM42b]
WP_016477553.1	181	hypothetical protein [Atopobium sp. oral taxon 199]
WP_012956166.1	181	rubrerythrin [Methanobrevibacter ruminantium]
WP_023060020.1	181	nigerythrin [Peptoniphilus sp. BV3AC2]
WP_008902469.1	181	rubrerythrin [Peptoniphilus duerdenii]
EEU95343.1	181	Rubrerythrin [Faecalibacterium prausnitzii A2-165]
WP_016358893.1	181	rubrerythrin [Methanobrevibacter sp. AbM4]

WP_003149581.1 181   rubrerythrin [Atopobium rimae]
WP_027871971.1 181   rubrerythrin [[Eubacterium] cellulosolvens]
WP_013252552.1 181   rubrerythrin [Olsenella uli]
WP_035437419.1 181   rubrerythrin [Atopobium sp. BS2]
WP_022790506.1 181   rubrerythrin [Faecalicoccus pleomorphus]
WP_040681576.1 181   rubrerythrin [Methanobrevibacter boviskoreani]
WP_042708061.1 181   rubrerythrin [Methanobrevibacter wolinii]
WP_023056273.1 181   nigerythrin [Peptoniphilus sp. BV3C26]
WP_022783138.1 181   rubrerythrin [Lachnospiraceae bacterium NK4A179]
WP_036574800.1 181   rubrerythrin [Olsenella uli]
WP_019132477.1 181   rubrerythrin [Peptoniphilus obesi]
WP_050355095.1 181   Rubrerythrin-2 [[Clostridium] purinilyticum]
WP_009430015.1 181   rubrerythrin [Peptoniphilus sp. oral taxon 375]
WP_042692434.1 181   rubrerythrin [Methanobrevibacter oralis]
WP_012808218.1 181   rubrerythrin [Atopobium parvulum]
WP_019189544.1 181   hypothetical protein [Levyella massiliensis]
WP_006964627.1 181   rubrerythrin-2 NADH peroxidase Rbr [Desulfotignum phosphitoxidans]
WP_038118186.1 181   rubrerythrin [Veillonella sp. AS16]
WP_024335103.1 181   Rubrerythrin-2 [Desulfotignum balticum]
WP_019214373.1 181   hypothetical protein [Fenollaria massiliensis]
CDE51587.1   181   rubrerythrin [Faecalibacterium sp. CAG:74]
WP_009351849.1 181   rubrerythrin [Veillonella sp. oral taxon 158]
WP_053964888.1 181   Rubrerythrin-2 [Clostridiales bacterium mt11]
WP_054748060.1 181   rubrerythrin [Veillonella rogosae]
WP_034438494.1 181   Rubrerythrin-2 [Tissierellia bacterium S5-A11]
WP_032110601.1 181   rubrerythrin [bacterium OL-1]
WP_055078131.1 181   Rubrerythrin-2 [Peptoniphilaceae bacterium SIT14]
WP_005387792.1 181   rubrerythrin [Veillonella dispar]
WP_040379809.1 181   rubrerythrin [Dialister succinatiphilus]
CBL15429.1   181   Rubrerythrin [Ruminococcus bromii L2-63]
WP_021147736.1 181   rubrerythrin [Veillonella parvula]
WP_012863888.1 181   rubrerythrin [Veillonella parvula]
CCX83958.1   181   rubrerythrin [Ruminococcus sp. CAG:108]
WP_004693830.1 181   rubrerythrin [Veillonella parvula]
WP_045078025.1 181   Rubrerythrin-2 [Peptoniphilus sp. 1-1]
WP_054253146.1 181   Rubrerythrin-2 [Clostridiales bacterium SIT11]
WP_008602900.1 181   MULTISPECIES: rubrerythrin [Veillonella]
WP_009278343.1 181   rubrerythrin [Olsenella sp. oral taxon 809]
WP_012955537.1 181   rubrerythrin [Methanobrevibacter ruminantium]
WP_021725159.1 181   rubrerythrin [Olsenella profusa]
WP_044975742.1 181   rubrerythrin [Ruminococcus sp. HUN007]
WP_005583253.1 181   Rubrerythrin-2 [[Clostridium] ultunense]
WP_036728664.1 181   Rubrerythrin-2 [Peptoniphilus sp. ChDC B134]
WP_021734392.1 181   rubrerythrin [Coriobacteriaceae bacterium BV3Ac1]
WP_009222283.1 181   rubrerythrin [Peptoniphilus sp. oral taxon

386]
WP_002562867.1 181   MULTISPECIES: hypothetical protein [Atopobium]
WP_028263668.1 181   rubrerythrin [Atopobium fossor]
WP_040381840.1 181   rubrerythrin [Dialister invisus]
WP_028256984.1 181   rubrerythrin [Veillonella montpellierensis]
WP_040210307.1 181   rubrerythrin [Clostridium polynesiense]
WP_005379557.1 181   MULTISPECIES: rubrerythrin [Veillonella]
WP_038151249.1 181   rubrerythrin [Veillonella montpellierensis]
AEA47072.1   181   Rubrerythrin [Archaeoglobus veneficus SNP6]
WP_048571236.1 181   rubrerythrin [Clostridium cylindrosporum]
KKK44280.1   181   Rubrerythrin-2 [Lokiarchaeum sp. GC14_75]
WP_035785124.1 181   rubrerythrin [Clostridium botulinum]
WP_012424796.1 181   rubrerythrin [Clostridium botulinum]
WP_010966861.1 181   reverse rubrerythrin-1 [Clostridium acetobutylicum]
WP_003371395.1 181   rubrerythrin [Clostridium botulinum]
WP_010966860.1 181   Reverse rubrerythrin-2 [Clostridium acetobutylicum]
WP_012449820.1 181   rubrerythrin [Clostridium botulinum]
EKQ50160.1   181   rubrerythrin [Clostridium sp. Maddingley MBC34-26]
WP_017209325.1 181   reverse rubrerythrin-1 [Clostridium beijerinckii]
WP_023976625.1 181   MULTISPECIES: reverse rubrerythrin-1 [Clostridium]
WP_012058541.1 181   rubrerythrin [Clostridium beijerinckii]
WP_026887193.1 181   reverse rubrerythrin-1 [Clostridium beijerinckii]
WP_040210278.1 181   reverse rubrerythrin-1 [Clostridium polynesiense]
WP_014902396.1 181   MULTISPECIES: rubrerythrin [Desulfosporosinus]
KJS49554.1   181   Reverse rubrerythrin-1 [Peptococcaceae bacterium BRH_c23]
WP_026901029.1 181   reverse rubrerythrin-1 [Peptostreptococcaceae bacterium VA2]
WP_009169244.1 181   rubrerythrin [Clostridium sp. DL-VIII]
WP_010075074.1 181   rubrerythrin [Clostridium cellulovorans]
WP_007781113.1 181   rubrerythrin [Desulfosporosinus youngiae]
WP_032078492.1 181   reverse rubrerythrin-1 [Clostridium drakei]
WP_040329943.1 181   reverse rubrerythrin-1 [Candidatus Clostridium anorexicamassiliense]
WP_010241650.1 181   rubrerythrin [Clostridium arbusti]
WP_053243458.1 181   reverse rubrerythrin-1 [Clostridium sp. DMHC 10]
WP_007063001.1 181   rubrerythrin [Clostridium carboxidivorans]
WP_026766821.1 181   reverse rubrerythrin-1 [Selenomonas ruminantium]
WP_044040325.1 181   reverse rubrerythrin-1 [Clostridium bornimense]
WP_007063007.1 181   rubrerythrin [Clostridium carboxidivorans]

WP_015404341.1 181   rubrerythrin [Desulfocapsa sulfexigens]
WP_015617781.1 181   rubrerythrin [Clostridium pasteurianum]
WP_026760095.1 181   MULTISPECIES: reverse rubrerythrin-1 [Selenomonas]
WP_053985041.1 181   reverse rubrerythrin-1 [Lachnospiraceae bacterium mt14]
WP_009614089.1 181   rubredoxin family protein [Desulfosporosinus sp. OT]
WP_039680639.1 181   reverse rubrerythrin-1 [Terrisporobacter othiniensis]
WP_005839508.1 181   rubredoxin/rubrerythrin [Mitsuokella multacida]
WP_033170387.1 181   reverse rubrerythrin-1 [Selenomonas sp. ND2010]
WP_021368989.1 181   reverse rubrerythrin-1 [Peptoclostridium difficile]
WP_027638883.1 181   reverse rubrerythrin-1 [Clostridium cadaveris]
WP_019553452.1 181   reverse rubrerythrin-1 [Propionispira raffinosivorans]
WP_003445045.1 181   rubrerythrin [Clostridium pasteurianum]
WP_021375175.1 181   reverse rubrerythrin-1 [Peptoclostridium difficile]
WP_012198114.1 181   rubrerythrin [Lachnoclostridium phytofermentans]
WP_002579670.1 181   MULTISPECIES: reverse rubrerythrin-2 [Clostridium]
WP_011928126.1 181   hypothetical protein [Pelotomaculum thermopropionicum]
WP_032117754.1 181   reverse rubrerythrin-1 [Clostridium sp. CL-2]
WP_003428360.1 181   rubrerythrin [Clostridium butyricum]
WP_040193195.1 181   reverse rubrerythrin-1 [Clostridium sp. CL-6]
WP_024833227.1 181   reverse rubrerythrin-1 [[Clostridium] josui]
WP_015706696.1 181   rubredoxin/rubrerythrin [Treponema primitia]
WP_018591402.1 181   reverse rubrerythrin-1 [Terrisporobacter glycolicus]
WP_029543055.1 181   reverse rubrerythrin-1 [Selenomonas ruminantium]
WP_050698519.1 181   reverse rubrerythrin-1 [Ruminococcaceae bacterium mt9]
WP_010296925.1 181   rubrerythrin [Clostridium senegalense]
WP_013240531.1 181   rubrerythrin [Clostridium ljungdahlii]
WP_017753262.1 181   reverse rubrerythrin-1 [Clostridium tyrobutyricum]
WP_023162461.1 181   rubrerythrin [Clostridium autoethanogenum]
WP_055264246.1 181   reverse rubrerythrin-1 [Clostridium disporicum]
WP_031518007.1 181   reverse rubrerythrin-1 [Desulfotomaculum alkaliphilum]
WP_034841783.1 181   reverse rubrerythrin-1 [[Clostridium] cellulosi]

WP_047809427.1 181   reverse rubrerythrin-1 [Desulfosporosinus acididurans]
WP_015924434.1 181   rubrerythrin [[Clostridium] cellulolyticum]
WP_009536750.1 181   hypothetical protein [Oribacterium asaccharolyticum]
WP_009213845.1 181   rubredoxin/rubrerythrin [Oribacterium sp. oral taxon 078]
WP_025486236.1 181   MULTISPECIES: reverse rubrerythrin-1 [Clostridiales]
ACR71087.1   181   ferredoxin hydrogenase [[[Eubacterium] eligens ATCC 27750]
WP_015392615.1 181   rbr3B: reverse rubrerythrin-2 [Clostridium saccharoperbutylacetonicum]
WP_041276384.1 181   reverse rubrerythrin-1 [Desulfosporosinus acidiphilus]
WP_050606614.1 181   reverse rubrerythrin-1 [Clostridium sp. mt5]
WP_006719183.1 181   rubrerythrin [Desulfitobacterium metallireducens]
WP_010296937.1 181   rubrerythrin [Clostridium senegalense]
WP_054743728.1 181   reverse rubrerythrin-1 [Cellulosilyticum ruminicola]
WP_004622187.1 181   rubrerythrin [[Clostridium] papyrosolvens]
WP_003357610.1 181   rubredoxin/rubrerythrin [Clostridium botulinum]
WP_023388933.1 181   reverse rubrerythrin-1 [Youngiibacter fragilis]
WP_032121531.1 181   reverse rubrerythrin-1 [Clostridium sp. LF2]
KJU83676.1   181   rubredoxin/rubrerythrin [Candidatus Magnetobacterium bavaricum]
WP_053469624.1 181   reverse rubrerythrin-1 [Clostridium sp. L74]
CDD84735.1   181   rubrerythrin [Bacteroides sp. CAG:462]
CBE03793.1   181   putative ruberythrin [Peptoclostridium difficile R20291]
WP_029452984.1 181   reverse rubrerythrin-1 [Clostridium algidicarnis]
WP_054680259.1 181   reverse rubrerythrin-1 [Clostridium sp. Hs50]
WP_003483406.1 181   MULTISPECIES: hypothetical protein [Clostridium]
WP_003361641.1 181   MULTISPECIES: rubredoxin/rubrerythrin [Clostridium]
WP_019851405.1 181   reverse rubrerythrin-1 [Desulfitobacterium sp. PCE1]
WP_041346489.1 181   reverse rubrerythrin-1 [Clostridium botulinum]
KJU81992.1   181   Rubrerythrin domain protein [Candidatus Magnetobacterium bavaricum]
WP_029162014.1 181   reverse rubrerythrin-1 [Clostridium scatologenes]
WP_011876967.1 181   rubrerythrin [Desulfotomaculum reducens]
WP_014795660.1 181   rubrerythrin [Desulfitobacterium dehalogenans]
WP_015263655.1 181   rubrerythrin [Desulfitobacterium

dichloroeliminans]
WP_021422950.1 181   reverse rubrerythrin-1 [Peptoclostridium difficile]
WP_003420048.1 181   rubredoxin/rubrerythrin [Peptoclostridium difficile]
WP_027637677.1 181   reverse rubrerythrin-1 [Clostridium cadaveris]
WP_014314678.1 181   MULTISPECIES: rubrerythrin [Clostridiales]
WP_021122238.1 181   reverse rubrerythrin-1 [[Clostridium] sordellii]
WP_011099088.1 181   rubredoxin/rubrerythrin domain-containing protein[Clostridium tetani]
WP_025985730.1 181   reverse rubrerythrin-1 [Peptoclostridium difficile]
WP_040192765.1 181   reverse rubrerythrin-1 [Clostridium sp. CL-6]
WP_005815821.1 181   rubredoxin [Desulfitobacterium hafniense]
WP_004627411.1 181   MULTISPECIES: rubrerythrin [Ruminiclostridium]
WP_021386818.1 181   reverse rubrerythrin-1 [Peptoclostridium difficile]
WP_023437670.1 181   rubredoxin/rubrerythrin domain-containingprotein[Clostridium tetani]
WP_042274928.1 181   reverse rubrerythrin-1 [Clostridium dakarense]
WP_003497225.1 181   MULTISPECIES: hypothetical protein [Clostridiales]
WP_021381897.1 181   reverse rubrerythrin-1 [Peptoclostridium difficile]
WP_021401233.1 181   reverse rubrerythrin-1 [Peptoclostridium difficile]
WP_027637675.1 181   reverse rubrerythrin-1 [Clostridium cadaveris]
WP_027637684.1 181   reverse rubrerythrin-1 [Clostridium cadaveris]
WP_054273453.1 181   reverse rubrerythrin-1 [Peptoclostridium difficile]
WP_009889371.1 181   rubrerythrin [Peptoclostridium difficile]
WP_003419930.1 181   rubredoxin/rubrerythrin [Peptoclostridium difficile]
WP_015945311.1 181   rubrerythrin [Desulfitobacterium hafniense]
WP_040192758.1 181   reverse rubrerythrin-1 [Clostridium sp. CL-6]
WP_054335332.1 181   reverse rubrerythrin-1 [Clostridia bacterium UC5.1-2E1]
WP_055667565.1 181   reverse rubrerythrin-1 [Clostridiaceae bacterium mt10]
WP_008907425.1 181   rubrerythrin [Caloramator australicus]
WP_014425238.1 181   rubredoxin [Selenomonas ruminantium]
WP_021369481.1 181   reverse rubrerythrin-1 [Peptoclostridium difficile]
WP_006440192.1 181   hypothetical protein [[Clostridium] hiranonis]
WP_021124551.1 181   reverse rubrerythrin-1 [[Clostridium] sordellii]
WP_021360524.1 181   reverse rubrerythrin-1 [Peptoclostridium difficile]
WP_021363476.1 181   reverse rubrerythrin-1 [Peptoclostridium

difficile]
WP_021433528.1 181   reverse rubrerythrin-1 [[Clostridium] bifermentans]
WP_021429493.1 181   MULTISPECIES: reverse rubrerythrin-1 [Clostridiales]
WP_024620053.1 181   reverse rubrerythrin-1 [[Clostridium] mangenotii]
WP_027701876.1 181   reverse rubrerythrin-1 [[Clostridium] mangenotii]
WP_055337486.1 181   reverse rubrerythrin-1 [[Clostridium] sordellii]
WP_055336033.1 181   reverse rubrerythrin-1 [[Clostridium] sordellii]
WP_021418689.1 181   reverse rubrerythrin-1 [Peptoclostridium difficile]
WP_025985727.1 181   reverse rubrerythrin-1 [Peptoclostridium difficile]
WP_054260861.1 181   reverse rubrerythrin-1 [Propionispora sp. Iso2/2]
WP_019542785.1 181   reverse rubrerythrin-1 [Selenomonas bovis]
WP_021384192.1 181   reverse rubrerythrin-1 [Peptoclostridium difficile]
WP_021390415.1 181   reverse rubrerythrin-1 [Peptoclostridium difficile]
WP_031584299.1 181   reverse rubrerythrin-1 [Selenomonas bovis]
WP_046823632.1 181   reverse rubrerythrin-1 [Clostridium sp. JC272]
WP_018112779.1 181   reverse rubrerythrin-1 [Peptoclostridium difficile]
WP_029502595.1 181   reverse rubrerythrin-1 [Lachnoclostridium phytofermentans]
WP_021428523.1 181   MULTISPECIES: reverse rubrerythrin-1 [Clostridiales]
WP_036376069.1 181   reverse rubrerythrin-1 [Mitsuokella jalaludinii]
WP_014968562.1 181   rubredoxin/rubrerythrin [Gottschalkia acidurici]
WP_007390382.1 181   MULTISPECIES: rubredoxin [Megasphaera]
WP_027626955.1 181   reverse rubrerythrin-1 [Terrisporobacter glycolicus]
CCY26177.1   181   putative uncharacterized protein [Firmicutes bacterium CAG:114]
WP_008539147.1 181   MULTISPECIES: reverse rubrerythrin-1 [Megamonas]
WP_027889027.1 181   reverse rubrerythrin-1 [Megamonas hypermegale]
WP_027104250.1 181   reverse rubrerythrin-1 [Lachnospiraceae bacterium V9D3004]
WP_057979336.1 181   reverse rubrerythrin-1 [Caloramator mitchellensis]
WP_009428022.1 181   rubredoxin [Oribacterium sp. oral taxon 108]
WP_022784106.1 181   reverse rubrerythrin-1 [Lachnospiraceae

bacterium NK4A179]

WP_013272124.1	181	rubrerythrin [[Clostridium] saccharolyticum]
CDC38988.1	181	rubrerythrin [Clostridium sp. CAG:352]
WP_028506104.1	181	reverse rubrerythrin-1 [Ruminococcus sp. FC2018]
WP_054323620.1	181	reverse rubrerythrin-1 [Clostridia bacterium UC5.1-1E11]
WP_003544592.1	181	MULTISPECIES: rubrerythrin [Desulfotomaculum]
WP_006875303.1	181	hypothetical protein [Anaerotruncus colihominis]
CCY90610.1	181	putative uncharacterized protein [Eubacterium sp.CAG:180]
CDB65585.1	181	ferredoxin hydrogenase [Eubacterium sp. CAG:248]
WP_022014119.1	181	rubrerythrin domain protein [Lachnospiraceae bacterium TF01-11]
WP_013388992.1	181	rubrerythrin [Ilyobacter polytropus]
CDA52160.1	181	putative uncharacterized protein [Clostridium sp.CAG:138]
WP_027399478.1	181	reverse rubrerythrin-1 [Anaerovorax odorimutans]
CDB25182.1	181	putative uncharacterized protein [Firmicutes bacterium CAG:552]
CDD30523.1	181	putative uncharacterized protein [Firmicutes bacterium CAG:94]
CDB69003.1	181	ferredoxin hydrogenase [Eubacterium sp. CAG:252]
KJS03434.1	181	Reverse rubrerythrin-1 [Peptococcaceae bacterium BRH_c4a]
CUQ76746.1	181	NADH peroxidase [Lachnospira pectinoschiza]
WP_019542424.1	181	hypothetical protein [Selenomonas bovis]
CDA91799.1	181	putative uncharacterized protein [Ruminococcus sp.CAG:563]
CDF10077.1	181	ferredoxin hydrogenase [Eubacterium sp. CAG:76]
WP_022748263.1	181	reverse rubrerythrin-1 [Lachnobacterium bovis]
WP_027422329.1	181	reverse rubrerythrin-1 [Lachnobacterium bovis]
WP_053982539.1	181	reverse rubrerythrin-1 [Lachnospiraceae bacterium mt14]
CDA40782.1	181	ferredoxin hydrogenase [Eubacterium eligens CAG:72]
CDE21329.1	181	putative uncharacterized protein [Acidiphilium sp.CAG:727]
WP_031584916.1	181	rubrerythrin [Selenomonas bovis]
WP_040635533.1	181	rubrerythrin [Mitsuokella multacida]
WP_042735634.1	181	reverse rubrerythrin-1 [Lachnospiraceae bacterium TWA4]
CDA19974.1	181	putative uncharacterized protein [Ruminococcus sp.CAG:488]
CDE94235.1	181	uncharacterized protein BN701_01689

[Acidaminococcus sp.CAG:542]
CUQ84707.1   181   NADH peroxidase [[Eubacterium] eligens]
WP_025488121.1 181   reverse rubrerythrin-1 [Clostridium sp. KLE 1755]
WP_036377470.1 181   rubrerythrin [Mitsuokella jalaludinii]
WP_008394151.1 181   MULTISPECIES: hypothetical protein [Clostridiales]
WP_055162053.1 181   rubrerythrin [Mitsuokella jalaludinii]
WP_055055809.1 181   reverse rubrerythrin-1 [Blautia obeum]
WP_007038534.1 181   hypothetical protein [[Clostridium] bolteae]
WP_003427482.1 181   rubredoxin [Peptoclostridium difficile]
WP_002592389.1 181   rubrerythrin [[Clostridium] clostridioforme]
WP_002576805.1 181   MULTISPECIES: rubrerythrin [Lachnoclostridium]
CDB45705.1   181   putative uncharacterized protein [Phascolarctobacterium sp. CAG:207]
WP_022375624.1 181   rubrerythrin [Anaerostipes hadrus]
WP_021413258.1 181   reverse rubrerythrin-1 [Peptoclostridium difficile]
WP_024737615.1 181   reverse rubrerythrin-1 [Clostridiales bacterium VE202-15]
WP_024737616.1 181   reverse rubrerythrin-1 [Clostridiales bacterium VE202-15]
WP_007862106.1 181   MULTISPECIES: hypothetical protein [Clostridiales]
WP_002578417.1 181   rubrerythrin [[Clostridium] bolteae]
WP_021418688.1 181   reverse rubrerythrin-1 [Peptoclostridium difficile]
WP_027435153.1 181   reverse rubrerythrin-1 [Lachnospiraceae bacterium AB2028]
WP_027642038.1 181   reverse rubrerythrin-1 [[Clostridium]clostridioforme]
WP_048928844.1 181   reverse rubrerythrin-1 [[Clostridium] bolteae]
WP_057571387.1 181   reverse rubrerythrin-1 [[Clostridium]clostridioforme]
WP_003529910.1 181   hypothetical protein [[Clostridium] leptum]
WP_007862081.1 181   MULTISPECIES: hypothetical protein [Clostridiales]
WP_002586763.1 181   rubrerythrin [[Clostridium] clostridioforme]
CCY70222.1   181   putative uncharacterized protein [Eubacterium sp.CAG:161]
CDA29638.1   181   putative uncharacterized protein [Eubacterium sp.CAG:156]
CDB34853.1   181   rubredoxin [Phascolarctobacterium sp. CAG:266]
WP_025578258.1 181   reverse rubrerythrin-1 [Blautia wexlerae]
WP_008705373.1 181   MULTISPECIES: hypothetical protein [Clostridiales]
WP_021127633.1 181   reverse rubrerythrin-1 [[Clostridium] sordellii]
WP_008723813.1 181   MULTISPECIES: hypothetical protein [unclassified Clostridiales (miscellaneous)]

WP_008688391.1 181   hypothetical protein [Eubacterium sp. 3_1_31]
WP_005361605.1 181   hypothetical protein [Eubacterium ventriosum]
WP_007862117.1 181   MULTISPECIES: hypothetical protein [Clostridiales]
CCY44619.1   181   putative uncharacterized protein [Clostridium sp. CAG:7]
CDA62420.1   181   putative uncharacterized protein [Firmicutes bacterium CAG:56]
CDB13530.1   181   rubrerythrin [Eubacterium sp. CAG:192]
WP_008723835.1 181   MULTISPECIES: hypothetical protein [unclassified Clostridiales (miscellaneous)]
WP_018112765.1 181   reverse rubrerythrin-1 [Peptoclostridium difficile]
CCZ03839.1   181   putative uncharacterized protein [Eubacterium sp.CAG:603]
WP_021720205.1 181   rubredoxin [Phascolarctobacterium succinatutens]
WP_021427426.1 181   reverse rubrerythrin-1 [Peptoclostridium difficile]
WP_021428526.1 181   reverse rubrerythrin-1 [[Clostridium] bifermentans]
WP_008723821.1 181   MULTISPECIES: hypothetical protein [unclassified Clostridiales (miscellaneous)]
WP_026835390.1 181   reverse rubrerythrin-1 [Eubacterium xylanophilum]
WP_022255097.1 181   hypothetical protein [Clostridia bacterium UC5.1-1D10]
CDD62318.1   181   putative uncharacterized protein [Clostridium sp.CAG:505]
WP_022462914.1 181   hypothetical protein [Fusicatenibacter saccharivorans]
WP_008375550.1 181   hypothetical protein [Coprococcus comes]
WP_024619944.1 181   MULTISPECIES: reverse rubrerythrin-1 [Clostridiales]
WP_055248064.1 181   reverse rubrerythrin-1 [Coprococcus comes]
CCY34029.1   181   ferredoxin hydrogenase [Ruminococcus sp. CAG:60]
WP_039903613.1 181   reverse rubrerythrin-1 [[Clostridium] spiroforme]
CCY97785.1   181   rubrerythrin [Ruminococcus sp. CAG:17]
WP_057567155.1 181   reverse rubrerythrin-1 [[Clostridium] sordellii]
WP_057584197.1 181   reverse rubrerythrin-1 [[Clostridium] sordellii]
CDC93674.1   181   putative uncharacterized protein [Firmicutes bacterium CAG:227]
WP_021434910.1 181   reverse rubrerythrin-1 [Peptoclostridium difficile]
WP_027096630.1 181   reverse rubrerythrin-1 [[Clostridium] viride]
CCX40997.1   181   putative uncharacterized protein [Firmicutes

bacterium CAG:102]
WP_054631319.1 181   reverse rubrerythrin-1 [[Clostridium] sordellii]
WP_057564995.1 181   reverse rubrerythrin-1 [[Clostridium] sordellii]
WP_057569606.1 181   reverse rubrerythrin-1 [[Clostridium] sordellii]
WP_021122239.1 181   reverse rubrerythrin-1 [[Clostridium] sordellii]
WP_026760838.1 181   MULTISPECIES: rubrerythrin [Selenomonas]
CCX50256.1   181   putative uncharacterized protein [Clostridium sp.CAG:226]
WP_021653835.1 181   rubredoxin [Clostridiales bacterium oral taxon 876]
WP_028128978.1 181   rubrerythrin [Selenomonas sp. AE3005]
WP_008792065.1 181   MULTISPECIES: hypothetical protein [Coprobacillus]
WP_022785697.1 181   reverse rubrerythrin-1 [Clostridiales bacterium NK3B98]
WP_026766088.1 181   rubrerythrin [Selenomonas ruminantium]
WP_033170043.1 181   rubrerythrin [Selenomonas sp. ND2010]
WP_009016227.1 181   MULTISPECIES: rubrerythrin [Acidaminococcus]
CCX38912.1   181   putative uncharacterized protein [Clostridium sp.CAG:1013]
WP_015559176.1 181   rubrerythrin [Ruminococcus champanellensis]
WP_055066445.1 181   reverse rubrerythrin-1 [Blautia obeum]
CBL21749.1   181   Rubrerythrin [Blautia obeum A2-162]
WP_015526390.1 181   rubrerythrin [Blautia sp. KLE 1732]
WP_055207507.1 181   rubrerythrin [Clostridium baratii]
WP_055226968.1 181   rubrerythrin [Clostridium baratii]
WP_021590131.1 180   rubrerythrin [Prevotella baroniae]
CDA16504.1   180   rubrerythrin [Clostridium sp. CAG:571]
CCY90684.1   180   rubrerythrin [Eubacterium sp. CAG:180]
CDD61490.1   180   rubrerythrin [Clostridium sp. CAG:505]
WP_009347617.1 180   rubrerythrin [Alloprevotella rava]
CCY01689.1   180   rubrerythrin [Prevotella sp. CAG:924]
WP_009164380.1 180   rubrerythrin [Pyramidobacter piscolens]
WP_055242907.1 180   rubrerythrin [Turicibacter sanguinis]
CDC26853.1   180   rubrerythrin [Firmicutes bacterium CAG:466]
WP_024854369.1 180   rubrerythrin [[Ruminococcus] gnavus]
WP_042705165.1 180   rubrerythrin [Methanomicrobium mobile]
AHF24944.1   180   rubrerythrin [uncultured bacterium Contig9]
WP_005348871.1 180   rubrerythrin [[Eubacterium] hallii]
WP_054332096.1 180   rubrerythrin [Clostridia bacterium UC5.1-2F7]
WP_009244495.1 180   rubrerythrin [[Ruminococcus] gnavus]
CCY13797.1   180   rubrerythrin [Eubacterium sp. CAG:146]
CDA96694.1   180   rubrerythrin [Prevotella sp. CAG:1320]
WP_055276667.1 180   rubrerythrin [Turicibacter sanguinis]
WP_048816914.1 180   Rubrerythrin-2 [Caldisphaera lagunensis]
WP_004844365.1 180   rubrerythrin [[Ruminococcus] gnavus]

WP_005330431.1	180	rubrerythrin [Dorea formicigenerans]
CCZ84644.1	180	putative uncharacterized protein [Ruminococcus sp.CAG:254]
WP_004345237.1	180	rubrerythrin [Prevotella buccae]
WP_004052304.1	180	hypothetical protein [Eubacterium plexicaudatum]
CDA65604.1	180	rubrerythrin [Prevotella copri CAG:164]
WP_028129999.1	180	rubrerythrin [Selenomonas sp. AE3005]
WP_005426680.1	180	rubrerythrin [Blautia obeum]
WP_007411192.1	180	rubrerythrin [Prevotella sp. MSX73]
CDC36998.1	180	rubrerythrin [Butyrivibrio sp. CAG:318]
CDE26198.1	180	rubrerythrin [Clostridium sp. CAG:440]
CDE88199.1	180	rubrerythrin [Clostridium sp. CAG:729]
WP_021686841.1	180	rubrerythrin [Treponema lecithinolyticum]
WP_024854730.1	180	rubrerythrin [[Ruminococcus] gnavus]
WP_024989952.1	180	rubrerythrin [Prevotella albensis]
CUM99384.1	180	NADH peroxidase [Eubacterium ramulus]
CDD20032.1	180	rubrerythrin [Prevotella sp. CAG:732]
WP_005947927.1	180	rubrerythrin [Blautia hydrogenotrophica]
WP_004340028.1	180	MULTISPECIES: rubrerythrin [Prevotella]
WP_046443863.1	180	rubrerythrin [Catabacter hongkongensis]
CUP48988.1	180	NADH peroxidase [Prevotella copri]
WP_015712567.1	180	rubrerythrin [Treponema azotonutricium]
WP_004081645.1	180	hypothetical protein [Clostridium sp. ASF502]
CDA45641.1	180	rubrerythrin [Prevotella sp. CAG:5226]
CDE92663.1	180	rubrerythrin [Acidaminococcus sp. CAG:542]
CCX58358.1	180	putative uncharacterized protein [Blautia hydrogenotrophica CAG:147]
WP_055164335.1	180	rubrerythrin [Blautia hydrogenotrophica]
WP_055055542.1	180	rubrerythrin [Blautia obeum]
CDE60058.1	180	rubrerythrin [Fusobacterium sp. CAG:439]
WP_009237929.1	180	rubrerythrin [Prevotella sp. oral taxon 472]
CBK96931.1	180	Rubrerythrin [[[Eubacterium] siraeum 70/3]
WP_028910684.1	180	rubrerythrin [Prevotella sp. AGR2160]
WP_033120175.1	180	rubrerythrin [Oscillibacter sp. ER4]
WP_050697209.1	180	rubrerythrin [Ruminococcaceae bacterium mt9]
WP_016524396.1	180	hypothetical protein [Treponema maltophilum]
WP_018462599.1	180	rubrerythrin [Prevotella paludivivens]
CCZ44707.1	180	putative uncharacterized protein [Bacteroides sp.CAG:545]
CDA56238.1	180	rubrerythrin [Prevotella sp. CAG:604]
WP_022270587.1	180	rubrerythrin [[Eubacterium] siraeum]
CDE41489.1	180	putative uncharacterized protein [Prevotella sp. CAG:279]
WP_026507807.1	180	rubrerythrin [Butyrivibrio sp. MC2013]
WP_029732392.1	180	rubrerythrin [Dorea sp. AGR2135]
WP_054323470.1	180	rubrerythrin [Clostridia bacterium UC5.1-1E11]
WP_006283330.1	180	rubrerythrin [Prevotella bryantii]
WP_005339393.1	180	rubrerythrin [Dorea formicigenerans]
CCX64508.1	180	rubrerythrin [Prevotella sp. CAG:1058]

WP_028513552.1	180	rubrerythrin [Ruminococcaceae bacterium AE2021]
WP_028527462.1	180	rubrerythrin [Ruminococcus gauvreauii]
WP_044923706.1	180	rubrerythrin [[Eubacterium] hallii]
WP_005355947.1	180	rubrerythrin [[Eubacterium] siraeum]
CCY66377.1	180	rubrerythrin [Prevotella sp. CAG:1124]
CCZ91768.1	180	rubredoxin [Clostridium sp. CAG:167]
WP_006849366.1	180	rubrerythrin [Prevotella copri]
WP_012938881.1	180	rubrerythrin [Acidaminococcus fermentans]
CBL22328.1	180	Rubrerythrin [Blautia obeum A2-162]
WP_055276665.1	180	rubrerythrin [Turicibacter sanguinis]
CUN11131.1	180	NADH peroxidase [[Ruminococcus] torques]
CCX86263.1	180	rubredoxin [Clostridium sp. CAG:590]
WP_031546485.1	180	rubrerythrin [Lachnospiraceae bacterium AC2014]
WP_039930887.1	180	rubrerythrin [Turicibacter sp. HGF1]
WP_044998372.1	180	rubrerythrin [Coprococcus eutactus]
WP_005336344.1	180	rubrerythrin [Dorea formicigenerans]
CDE95820.1	180	rubrerythrin [Clostridium sp. CAG:914]
WP_007888452.1	180	rubrerythrin [Roseburia inulinivorans]
WP_055305925.1	180	rubrerythrin [Turicibacter sanguinis]
WP_055156848.1	180	rubrerythrin [[Ruminococcus] torques]
WP_005946411.1	180	rubrerythrin [Faecalibacterium prausnitzii]
CCX44737.1	180	rubrerythrin [Prevotella sp. CAG:1031]
CDD59544.1	180	rubredoxin [Clostridium sp. CAG:43]
WP_022444263.1	180	rubrerythrin [Sutterellaceae bacterium ND3]
WP_016440089.1	180	hypothetical protein [Coprococcus sp. HPP0048]
WP_019188816.1	180	rubrerythrin [Prevotella conceptionensis]
CCY28800.1	180	rubrerythrin [Acholeplasma sp. CAG:878]
WP_027407491.1	180	rubrerythrin [Anaerovibrio sp. RM50]
WP_033120075.1	180	rubrerythrin [Oscillibacter sp. ER4]
WP_040551028.1	180	rubrerythrin [Pyramidobacter piscolens]
WP_044953615.1	180	rubrerythrin [Faecalibacterium prausnitzii]
WP_055242904.1	180	rubrerythrin [Turicibacter sanguinis]
WP_008393445.1	180	MULTISPECIES: rubrerythrin [Clostridiales]
WP_016281908.1	180	hypothetical protein [Lachnospiraceae bacterium A4]
CDD08618.1	180	putative uncharacterized protein [Dorea sp. CAG:317]
CDE43560.1	180	rubrerythrin [Clostridium sp. CAG:768]
WP_021583050.1	180	rubrerythrin [Prevotella pleuritidis]
WP_026766619.1	180	rubrerythrin [Selenomonas ruminantium]
WP_040763669.1	180	rubrerythrin [Turicibacter sanguinis]
WP_044938739.1	180	rubrerythrin [Pseudobutyrivibrio sp. LB2011]
WP_055164419.1	180	rubrerythrin [Turicibacter sanguinis]
WP_018968079.1	180	rubrerythrin [Prevotella loescheii]
CDC63398.1	180	rubrerythrin [Bacteroides sp. CAG:770]
WP_027397854.1	180	rubrerythrin [Anaerovibrio lipolyticus]
WP_041140090.1	180	rubrerythrin [Clostridiaceae bacterium GM1]
WP_055165155.1	180	rubrerythrin [Turicibacter sanguinis]

WP_009015965.1	180	MULTISPECIES: rubrerythrin [Acidaminococcus]
WP_013607594.1	180	rubrerythrin [Sphaerochaeta globosa]
WP_009757251.1	180	rubrerythrin [Lachnospiraceae bacterium 2_1_46FAA]
WP_007901186.1	180	rubrerythrin [Prevotella stercorea]
CCZ14280.1	180	rubrerythrin [Prevotella sp. CAG:487]
CDB43508.1	180	rubrerythrin [Firmicutes bacterium CAG:240]
WP_005604292.1	180	rubrerythrin [Butyrivibrio crossotus]
CBK75518.1	180	Rubrerythrin [Butyrivibrio fibrisolvens 16/4]
WP_028242960.1	180	rubrerythrin [Pseudobutyrivibrio ruminis]
WP_028247201.1	180	rubrerythrin [Pseudobutyrivibrio ruminis]
WP_044960666.1	180	MULTISPECIES: rubrerythrin [Shuttleworthia]
CDB34087.1	180	rubrerythrin [Eggerthella sp. CAG:209]
WP_022374410.1	180	rubrerythrin [Anaerostipes hadrus]
WP_013063889.1	180	MULTISPECIES: rubrerythrin [Prevotella]
WP_028903881.1	180	rubrerythrin [Prevotella sp. P6B4]
WP_055145842.1	180	rubrerythrin [[Ruminococcus] torques]
CCY62622.1	180	putative uncharacterized protein [Clostridium sp.CAG:967]
WP_009231859.1	180	rubrerythrin [Prevotella sp. oral taxon 317]
WP_025066484.1	180	rubrerythrin [Prevotella enoeca]
WP_033153031.1	180	rubrerythrin [Pseudobutyrivibrio ruminis]
CDB05400.1	180	rubrerythrin [Prevotella sp. CAG:520]
WP_021683597.1	180	rubrerythrin [Ruminococcus callidus]
WP_003865606.1	180	rubrerythrin [Holdemanella biformis]
WP_004223970.1	180	rubrerythrin [Blautia hansenii]
WP_011833371.1	180	rubrerythrin [Methanocorpusculum labreanum]
WP_009606318.1	180	rubrerythrin [Turicibacter sp. HGF1]
CDA96855.1	180	rubrerythrin [Bacteroides sp. CAG:709]
ACA61172.1	180	rubrerythrin [uncultured microorganism]
WP_006785031.1	180	MULTISPECIES: rubrerythrin [Turicibacter]
WP_028912494.1	180	rubrerythrin [Prevotella sp. MA2016]
CDE09223.1	180	rubrerythrin [Bacillus sp. CAG:988]
WP_006255478.1	180	rubrerythrin [Alloprevotella tannerae]
CDA33512.1	180	putative uncharacterized protein [Firmicutes bacterium CAG:536]
WP_033147746.1	180	rubrerythrin [Prevotella sp. P6B1]
WP_007572869.1	180	rubrerythrin [Prevotella multisaccharivorax]
WP_042700781.1	180	rubrerythrin [Methanocorpusculum bavaricum]
CDA23108.1	180	rubrerythrin [Mycoplasma sp. CAG:611]
CDE07802.1	180	rubrerythrin [Prevotella sp. CAG:485]
WP_025816016.1	180	rubrerythrin [Prevotella shahii]
WP_028234563.1	180	rubrerythrin [Pseudobutyrivibrio sp. MD2005]
CDE73954.1	180	rubrerythrin [Clostridium sp. CAG:451]
WP_036877086.1	180	rubrerythrin [Prevotella oryzae]
WP_055245272.1	180	rubrerythrin [Turicibacter sanguinis]
WP_055305287.1	180	rubrerythrin [Turicibacter sanguinis]
AIV03537.1	180	rubrerythrin [Candidatus Mycoplasma girerdii]
WP_044937240.1	180	rubrerythrin [Dorea sp. 5-2]
WP_055277717.1	180	rubrerythrin [Turicibacter sanguinis]

WP_007045590.1	180	rubrerythrin [Subdoligranulum variabile]
CCY80497.1	180	rubrerythrin [Prevotella sp. CAG:1185]
WP_009227720.1	180	rubrerythrin [Prevotella sp. oral taxon 299]
KSV58203.1	180	rubrerythrin [Acetivibrio ethanolgignens]
CDA13198.1	180	rubrerythrin [Anaerotruncus sp. CAG:528]
WP_027429851.1	180	rubrerythrin [Lachnospiraceae bacterium AD3010]
WP_018361423.1	180	rubrerythrin [Prevotella nanceiensis]
WP_023050766.1	180	hypothetical protein [Cetobacterium somerae]
WP_024731307.1	180	rubrerythrin [Anaerotruncus colihominis]
WP_021854154.1	180	rubrerythrin [Prevotella sp. 109]
WP_042519718.1	180	MULTISPECIES: rubrerythrin [Prevotella]
WP_010242416.1	180	rubrerythrin [Peptoniphilus rhinitidis]
WP_055230955.1	180	rubrerythrin [Roseburia hominis]
WP_014080245.1	180	rubrerythrin [Roseburia hominis]
WP_004822752.1	180	rubrerythrin [Peptoniphilus indolicus]
CDE57343.1	180	rubrerythrin [Prevotella sp. CAG:873]
WP_040784439.1	180	rubrerythrin [Marvinbryantia formatexigens]
WP_044996713.1	180	rubrerythrin [Lachnospiraceae bacterium JC7]
WP_019138267.1	180	rubrerythrin [Peptoniphilus timonensis]
WP_041251102.1	180	rubrerythrin [Filifactor alocis]
WP_019124401.1	180	rubrerythrin [Peptoniphilus grossensis]
WP_040197521.1	180	rubrerythrin [Candidatus Soleaferrea massiliensis]
WP_042398491.1	180	MULTISPECIES: rubrerythrin [Clostridium]
CDE40567.1	180	rubrerythrin [Prevotella sp. CAG:279]
CCY80176.1	180	rubrerythrin [Mycoplasma sp. CAG:877]
WP_015536279.1	180	rubrerythrin [Faecalitalea cylindroides]
CBL41149.1	180	Rubrerythrin [butyrate-producing bacterium SS3/4]
CCX48367.1	180	rubrerythrin [Bacteroides sp. CAG:927]
WP_027450343.1	180	rubrerythrin [Prevotella brevis]
WP_002842434.1	180	rubrerythrin [Finegoldia magna]
WP_021876484.1	180	rubrerythrin [Clostridium chauvoei]
WP_002835151.1	180	rubrerythrin [Finegoldia magna]
WP_019107185.1	180	rubrerythrin [Peptoniphilus senegalensis]
WP_044959900.1	180	rubrerythrin [Faecalibacterium prausnitzii]
WP_002837301.1	180	rubrerythrin [Finegoldia magna]
WP_027455395.1	180	rubrerythrin [Prevotella brevis]
CDE87859.1	180	rubrerythrin [Prevotella sp. CAG:891]
WP_032119913.1	180	rubrerythrin [Clostridium sp. CL-2]
WP_054696655.1	180	rubrerythrin [Syntrophomonas palmitatica]
WP_008680255.1	180	rubrerythrin [Clostridium sp. 7_2_43FAA]
WP_005956494.1	180	rubrerythrin [Peptoniphilus harei]
CCZ56938.1	180	rubrerythrin [Clostridium sp. CAG:762]
WP_055264493.1	180	rubrerythrin [Clostridium disporicum]
WP_016317064.1	180	hypothetical protein [Anaerotruncus sp. G3(2012)]
CBK96983.1	180	Rubrerythrin [[[Eubacterium] siraeum 70/3]
CDC48306.1	180	rubrerythrin [Eubacterium siraeum CAG:80]

WP_055142009.1 180   rubrerythrin [[Eubacterium] siraeum]
WP_036912955.1 180   rubrerythrin [Prevotella sp. FD3004]
CDC26676.1   180   rubrerythrin [Prevotella sp. CAG:386]
CBL33326.1   180   Rubrerythrin [[[Eubacterium] siraeum V10Sc8a]
WP_005356083.1 180   rubrerythrin [[Eubacterium] siraeum]
WP_007719492.1 180   rubrerythrin [[Clostridium] asparagiforme]
WP_021660980.1 180   rubrerythrin [Clostridium sp. ATCC 29733]
EKQ58172.1   180   rubrerythrin [Clostridium sp. Maddingley
MBC34-26]
WP_026886214.1 180   rubrerythrin [Clostridium beijerinckii]
WP_047000292.1 180   rubrerythrin [Clostridium sp. C8]
WP_009172524.1 180   rubrerythrin [Clostridium sp. DL-VIII]
WP_002582135.1 180   hypothetical protein [Clostridium butyricum]
WP_031202046.1 180   rubrerythrin [Firmicutes bacterium M10-2]
WP_035761793.1 180   MULTISPECIES: rubrerythrin [Clostridium]
WP_011967917.1 180   MULTISPECIES: rubrerythrin [Clostridium]
WP_043666277.1 180   rubrerythrin [Clostridium butyricum]
WP_003411771.1 180   rubrerythrin [Clostridium butyricum]
WP_026887062.1 180   reverse rubrerythrin-1 [Clostridium
beijerinckii]
WP_027637123.1 180   MULTISPECIES: rubrerythrin [Clostridium]
WP_024040320.1 180   rubrerythrin [Clostridium butyricum]
WP_023976408.1 180   reverse rubrerythrin-1 [Clostridium
pasteurianum]
WP_008679657.1 180   reverse rubrerythrin-2 [Clostridium sp.
7_2_43FAA]
WP_017211676.1 180   reverse rubrerythrin-1 [Clostridium
beijerinckii]
WP_039768546.1 180   reverse rubrerythrin-1 [Clostridium diolis]
WP_014183947.1 180   rubrerythrin [Desulfosporosinus orientis]
WP_047831183.1 180   reverse rubrerythrin-1 [Peptococcaceae
bacterium CEB3]
WP_016208759.1 180   rubrerythrin [Clostridium sartagoforme]
WP_009169613.1 180   rubrerythrin [Clostridium sp. DL-VIII]
WP_027631620.1 180   rubrerythrin [Clostridium hydrogeniformans]
WP_026889396.1 180   reverse rubrerythrin-1 [Clostridium
beijerinckii]
WP_032077930.1 180   reverse rubrerythrin-1 [Clostridium drakei]
WP_040335676.1 180   reverse rubrerythrin-1 [Candidatus
Magnetobacterium casensis]
WP_014183943.1 180   rubrerythrin [Desulfosporosinus orientis]
WP_021803118.1 180   rubredoxin/rubrerythrin [Clostridium
intestinale]
WP_018591171.1 180   reverse rubrerythrin-1 [Terrisporobacter
glycolicus]
WP_029161265.1 180   reverse rubrerythrin-1 [Clostridium
scatologenes]
WP_038673542.1 180   reverse rubrerythrin-1 [Pelosinus sp. UFO1]
WP_039680822.1 180   reverse rubrerythrin-1 [Terrisporobacter
othiniensis]

WP_013624817.1	180	rubrerythrin [Syntrophobotulus glycolicus]
WP_007061581.1	180	rubrerythrin [Clostridium carboxidivorans]
WP_013655659.1	180	rubrerythrin [Cellulosilyticum lentocellum]
WP_028129453.1	180	reverse rubrerythrin-1 [Selenomonas sp. AE3005]
WP_055277734.1	180	reverse rubrerythrin-1 [Clostridium disporicum]
WP_034440450.1	180	reverse rubrerythrin-1 [Candidatus Clostridium anorexicamassiliense]
WP_021876105.1	180	Rubredoxin/rubrerythrin [Clostridium chauvoei]
WP_011737312.1	180	rubrerythrin [Pelobacter propionicus]
WP_054740144.1	180	reverse rubrerythrin-1 [Cellulosilyticum ruminicola]
WP_016207221.1	180	MULTISPECIES: rubredoxin/rubrerythrin [Clostridium]
WP_039311276.1	180	reverse rubrerythrin-1 [Clostridium baratii]
WP_005215250.1	180	rubredoxin [Clostridium celatum]
WP_045724680.1	180	reverse rubrerythrin-1 [Clostridium baratii]
WP_055207284.1	180	reverse rubrerythrin-1 [Clostridium baratii]
WP_055275131.1	180	reverse rubrerythrin-1 [Clostridium disporicum]
WP_042393911.1	180	reverse rubrerythrin-1 [Clostridium sp. JCC]
WP_013758611.1	180	rubrerythrin [Treponema brennaborense]
CCZ51604.1	180	rubrerythrin [Acinetobacter sp. CAG:196]
WP_010254519.1	180	rubredoxin/rubrerythrin [Treponema primitia]
WP_007158001.1	180	rubrerythrin [Oribacterium sinus]
WP_005805703.1	180	MULTISPECIES: rubrerythrin/rubredoxin domain-containing protein [Candidatus Arthromitus]
WP_014094934.1	180	rubrerythrin [Candidatus Arthromitus sp. SFB-rat-Yit]
WP_029502816.1	180	reverse rubrerythrin-1 [Lachnoclostridium phytofermentans]
WP_012201811.1	180	rubrerythrin [Lachnoclostridium phytofermentans]
WP_011968748.1	180	MULTISPECIES: rubrerythrin [Clostridium]
WP_038323489.1	180	reverse rubrerythrin-1 [bacterium MS4]
WP_012425682.1	180	rubredoxin/rubrerythrin [Clostridium botulinum]
WP_012449948.1	180	rubredoxin/rubrerythrin [Clostridium botulinum]
WP_003371126.1	180	rubredoxin/rubrerythrin [Clostridium botulinum]
EKQ53045.1	180	rubrerythrin [Clostridium sp. Maddingley MBC34-26]
WP_017352816.1	180	reverse rubrerythrin-1 [Clostridium botulinum]
CDE43561.1	180	rubrerythrin Rbr2 [Clostridium sp. CAG:768]
WP_039680961.1	180	reverse rubrerythrin-1 [Terrisporobacter othiniensis]
WP_041081659.1	180	reverse rubrerythrin-1 [Clostridium botulinum]
WP_009533906.1	180	hypothetical protein [Oribacterium parvum]

WP_012058703.1 180   MULTISPECIES: rubrerythrin [Clostridium]
WP_041897109.1 180   reverse rubrerythrin-1 [Clostridium beijerinckii]
CUH92191.1   180   hypothetical protein SD1D_0640 [Herbinix sp. SD1D]
WP_006905877.1 180   MULTISPECIES: hypothetical protein [Shuttleworthia]
EGN30209.1   180   hypothetical protein HMPREF0994_06290 [Lachnospiraceae bacterium 3_1_57FAA_CT1]
WP_022747165.1 180   rbr3A: reverse rubrerythrin-1 [Clostridium saccharobutylicum]
WP_024837709.1 180   reverse rubrerythrin-1 [Clostridium sp. 12(A)]
WP_035785201.1 180   reverse rubrerythrin-1 [Clostridium botulinum]
WP_022743889.1 180   rbr3A: reverse rubrerythrin-1 [Clostridium saccharobutylicum]
WP_035148181.1 180   reverse rubrerythrin-1 [Clostridium tetanomorphum]
WP_027097122.1 180   reverse rubrerythrin-1 [Clostridium paraputrificum]
WP_002605222.1 180   rubrerythrin [Hungatella hathewayi]
WP_022030364.1 180   rubrerythrin [Hungatella hathewayi]
WP_027097123.1 180   reverse rubrerythrin-1 [Clostridium paraputrificum]
WP_012423911.1 180   rubredoxin/rubrerythrin [Clostridium botulinum]
WP_012425370.1 180   rubredoxin/rubrerythrin [Clostridium botulinum]
ERI98691.1   180   Rubrerythrin [Clostridium sp. ATCC 29733]
WP_041081473.1 180   reverse rubrerythrin-1 [Clostridium botulinum]
WP_012449600.1 180   rubredoxin/rubrerythrin [Clostridium botulinum]
WP_003369443.1 180   rubredoxin/rubrerythrin [Clostridium botulinum]
WP_009644236.1 180   MULTISPECIES: rubrerythrin domain protein[Mogibacterium]
WP_018596132.1 180   reverse rubrerythrin-1 [Blautia producta]
CDC73911.1   180   putative uncharacterized protein [Firmicutes bacterium CAG:272]
WP_022744751.1 180   rbr3B: reverse rubrerythrin-2 [Clostridium saccharobutylicum]
WP_023974650.1 180   reverse rubrerythrin-1 [Clostridium pasteurianum]
WP_024729191.1 180   reverse rubrerythrin-1 [Clostridiales bacterium VE202-09]
WP_027624120.1 180   reverse rubrerythrin-1 [Clostridium lundense]
WP_013274655.1 180   rubrerythrin [[Clostridium] saccharolyticum]
WP_026891073.1 180   reverse rubrerythrin-1 [[Clostridium] aerotolerans]
WP_012450324.1 180   rubredoxin/rubrerythrin [Clostridium botulinum]

WP_035294359.1 180 reverse rubrerythrin-1 [Clostridium sp. KNHs214]
WP_044938429.1 180 reverse rubrerythrin-1 [Blautia schinkii]
WP_006568293.1 180 MULTISPECIES: hypothetical protein [Anaerostipes]
WP_009004554.1 180 MULTISPECIES: rubrerythrin [Clostridiales]
WP_024292975.1 180 MULTISPECIES: reverse rubrerythrin-1[Lachnoclostridium]
WP_025653785.1 180 MULTISPECIES: reverse rubrerythrin-1 [Clostridiales]
WP_038280209.1 180 reverse rubrerythrin-1 [[Clostridium] celerecrescens]
WP_031475296.1 180 reverse rubrerythrin-1 [Eubacterium desmolans]
WP_025230384.1 180 MULTISPECIES: reverse rubrerythrin-1 [Clostridiales]
WP_004607608.1 180 hypothetical protein [[Clostridium] scindens]
WP_049728430.1 180 reverse rubrerythrin-1 [Dorea sp. D27]
WP_006443546.1 180 hypothetical protein [[Clostridium] hylemonae]
WP_007286714.1 180 MULTISPECIES: hypothetical protein [Clostridiales]
WP_004034056.1 180 hypothetical protein [Clostridium sp. ASF356]
WP_044994951.1 180 reverse rubrerythrin-1 [Lachnospiraceae bacterium JC7]
WP_055070305.1 180 reverse rubrerythrin-1 [Clostridium sp. ND2]
WP_055071954.1 180 reverse rubrerythrin-1 [Clostridium sp. ND2]
EQH65485.1 180 "reverse rubrerythrin-1, partial [Clostridium difficile DA00273]"
EQI93102.1 180 "reverse rubrerythrin-1, partial [Clostridium difficile P3]"
WP_027626947.1 180 reverse rubrerythrin-1 [Terrisporobacter glycolicus]
WP_055259105.1 180 reverse rubrerythrin-1 [Sarcina ventriculi]
WP_055087547.1 180 reverse rubrerythrin-1 [Intestinibacter bartlettii]
WP_003475303.1 180 rubredoxin/rubrerythrin [Clostridium perfringens]
EQJ36708.1 180 "reverse rubrerythrin-1, partial [Clostridium difficile P21]"
WP_054260703.1 180 reverse rubrerythrin-1 [Propionispora sp. Iso2/2]
WP_007286712.1 180 MULTISPECIES: hypothetical protein [Clostridiales]
ETI94334.1 180 hypothetical protein Q606_CBAC00293G0002 [Intestinibacter bartlettii DORA_8_9]
WP_003459925.1 180 rubredoxin/rubrerythrin [Clostridium perfringens]
WP_002598391.1 180 reverse rubrerythrin-1 [Clostridium colicanis]
WP_006439158.1 180 hypothetical protein [[Clostridium] hiranonis]
WP_003472992.1 180 rubredoxin/rubrerythrin [Clostridium perfringens]

WP_054199186.1 180   reverse rubrerythrin-1 [Clostridium baratii]
WP_011591188.1 180   rubredoxin/rubrerythrin [Clostridium perfringens]
WP_003479396.1 180   reverse rubrerythrin-1 [Clostridium perfringens]
WP_039310885.1 180   reverse rubrerythrin-1 [Clostridium baratii]
WP_002598390.1 180   reverse rubrerythrin-1 [Clostridium colicanis]
WP_003457010.1 180   rubredoxin/rubrerythrin [Clostridium perfringens]
ABG86148.1   180   rubredoxin/rubrerythrin [Clostridium perfringens SM101]
CCX79790.1   180   rubrerythrin [Clostridium sp. CAG:715]
WP_024615677.1 180   reverse rubrerythrin-1 [Clostridium sp. Ade.TY]
WP_003453016.1 180   rubredoxin/rubrerythrin [Clostridium perfringens]
WP_031392604.1 180   hypothetical protein [Clostridium sp. KNHs209]
CDE62001.1   180   rubrerythrin [Fusobacterium sp. CAG:439]
CCY24709.1   180   putative uncharacterized protein [Brachyspira sp.CAG:484]
CDE44386.1   180   rubrerythrin [Clostridium sp. CAG:768]
CCY62119.1   180   rubrerythrin [Clostridium sp. CAG:967]
WP_027295613.1 180   MULTISPECIES: reverse rubrerythrin-1 [Robinsoniella]
CRZ34813.1   180   hypothetical protein HHT355_1612 [Herbinix hemicellulosilytica]
CDC20975.1   180   putative rubperoxin [Eubacterium sp. CAG:274]
WP_031474774.1 180   reverse rubrerythrin-1 [[Clostridium] aminophilum]
WP_013702649.1 180   rubrerythrin [Treponema succinifaciens]
WP_021168944.1 180   MULTISPECIES: rubrerythrin [Sporomusa]
WP_055259103.1 180   reverse rubrerythrin-1 [Sarcina ventriculi]
WP_021658517.1 180   rubredoxin [Clostridium sp. ATCC 29733]
WP_027869407.1 180   reverse rubrerythrin-1 [Eubacterium sp. AB3007]
CCY53565.1   180   rubrerythrin [Coprococcus sp. CAG:782]
CDC02442.1   180   rubrerythrin [Eubacterium sp. CAG:202]
WP_023345662.1 180   hypothetical protein [Firmicutes bacterium ASF500]
WP_033164382.1 180   reverse rubrerythrin-1 [Clostridium sp. KNHs205]
WP_016320693.1 180   hypothetical protein [Oscillibacter sp. 1-3]
WP_007960924.1 180   rubrerythrin [Pelosinus fermentans]
WP_044923016.1 180   reverse rubrerythrin-1 [Lachnospiraceae bacterium MA2020]
WP_027115603.1 180   reverse rubrerythrin-1 [Lachnospiraceae bacterium P6B14]
WP_035642797.1 180   reverse rubrerythrin-1 [Lachnospiraceae bacterium FE2018]
WP_004844185.1 180   hypothetical protein [[Ruminococcus] gnavus]

WP_022781136.1 180   MULTISPECIES: reverse rubrerythrin-1
[unclassified Lachnospiraceae]
WP_008400622.1 180   hypothetical protein [Clostridium sp. L2-50]
WP_005334966.1 180   hypothetical protein [Dorea formicigenerans]
CDD06940.1   180   putative uncharacterized protein [Dorea sp.
CAG:317]
CBK79685.1   180   Rubrerythrin [Coprococcus catus GD/7]
CBK81751.1   180   Rubrerythrin [Coprococcus catus GD/7]
WP_015527602.1 180   rubrerythrin [Ruminococcus faecis]
WP_031391847.1 180   reverse rubrerythrin-1 [Clostridium sp.
KNHs209]
WP_016306394.1 180   hypothetical protein [Lachnospiraceae
bacterium A2]
CCY59897.1   180   rubredoxin [Clostridium sp. CAG:264]
WP_055145060.1 180   reverse rubrerythrin-1 [[Ruminococcus]
torques]
WP_055158586.1 180   reverse rubrerythrin-1 [[Ruminococcus]
torques]
WP_004847244.1 180   hypothetical protein [[Ruminococcus] torques]
CCZ94661.1   180   putative uncharacterized protein
[Corallococcus sp.CAG:1435]
CDF14908.1   180   rubrerythrin [Eubacterium sp. CAG:581]
WP_026512544.1 180   MULTISPECIES: reverse rubrerythrin-1
[Butyrivibrio]
KSV58549.1   180   Reverse rubrerythrin-1 [Acetivibrio
ethanolgignens]
WP_005341890.1 180   MULTISPECIES: hypothetical protein [Dorea]
WP_016216335.1 180   hypothetical protein [Eubacterium sp. 14-2]
CDD54723.1   180   putative uncharacterized protein [Clostridium
sp. CAG:43]
WP_006857073.1 180   rubredoxin/rubrerythrin [Roseburia
intestinalis]
WP_006571398.1 180   hypothetical protein [Pseudoflavonifractor
capillosus]
CDC39782.1   180   rubrerythrin [Butyrivibrio sp. CAG:318]
WP_034239731.1 180   reverse rubrerythrin-1 [Lachnospiraceae
bacterium 28-4]
WP_055066443.1 180   reverse rubrerythrin-1 [Blautia obeum]
WP_007049679.1 180   hypothetical protein [Anaerofustis
stercorihominis]
WP_007493606.1 180   MULTISPECIES: rubredoxin [Clostridiales]
WP_016285675.1 180   hypothetical protein [Lachnospiraceae
bacterium 3-1]
ERI72828.1   180   rubredoxin [Clostridium sp. KLE 1755]
WP_005608304.1 180   hypothetical protein [Ruminococcus lactaris]
WP_055257718.1 180   reverse rubrerythrin-1 [Sarcina ventriculi]
WP_055055811.1 180   reverse rubrerythrin-1 [Blautia obeum]
CDB19579.1   180   putative uncharacterized protein [Blautia sp.
CAG:52]
CDE71449.1   180   putative uncharacterized protein

[Subdoligranulum sp.CAG:314]
WP_031272092.1 180   reverse rubrerythrin-1 [Lachnospiraceae bacterium 10-1]
WP_015394341.1 180   rubrerythrin [Clostridium saccharoperbutylacetonicum]
CDA97790.1   180   rubrerythrin [Firmicutes bacterium CAG:65]
WP_035633883.1 180   reverse rubrerythrin-1 [Lachnospiraceae bacterium ND2006]
WP_008981661.1 180   rubrerythrin [Ruminococcaceae bacterium D16]
WP_006782397.1 180   hypothetical protein [Hungatella hathewayi]
WP_009325791.1 180   hypothetical protein [Subdoligranulum sp. 4_3_54A2FAA]
WP_002572966.1 180   "rubrerythrin, partial [[Clostridium] bolteae]"
CBL39948.1   180   Rubrerythrin [butyrate-producing bacterium SS3/4]
WP_028527707.1 180   reverse rubrerythrin-1 [Ruminococcus gauvreauii]
WP_029543591.1 180   rubrerythrin [Selenomonas ruminantium]
WP_040784386.1 180   reverse rubrerythrin-1 [Marvinbryantia formatexigens]
WP_004081700.1 180   hypothetical protein [Clostridium sp. ASF502]
WP_016224421.1 180   hypothetical protein [Lachnospiraceae bacterium 3-2]
CCY11067.1   180   putative uncharacterized protein [Clostridium sp. CAG:81]
WP_021738063.1 180   rubredoxin [Eubacterium ramulus]
CDC45507.1   180   putative uncharacterized protein [Clostridium sp. CAG:58]
WP_008397676.1 180   rubredoxin/rubrerythrin [Clostridium sp. M62/1]
WP_033117356.1 180   reverse rubrerythrin-1 [Intestinimonas butyriciproducens]
WP_006427230.1 180   hypothetical protein [Dorea longicatena]
WP_004058922.1 180   hypothetical protein [Eubacterium plexicaudatum]
WP_016219697.1 180   hypothetical protein [Dorea sp. 5-2]
CCZ34857.1   180   putative uncharacterized protein [Firmicutes bacterium CAG:646]
CDA05093.1   180   putative uncharacterized protein [Blautia sp. CAG:257]
CDA13727.1   180   putative uncharacterized protein [Firmicutes bacterium CAG:212]
CDD46860.1   180   rubrerythrin [Firmicutes bacterium CAG:534]
WP_005601917.1 180   rubredoxin/rubrerythrin [Butyrivibrio crossotus]
WP_024733892.1 180   reverse rubrerythrin-1 [Clostridium sp. BR72]
WP_054334444.1 180   reverse rubrerythrin-1 [Clostridia bacterium UC5.1-2E3]
WP_003415194.1 180   MULTISPECIES: rubrerythrin [Clostridium]

WP_009270851.1 180   MULTISPECIES: hypothetical protein
[Clostridiales]
WP_014080957.1 180   ferredoxin hydrogenase [Roseburia hominis]
WP_003424172.1 180   rubrerythrin [Clostridium butyricum]
WP_016298041.1 180   hypothetical protein [Lachnospiraceae
bacterium M18-1]
CDE72247.1   180   putative uncharacterized protein
[Acidaminococcus sp.CAG:917]
WP_003021715.1 180   rubredoxin/rubrerythrin [Blautia hansenii]
WP_028086555.1 180   reverse rubrerythrin-1 [Dorea longicatena]
WP_033141649.1 180   reverse rubrerythrin-1 [Blautia producta]
WP_046057937.1 180   reverse rubrerythrin-1 [Clostridium sp.
IBUN62F]
CUP75267.1   180   NADH peroxidase [Flavonifractor plautii]
CUQ40634.1   180   rubrerythrin [Flavonifractor plautii]
WP_009272336.1 180   rubrerythrin [Erysipelotrichaceae bacterium
3_1_53]
WP_009267060.1 180   hypothetical protein
[[Clostridium]glycyrrhizinilyticum]
WP_014430969.1 180   rubrerythrin [Selenomonas ruminantium]
WP_019163241.1 180   reverse rubrerythrin-1 [Ruminococcus sp.
JC304]
CCZ91739.1   180   rubrerythrin [Clostridium sp. CAG:167]
CDC11915.1   180   rubrerythrin [Roseburia sp. CAG:45]
CDC42140.1   180   putative uncharacterized protein [Firmicutes
bacterium CAG:424]
WP_050617003.1 180   reverse rubrerythrin-1 [Intestinimonas sp.
GD2]
WP_054327319.1 180   reverse rubrerythrin-1 [Clostridia bacterium
UC5.1-1D1]
WP_002580857.1 180   rubrerythrin [Clostridium butyricum]
WP_002579108.1 180   rubrerythrin [Clostridium butyricum]
CDB14181.1   180   putative uncharacterized protein [Clostridium
sp.CAG:221]
CDB75905.1   180   putative uncharacterized protein [Clostridium
sp.CAG:265]
CDD45254.1   180   putative uncharacterized protein [Clostridium
sp.CAG:299]
WP_003415710.1 180   rubrerythrin [Clostridium butyricum]
WP_008817908.1 180   hypothetical protein [[Clostridium] innocuum]
CDF43599.1   180   putative uncharacterized protein [Roseburia
sp. CAG:182]
WP_004613190.1 180   hypothetical protein [Tyzzerella nexilis]
WP_003021709.1 180   rubredoxin/rubrerythrin [Blautia hansenii]
WP_033126120.1 180   reverse rubrerythrin-1 [Eubacterium sp. ER2]
WP_044938435.1 180   reverse rubrerythrin-1 [Blautia schinkii]
WP_005952475.1 180   hypothetical protein [Blautia
hydrogenotrophica]
WP_029201912.1 180   reverse rubrerythrin-1 [Oribacterium sp.
NK2B42]

WP_009461850.1 180   hypothetical protein [Lachnospiraceae bacterium 2_1_46FAA]
WP_002609625.1 180   hypothetical protein [[Clostridium] innocuum]
CUQ48639.1   180   NADH peroxidase [[Ruminococcus] torques]
WP_007286718.1 180   MULTISPECIES: hypothetical protein [Clostridiales]
WP_008977195.1 180   MULTISPECIES: hypothetical protein [Coprococcus]
ETI95472.1   180   hypothetical protein Q606_CBAC00186G0004 [Intestinibacter bartlettii DORA_8_9]
WP_022045230.1 180   hypothetical protein [Roseburia faecis]
WP_026658118.1 180   reverse rubrerythrin-1 [Butyrivibrio sp. AC2005]
WP_031548301.1 180   reverse rubrerythrin-1 [Oribacterium sp. FC2011]
WP_006568456.1 180   MULTISPECIES: hypothetical protein [Anaerostipes]
CCX75378.1   180   putative uncharacterized protein [Dorea sp. CAG:105]
WP_036607067.1 180   reverse rubrerythrin-1 [Oribacterium sp. P6A1]
WP_032120010.1 180   reverse rubrerythrin-1 [Clostridium sp. CL-2]
WP_029323727.1 180   reverse rubrerythrin-1 [Butyrivibrio sp. AE3004]
WP_042256255.1 180   reverse rubrerythrin-1 [Butyrivibrio proteoclasticus]
WP_032120009.1 180   reverse rubrerythrin-1 [Clostridium sp. CL-2]
WP_003538430.1 180   MULTISPECIES: hypothetical protein[Erysipelotrichaceae]
CDE55747.1   180   putative uncharacterized protein [Roseburia sp. CAG:303]
CDC92153.1   180   rubrerythrin [Roseburia sp. CAG:380]
CDE69531.1   180   rubrerythrin [Clostridium sp. CAG:277]
WP_026663051.1 180   reverse rubrerythrin-1 [Butyrivibrio proteoclasticus]
CDD74623.1   180   rubrerythrin [Clostridium sp. CAG:62]
WP_024866097.1 180   reverse rubrerythrin-1 [Butyrivibrio sp. FCS014]
WP_027295617.1 180   MULTISPECIES: reverse rubrerythrin-1 [Robinsoniella]
WP_022764947.1 180   reverse rubrerythrin-1 [Butyrivibrio sp. XPD2006]
WP_027295615.1 180   MULTISPECIES: reverse rubrerythrin-1 [Robinsoniella]
CDB03151.1   180   putative uncharacterized protein [Firmicutes bacterium CAG:145]
CDE46599.1   180   putative uncharacterized protein [Faecalibacterium sp.CAG:74]
CBL21751.1   180   Rubrerythrin [Blautia obeum A2-162]
WP_055283308.1 180   reverse rubrerythrin-1 [Dorea longicatena]
WP_005345674.1 180   hypothetical protein [[Eubacterium] hallii]

WP_029231437.1	180	reverse rubrerythrin-1 [Butyrivibrio sp. VCB2006]
AIU44562.1	180	hypothetical protein [Cyanophora paradoxa]
NP_043245.1	180	hypothetical protein CypaCp108 [Cyanophora paradoxa]
WP_015492567.1	179	rubrerythrin [Thermoplasmatales archaeon BRNA1]
WP_011016402.1	179	rubrerythrin [Fusobacterium nucleatum]
WP_032885008.1	179	rubrerythrin [Fusobacterium nucleatum]
WP_023041007.1	179	hypothetical protein [Fusobacterium nucleatum]
WP_029598567.1	179	rubrerythrin [Fusobacterium nucleatum]
CDD26900.1	179	rubrerythrin [Clostridium sp. CAG:452]
CCY16327.1	179	rubrerythrin [Prevotella sp. CAG:755]
CDE96741.1	179	rubrerythrin [Clostridium sp. CAG:567]
CCY68912.1	179	putative uncharacterized protein [Eubacterium sp.CAG:161]
CCX52771.1	179	rubrerythrin [Alistipes sp. CAG:514]
WP_024834200.1	179	rubrerythrin [[Clostridium] josui]
WP_033164920.1	179	rubrerythrin [Clostridium sp. KNHs205]
WP_016146734.1	179	hypothetical protein [Butyricicoccus pullicaecorum]
CDA80134.1	179	rubrerythrin [Clostridium sp. CAG:594]
WP_013739571.1	179	rubrerythrin [Sphaerochaeta coccoides]
WP_014313755.1	179	rubrerythrin [Clostridium sp. BNL1100]
CCX38408.1	179	rubrerythrin [Clostridium sp. CAG:1013]
WP_020814596.1	179	rubrerythrin [[Clostridium] papyrosolvens]
WP_016285249.1	179	hypothetical protein [Lachnospiraceae bacterium 3-1]
WP_021329607.1	179	rubrerythrin [Treponema socranskii]
WP_015925322.1	179	rubrerythrin [[Clostridium] cellulolyticum]
WP_029502037.1	179	rubrerythrin [Lachnoclostridium phytofermentans]
WP_005965799.1	179	rubrerythrin [Fusobacterium periodonticum]
WP_009106999.1	179	rubrerythrin [Treponema sp. JC4]
WP_009139071.1	179	rubrerythrin [Slackia piriformis]
CCY05220.1	179	rubrerythrin [Eggerthella sp. CAG:1427]
CDD17938.1	179	rubrerythrin [Clostridium sp. CAG:798]
CFX22868.1	179	Rubrerythrin [Syntrophomonas zehnderi OL-4]
WP_013709461.1	179	rubrerythrin [Coriobacterium glomerans]
WP_016520958.1	179	hypothetical protein [Treponema socranskii]
WP_013758159.1	179	rubrerythrin [Treponema brennaborense]
CDB31680.1	179	rubrerythrin [Clostridium sp. CAG:575]
WP_005971325.1	179	rubrerythrin [Fusobacterium periodonticum]
WP_027728244.1	179	rubrerythrin [Treponema sp. C6A8]
CCX49448.1	179	rubrerythrin [Clostridium sp. CAG:226]
WP_029494302.1	179	rubrerythrin [Fusobacterium hwasookii]
WP_012803245.1	179	rubrerythrin [Cryptobacterium curtum]
WP_016316506.1	179	hypothetical protein [Anaerotruncus sp. G3(2012)]
CCZ59769.1	179	rubrerythrin [Clostridium sp. CAG:710]

CDD23466.1   179   rubredoxin [Firmicutes bacterium CAG:345]
CDE77866.1   179   rubrerythrin [Ruminococcus sp. CAG:353]
WP_006568167.1 179   MULTISPECIES: rubrerythrin [Anaerostipes]
WP_009256007.1 179   MULTISPECIES: rubrerythrin [Clostridiales]
WP_005915662.1 179   rubrerythrin [Fusobacterium hwasookii]
WP_016213828.1 179   hypothetical protein [Eubacterium sp. 14-2]
WP_024729110.1 179   rubrerythrin [Clostridiales bacterium
VE202-09]
CDC06833.1   179   rubrerythrin [Clostridium sp. CAG:343]
WP_020223651.1 179   rubrerythrin [Holdemania massiliensis]
WP_044971122.1 179   rubrerythrin [Clostridiaceae bacterium MS3]
CDA30159.1   179   rubrerythrin [Clostridium sp. CAG:492]
CDD46883.1   179   putative uncharacterized protein [Firmicutes
bacterium CAG:534]
CDF30376.1   179   rubrerythrin [Methanoculleus sp. CAG:1088]
WP_029494025.1 179   rubrerythrin [Fusobacterium hwasookii]
WP_035624971.1 179   rubrerythrin [Lachnospiraceae bacterium C6A11]
WP_012798419.1 179   rubrerythrin [Slackia heliotrinireducens]
CDA52928.1   179   rubrerythrin [Clostridium sp. CAG:533]
CDB13165.1   179   rubredoxin [Eubacterium sp. CAG:192]
WP_021635601.1 179   rubrerythrin [Clostridium sp. KLE 1755]
WP_006059148.1 179   rubrerythrin [Holdemania filiformis]
WP_027097943.1 179   rubrerythrin [Clostridium paraputrificum]
CCY88063.1   179   rubrerythrin [Mycoplasma sp. CAG:956]
CDE15165.1   179   rubrerythrin [Clostridium sp. CAG:470]
CDF00280.1   179   rubrerythrin [Clostridium sp. CAG:813]
WP_031392778.1 179   rubrerythrin [Clostridium sp. KNHs209]
WP_035770283.1 179   rubrerythrin [Clostridium cadaveris]
WP_022000948.1 179   rubredoxin [Coprobacillus sp. 8_1_38FAA]
CCZ56623.1   179   putative uncharacterized protein [Clostridium
sp.CAG:1219]
CDB41319.1   179   putative uncharacterized protein [Ruminococcus
sp.CAG:177]
CDD29453.1   179   rubrerythrin [Firmicutes bacterium CAG:94]
CDE15939.1   179   rubrerythrin [Clostridium sp. CAG:288]
CDE50453.1   179   rubrerythrin [Firmicutes bacterium CAG:460]
AHF24785.1   179   rubrerythrin [uncultured bacterium Contig1495]
WP_031271282.1 179   rubrerythrin [Lachnospiraceae bacterium 10-1]
WP_054326817.1 179   rubrerythrin [Clostridia bacterium UC5.1-1D1]
CDF20101.1   179   rubrerythrin [Clostridium sp. CAG:609]
WP_012198555.1 179   rubrerythrin [Lachnoclostridium
phytofermentans]
WP_016300031.1 179   hypothetical protein [Lachnospiraceae
bacterium COE1]
CDB28274.1   179   rubrerythrin [Firmicutes bacterium CAG:582]
WP_049179063.1 179   rubrerythrin [Clostridium botulinum]
WP_010680630.1 179   rubrerythrin [Fusobacterium gonidiaformans]
CDD17291.1   179   rubrerythrin [Alistipes sp. CAG:435]
CDD27935.1   179   rubrerythrin [Clostridium sp. CAG:433]
WP_041612062.1 179   rubrerythrin [Treponema succinifaciens]

WP_025435115.1 179   rubrerythrin [Eubacterium acidaminophilum]
WP_029471982.1 179   rubrerythrin [Clostridiales bacterium VE202-08]
WP_048097814.1 179   rubrerythrin [Candidatus Methanomethylophilus alvus]
WP_012423529.1 179   rubrerythrin [Clostridium botulinum]
WP_016290007.1 179   hypothetical protein [Lachnospiraceae bacterium 28-4]
CDE05263.1   179   putative uncharacterized protein [Anaerotruncus sp.CAG:390]
WP_022939357.1 179   rubrerythrin [Dielma fastidiosa]
WP_014271628.1 179   rubrerythrin [Sphaerochaeta pleomorpha]
WP_022250578.1 179   hypothetical protein [Tyzzerella nexilis]
CDD68090.1   179   rubrerythrin [Eggerthella sp. CAG:368]
WP_038326154.1 179   rubrerythrin [bacterium MS4]
WP_003373915.1 179   rubrerythrin [Clostridium botulinum]
WP_008800844.1 179   rubrerythrin [Fusobacterium gonidiaformans]
WP_004038239.1 179   hypothetical protein [Clostridium sp. ASF356]
CDC31478.1   179   rubrerythrin [Clostridium sp. CAG:508]
WP_012820339.1 179   rubrerythrin [Fibrobacter succinogenes]
EFQ07190.1   179   Rubrerythrin [Faecalibacterium cf. prausnitzii KLE1255]
WP_029507115.1 179   rubrerythrin [Lachnospiraceae bacterium AC2012]
WP_008701223.1 179   rubrerythrin [Fusobacterium nucleatum]
WP_008810906.1 179   rubrerythrin [Parasutterella excrementihominis]
CCZ88890.1   179   rubrerythrin [Coprobacillus sp. CAG:605]
WP_009141211.1 179   rubrerythrin [Collinsella tanakaei]
CCY99736.1   179   rubrerythrin [Clostridium sp. CAG:793]
WP_035136761.1 179   rubrerythrin [Collinsella sp. 4_8_47FAA]
WP_055252097.1 179   rubrerythrin [Collinsella aerofaciens]
WP_008795242.1 179   rubrerythrin [Fusobacterium nucleatum]
WP_014423549.1 179   rubrerythrin [Selenomonas ruminantium]
WP_020072755.1 179   rubrerythrin [[Clostridium] sporosphaeroides]
EUB13476.1   179   rubrerythrin [Shuttleworthia sp. MSX8B]
WP_035133587.1 179   rubrerythrin [Clostridium sulfidigenes]
WP_050699362.1 179   rubrerythrin [Ruminococcaceae bacterium mt9]
WP_054324477.1 179   rubrerythrin [Clostridia bacterium UC5.1-1E11]
WP_004847609.1 179   rubrerythrin [[Ruminococcus] torques]
WP_029758570.1 179   rubrerythrin [Fusobacterium nucleatum]
WP_032509693.1 179   rubrerythrin [Peptoclostridium difficile]
WP_009606327.1 179   MULTISPECIES: rubrerythrin [Turicibacter]
WP_009324580.1 179   rubrerythrin [Subdoligranulum sp. 4_3_54A2FAA]
WP_027433757.1 179   MULTISPECIES: rubrerythrin [unclassified Lachnospiraceae]
WP_004617814.1 179   rubrerythrin [[Clostridium] papyrosolvens]
WP_009059691.1 179   rubrerythrin [Clostridium sp. MSTE9]
CCZ50691.1   179   putative uncharacterized protein [Acinetobacter sp.CAG:196]

WP_035394827.1	179	rubrerythrin [Faecalibacterium prausnitzii]
CDB98419.1	179	rubrerythrin [Firmicutes bacterium CAG:41]
CDE13265.1	179	putative uncharacterized protein [Ruminococcus sp.CAG:330]
WP_032506536.1	179	MULTISPECIES: rubrerythrin [Peptoclostridium]
WP_054272783.1	179	rubrerythrin [Peptoclostridium difficile]
WP_055285276.1	179	rubrerythrin [Collinsella aerofaciens]
WP_006235152.1	179	rubrerythrin [Collinsella aerofaciens]
WP_005981983.1	179	rubrerythrin [Fusobacterium ulcerans]
WP_006723314.1	179	rubrerythrin [Collinsella intestinalis]
WP_042433776.1	179	rubrerythrin [Clostridium sp. JCD]
WP_054269479.1	179	rubrerythrin [Peptoclostridium difficile]
WP_019190075.1	179	rubrerythrin [Levyella massiliensis]
CCZ85298.1	179	rubrerythrin [Firmicutes bacterium CAG:631]
CBK99869.1	179	Rubrerythrin [Faecalibacterium prausnitzii L2-6]
WP_026835434.1	179	rubrerythrin [Eubacterium xylanophilum]
WP_028505733.1	179	rubrerythrin [Ruminococcus sp. FC2018]
WP_032514434.1	179	rubrerythrin [Peptoclostridium difficile]
WP_014827598.1	179	rubrerythrin [Desulfosporosinus acidiphilus]
CCY94221.1	179	rubrerythrin [Firmicutes bacterium CAG:884]
CDD36467.1	179	rubredoxin [Clostridium sp. CAG:356]
WP_026759895.1	179	MULTISPECIES: rubrerythrin [Selenomonas]
WP_032521007.1	179	rubrerythrin [Peptoclostridium difficile]
WP_019239403.1	179	rubrerythrin [Collinsella sp. GD3]
CDD54936.1	179	rubredoxin [Clostridium sp. CAG:43]
WP_008797604.1	179	rubrerythrin [Fusobacterium nucleatum]
WP_037278365.1	179	rubrerythrin [Ruminococcaceae bacterium AB4001]
WP_005951127.1	179	rubrerythrin [Fusobacterium varium]
CDB39237.1	179	rubrerythrin [Azospirillum sp. CAG:260]
CDE96735.1	179	rubredoxin [Clostridium sp. CAG:567]
CDF22420.1	179	rubrerythrin [Prevotella sp. CAG:617]
WP_023035965.1	179	MULTISPECIES: hypothetical protein [Fusobacterium]
WP_039633107.1	179	rubrerythrin [Clostridium argentinense]
WP_044212878.1	179	rubrerythrin [Peptoclostridium difficile]
WP_005884352.1	179	rubrerythrin [Fusobacterium mortiferum]
CDC13048.1	179	rubrerythrin [Clostridium sp. CAG:413]
CDC18311.1	179	rubrerythrin [Clostridium sp. CAG:306]
WP_032845550.1	179	rubrerythrin [Fusobacterium sp. CM22]
WP_010167086.1	179	rubrerythrin [Epulopiscium sp. 'N.t. morphotype B']
WP_016224335.1	179	hypothetical protein [Lachnospiraceae bacterium 3-2]
CCX42296.1	179	rubrerythrin [Clostridium sp. CAG:1024]
CDA07767.1	179	rubrerythrin [Fusobacterium sp. CAG:649]
WP_048111754.1	179	rubrerythrin [Candidatus Methanoplasma termitum]
WP_044950613.1	179	rubrerythrin [Lachnospiraceae bacterium

YSB2008]

WP_008802455.1	179	rubrerythrin [Fusobacterium nucleatum]
CCY27208.1	179	putative uncharacterized protein [Acholeplasma sp.CAG:878]
CDA45609.1	179	rubrerythrin [Proteobacteria bacterium CAG:139]
CDE39314.1	179	rubrerythrin [Firmicutes bacterium CAG:321]
WP_032834733.1	179	MULTISPECIES: rubrerythrin [Fusobacterium]
EOS79066.1	179	hypothetical protein C817_03134 [Dorea sp. 5-2]
WP_019128718.1	179	rubrerythrin [Enorma massiliensis]
CCZ29892.1	179	rubrerythrin [Proteobacteria bacterium CAG:495]
CDB50900.1	179	putative uncharacterized protein [Clostridium sp.CAG:217]
CDC27376.1	179	rubrerythrin [Faecalibacterium sp. CAG:82]
CDF45177.1	179	putative uncharacterized protein [Roseburia sp. CAG:100]
WP_031556588.1	179	rubrerythrin [Lachnospira multipara]
WP_041921225.1	179	rubrerythrin [Ilyobacter polytropus]
WP_009271666.1	179	rubrerythrin [Erysipelotrichaceae bacterium 3_1_53]
WP_020309941.1	179	MULTISPECIES: rubredoxin [Megasphaera]
CDB92462.1	179	putative uncharacterized protein [Clostridium sp.CAG:302]
CDF12085.1	179	rubrerythrin [Mycoplasma sp. CAG:776]
WP_006720705.1	179	rubrerythrin [Collinsella stercoris]
WP_044505417.1	179	rubrerythrin [Megasphaera massiliensis]
WP_054332540.1	179	rubrerythrin [Clostridia bacterium UC5.1-2F7]
WP_005898309.1	179	rubrerythrin [Fusobacterium nucleatum]
CDB53520.1	179	rubrerythrin [Azospirillum sp. CAG:239]
CDE55637.1	179	rubredoxin [Clostridium sp. CAG:269]
WP_023275240.1	179	hypothetical protein [Mucispirillum schaedleri]
WP_006626708.1	179	rubrerythrin [Bulleidia extructa]
WP_035642193.1	179	rubrerythrin [Lachnospiraceae bacterium FE2018]
WP_037329196.1	179	rubrerythrin [Ruminococcus flavefaciens]
WP_034210070.1	179	rubrerythrin [Lachnospira multipara]
WP_009423578.1	179	rubrerythrin [Fusobacterium sp. oral taxon 370]
WP_020225882.1	179	rubrerythrin [Holdemania massiliensis]
WP_002606495.1	179	MULTISPECIES: rubrerythrin [Firmicutes]
WP_014015207.1	179	rubrerythrin [Megasphaera elsdenii]
CDC61199.1	179	putative uncharacterized protein [Clostridium sp.CAG:417]
CDE97313.1	179	rubredoxin [Clostridium sp. CAG:567]
WP_023036631.1	179	hypothetical protein [Fusobacterium nucleatum]
WP_008691128.1	179	rubrerythrin [Eubacterium sp. 3_1_31]
WP_011010075.1	179	rubrerythrin [Clostridium perfringens]

WP_003477208.1 179   rubrerythrin [Clostridium perfringens]
WP_003465844.1 179   rubrerythrin [Clostridium perfringens]
WP_003449244.1 179   rubrerythrin [Clostridium perfringens]
CDA17856.1   179   rubrerythrin [Acetobacter sp. CAG:267]
WP_057257921.1 179   rubrerythrin [Clostridium perfringens]
CDE95038.1   179   putative uncharacterized protein [Clostridium sp.CAG:914]
WP_038353045.1 179   rubrerythrin [Eubacterium limosum]
WP_006059979.1 179   rubrerythrin [Holdemania filiformis]
WP_008693574.1 179   rubrerythrin [Fusobacterium nucleatum]
WP_029491128.1 179   rubrerythrin [Fusobacterium hwasookii]
WP_013382069.1 179   rubrerythrin [Eubacterium limosum]
WP_022420003.1 179   rubrerythrin [[Eubacterium] dolichum]
WP_015535124.1 179   rubrerythrin [Faecalitalea cylindroides]
WP_036202445.1 179   rubrerythrin [Megasphaera elsdenii]
CDE98746.1   179   rubrerythrin [Clostridium sp. CAG:628]
WP_024730724.1 179   rubrerythrin [Anaerotruncus colihominis]
ABI67997.1   179   rubrerythrin [Syntrophomonas wolfei subsp. wolfei str.Goettingen G311]
WP_040467432.1 179   rubrerythrin [Eubacterium brachy]
ALG48231.1   179   Rubrerythrin [Clostridium perfringens]
CDE37650.1   179   rubrerythrin [Mycoplasma sp. CAG:472]
ALF18353.1   179   rubrerythrin [Fusobacterium nucleatum subsp. animalis]
CCX65357.1   179   putative uncharacterized protein [Firmicutes bacterium CAG:791]
CDA60181.1   179   rubrerythrin [Clostridium sp. CAG:524]
WP_005911088.1 179   MULTISPECIES: rubrerythrin [Fusobacterium]
WP_008799165.1 179   rubrerythrin [Fusobacterium nucleatum]
WP_018590403.1 179   rubrerythrin [Terrisporobacter glycolicus]
WP_020223950.1 179   rubrerythrin [Holdemania massiliensis]
WP_023039557.1 179   hypothetical protein [Fusobacterium nucleatum]
WP_006058763.1 179   rubrerythrin [Holdemania filiformis]
WP_039680317.1 179   rubrerythrin [Terrisporobacter othiniensis]
WP_040001503.1 179   rubrerythrin [[Eubacterium] infirmum]
WP_006524887.1 179   rubrerythrin [Solobacterium moorei]
WP_009144889.1 179   rubrerythrin [Phascolarctobacterium succinatutens]
CCX54173.1   179   rubrerythrin [Bacteroides sp. CAG:1060]
CDD12344.1   179   rubrerythrin [Phascolarctobacterium succinatutens CAG:287]
CCX35743.1   179   putative uncharacterized protein [Clostridium sp.CAG:1000]
WP_028077463.1 179   rubrerythrin [Solobacterium moorei]
CDD03337.1   179   rubrerythrin [Ruminococcus sp. CAG:382]
WP_005891991.1 179   rubrerythrin [Fusobacterium nucleatum]
WP_009146377.1 179   rubrerythrin [Phascolarctobacterium succinatutens]
CDE32747.1   179   rubrerythrin [Ruminococcus sp. CAG:403]
WP_032089470.1 179   rubrerythrin [bacterium LF-3]

WP_028512264.1	179	rubrerythrin [Ruminococcaceae bacterium AE2021]
WP_005954576.1	179	rubrerythrin [Fusobacterium necrophorum]
WP_008908066.1	179	rubrerythrin [Caloramator australicus]
WP_027936832.1	179	rubrerythrin [Anaeroarcus burkinensis]
WP_018701561.1	179	rubrerythrin [Anaeromusa acidaminophila]
WP_029600159.1	179	rubrerythrin [Fusobacterium nucleatum]
WP_034433126.1	179	rubrerythrin [Clostridiales bacterium S5-A14a]
WP_057976096.1	179	rubrerythrin [Caloramator mitchellensis]
CCZ07142.1	179	putative uncharacterized protein [Clostridium sp.CAG:127]
WP_016319778.1	179	hypothetical protein [Firmicutes bacterium M10-2]
CCX75609.1	179	rubredoxin [Dorea sp. CAG:105]
WP_006440736.1	179	rubrerythrin [[Clostridium] hiranonis]
WP_057058621.1	179	rubrerythrin [Bacteroides fragilis]
WP_035378533.1	179	rubrerythrin [Fervidicella metallireducens]
CDC03154.1	179	putative uncharacterized protein [Eubacterium sp.CAG:202]
WP_021659528.1	179	rubrerythrin [Clostridium sp. ATCC 29733]
WP_012415204.1	179	rubrerythrin [Elusimicrobium minutum]
CDC78341.1	179	rubrerythrin [Clostridium sp. CAG:465]
WP_042704568.1	179	rubrerythrin [Methanobrevibacter arboriphilus]
WP_025489653.1	179	hypothetical protein [Clostridiales bacterium VE202-27]
WP_035134247.1	179	rubrerythrin [Clostridium sulfidigenes]
WP_044036981.1	179	rubrerythrin [Clostridium bornimense]
WP_002568997.1	179	MULTISPECIES: rubrerythrin [Lachnoclostridium]
WP_002586762.1	179	rubrerythrin [[Clostridium] clostridioforme]
WP_027643720.1	179	rubrerythrin [[Clostridium] clostridioforme]
WP_033144019.1	179	hypothetical protein [Blautia producta]
WP_022202198.1	179	hypothetical protein [[Clostridium] clostridioforme]
WP_007862118.1	179	MULTISPECIES: rubrerythrin [Clostridiales]
WP_008723809.1	179	MULTISPECIES: rubrerythrin [unclassified Clostridiales (miscellaneous)]
WP_027308395.1	179	rubrerythrin [Caloramator sp. ALD01]
WP_021636018.1	179	rubrerythrin [Clostridium sp. KLE 1755]
WP_044967375.1	179	hypothetical protein [Clostridiaceae bacterium MS3]
CUQ24787.1	179	NADH peroxidase [Fusicatenibacter]
WP_050005344.1	179	MULTISPECIES: rubrerythrin [unclassified Ruminococcaceae]
WP_009253215.1	179	MULTISPECIES: hypothetical protein [Clostridiales]
WP_018660847.1	179	rubrerythrin [Thermobrachium celere]
WP_040192962.1	179	rubrerythrin [Clostridium sp. CL-6]
WP_018593705.1	179	hypothetical protein [Blautia producta]
WP_040379305.1	179	rubrerythrin [Dethiobacter alkaliphilus]
WP_055666846.1	179	rubrerythrin [Clostridiaceae bacterium mt10]

CDD58548.1   179   rubrerythrin [Eggerthella sp. CAG:298]
WP_027113256.1 179   MULTISPECIES: reverse rubrerythrin-1 [unclassified Lachnospiraceae]
WP_046442318.1 179   reverse rubrerythrin-1 [Catabacter hongkongensis]
WP_024614812.1 179   rubrerythrin [Clostridium sp. Ade.TY]
CDA18773.1   179   rubrerythrin [Acetobacter sp. CAG:267]
WP_033165076.1 179   hypothetical protein [Clostridium sp. KNHs205]
WP_027423106.1 179   reverse rubrerythrin-1 [Lachnospiraceae bacterium AC3007]
WP_021366562.1 179   "reverse rubrerythrin-1, partial [Peptoclostridium difficile]"
WP_028329766.1 179   rubrerythrin [Brachyspira alvinipulli]
EQI82861.1   179   "reverse rubrerythrin-1, partial [Clostridium difficile Y401]"
WP_021366554.1 179   "reverse rubrerythrin-1, partial [Peptoclostridium difficile]"
WP_008726336.1 179   rubrerythrin fusion protein [Brachyspira hampsonii]
ELV06536.1   179   rubrerythrin fusion protein [Brachyspira hampsonii 30599]
CCY62621.1   179   putative uncharacterized protein [Clostridium sp.CAG:967]
WP_040843035.1 179   reverse rubrerythrin-1 [Treponema saccharophilum]
WP_014488538.1 179   MULTISPECIES: rubrerythrin [Brachyspira]
WP_013113095.1 179   rubrerythrin [Brachyspira murdochii]
WP_015711885.1 179   rubredoxin/rubrerythrin [Treponema azotonutricium]
CBL16263.1   179   Rubrerythrin [Ruminococcus bromii L2-63]
CDE88846.1   179   rubrerythrin [Clostridium sp. CAG:729]
WP_026517757.1 179   reverse rubrerythrin-1 [Butyrivibrio sp. MC2021]
WP_014932818.1 179   rubrerythrin [Brachyspira pilosicoli]
WP_020005024.1 179   rubrerythrin [Brachyspira innocens]
WP_026509021.1 179   MULTISPECIES: reverse rubrerythrin-1 [Butyrivibrio]
CCY23451.1   179   rubrerythrin [Brachyspira sp. CAG:484]
WP_040437975.1 179   reverse rubrerythrin-1 [[Clostridium] methylpentosum]
WP_016147133.1 179   hypothetical protein [Butyricicoccus pullicaecorum]
WP_034235716.1 179   reverse rubrerythrin-1 [Lachnospiraceae bacterium AC2029]
CCX84523.1   179   putative uncharacterized protein [Eubacterium sp. CAG:86]
CDA11980.1   179   putative uncharacterized protein [Anaerotruncus sp.CAG:528]
CDA69259.1   179   putative uncharacterized protein [Clostridium sp.CAG:510]

WP_016280494.1 179   hypothetical protein [Lachnospiraceae bacterium A4]
CCY68263.1   179   putative uncharacterized protein [Clostridium sp.CAG:678]
EOS29718.1   179   hypothetical protein C807_02759 [Lachnospiraceae bacterium 28-4]
CCY21366.1   179   putative uncharacterized protein [Firmicutes bacterium CAG:24]
EOS73637.1   179   hypothetical protein C819_03693 [Lachnospiraceae bacterium 10-1]
CDF08541.1   179   putative uncharacterized protein [Firmicutes bacterium CAG:95]
WP_022772152.1 179   reverse rubrerythrin-1 [Butyrivibrio sp. AE2015]
CDE36859.1   179   putative uncharacterized protein [Eubacterium sp. CAG:38]
WP_026670339.1 179   reverse rubrerythrin-1 [Butyrivibrio sp. AE3006]
CCY59447.1   179   putative uncharacterized protein [Clostridium sp.CAG:632]
WP_026524519.1 179   reverse rubrerythrin-1 [Butyrivibrio sp. MB2005]
ACN84712.1   179   putative rubrerythrin [Brachyspira hyodysenteriae WA1]
WP_022362346.1 179   hypothetical protein [[Bacteroides] pectinophilus]
WP_026495460.1 179   reverse rubrerythrin-1 [Butyrivibrio sp. WCD3002]
WP_026665369.1 179   reverse rubrerythrin-1 [Butyrivibrio sp. FC2001]
WP_026492710.1 179   reverse rubrerythrin-1 [Butyrivibrio sp. XPD2002]
ADL32783.1   179   rubrerythrin Rbr1 [Butyrivibrio proteoclasticus B316]
WP_035765516.1 179   reverse rubrerythrin-1 [Butyrivibrio sp. NC2002]
WP_026505531.1 179   reverse rubrerythrin-1 [Butyrivibrio sp. NC3005]
WP_034445127.1 179   reverse rubrerythrin-1 [Butyrivibrio sp. AE2032]
AEQ21849.1   179   rubrerythrin [Acidaminococcus intestini RyC-MR95]
WP_026526230.1 179   reverse rubrerythrin-1 [Butyrivibrio sp. VCD2006]
WP_026651398.1 179   reverse rubrerythrin-1 [Butyrivibrio proteoclasticus]
WP_022761780.1 179   MULTISPECIES: reverse rubrerythrin-1 [Butyrivibrio]
WP_022777364.1 179   reverse rubrerythrin-1 [Butyrivibrio sp. AE3009]

WP_026489234.1 179   reverse rubrerythrin-1 [Butyrivibrio sp. XBB1001]
3QHB   179   "Chain A, Crystal Structure Of Oxidized Symerythrin From Cyanophora Paradoxa"
WP_022932391.1 178   rubrerythrin [Treponema bryantii]
EMR75233.1   178   rubrerythrin [Thermoplasmatales archaeon SCGC AB-540-F20]
KKC30417.1   178   rubrerythrin [Caldanaerobacter subterraneus subsp.pacificus DSM 12653]
WP_006354878.1 178   rubrerythrin [[Clostridium] methylpentosum]
EEQ48736.1   178   Rubrerythrin [Selenomonas flueggei ATCC 43531]
CDA28954.1   178   putative uncharacterized protein [Eubacterium sp.CAG:156]
WP_008689243.1 178   rubrerythrin [Eubacterium sp. 3_1_31]
WP_035639684.1 178   rubrerythrin [Lachnospiraceae bacterium ND2006]
EFM23573.1   178   Rubrerythrin [Selenomonas sp. oral taxon 149 str.67H29BP]
WP_027356157.1 178   rubrerythrin [Desulfotomaculum thermocisternum]
WP_022936778.1 178   rubrerythrin [Dielma fastidiosa]
WP_015837207.1 178   rubrerythrin [Geobacter sp. M21]
WP_008981783.1 178   rubrerythrin [Ruminococcaceae bacterium D16]
WP_002703388.1 178   rubrerythrin [Treponema saccharophilum]
CCY74795.1   178   rubrerythrin [Brachyspira sp. CAG:700]
WP_009623251.1 178   rubrerythrin [Desulfosporosinus sp. OT]
CDD09682.1   178   rubrerythrin [Clostridium sp. CAG:349]
WP_029472984.1 178   rubrerythrin [Clostridiales bacterium VE202-08]
KGP74759.1   178   rubrerythrin [Desulfosporosinus sp. Tol-M]
WP_045173571.1 178   rubrerythrin [Caldicellulosiruptor sp. Wai35.B1]
WP_012530747.1 178   rubrerythrin [Geobacter bemidjiensis]
WP_024622279.1 178   rubrerythrin [[Clostridium] mangenotii]
WP_013821233.1 178   rubrerythrin [Desulfotomaculum kuznetsovii]
EOS77141.1   178   hypothetical protein C819_01167 [Lachnospiraceae bacterium 10-1]
WP_019880334.1 178   rubrerythrin [Succinispira mobilis]
WP_013485840.1 178   rubrerythrin [Ethanoligenens harbinense]
WP_013626084.1 178   rubrerythrin [Syntrophobotulus glycolicus]
WP_005362582.1 178   rubrerythrin [Eubacterium ventriosum]
AEB14972.1   178   Rubrerythrin [Treponema succinifaciens DSM 2489]
WP_023053787.1 178   rubrerythrin [Megasphaera sp. BV3C16-1]
WP_027128952.1 178   rubrerythrin [Fusobacterium perfoetens]
WP_002608358.1 178   MULTISPECIES: rubrerythrin [Firmicutes]
WP_009268439.1 178   rubrerythrin [Lachnospiraceae bacterium 1_4_56FAA]
WP_026842025.1 178   rubrerythrin [Geobacter bremensis]
CDA63156.1   178   putative uncharacterized protein [Clostridium

sp.CAG:169]
CDD93027.1   178   rubrerythrin [Coprobacillus sp. CAG:826]
WP_027431953.1 178   rubrerythrin [Lachnospira multipara]
CCX91739.1   178   rubredoxin [Succinatimonas sp. CAG:777]
WP_033169814.1 178   rubrerythrin [Selenomonas sp. ND2010]
WP_019230470.1 178   hypothetical protein [Sedimentibacter sp. B4]
WP_039766702.1 178   rubrerythrin [Caldicellulosiruptor sp. F32]
WP_045168709.1 178   rubrerythrin [Caldicellulosiruptor sp. Rt8.B8]
CDB03504.1   178   rubrerythrin [Firmicutes bacterium CAG:145]
WP_044919786.1 178   rubrerythrin [Lachnospiraceae bacterium
MA2020]
WP_006862834.1 178   rubrerythrin [Marvinbryantia formatexigens]
WP_015392522.1 178   rubrerythrin [Clostridium
saccharoperbutylacetonicum]
WP_027293863.1 178   MULTISPECIES: rubrerythrin [Robinsoniella]
WP_045164505.1 178   rubrerythrin [Thermoanaerobacter
cellulolyticus]
EQE67257.1   178   rubrerythrin family protein [Peptoclostridium
difficile CD44]
WP_011917929.1 178   rubrerythrin [Caldicellulosiruptor
saccharolyticus]
WP_012301673.1 178   rubrerythrin [Candidatus Desulforudis
audaxviator]
WP_029542833.1 178   rubrerythrin [Selenomonas ruminantium]
CDC01717.1   178   rubrerythrin [Eubacterium sp. CAG:202]
WP_039696657.1 178   rubrerythrin [Streptococcus equinus]
WP_042437063.1 178   rubrerythrin [Senegalimassilia anaerobia]
WP_003418513.1 178   rubrerythrin [Peptoclostridium difficile]
CDD01679.1   178   rubredoxin [Clostridium sp. CAG:91]
WP_022387718.1 178   rubrerythrin [Collinsella sp. MS5]
KPI56721.1   178   rubrerythrin [Peptoclostridium difficile]
WP_013243788.1 178   rubrerythrin [Brachyspira pilosicoli]
WP_014935420.1 178   rubrerythrin [Brachyspira pilosicoli]
WP_019225110.1 178   MULTISPECIES: rubrerythrin [Dehalobacter]
EQE38276.1   178   rubrerythrin family protein [Peptoclostridium
difficile CD38]
WP_034451650.1 178   rubrerythrin [Butyrivibrio sp. AE2032]
CCY43495.1   178   rubredoxin [Firmicutes bacterium CAG:124]
EQJ98241.1   178   rubrerythrin family protein [Clostridium
difficile P51]
WP_015720234.1 178   rubrerythrin [Geobacter sp. M18]
WP_006355112.1 178   rubrerythrin [[Clostridium] methylpentosum]
WP_016322976.1 178   hypothetical protein [Oscillibacter sp. 1-3]
EQF26450.1   178   rubrerythrin family protein [Peptoclostridium
difficile CD160]
WP_048817753.1 178   rubrerythrin [Desulfotomaculum hydrothermale]
WP_007393324.1 178   rubrerythrin [Megasphaera sp. UPII 135-E]
WP_006782398.1 178   rubrerythrin [Hungatella hathewayi]
CDD63385.1   178   rubrerythrin [Firmicutes bacterium CAG:341]
WP_021315908.1 178   rubrerythrin [Dehalobacter sp. UNSWDHB]

WP_024343854.1	178	rubrerythrin [Streptococcus equinus]
CBL41462.1	178	Rubrerythrin [butyrate-producing bacterium SS3/4]
WP_048513428.1	178	rubrerythrin [Megasphaera cerevisiae]
WP_010244460.1	178	rubrerythrin [Acetivibrio cellulolyticus]
KKM11446.1	178	rubrerythrin [Clostridiales bacterium PH28_bin88]
CDE26860.1	178	rubrerythrin [Clostridium sp. CAG:307]
ADO83345.1	178	Rubrerythrin [Ilyobacter polytropus DSM 2926]
CCY08662.1	178	rubrerythrin [Clostridium sp. CAG:81]
WP_011342577.1	178	rubrerythrin [Pelobacter carbinolicus]
WP_008723547.1	178	rubrerythrin [Brachyspira hampsonii]
WP_013658149.1	178	rubrerythrin [Cellulosilyticum lentocellum]
WP_006941331.1	178	rubrerythrin [Megasphaera micronuciformis]
ELV06264.1	178	rubrerythrin [Brachyspira hampsonii 30599]
WP_014488879.1	178	MULTISPECIES: rubrerythrin [Brachyspira]
CDA77831.1	178	putative uncharacterized protein [Clostridium sp.CAG:242]
WP_024733893.1	178	rubrerythrin [Clostridiales bacterium VE202-14]
WP_015044344.1	178	MULTISPECIES: rubrerythrin [Dehalobacter]
WP_027399522.1	178	rubrerythrin [Anaerovorax odorimutans]
WP_004800512.1	178	rubrerythrin [[Eubacterium] dolichum]
WP_007050132.1	178	rubrerythrin [Anaerofustis stercorihominis]
WP_008752418.1	178	rubrerythrin [Lachnoanaerobaculum saburreum]
WP_021431117.1	178	rubrerythrin family protein [[Clostridium]bifermentans]
WP_012671700.1	178	rubrerythrin [Brachyspira hyodysenteriae]
WP_042356834.1	178	rubrerythrin [Bacillus rubiinfantis]
WP_047111490.1	178	rubrerythrin [Brachyspira hyodysenteriae]
WP_048621151.1	178	rubrerythrin [Clostridium sp. BR72]
WP_057542265.1	178	rubrerythrin [[Clostridium] sordellii]
WP_013276576.1	178	rubrerythrin [Thermosediminibacter oceani]
WP_035118457.1	178	rubrerythrin [Clostridium sp. NCR]
WP_009446971.1	178	rubrerythrin [Lachnospiraceae bacterium oral taxon 082]
WP_028330731.1	178	rubrerythrin [Brachyspira alvinipulli]
WP_054631584.1	178	rubrerythrin [[Clostridium] sordellii]
WP_021127943.1	178	rubrerythrin family protein [[Clostridium] sordellii]
WP_034215548.1	178	rubrerythrin [Lachnoanaerobaculum sp. MSX33]
WP_046824347.1	178	rubrerythrin [Clostridium sp. JC272]
WP_013411122.1	178	rubrerythrin [Caldicellulosiruptor owensensis]
WP_009219421.1	178	rubrerythrin [Lachnoanaerobaculum sp. ICM7]
WP_021125837.1	178	rubrerythrin family protein [[Clostridium] sordellii]
AEE91227.1	178	Rubrerythrin [Tepidanaerobacter acetatoxydans Re1]
WP_057584028.1	178	rubrerythrin [[Clostridium] sordellii]
WP_007391020.1	178	rubrerythrin [Megasphaera sp. UPII 199-6]

WP_034813935.1 178   rubrerythrin [[Eubacterium] sulci]
WP_009369512.1 178   rubrerythrin [Megasphaera genomosp. type_1]
WP_057539896.1 178   rubrerythrin [[Clostridium] sordellii]
WP_007594112.1 178   rubrerythrin [Lachnoanaerobaculum sp. OBRC5-5]
WP_006790662.1 178   rubrerythrin [Anaeroglobus geminatus]
EHO86956.1   178   hypothetical protein HMPREF0380_00054
[Eubacterium infirmum F0142]
EHL65530.1   178   hypothetical protein HMPREF1032_01073
[Subdoligranulum sp. 4_3_54A2FAA]
WP_028307941.1 178   rubrerythrin [Desulfitibacter alkalitolerans]
WP_021429180.1 178   MULTISPECIES: rubrerythrin family
protein[Clostridiales]
WP_046821582.1 178   rubrerythrin [Clostridium sp. JC272]
WP_049728429.1 178   rubrerythrin [Dorea sp. D27]
WP_050624456.1 178   rubrerythrin [Clostridium sp. GD3]
WP_013289525.1 178   rubrerythrin [Caldicellulosiruptor
obsidiansis]
WP_021432674.1 178   rubrerythrin family protein
[[Clostridium]bifermentans]
WP_021433368.1 178   rubrerythrin family protein
[[Clostridium]bifermentans]
WP_033126119.1 178   rubrerythrin [Eubacterium sp. ER2]
WP_009004553.1 178   MULTISPECIES: rubrerythrin [Clostridiales]
WP_013431563.1 178   MULTISPECIES: rubrerythrin
[Caldicellulosiruptor]
WP_013404300.1 178   MULTISPECIES: rubrerythrin
[Caldicellulosiruptor]
WP_025653786.1 178   rubrerythrin [Clostridiales bacterium
VE202-21]
WP_050639143.1 178   MULTISPECIES: rubrerythrin [Clostridiales]
CCZ01374.1   178   rubrerythrin [Paraprevotella clara CAG:116]
WP_002605223.1 178   rubrerythrin [Hungatella hathewayi]
WP_022030365.1 178   rubrerythrin [Hungatella hathewayi]
WP_055070589.1 178   rubrerythrin [Clostridium sp. ND2]
WP_023345803.1 178   hypothetical protein [Firmicutes bacterium
ASF500]
WP_026891072.1 178   rubrerythrin [[Clostridium] aerotolerans]
WP_024837710.1 178   rubrerythrin [Clostridium sp. 12(A)]
WP_025230385.1 178   rubrerythrin [Clostridium sp. ASBs410]
WP_024292974.1 178   MULTISPECIES: rubrerythrin [Lachnoclostridium]
WP_038280208.1 178   rubrerythrin [[Clostridium] celerecrescens]
WP_049178950.1 178   rubrerythrin [Clostridium botulinum]
CDA05092.1   178   putative uncharacterized protein [Blautia sp.
CAG:257]
WP_027098775.1 178   rubrerythrin [Clostridium paraputrificum]
KJS83648.1   178   rubrerythrin [Peptococcaceae bacterium
BICA1-8]
KJS23214.1   178   rubrerythrin [Clostridiaceae bacterium
BRH_c20a]
KPK22420.1   178   "rubrerythrin, partial [Desulfobacterales

bacterium SG8_35_2]"
WP_055254197.1 178 rubrerythrin [Clostridium paraputrificum]
WP_013274656.1 178 rubrerythrin [[Clostridium] saccharolyticum]
WP_054629717.1 178 rubrerythrin [[Clostridium] sordellii]
WP_021127187.1 178 rubrerythrin family protein [[Clostridium] sordellii]
WP_033141650.1 178 rubrerythrin [Blautia producta]
WP_044938433.1 178 rubrerythrin [Blautia schinkii]
WP_057562474.1 178 rubrerythrin [[Clostridium] sordellii]
CDE88200.1 178 putative uncharacterized protein [Clostridium sp.CAG:729]
WP_040435119.1 178 rubrerythrin [[Clostridium] hylemonae]
WP_049039370.1 178 rubrerythrin [Clostridium perfringens]
WP_003477939.1 178 rubrerythrin [Clostridium perfringens]
WP_018596131.1 178 rubrerythrin [Blautia producta]
WP_003473134.1 178 rubrerythrin [Clostridium perfringens]
WP_003481393.1 178 rubrerythrin [Clostridium perfringens]
WP_003460610.1 178 rubrerythrin [Clostridium perfringens]
WP_045725837.1 178 rubrerythrin [Clostridium baratii]
WP_003453572.1 178 rubrerythrin [Clostridium perfringens]
WP_054199234.1 178 rubrerythrin [Clostridium baratii]
WP_055208052.1 178 rubrerythrin [Clostridium baratii]
WP_003466528.1 178 rubrerythrin [Clostridium perfringens]
WP_039311218.1 178 rubrerythrin [Clostridium baratii]
WP_054351543.1 178 rubrerythrin [Clostridia bacterium UC5.1-1D4]
CDC42139.1 178 putative uncharacterized protein [Firmicutes bacterium CAG:424]
WP_031473419.1 178 rubrerythrin [Eubacterium desmolans]
WP_013048613.1 178 rubrerythrin [Aminobacterium colombiense]
WP_028026273.1 178 rubrerythrin [Enterorhabdus mucosicola]
WP_022739967.1 178 rubrerythrin [Adlercreutzia equolifaciens]
WP_010076049.1 178 rubrerythrin [Clostridium cellulovorans]
WP_009248400.1 178 rubrerythrin [[Clostridium] scindens]
WP_016308901.1 178 hypothetical protein [Enterorhabdus caecimuris]
CDE92966.1 178 rubrerythrin [Fusobacterium sp. CAG:815]
WP_040917561.1 178 reverse rubrerythrin-1 [Subdoligranulum variabile]
AKQ47590.1 178 hypothetical protein TH63_09215 [Rufibacter sp. DG31D]
EQJ24546.1 178 "reverse rubrerythrin-1, partial [Clostridium difficile P9]"
ERM40919.1 178 "reverse rubrerythrin-1, partial [Clostridium difficile P64]"
EQJ24240.1 178 "reverse rubrerythrin-1, partial [Clostridium difficile P9]"
EQJ35399.1 178 "reverse rubrerythrin-1, partial [Clostridium difficile P20]"
CDE99076.1 178 rubrerythrin [Clostridium sp. CAG:813]
WP_021381700.1 178 "reverse rubrerythrin-1, partial

[Peptoclostridium difficile]"
WP_049589495.1 178   "reverse rubrerythrin-1, partial
[[Clostridium]clostridioforme]"
CDB51231.1   178   putative uncharacterized protein [Clostridium
sp.CAG:217]
WP_011143999.1 178   hypothetical protein [Gloeobacter violaceus]
WP_023174185.1 178   rubrerythrin [Gloeobacter kilaueensis]
WP_013238361.1 177   MULTISPECIES: rubrerythrin [Clostridium]
WP_013781664.1 177   rubrerythrin [Mahella australiensis]
CDC21482.1   177   rubrerythrin [Eubacterium sp. CAG:274]
CDD22375.1   177   rubrerythrin [Firmicutes bacterium CAG:313]
WP_047830918.1 177   rubrerythrin [Peptococcaceae bacterium CEB3]
WP_007063177.1 177   rubrerythrin [Clostridium carboxidivorans]
CDE72914.1   177   rubrerythrin [Acidaminococcus sp. CAG:917]
CDE84688.1   177   rubrerythrin [Clostridium sp. CAG:273]
WP_039980914.1 177   MULTISPECIES: rubrerythrin [Selenomonas]
WP_040572330.1 177   rubrerythrin [Selenomonas flueggei]
CCZ17737.1   177   rubrerythrin [Clostridium sp. CAG:780]
WP_003527938.1 177   rubrerythrin [[Clostridium] leptum]
CDC70172.1   177   rubredoxin [Staphylococcus sp. CAG:324]
CDE71935.1   177   rubrerythrin [Subdoligranulum sp. CAG:314]
KJS67185.1   177   rubrerythrin [Peptococcaceae bacterium
BICA1-7]
CDA59295.1   177   rubrerythrin [Clostridium sp. CAG:245]
ADI01029.1   177   Rubrerythrin [Syntrophothermus lipocalidus DSM
12680]
WP_006306094.1 177   MULTISPECIES: rubrerythrin [Veillonellaceae]
WP_009656134.1 177   rubrerythrin [Selenomonas sp. FOBRC6]
WP_031586436.1 177   rubrerythrin [Selenomonas bovis]
WP_054329943.1 177   rubrerythrin [Clostridia bacterium UC5.1-1D10]
WP_019542942.1 177   rubrerythrin [Selenomonas bovis]
WP_009439770.1 177   rubrerythrin [Selenomonas sp. oral taxon 138]
WP_032076770.1 177   rubrerythrin [Clostridium drakei]
WP_019552923.1 177   rubrerythrin [Propionispira raffinosivorans]
WP_021685384.1 177   rubrerythrin [Selenomonas sp. oral taxon 892]
WP_009657476.1 177   MULTISPECIES: rubrerythrin [Selenomonas]
WP_006693980.1 177   rubrerythrin [Selenomonas noxia]
WP_022790134.1 177   rubrerythrin [Faecalicoccus pleomorphus]
WP_029162604.1 177   rubrerythrin [Clostridium scatologenes]
WP_055310088.1 177   "rubrerythrin, partial [Collinsella
aerofaciens]"
WP_027889098.1 177   rubrerythrin [Megamonas hypermegale]
WP_015395546.1 177   rubrerythrin [Clostridium
saccharoperbutylacetonicum]
WP_037350416.1 177   rubrerythrin [Selenomonas sp. oral taxon 137]
CDB03564.1   177   rubrerythrin [Firmicutes bacterium CAG:145]
WP_026762330.1 177   rubrerythrin [Selenomonas artemidis]
KJS17558.1   177   rubrerythrin [Peptococcaceae bacterium
BRH_c4b]
WP_008539289.1 177   rubrerythrin [Megamonas funiformis]

CCO06940.1	177	Rubrerythrin [Desulfotomaculum hydrothermale Lam5 = DSM 18033]
WP_014183901.1	177	rubrerythrin [Desulfosporosinus orientis]
WP_021659805.1	177	rubredoxin [Clostridium sp. ATCC 29733]
WP_034600608.1	177	rubrerythrin [Desulfosporosinus sp. HMP52]
WP_042340030.1	177	rubrerythrin [Desulfosporosinus youngiae]
CDC47673.1	177	rubrerythrin [Clostridium sp. CAG:58]
WP_027627709.1	177	rubrerythrin [[Clostridium] cellobioparum]
WP_018999935.1	177	rubrerythrin [Megamonas rupellensis]
CBL05634.1	177	Rubrerythrin [Megamonas hypermegale ART12/1]
WP_013114436.1	177	rubrerythrin [Brachyspira murdochii]
WP_008398455.1	177	rubrerythrin [Clostridium sp. M62/1]
WP_020004453.1	177	rubrerythrin [Brachyspira innocens]
WP_054333856.1	177	rubrerythrin [Clostridia bacterium UC5.1-2E3]
WP_010244376.1	177	rubrerythrin [Acetivibrio cellulolyticus]
WP_014902697.1	177	rubrerythrin [Desulfosporosinus meridiei]
WP_050618655.1	177	rubrerythrin [Intestinimonas sp. GD2]
WP_006571644.1	177	rubrerythrin [Pseudoflavonifractor capillosus]
WP_007782240.1	177	rubrerythrin [Desulfosporosinus youngiae]
CDC73833.1	177	rubredoxin [Oscillibacter sp. CAG:155]
WP_006192533.1	177	MULTISPECIES: rubrerythrin [Selenomonas]
WP_027095820.1	177	rubrerythrin [[Clostridium] viride]
CDE11159.1	177	rubredoxin [Clostridium sp. CAG:354]
WP_041591522.1	177	rubrerythrin [Tepidanaerobacter acetatoxydans]
EKD51722.1	177	rubrerythrin [uncultured bacterium]
CCY19244.1	177	rubrerythrin [Eubacterium sp. CAG:786]
WP_013841576.1	177	rubrerythrin [Desulfotomaculum ruminis]
CCY26517.1	177	rubredoxin [Firmicutes bacterium CAG:114]
WP_007491437.1	177	MULTISPECIES: rubrerythrin [Clostridiales]
CDA92348.1	177	rubrerythrin [Firmicutes bacterium CAG:238]
WP_003506939.1	177	MULTISPECIES: rubrerythrin [Clostridiales]
CCY73427.1	177	rubrerythrin [Eubacterium sp. CAG:115]
CCZ94336.1	177	putative uncharacterized protein [Corallococcus sp.CAG:1435]
ABO50727.1	177	Rubrerythrin [Desulfotomaculum reducens MI-1]
WP_008711668.1	177	MULTISPECIES: rubrerythrin [Synergistaceae]
WP_021748137.1	177	MULTISPECIES: rubrerythrin [Oscillibacter]
CCX74263.1	177	rubrerythrin [Firmicutes bacterium CAG:83]
WP_027869841.1	177	rubrerythrin [Eubacterium sp. AB3007]
WP_037978119.1	177	rubrerythrin [Synergistes jonesii]
CDD42656.1	177	rubrerythrin [Clostridium sp. CAG:299]
WP_054032635.1	177	rubrerythrin [Desulfatitalea tepidiphila]
CCY08073.1	177	rubrerythrin [Coprobacillus sp. CAG:698]
WP_023625366.1	177	"rubrerythrin, partial [Clostridium tyrobutyricum]"
WP_040659995.1	177	rubrerythrin [Oscillibacter ruminantium]
WP_027105839.1	177	rubrerythrin [Lachnospiraceae bacterium V9D3004]
WP_033119021.1	177	rubrerythrin [Intestinimonas butyriciproducens]

WP_021283307.1 177 hypothetical protein [Clostridium sp. BL8]
WP_017416269.1 177 hypothetical protein [Clostridium tunisiense]
CCZ85072.1 177 protein with rubredoxin/rubrerythrin domain [Firmicutes bacterium CAG:631]
EQI42167.1 177 "reverse rubrerythrin-1, partial [Clostridium difficile Y202]"
EQI41801.1 177 "reverse rubrerythrin-1, partial [Clostridium difficile Y202]"
CDD21394.1 177 rubredoxin/rubrerythrin [Firmicutes bacterium CAG:313]
WP_021427044.1 177 "reverse rubrerythrin-1, partial [Peptoclostridium difficile]"
CCY07493.1 177 rubrerythrin [Coprobacillus sp. CAG:698]
CDC33602.1 177 putative uncharacterized protein [Eubacterium sp.CAG:251]
WP_003526982.1 177 "hypothetical protein, partial [[Clostridium]clostridioforme]"
KKR18417.1 177 hypothetical protein UT47_C0002G0156 [candidate division CPR2 bacterium GW2011_GWC2_39_35]
ACM24105.1 176 Rubrerythrin [Thermotoga neapolitana DSM 4359]
KPJ54463.1 176 rubrerythrin [candidate division TA06 bacterium DG_24]
WP_052219092.1 176 rubrerythrin [Thermincola ferriacetica]
CDB25966.1 176 rubrerythrin [Firmicutes bacterium CAG:552]
WP_013119345.1 176 rubrerythrin [Thermincola potens]
WP_022582615.1 176 "reverse rubrerythrin-1, partial [Peptoclostridium difficile]"
WP_042745527.1 176 reverse rubrerythrin-1 [Peptoclostridium difficile]
EQF25406.1 176 "reverse rubrerythrin-1, partial [Peptoclostridium difficile CD160]"
EQH48511.1 176 "reverse rubrerythrin-1, partial [Clostridium difficile DA00246]"
WP_022582623.1 176 "reverse rubrerythrin-1, partial [Peptoclostridium difficile]"
WP_021413664.1 176 "reverse rubrerythrin-1, partial [Peptoclostridium difficile]"
EQF25525.1 176 "reverse rubrerythrin-1, partial [Peptoclostridium difficile CD160]"
WP_033127650.1 176 reverse rubrerythrin-1 [Clostridium butyricum]
CAB49805.1 175 rr rubrerythrin [Pyrococcus abyssi GE5]
KJR96291.1 175 rubrerythrin [Peptococcaceae bacterium BRH_c4a]
KKQ54663.1 175 Rubrerythrin [Parcubacteria (Falkowbacteria) bacterium GW2011_GWF2_38_1205]
WP_031535556.1 175 MULTISPECIES: rubrerythrin [Bacteroides]
KMY69297.1 175 rubrerythrin [Desulfocarbo indianensis]
EQE41816.1 175 "reverse rubrerythrin-1, partial [Peptoclostridium difficile CD40]"
EQG26477.1 175 "reverse rubrerythrin-1, partial

[Peptoclostridium difficile DA00114]"
EQE20366.1 175 "reverse rubrerythrin-1, partial [Peptoclostridium difficile CD17]"
EQE26224.1 175 "reverse rubrerythrin-1, partial [Peptoclostridium difficile CD21]"
EQH82730.1 175 "reverse rubrerythrin-1, partial [Clostridium difficile DA00307]"
EQF91822.1 175 "reverse rubrerythrin-1, partial [Peptoclostridium difficile 824]"
EQF54971.1 175 "reverse rubrerythrin-1, partial [Peptoclostridium difficile CD181]"
EQE78858.1 175 "reverse rubrerythrin-1, partial [Peptoclostridium difficile CD51]"
WP_021423348.1 175 "reverse rubrerythrin-1, partial [Peptoclostridium difficile]"
AKB68801.1 174 Rubrerythrin [Methanosarcina mazei LYC]
AGF97217.1 174 Rubrerythrin [Methanosarcina mazei Tuc01]
AAM31500.1 174 Rubrerythrin [Methanosarcina mazei Go1]
AKB41797.1 174 Rubrerythrin [Methanosarcina mazei WWM610]
AKB71444.1 174 Rubrerythrin [Methanosarcina mazei C16]
WP_054839656.1 174 rubrerythrin [Thermococcus sp. JCM 11816]
WP_042315580.1 174 rubrerythrin [Desulfotomaculum acetoxidans]
EIP85948.1 174 Rubrerythrin [Burkholderia thailandensis MSMB43]
EQE12964.1 174 "reverse rubrerythrin-1, partial [Peptoclostridium difficile CD8]"
EQF07972.1 174 "reverse rubrerythrin-1, partial [Peptoclostridium difficile CD132]"
EQH20701.1 174 "reverse rubrerythrin-1, partial [Clostridium difficile DA00210]"
WP_021424800.1 174 "reverse rubrerythrin-1, partial [Peptoclostridium difficile]"
WP_021427554.1 174 "reverse rubrerythrin-1, partial [Peptoclostridium difficile]"
WP_028585433.1 173 rubrerythrin [Desulfobulbus mediterraneus]
WP_035556493.1 173 rubrerythrin [Hippea sp. KM1]
AAM23767.1 173 Rubrerythrin [Caldanaerobacter subterraneus subsp.tengcongensis MB4]
ERM93235.1 173 rubrerythrin [Caldanaerobacter subterraneus subsp.yonseiensis KB-1]
EKD37078.1 173 rubrerythrin [uncultured bacterium]
KJS03527.1 173 rubrerythrin [Desulfobulbaceae bacterium BRH_c16a]
KPK99801.1 173 rubrerythrin [candidate division Zixibacteria bacterium SM23_73_3]
KPL19629.1 173 rubrerythrin [candidate division Zixibacteria bacterium SM23_81]
ABD42435.1 173 Rubrerythrin [Methanospirillum hungatei JF-1]
WP_015948259.1 173 MULTISPECIES: rubrerythrin [Desulfatibacillum]
KJS28763.1 173 rubrerythrin [Desulfatitalea sp. BRH_c12]

WP_028052789.1 173   rubrerythrin [Carboxydothermus ferrireducens]
EKD38415.1   173   rubrerythrin [uncultured bacterium]
WP_011345464.1 173   rubrerythrin [Carboxydothermus hydrogenoformans]
CDE90568.1   173   rubrerythrin [Clostridium sp. CAG:389]
WP_020877417.1 173   rubrerythrin [Desulfococcus multivorans]
WP_016322509.1 173   hypothetical protein [Oscillibacter sp. 1-3]
EQF21530.1   173   "reverse rubrerythrin-1, partial [Peptoclostridium difficile CD144]"
WP_021419068.1 173   "reverse rubrerythrin-1, partial [Peptoclostridium difficile]"
WP_021419502.1 173   "reverse rubrerythrin-1, partial [Peptoclostridium difficile]"
WP_042265326.1 173   reverse rubrerythrin-1 [Clostridium butyricum]
KPQ41704.1   173   Rubrerythrin [Candidatus Methanoperedens sp. BLZ1]
WP_022658071.1 172   rubrerythrin [Desulfovibrio desulfuricans]
WP_012868858.1 172   rubrerythrin [Thermanaerovibrio acidaminovorans]
EMR74224.1   172   "rubrerythrin, partial [Thaumarchaeota archaeon SCGC AB-539-E09]"
WP_024333211.1 172   rubrerythrin [Desulfotignum balticum]
WP_006967936.1 172   rubrerythrin [Desulfotignum phosphitoxidans]
WP_053985735.1 172   hypothetical protein [Lachnospiraceae bacterium mt14]
WP_031515775.1 172   rubrerythrin [Desulfotomaculum alkaliphilum]
EQF15708.1   172   "reverse rubrerythrin-1, partial [Peptoclostridium difficile CD144]"
KKR21611.1   172   hypothetical protein UT50_C0006G0025 [Parcubacteria(Moranbacteria) bacterium GW2011_GWA2_39_41]
WP_011345154.1 172   MULTISPECIES: hypothetical protein [Carboxydothermus]
KKU52183.1   172   hypothetical protein UX75_C0055G0005 [Parcubacteria(Moranbacteria) bacterium GW2011_GWE2_47_10]
KKQ13863.1   172   hypothetical protein US27_C0008G0046 [Parcubacteria(Moranbacteria) bacterium GW2011_GWF1_36_78]
EKE10955.1   172   Rubrerythrin [uncultured bacterium]
KKP79537.1   172   hypothetical protein UR78_C0012G0028 [Parcubacteria(Moranbacteria) bacterium GW2011_GWF2_35_39]
EKE16443.1   172   Rubrerythrin [uncultured bacterium]
KKP71219.1   172   hypothetical protein UR69_C0001G0043 [Parcubacteria(Moranbacteria) bacterium GW2011_GWE2_35_2-]
WP_048146717.1 171   rubrerythrin [Pyrococcus abyssi]
WP_048147757.1 171   rubrerythrin [Palaeococcus ferrophilus]
WP_050003413.1 171   rubrerythrin [Thermococcus eurythermalis]
WP_013749069.1 171   rubrerythrin [Pyrococcus sp. NA2]
WP_011012427.1 171   rubrerythrin [Pyrococcus furiosus]
WP_012571826.1 171   rubrerythrin [Thermococcus onnurineus]
WP_010478456.1 171   rubrerythrin [Thermococcus zilligii]
WP_014011728.1 171   rubrerythrin [Thermococcus sp. 4557]

WP_004068452.1 171 rubrerythrin [Thermococcus litoralis]
WP_042689964.1 171 rubrerythrin [Thermococcus nautili]
WP_055429768.1 171 rubrerythrin [Thermococcus thioreducens]
WP_015850161.1 171 rubrerythrin [Thermococcus sibiricus]
WP_013466628.1 171 rubrerythrin [Thermococcus barophilus]
WP_042679825.1 171 rubrerythrin [Thermococcus paralvinellae]
WP_011249478.1 171 rubrerythrin [Thermococcus kodakarensis]
AAB88944.1 171 rubrerythrin (rr4) [Archaeoglobus fulgidus DSM 4304]
WP_014121753.1 171 rubrerythrin [Thermococcus sp. AM4]
WP_055281182.1 171 rubrerythrin [Thermococcus sp. EP1]
WP_013905722.1 171 rubrerythrin [Pyrococcus yayanosii]
WP_048103648.1 171 rubrerythrin [Aciduliprofundum sp. MAR08-339]
KPK68360.1 171 rubrerythrin [candidate division WOR_3 bacterium SM23_60]
EFK11851.1 171 rubredoxin [delta proteobacterium NaphS2]
WP_048086195.1 171 rubrerythrin [Archaeoglobus veneficus]
EQG96766.1 171 "reverse rubrerythrin-1, partial [Clostridium difficile DA00193]"
WP_021434702.1 171 "reverse rubrerythrin-1, partial [Peptoclostridium difficile]"
WP_021422858.1 171 "reverse rubrerythrin-1, partial [Peptoclostridium difficile]"
WP_021368959.1 171 "reverse rubrerythrin-1, partial [Peptoclostridium difficile]"
WP_018962813.1 170 rubrerythrin [Coprothermobacter platensis]
3MPS 170 "Chain A, Peroxide Bound Oxidized Rubrerythrin From Pyrococcus Furiosus"
AFL96038.1 170 rubrerythrin [Thermococcus cleftensis]
WP_012583998.1 170 rubrerythrin [Dictyoglomus turgidum]
WP_012547135.1 170 rubrerythrin [Dictyoglomus thermophilum]
WP_008192590.1 170 MULTISPECIES: rubrerythrin [Thermotoga]
WP_048064432.1 170 rubrerythrin [Archaeoglobus fulgidus]
WP_038068166.1 170 MULTISPECIES: rubrerythrin [Thermotoga]
WP_004081119.1 170 MULTISPECIES: rubrerythrin [Thermotoga]
WP_038054427.1 170 rubrerythrin [Thermotoga sp. Mc24]
WP_036226132.1 170 rubrerythrin [Mesoaciditoga lauensis]
WP_041082786.1 170 rubrerythrin [Thermotoga profunda]
WP_041075499.1 170 rubrerythrin [Thermotoga caldifontis]
WP_045088447.1 170 rubrerythrin [Defluviitoga tunisiensis]
WP_014164013.1 170 rubrerythrin [Thermovirga lienii]
WP_041638405.1 170 rubrerythrin [Marinitoga piezophila]
GAP12487.1 170 rubrerythrin [Longilinea arvoryzae]
WP_006008182.1 170 rubrerythrin [Desulfovibrio piger]
AIY91169.1 170 Rubrerythrin [Geoglobus acetivorans]
ADK30796.1 170 rubrerythrin [Brachyspira pilosicoli 95/1000]
XP_001314607.1 170 rubrerythrin [Trichomonas vaginalis G3]
WP_021418492.1 170 "reverse rubrerythrin-1, partial [Peptoclostridium difficile]"
WP_043897810.1 170 reverse rubrerythrin-1 [Peptoclostridium

difficile]
EQH29722.1   170   "reverse rubrerythrin-1, partial [Clostridium difficile DA00215]"
EQH29766.1   170   "reverse rubrerythrin-1, partial [Clostridium difficile DA00215]"
WP_032523495.1 170   reverse rubrerythrin-1 [Peptoclostridium difficile]
WP_032507558.1 170   reverse rubrerythrin-1 [Peptoclostridium difficile]
WP_032520770.1 170   reverse rubrerythrin-1 [Peptoclostridium difficile]
WP_032549557.1 170   reverse rubrerythrin-1 [Peptoclostridium difficile]
WP_043897815.1 170   reverse rubrerythrin-1 [Peptoclostridium difficile]
WP_032507018.1 170   reverse rubrerythrin-1 [Peptoclostridium difficile]
WP_032517945.1 170   reverse rubrerythrin-1 [Peptoclostridium difficile]
WP_032544195.1 170   reverse rubrerythrin-1 [Peptoclostridium difficile]
WP_032508947.1 170   reverse rubrerythrin-1 [Peptoclostridium difficile]
WP_032512601.1 170   reverse rubrerythrin-1 [Peptoclostridium difficile]
WP_032541984.1 170   reverse rubrerythrin-1 [Peptoclostridium difficile]
WP_032544544.1 170   reverse rubrerythrin-1 [Peptoclostridium difficile]
WP_032517225.1 170   reverse rubrerythrin-1 [Peptoclostridium difficile]
WP_043896900.1 170   reverse rubrerythrin-1 [Peptoclostridium difficile]
WP_021419183.1 170   "reverse rubrerythrin-1, partial [Peptoclostridium difficile]"
WP_021424112.1 170   "reverse rubrerythrin-1, partial [Peptoclostridium difficile]"
WP_044213206.1 170   reverse rubrerythrin-1 [Peptoclostridium difficile]
WP_046059266.1 170   "reverse rubrerythrin-1, partial [Clostridium sp.IBUN22A]"
WP_054322877.1 170   "reverse rubrerythrin-1, partial [Clostridia bacterium UC5.1-2H6]"
WP_049975870.1 170   hypothetical protein [Azospirillum sp. B506]
WP_012543506.1 169   rubrerythrin [Coprothermobacter proteolyticus]
WP_028844655.1 169   rubrerythrin [Thermodesulfovibrio thiophilus]
WP_012056856.1 169   rubrerythrin [Thermosipho melanesiensis]
CEP79124.1   169   rubrerythrin [Defluviitoga tunisiensis]
WP_035586472.1 169   rubrerythrin [Hippea jasoniae]
WP_048086687.1 169   rubrerythrin [Ferroglobus placidus]

ABX32326.1   169   Rubrerythrin [Petrotoga mobilis SJ95]
WP_053549390.1 169   rubrerythrin [Desulfuromonas sp. WTL]
WP_027716104.1 169   rubrerythrin [Desulfuromonas sp. TF]
WP_040201673.1 169   rubrerythrin [Geoalkalibacter subterraneus]
AEX84558.1   169   rubrerythrin [Marinitoga piezophila KA3]
WP_029909819.1 169   rubrerythrin [Pelobacter seleniigenes]
WP_047267758.1 169   rubrerythrin [Marinitoga sp. 1197]
WP_047265595.1 169   rubrerythrin [Marinitoga sp. 1155]
WP_048093904.1 169   rubrerythrin [Geoglobus acetivorans]
XP_002682904.1 169   predicted protein [Naegleria gruberi]
WP_040096287.1 169   rubrerythrin [Geoalkalibacter ferrihydriticus]
WP_011339735.1 169   rubrerythrin [Pelobacter carbinolicus]
WP_052611321.1 169   hypothetical protein [Berkelbacteria bacterium GW2011_GWE1_39_12]
WP_021386987.1 169   "reverse rubrerythrin-1, partial [Peptoclostridium difficile]"
WP_017353160.1 169   "reverse rubrerythrin-1, partial [Clostridium botulinum]"
WP_021386989.1 169   "reverse rubrerythrin-1, partial [Peptoclostridium difficile]"
WP_029523420.1 169   bacterioferritin [Persephonella sp. KM09-Lau-8]
WP_012469849.1 168   rubrerythrin [Geobacter lovleyi]
WP_008086423.1 168   rubrerythrin [Aciduliprofundum boonei]
WP_012997248.1 168   rubrerythrin [Aciduliprofundum boonei]
WP_012579699.1 168   rubrerythrin [Thermosipho africanus]
WP_011940927.1 168   rubrerythrin [Geobacter uraniireducens]
WP_012648515.1 168   rubrerythrin [Geobacter daltonii]
AGB04028.1   168   rubrerythrin [Aciduliprofundum sp. MAR08-339]
WP_054698468.1 168   rubrerythrin [Geobacter toluenoxydans]
WP_010943250.1 168   rubrerythrin [Geobacter sulfurreducens]
WP_052400311.1 168   rubrerythrin [Geoglobus acetivorans]
WP_039643317.1 168   rubrerythrin [Geobacter soli]
WP_013682176.1 168   rubrerythrin [Hippea maritima]
KPK91888.1   168   rubrerythrin [Deltaproteobacteria bacterium SM23_61]
WP_011736481.1 168   rubrerythrin [Pelobacter propionicus]
ABM95054.1   168   putative oxidative stress related rubrerythrin protein[Methylibium petroleiphilum PM1]
CDD05192.1   168   rubrerythrin [Prevotella sp. CAG:592]
ABN06288.1   168   Rubrerythrin [Methanocorpusculum labreanum Z]
WP_028324954.1 168   rubrerythrin [Desulfatirhabdium butyrativorans]
CDG81220.1   168   rubrerythrin family protein [Janthinobacterium agaricidamnosum NBRC 102515 = DSM 9628]
WP_015949150.1 168   MULTISPECIES: rubrerythrin [Desulfatibacillum]
WP_042258220.1 168   "reverse rubrerythrin-1, partial [Clostridium perfringens]"
WP_054876077.1 168   hypothetical protein [Oxobacter pfennigii]
KPL03135.1   167   rubrerythrin [candidate division Zixibacteria

bacterium SM23_73_2]
WP_013298645.1 167   rubrerythrin [Thermoanaerobacterium thermosaccharolyticum]
KQC10905.1   167   rubrerythrin [Smithella sp. SDB]
WP_014756965.1 167   rubrerythrin [Thermoanaerobacterium aotearoense]
WP_026487452.1 167   rubrerythrin [Caldanaerobius polysaccharolyticus]
WP_013788650.1 167   rubrerythrin [Thermoanaerobacterium xylanolyticum]
WP_033191165.1 167   rubrerythrin [Fervidobacterium islandicum]
WP_011993652.1 167   rubrerythrin [Fervidobacterium nodosum]
WP_045411367.1 167   rubrerythrin [Thermoanaerobacterium saccharolyticum]
WP_015312314.1 167   rubrerythrin [Thermoanaerobacterium thermosaccharolyticum]
KON34119.1   167   rubrerythrin [miscellaneous Crenarchaeota group-1 archaeon SG8-32-1]
WP_006928424.1 167   rubrerythrin [Caldithrix abyssi]
WP_014453415.1 167   rubrerythrin [Caldisericum exile]
KLU40017.1   167   rubrerythrin [Peptococcaceae bacterium 1109]
EQB65218.1   167   rubrerythrin [Thermoplasmatales archaeon I-plasma]
WP_046736262.1 167   rubrerythrin [Dehalogenimonas sp. WBC-2]
WP_012994590.1 167   rubrerythrin [Thermoanaerobacter italicus]
WP_013931403.1 167   rubrerythrin [Pseudothermotoga thermarum]
CBE68344.1   167   Rubrerythrin [Candidatus Methylomirabilis oxyfera]
WP_027364126.1 167   rubrerythrin [Desulfotomaculum alcoholivorax]
WP_009051757.1 167   MULTISPECIES: rubrerythrin [Thermoanaerobacter]
WP_012003643.1 167   MULTISPECIES: rubrerythrin [Pseudothermotoga]
WP_003866895.1 167   MULTISPECIES: rubrerythrin [Thermoanaerobacter]
KPL06140.1   167   rubrerythrin [bacterium SM23_57]
WP_013149890.1 167   rubrerythrin [Thermoanaerobacter mathranii]
WP_026176732.1 167   rubrerythrin [Desulfurispora thermophila]
WP_028991827.1 167   rubrerythrin [Thermoanaerobacter thermocopriae]
WP_049684593.1 167   rubrerythrin [Thermoanaerobacter kivui]
KKK46260.1   167   Rubrerythrin-2 [Lokiarchaeum sp. GC14_75]
WP_041587102.1 167   rubrerythrin [Caldanaerobacter subterraneus]
WP_014808276.1 167   rubrerythrin [Desulfomonile tiedjei]
KON26400.1   167   rubrerythrin [miscellaneous Crenarchaeota group archaeon SMTZ1-55]
WP_031313880.1 167   rubrerythrin [Caldanaerobacter subterraneus]
KON31686.1   167   rubrerythrin [miscellaneous Crenarchaeota group-1 archaeon SG8-32-3]
KON28805.1   167   rubrerythrin [miscellaneous Crenarchaeota group archaeon SMTZ-80]

WP_014451123.1 167   rubrerythrin [Fervidobacterium pennivorans]
KJS11916.1   167   rubrerythrin [Peptococcaceae bacterium BRH_c8a]
KRT72065.1   167   rubrerythrin [Deltaproteobacteria bacterium CSP1-8]
KKP71050.1   167   Rubrerythrin [Parcubacteria (Moranbacteria) bacterium GW2011_GWE2_35_2-]
KPJ99824.1   167   rubrerythrin [Nitrospira bacterium SG8_35_4]
WP_014810435.1 167   rubrerythrin [Desulfomonile tiedjei]
WP_025391425.1 167   rubrerythrin [Desulfurella acetivorans]
KKP90009.1   167   rubrerythrin/rubredoxin [Parcubacteria bacterium GW2011_GWC1_36_108]
KON34228.1   167   rubrerythrin [miscellaneous Crenarchaeota group-6 archaeon AD8-1]
KKQ52052.1   167   Rubrerythrin [Parcubacteria bacterium GW2011_GWD2_38_11]
EKD46580.1   167   Rubrerythrin [uncultured bacterium]
EKE20319.1   167   Rubrerythrin [uncultured bacterium]
WP_019178603.1 167   hypothetical protein [Methanomassiliicoccus luminyensis]
WP_012624529.1 167   rubrerythrin [Desulfovibrio desulfuricans]
KQM11864.1   167   rubrerythrin [Methanomassiliicoccales archaeon RumEn M1]
KKP92427.1   167   Rubrerythrin [Parcubacteria (Moranbacteria) bacterium GW2011_GWD2_36_12]
EMS73422.1   167   rubrerythrin [[[Clostridium] termitidis CT1112]
WP_008686080.1 167   MULTISPECIES: rubrerythrin [Desulfovibrio]
EKE19003.1   167   rubrerythrin [uncultured bacterium]
WP_015284115.1 167   rubrerythrin [Methanoregula formicica]
WP_006524683.1 167   rubrerythrin [Desulfotomaculum gibsoniae]
WP_011991094.1 167   rubrerythrin [Methanoregula boonei]
KPK65759.1   167   rubrerythrin [Planctomycetes bacterium SM23_32]
WP_026783776.1 167   rubrerythrin [Pleomorphomonas koreensis]
WP_007292222.1 167   rubrerythrin [delta proteobacterium MLMS-1]
KJS28756.1   167   rubrerythrin [Desulfatitalea sp. BRH_c12]
EXY29494.1   167   rubrerythrin family protein [Bacteroides fragilis str.3397 T10]
WP_007294941.1 167   rubrerythrin [delta proteobacterium MLMS-1]
EXY93197.1   167   rubrerythrin family protein [Bacteroides fragilis str.3998T(B)3]
CDC51032.1   167   uncharacterized protein BN532_01589 [Bacteroides finegoldii CAG:203]
XP_005839869.1 167   hypothetical protein GUITHDRAFT_92111 [Guillardia theta CCMP2712]
WP_012175599.1 167   rubrerythrin [Desulfococcus oleovorans]
WP_026790807.1 167   rubrerythrin [Pleomorphomonas oryzae]
WP_041273462.1 167   rubrerythrin [Desulfobacterium autotrophicum]
WP_021403448.1 167   "reverse rubrerythrin-1, partial

[Peptoclostridium difficile]"
EKE02998.1	167	rubrerythrin [uncultured bacterium]
WP_014747064.1	167	rubrerythrin [Tistrella mobilis]
WP_018590505.1	167	"reverse rubrerythrin-1, partial
[Terrisporobacter glycolicus]"
WP_021403554.1	167	"reverse rubrerythrin-1, partial
[Peptoclostridium difficile]"
WP_013011601.1	167	bacterioferritin [Denitrovibrio acetiphilus]
WP_029896351.1	166	rubrerythrin [Desulfovibrio sp. L21-Syr-AB]
WP_013217581.1	166	rubrerythrin [Dehalogenimonas
lykanthroporepellens]
WP_024822853.1	166	rubrerythrin [Aminobacterium mobile]
WP_011421245.1	166	rubrerythrin [Anaeromyxobacter dehalogenans]
WP_015591147.1	166	rubrerythrin [Archaeoglobus sulfaticallidus]
WP_012525731.1	166	rubrerythrin [Anaeromyxobacter sp. K]
WP_011714834.1	166	rubrerythrin [Magnetococcus marinus]
GAO05004.1	166	rubrerythrin-2 [Anaeromyxobacter sp. PSR-1]
ADC66619.1	166	Rubrerythrin [Ferroglobus placidus DSM 10642]
WP_048094384.1	166	rubrerythrin [Geoglobus ahangari]
WP_052645240.1	166	rubrerythrin [haloalkaliphilic bacterium
ACht6-1]
KON27072.1	166	rubrerythrin [miscellaneous Crenarchaeota
group archaeon SMTZ-80]
WP_048094588.1	166	rubrerythrin [Geoglobus ahangari]
WP_022666012.1	166	rubrerythrin [Desulfospira joergensenii]
WP_006002158.1	166	rubrerythrin [Desulfuromonas acetoxidans]
WP_020447997.1	166	rubrerythrin [Candidatus Methanomassiliicoccus
intestinalis]
WP_011362987.1	166	rubrerythrin [Chlorobium chlorochromatii]
WP_028317500.1	166	rubrerythrin [Desulfobulbus elongatus]
WP_028578759.1	166	rubrerythrin [Desulfobulbus japonicus]
WP_007314653.1	166	rubrerythrin [Methanolinea tarda]
WP_028583963.1	166	rubrerythrin [Desulfobulbus mediterraneus]
CDE54245.1	166	rubredoxin [Roseburia sp. CAG:303]
WP_011745403.1	166	rubrerythrin [Chlorobium phaeobacteroides]
WP_012475042.1	166	rubrerythrin [Chlorobium phaeobacteroides]
WP_011357341.1	166	rubrerythrin [Pelodictyon luteolum]
WP_004040621.1	166	rubrerythrin [Methanofollis liminatans]
WP_011890428.1	166	rubrerythrin [Chlorobium phaeovibrioides]
WP_015904760.1	166	rubrerythrin [Desulfobacterium autotrophicum]
WP_048068074.1	166	rubrerythrin [Methanospirillum hungatei]
EKE25211.1	166	rubrerythrin [uncultured bacterium]
WP_012502580.1	166	rubrerythrin [Chlorobaculum parvum]
WP_012966954.1	166	rubrerythrin [Ferroglobus placidus]
WP_035239082.1	166	rubrerythrin [Desulfobacter vibrioformis]
EKD58343.1	166	rubrerythrin [uncultured bacterium]
WP_012475810.1	166	rubrerythrin [Chlorobium phaeobacteroides]
WP_004079793.1	166	rubrerythrin [Methanoplanus limicola]
WP_052881507.1	166	rubrerythrin [Verrucomicrobia bacterium L21-
Fru-AB]

EQC69275.1	166	Rubrerythrin [Streptococcus sp. HSISB1]
WP_010932995.1	166	rubrerythrin [Chlorobaculum tepidum]
WP_015721667.1	166	rubrerythrin [Geobacter sp. M18]
WP_013330544.1	166	rubrerythrin [Methanolacinia petrolearia]
EKE18894.1	166	rubrerythrin [uncultured bacterium]
EFW89038.1	166	Rubrerythrin [Streptococcus equinus ATCC 9812]
WP_012633023.1	166	rubrerythrin [Anaeromyxobacter dehalogenans]
WP_012097967.1	166	rubrerythrin [Anaeromyxobacter sp. Fw109-5]
WP_004514276.1	166	rubrerythrin [Geobacter metallireducens]
KGO35800.1	166	rubrerythrin [Desulfobulbus sp. Tol-SR]
WP_048149367.1	166	rubrerythrin [Methanolacinia paynteri]
WP_015402606.1	166	rubrerythrin [Desulfocapsa sulfexigens]
KJS03222.1	166	rubrerythrin [Desulfobulbaceae bacterium BRH_c16a]
WP_015725946.1	166	rubrerythrin [Desulfobulbus propionicus]
WP_048183367.1	166	rubrerythrin [Methanoculleus sp. S3Fa]
WP_012617002.1	166	rubrerythrin [Methanosphaerula palustris]
WP_012774603.1	166	rubrerythrin [Geobacter sp. M21]
WP_048113184.1	166	rubrerythrin [Methanoculleus sp. MH98A]
WP_026840800.1	166	rubrerythrin [Geobacter bremensis]
WP_012531754.1	166	rubrerythrin [Geobacter bemidjiensis]
WP_011843024.1	166	rubrerythrin [Methanoculleus marisnigri]
WP_004074491.1	166	rubrerythrin [Desulfobacter postgatei]
WP_006966061.1	166	MULTISPECIES: rubrerythrin-2 NADH peroxidase Rbr[Desulfotignum]
WP_048061934.1	166	rubrerythrin [Methanocorpusculum labreanum]
KQC05233.1	166	rubrerythrin [Methanoculleus sp. SDB]
WP_014585762.1	166	rubrerythrin [Methanosaeta harundinacea]
WP_012507737.1	166	rubrerythrin [Pelodictyon phaeoclathratiforme]
KQC14425.1	166	rubrerythrin [Methanosaeta sp. SDB]
WP_011384243.1	166	rubrerythrin [Magnetospirillum magneticum]
WP_008619161.1	166	rubrerythrin [Magnetospirillum sp. SO-1]
WP_042706083.1	166	rubrerythrin [Methanomicrobium mobile]
WP_012507603.1	166	rubrerythrin [Pelodictyon phaeoclathratiforme]
WP_048104377.1	166	rubrerythrin [Methanoculleus bourgensis]
WP_013450750.1	166	rubrerythrin [Calditerrivibrio nitroreducens]
WP_012506730.1	166	rubrerythrin [Prosthecochloris aestuarii]
WP_011832490.1	166	rubrerythrin [Methanocorpusculum labreanum]
WP_021132457.1	166	rubrerythrin [Phaeospirillum fulvum]
WP_028322189.1	166	rubrerythrin [Desulfatiglans anilini]
WP_042696680.1	166	rubrerythrin [Methanocorpusculum bavaricum]
WP_002725405.1	166	rubrerythrin [Phaeospirillum molischianum]
WP_042696678.1	166	rubrerythrin [Methanocorpusculum bavaricum]
WP_047000645.1	166	reverse rubrerythrin-1 [Clostridium sp. C8]
WP_037466081.1	166	hypothetical protein [Smithella sp. F21]
WP_018591361.1	166	"reverse rubrerythrin-1, partial [Terrisporobacter glycolicus]"
WP_049180332.1	166	"reverse rubrerythrin-1, partial [Clostridium botulinum]"
WP_006362454.1	166	MULTISPECIES: rubrerythrin [Slackia]

WP_021427185.1 166   "reverse rubrerythrin-1, partial [Peptoclostridium difficile]"
WP_042258594.1 166   "reverse rubrerythrin-1, partial [Clostridium perfringens]"
KPK23400.1   166   hypothetical protein AMJ61_15820 [Desulfobacterales bacterium SG8_35_2]
WP_013504695.1 166   hypothetical protein [Desulfurispirillum indicum]
WP_048152458.1 165   rubrerythrin [Thermococcus cleftensis]
WP_025323592.1 165   rubrerythrin [Deferrisoma camini]
WP_049674699.1 165   rubrerythrin [Desulfocarbo indianensis]
WP_013258072.1 165   rubrerythrin [Desulfarculus baarsii]
WP_010936184.1 165   rubrerythrin [Dehalococcoides mccartyi]
WP_020880966.1 165   rubrerythrin [Desulfovibrio sp. X2]
WP_054869501.1 165   rubrerythrin [Methanosarcina mazei]
KSV18777.1   165   rubrerythrin [Dehalococcoides mccartyi]
WP_023651956.1 165   rubrerythrin-like protein [Dehalococcoides mccartyi]
WP_041341307.1 165   rubrerythrin [Dehalococcoides mccartyi]
BAS31457.1   165   rubrerythrin [Dehalococcoides mccartyi IBARAKI]
KPL79743.1   165   rubrerythrin [Levilinea saccharolytica]
WP_012984175.1 165   rubrerythrin [Dehalococcoides mccartyi]
WP_048178756.1 165   rubrerythrin [Methanosarcina sp. MTP4]
WP_034376723.1 165   rubrerythrin [Dehalococcoides mccartyi]
WP_011928828.1 165   rubrerythrin [Dehalococcoides mccartyi]
WP_012881680.1 165   MULTISPECIES: rubrerythrin [Dehalococcoides]
WP_008515857.1 165   rubrerythrin [Dethiobacter alkaliphilus]
WP_013559280.1 165   rubrerythrin [Anaerolinea thermophila]
WP_011744791.1 165   rubrerythrin [Chlorobium phaeobacteroides]
WP_028051455.1 165   rubrerythrin [Carboxydothermus ferrireducens]
WP_012466562.1 165   rubrerythrin [Chlorobium limicola]
WP_042316131.1 165   rubrerythrin [Desulfotomaculum acetoxidans]
ABK14533.1   165   Rubrerythrin [Methanosaeta thermophila PT]
KJS17554.1   165   rubrerythrin [Peptococcaceae bacterium BRH_c4b]
WP_011367301.1 165   rubrerythrin [Desulfovibrio alaskensis]
WP_005659502.1 165   rubrerythrin [Dethiosulfovibrio peptidovorans]
WP_011343668.1 165   rubrerythrin [Carboxydothermus hydrogenoformans]
WP_028321389.1 165   rubrerythrin [Desulfatiglans anilini]
WP_040872871.1 165   rubrerythrin [delta proteobacterium NaphS2]
GAP22457.1   165   rubrerythrin [Leptolinea tardivitalis]
WP_033378817.1 165   rubrerythrin [Hippea alviniae]
WP_021759334.1 165   rubrerythrin [Desulfovibrio gigas]
WP_011372372.1 165   rubrerythrin [Sulfurimonas denitrificans]
WP_037466933.1 165   rubrerythrin [Smithella sp. F21]
WP_029476621.1 165   rubrerythrin [Dehalococcoidia bacterium SCGC AB-539-J10]
WP_013162771.1 165   rubrerythrin [Desulfurivibrio alkaliphilus]

WP_015334717.1	165	rubrerythrin [Desulfovibrio hydrothermalis]
WP_013516189.1	165	rubrerythrin [Desulfovibrio aespoeensis]
WP_013006779.1	165	rubrerythrin [Deferribacter desulfuricans]
WP_040620135.1	165	rubrerythrin [Smithella sp. ME-1]
WP_044927286.1	165	MULTISPECIES: rubrerythrin [Coprobacillus]
WP_015415400.1	165	rubrerythrin [Desulfovibrio piezophilus]
WP_014322929.1	165	rubrerythrin [Desulfovibrio desulfuricans]
WP_015852578.1	165	rubrerythrin [Desulfovibrio salexigens]
WP_041584958.1	165	rubrerythrin [Syntrophus aciditrophicus]
WP_013719306.1	165	rubrerythrin [Methanosaeta concilii]
WP_044600109.1	165	rubrerythrin [Candidatus Stoquefichus massiliensis]
WP_027722992.1	165	rubrerythrin [Desulfovibrio zosterae]
WP_050635874.1	165	rubrerythrin [Candidatus Stoquefichus sp. SB1]
WP_014489123.1	165	rubrerythrin [Brachyspira intermedia]
WP_013113764.1	165	rubrerythrin [Brachyspira murdochii]
WP_020005329.1	165	rubrerythrin [Brachyspira innocens]
WP_008721296.1	165	rubrerythrin [Brachyspira hampsonii]
WP_014933721.1	165	rubrerythrin [Brachyspira pilosicoli]
ELV05465.1	165	rubrerythrin [Brachyspira hampsonii 30599]
WP_012671462.1	165	rubrerythrin [Brachyspira hyodysenteriae]
WP_048593237.1	165	rubrerythrin [Brachyspira suanatina]
WP_006299751.1	165	rubrerythrin [Aminomonas paucivorans]
WP_041747618.1	165	rubrerythrin [Brachyspira pilosicoli]
WP_050754557.1	165	MULTISPECIES: rubrerythrin [Erysipelotrichaceae]
WP_028328767.1	165	rubrerythrin [Brachyspira alvinipulli]
KPK27344.1	165	rubrerythrin [Nitrospira bacterium SG8_3]
WP_023379468.1	165	rubrerythrin [Smithella sp. ME-1]
KFO67048.1	165	hypothetical protein ER57_13680 [Smithella sp. SCADC]
KQC09507.1	165	hypothetical protein APR62_14055 [Smithella sp. SDB]
WP_011485715.1	165	rubrerythrin [Polaromonas sp. JS666]
WP_051039317.1	165	hypothetical protein [Fischerella muscicola]
EQE19820.1	165	"reverse rubrerythrin-1, partial [Peptoclostridium difficile CD17]"
EQF85933.1	165	"reverse rubrerythrin-1, partial [Peptoclostridium difficile 824]"
WP_028534255.1	165	hypothetical protein [Paludibacterium yongneupense]
WP_012663569.1	165	bacterioferritin [Nautilia profundicola]
WP_036802802.1	165	ferritin [Planomicrobium glaciei]
AAB89608.1	164	rubrerythrin (rr3) [Archaeoglobus fulgidus DSM 4304]
KPQ44480.1	164	rubrerythrin [Candidatus Methanoperedens sp. BLZ1]
WP_012545398.1	164	MULTISPECIES: rubrerythrin [Thermodesulfovibrio]
GAP07409.1	164	rubrerythrin [Anaerolinea thermolimosa]

WP_007527771.1	164	rubrerythrin [Desulfovibrio sp. A2]
WP_035069165.1	164	rubrerythrin [Desulfovibrio termitidis]
WP_045212385.1	164	rubrerythrin [Desulfonatronovibrio magnus]
WP_028894877.1	164	rubrerythrin [Syntrophorhabdus aromaticivorans]
KPK45807.1	164	rubrerythrin [Nitrospira bacterium SM23_35]
WP_015946789.1	164	rubrerythrin [Desulfovibrio vulgaris]
KPJ99002.1	164	rubrerythrin [Nitrospira bacterium SG8_35_4]
WP_028844256.1	164	rubrerythrin [Thermodesulfovibrio thiophilus]
KJS29470.1	164	rubrerythrin [Desulfatitalea sp. BRH_c12]
WP_007290354.1	164	rubrerythrin [Thermosinus carboxydivorans]
KPK36146.1	164	rubrerythrin [Nitrospira bacterium SG8_35_1]
WP_027175895.1	164	rubrerythrin [Desulfovibrio aminophilus]
WP_041534415.1	164	rubrerythrin [Petrotoga mobilis]
CBX28442.1	164	hypothetical protein N47_G37660 [uncultured Desulfobacterium sp.]
WP_027368112.1	164	rubrerythrin [Desulfovibrio africanus]
KHE91175.1	164	rubrerythrin [Candidatus Scalindua brodae]
WP_005989355.1	164	rubrerythrin [Desulfovibrio africanus]
CCZ22059.1	164	rubrerythrin [Acetobacter sp. CAG:977]
WP_014259552.1	164	rubrerythrin [Desulfovibrio africanus]
WP_020887209.1	164	rubrerythrin [Desulfovibrio alkalitolerans]
WP_015752071.1	164	rubrerythrin [Desulfohalobium retbaense]
WP_031386858.1	164	rubrerythrin [Desulfonatronum thiodismutans]
WP_027371531.1	164	rubrerythrin [Desulfovermiculus halophilus]
WP_028571493.1	164	rubrerythrin [Desulfonatronum lacustre]
WP_028575300.1	164	rubrerythrin [Desulfonatronovibrio hydrogenovorans]
WP_008868581.1	164	rubrerythrin [Desulfonatronospira thiodismutans]
WP_014793708.1	164	rubrerythrin [Desulfitobacterium dehalogenans]
WP_014809875.1	164	rubrerythrin [Desulfomonile tiedjei]
WP_019850253.1	164	rubrerythrin [Desulfitobacterium sp. PCE1]
WP_010939592.1	164	rubrerythrin [Desulfovibrio vulgaris]
WP_045221140.1	164	rubrerythrin [Desulfonatronum thioautotrophicum]
WP_047808554.1	164	rubrerythrin [Desulfosporosinus acididurans]
WP_014254212.1	164	rubrerythrin [[Clostridium] clariflavum]
WP_043638769.1	164	rubrerythrin [Desulfovibrio sp. TomC]
WP_005992438.1	164	rubrerythrin [Desulfovibrio fructosivorans]
WP_027185433.1	164	rubrerythrin [Desulfovibrio inopinatus]
WP_028990822.1	164	rubrerythrin [Thermacetogenium phaeum]
WP_027177668.1	164	rubrerythrin [Desulfovibrio bastinii]
WP_047776993.1	164	rubrerythrin [[Eubacterium] fissicatena]
WP_027191310.1	164	rubrerythrin [Desulfovibrio putealis]
EKO38955.1	164	rubrerythrin [Desulfovibrio magneticus str. Maddingley MBC34]
WP_022661365.1	164	rubrerythrin [Desulfovibrio longus]
WP_050741777.1	164	rubrerythrin [Acetobacterium bakii]
WP_015860664.1	164	rubrerythrin [Desulfovibrio magneticus]

WP_019878545.1	164	hypothetical protein [Succinispira mobilis]
KPK26983.1	164	rubrerythrin [Desulfobacterales bacterium SG8_35_2]
WP_009109085.1	164	rubrerythrin [Desulfovibrio sp. U5L]
WP_009179536.1	164	rubrerythrin [Desulfovibrio sp. FW1012B]
WP_013707102.1	164	rubrerythrin [Desulfobacca acetoxidans]
WP_021168646.1	164	MULTISPECIES: rubrerythrin [Sporomusa]
WP_027186777.1	164	rubrerythrin [Desulfovibrio cuneatus]
WP_024826379.1	164	rubrerythrin [Desulfovibrio magneticus]
WP_027355173.1	164	rubrerythrin [Desulfosarcina sp. BuS5]
WP_012158491.1	164	rubrerythrin [Alkaliphilus oremlandii]
WP_015262016.1	164	rubrerythrin [Desulfitobacterium dichloroeliminans]
WP_007957397.1	164	rubrerythrin [Pelosinus fermentans]
WP_028577094.1	164	rubrerythrin [Desulfomicrobium escambiense]
KPJ99622.1	164	rubrerythrin [Desulfobacterales bacterium SG8_35]
WP_007785527.1	164	rubrerythrin [Desulfosporosinus youngiae]
WP_007937890.1	164	MULTISPECIES: rubrerythrin [Pelosinus]
WP_019999322.1	164	MULTISPECIES: rubrerythrin [Desulfovibrio]
WP_026393673.1	164	rubrerythrin [Acetobacterium dehalogenans]
WP_053964747.1	164	rubrerythrin [Clostridiales bacterium mt11]
WP_029459899.1	164	rubrerythrin [Desulfovibrio alcoholivorans]
WP_014354813.1	164	rubrerythrin [Acetobacterium woodii]
WP_015774642.1	164	rubrerythrin [Desulfomicrobium baculatum]
WP_013113702.1	164	rubrerythrin [Brachyspira murdochii]
WP_009624457.1	164	rubrerythrin [Desulfosporosinus sp. OT]
WP_014186330.1	164	rubrerythrin [Desulfosporosinus orientis]
WP_020005171.1	164	rubrerythrin [Brachyspira innocens]
WP_018213067.1	164	rubrerythrin [Desulfitobacterium hafniense]
WP_019229433.1	164	rubrerythrin [Sedimentibacter sp. B4]
WP_005814501.1	164	rubrerythrin [Desulfitobacterium hafniense]
WP_011459575.1	164	rubrerythrin [Desulfitobacterium hafniense]
GAP41044.1	164	rubrerythrin [Anaerolineaceae bacterium TC1]
WP_028329148.1	164	rubrerythrin [Brachyspira alvinipulli]
WP_031482060.1	164	rubrerythrin [Desulfovibrio frigidus]
WP_028587981.1	164	rubrerythrin [Desulfocurvus vexinensis]
WP_022667400.1	164	rubrerythrin [Desulfospira joergensenii]
WP_018232473.1	164	rubrerythrin [Thioalkalivibrio thiocyanodenitrificans]
WP_012637259.1	164	rubrerythrin [Thioalkalivibrio sulfidiphilus]
WP_018954309.1	164	rubrerythrin [Thioalkalivibrio sulfidiphilus]
WP_009139809.1	164	rubrerythrin [Slackia piriformis]
EQK60961.1	164	"reverse rubrerythrin-1, partial [Clostridium difficile F200]"
WP_051833736.1	164	hypothetical protein [Streptomyces sp. NRRL S-646]
WP_024790430.1	164	bacterioferritin [Lebetimonas sp. JH292]
WP_022063714.1	164	bacterioferritin [Alistipes inops]
WP_048141467.1	163	MULTISPECIES: rubrerythrin [Methanosarcina]

WP_048136233.1 163   MULTISPECIES: rubrerythrin [Methanosarcina]
WP_011020686.1 163   rubrerythrin [Methanosarcina acetivorans]
WP_048169878.1 163   rubrerythrin [Methanosarcina siciliae]
WP_048129763.1 163   MULTISPECIES: rubrerythrin [Methanosarcina]
WP_048171354.1 163   rubrerythrin [Methanosarcina sp. 2.H.A.1B.4]
KKO18075.1   163   rubrerythrin [Candidatus Brocadia fulgida]
WP_013120444.1 163   rubrerythrin [Thermincola potens]
WP_007221461.1 163   rubrerythrin [Candidatus Jettenia caeni]
WP_054297985.1 163   rubrerythrin [Methanosarcina sp. E03.2]
WP_010876457.1 163   MULTISPECIES: rubrerythrin
[Methanothermobacter]
WP_048124565.1 163   rubrerythrin [Methanosarcina lacustris]
WP_015052984.1 163   rubrerythrin [Methanolobus psychrophilus]
WP_013898808.1 163   rubrerythrin [Methanosalsum zhilinae]
WP_015325046.1 163   rubrerythrin [Methanomethylovorans hollandica]
WP_048039058.1 163   rubrerythrin [Methanosarcina mazei]
WP_048064557.1 163   rubrerythrin [Archaeoglobus fulgidus]
WP_048038283.1 163   rubrerythrin [Methanosarcina mazei]
WP_048045730.1 163   rubrerythrin [Methanosarcina mazei]
WP_048040732.1 163   rubrerythrin [Methanosarcina mazei]
WP_052562390.1 163   rubrerythrin [Candidatus Brocadia sinica]
WP_048205652.1 163   rubrerythrin [Methanococcoides methylutens]
WP_048142095.1 163   rubrerythrin [Methanosarcina horonobensis]
WP_023845987.1 163   rubrerythrin [Methanolobus tindarius]
WP_013036917.1 163   rubrerythrin [Methanohalophilus mahii]
WP_048166711.1 163   rubrerythrin [Methanosarcina thermophila]
WP_048044107.1 163   rubrerythrin [Methanosarcina mazei]
WP_048194941.1 163   rubrerythrin [Methanococcoides methylutens]
WP_048036869.1 163   MULTISPECIES: rubrerythrin [Methanosarcina]
WP_013194786.1 163   rubrerythrin [Methanohalobium evestigatum]
WP_003543418.1 163   rubrerythrin [Desulfotomaculum nigrificans]
WP_013810265.1 163   rubrerythrin [Desulfotomaculum
carboxydivorans]
WP_052216807.1 163   rubrerythrin [Thermincola ferriacetica]
WP_013296031.1 163   rubrerythrin [Methanothermobacter
marburgensis]
WP_013645715.1 163   rubrerythrin [Methanobacterium lacus]
WP_013825701.1 163   rubrerythrin [Methanobacterium paludis]
WP_048080740.1 163   MULTISPECIES: rubrerythrin [Methanobacterium]
EKE10843.1   163   rubrerythrin [uncultured bacterium]
WP_048073524.1 163   rubrerythrin [Methanobacterium formicicum]
KKT88085.1   163   Rubrerythrin [Parcubacteria (Moranbacteria)
bacterium GW2011_GWC2_45_10]
WP_048085310.1 163   rubrerythrin [Methanobacterium formicicum]
CEL24457.1   163   rubrerythrin [Methanobacterium formicicum]
WP_013645504.1 163   rubrerythrin [Methanobacterium lacus]
WP_048190143.1 163   rubrerythrin [Methanobacterium sp. SMA-27]
WP_011499127.1 163   rubrerythrin [Methanococcoides burtonii]
WP_011699316.1 163   rubrerythrin [Syntrophobacter fumaroxidans]
WP_028307879.1 163   rubrerythrin [Desulfitibacter alkalitolerans]

WP_038667599.1	163	rubrerythrin [Pelosinus sp. UFO1]
WP_004031857.1	163	rubrerythrin [Methanobacterium formicicum]
EKQ55832.1	163	rubrerythrin [Methanobacterium sp. Maddingley MBC34]
WP_013841597.1	163	rubrerythrin [Desulfotomaculum ruminis]
WP_006584377.1	163	rubrerythrin [Thermanaerovibrio velox]
WP_013825223.1	163	rubrerythrin [Methanobacterium paludis]
WP_008409630.1	163	Nigerythrin [Desulfotomaculum hydrothermale]
WP_053955337.1	163	rubrerythrin [Clostridiaceae bacterium mt12]
ELV06683.1	163	rubrerythrin [Brachyspira hampsonii 30599]
KHE91955.1	163	rubrerythrin [Candidatus Scalindua brodae]
WP_034601167.1	163	rubrerythrin [Desulfosporosinus sp. HMP52]
WP_014903967.1	163	rubrerythrin [Desulfosporosinus meridiei]
GAE17444.1	163	rubrerythrin [Bacteroides pyogenes DSM 20611 = JCM 6294]
GAE82992.1	163	rubrerythrin [Bacteroides reticulotermitis JCM 10512]
KDS14827.1	163	reverse rubrerythrin-1 [Bacteroides ovatus str. 3725 D1 iv]
WP_021406060.1	163	"reverse rubrerythrin-1, partial [Peptoclostridium difficile]"
WP_034279238.1	163	rubrerythrin [Alkanindiges illinoisensis]
WP_034421862.1	162	rubrerythrin [Clostridiales bacterium DRI-13]
WP_005026000.1	162	MULTISPECIES: rubrerythrin [Bilophila]
KJS21508.1	162	rubrerythrin [Clostridiaceae bacterium BRH_c20a]
WP_008710299.1	162	MULTISPECIES: rubrerythrin [Synergistaceae]
KJS83102.1	162	rubrerythrin [Peptococcaceae bacterium BICA1-8]
WP_054325105.1	162	rubrerythrin [Clostridia bacterium UC5.1-1E3]
CAA10002.1	162	rubrerythrin [Clostridium acetobutylicum]
WP_020064548.1	162	rubrerythrin [Brachyspira hyodysenteriae]
WP_048593286.1	162	rubrerythrin [Brachyspira suanatina]
WP_014489071.1	162	rubrerythrin [Brachyspira intermedia]
WP_008721198.1	162	rubrerythrin [Brachyspira hampsonii]
WP_047116386.1	162	rubrerythrin [Brachyspira hyodysenteriae]
WP_038485928.1	162	rubrerythrin [Collimonas arenae]
EYB13488.1	162	reverse rubrerythrin-1 [Bacteroides fragilis str. S38L3]
WP_021364076.1	162	"reverse rubrerythrin-1, partial [Peptoclostridium difficile]"
WP_051458684.1	162	hypothetical protein [Microbispora sp. ATCC PTA-5024]
WP_021361299.1	162	"MULTISPECIES: reverse rubrerythrin-1, partial[Peptoclostridium]"
WP_021363132.1	162	"reverse rubrerythrin-1, partial [Peptoclostridium difficile]"
WP_021368853.1	162	"reverse rubrerythrin-1, partial [Peptoclostridium difficile]"
WP_036807682.1	162	rubrerythrin [Polaromonas sp. CG9_12]

WP_021363133.1	162	"reverse rubrerythrin-1, partial [Peptoclostridium difficile]"
WP_013626212.1	161	rubrerythrin [Syntrophobotulus glycolicus]
WP_020492729.1	161	rubrerythrin [Dehalobacter sp. FTH1]
WP_019226078.1	161	MULTISPECIES: rubrerythrin [Dehalobacter]
WP_015042799.1	161	MULTISPECIES: rubrerythrin [Dehalobacter]
WP_045573045.1	161	rubrerythrin [Desulfosporosinus sp. I2]
WP_037974748.1	161	rubrerythrin [Synergistes jonesii]
KQC08978.1	161	"rubrerythrin, partial [Smithella sp. SDB]"
WP_011239234.1	161	MULTISPECIES: rubrerythrin [Rhodocyclaceae]
WP_050415911.1	161	rubrerythrin [Azoarcus sp. CIB]
WP_011903215.1	161	rubrerythrin [Polynucleobacter necessarius]
WP_015421332.1	161	rubrerythrin [beta proteobacterium CB]
WP_018992010.1	161	rubrerythrin [Azoarcus toluclasticus]
WP_035038978.1	161	rubrerythrin [Aquabacterium sp. NJ1]
WP_011143723.1	161	rubrerythrin [Gloeobacter violaceus]
WP_015436017.1	161	rubrerythrin/nigerythrin-like protein [Azoarcus sp.KH32C]
WP_023457583.1	161	hypothetical protein [Asticcacaulis sp. AC466]
BAA30155.1	161	161aa long hypothetical protein [Pyrococcus horikoshii OT3]
EDS04434.1	160	Rubrerythrin [Alistipes putredinis DSM 17216]
WP_021388415.1	160	"reverse rubrerythrin-1, partial [Peptoclostridium difficile]"
WP_011462424.1	160	rubrerythrin [Rhodoferax ferrireducens]
WP_029708264.1	160	rubrerythrin [Rhodoferax saidenbachensis]
CBA30746.1	160	hypothetical protein Csp_C25100 [Curvibacter putative symbiont of Hydra magnipapillata]
WP_037514667.1	160	rubrerythrin [Sphingobium xenophagum]
WP_021388484.1	160	"reverse rubrerythrin-1, partial [Peptoclostridium difficile]"
WP_046865787.1	160	bacterioferritin [Microvirga sp. JC119]
CAI82571.1	159	rubrerythrin [Dehalococcoides mccartyi CBDB1]
WP_009870344.1	159	rubrerythrin [Magnetospirillum magnetotacticum]
WP_018152332.1	159	rubrerythrin [Leeia oryzae]
CBK62988.1	159	Rubrerythrin [Alistipes shahii WAL 8301]
KJS39043.1	159	rubrerythrin [Hyphomonas sp. BRH_c22]
WP_051693705.1	159	rubrerythrin [Thioclava dalianensis]
WP_014725839.1	159	MULTISPECIES: rubrerythrin [Burkholderia]
WP_028215273.1	159	rubrerythrin [Burkholderia mimosarum]
WP_028362902.1	159	rubrerythrin [Burkholderia sp. JPY366]
WP_043060760.1	159	MULTISPECIES: rubrerythrin [Sphingomonas]
WP_018083864.1	159	hypothetical protein [Asticcacaulis benevestitus]
WP_011880104.1	159	rubrerythrin [Burkholderia vietnamiensis]
WP_038716035.1	159	rubrerythrin [Burkholderia sp. lig30]
WP_042299682.1	159	rubrerythrin [Burkholderia kururiensis]
WP_026121075.1	159	rubrerythrin [Burkholderia kururiensis]
WP_026913138.1	159	rubrerythrin [Perlucidibaca piscinae]

WP_022583294.1	159	"reverse rubrerythrin-1, partial [Peptoclostridium difficile]"
KJS65685.1	158	rubrerythrin [Peptococcaceae bacterium BICA1-7]
ACV64248.1	158	Rubrerythrin [Desulfotomaculum acetoxidans DSM 771]
CCM63087.1	158	Rubrerythrin [Candidatus Microthrix parvicella RN1]
EAW30272.1	158	Rubrerythrin [marine gamma proteobacterium HTCC2143]
WP_046020224.1	158	rubrerythrin [Magnetospira sp. QH-2]
WP_011385790.1	158	rubrerythrin [Magnetospirillum magneticum]
WP_008614666.1	158	rubrerythrin [Magnetospirillum sp. SO-1]
WP_057289674.1	158	rubrerythrin [Noviherbaspirillum sp. Root189]
WP_029134682.1	158	rubrerythrin [Sedimenticola selenatireducens]
WP_014100396.1	158	rubrerythrin [Chloracidobacterium thermophilum]
WP_019141698.1	158	hypothetical protein [Herbaspirillum massiliense]
WP_014802001.1	158	rubrerythrin [Turneriella parva]
WP_011993053.1	158	rubrerythrin [Xanthobacter autotrophicus]
EXS70887.1	158	rubrerythrin [Sphingobium sp. Ant17]
WP_033965228.1	158	rubrerythrin [Sphingomonas sp. Ant H11]
KIU26858.1	158	rubrerythrin [Sphingomonas taxi]
WP_024342668.1	158	MULTISPECIES: rubrerythrin [Bradyrhizobium]
WP_055281205.1	158	rubrerythrin [Thermococcus sp. EP1]
WP_048053292.1	158	rubrerythrin [Pyrococcus horikoshii]
AKB25163.1	157	Rubrerythrin [Methanosarcina sp. MTP4]
WP_052571646.1	157	rubrerythrin [Geothrix fermentans]
WP_013337342.1	157	rubrerythrin [Vulcanisaeta distributa]
WP_013604022.1	157	rubrerythrin [Vulcanisaeta moutnovskia]
WP_028328776.1	157	rubrerythrin [Brachyspira alvinipulli]
WP_040041490.1	157	rubrerythrin [Herbaspirillum sp. TSA66]
KJR72429.1	157	rubrerythrin [Vulcanisaeta sp. AZ3]
WP_011312829.1	157	rubrerythrin [Thiobacillus denitrificans]
WP_018078062.1	157	rubrerythrin [Thiobacillus denitrificans]
WP_018506997.1	157	rubrerythrin [Thiobacillus thioparus]
WP_031404414.1	157	rubrerythrin [Thiomonas sp. FB-Cd]
WP_042258990.1	157	"reverse rubrerythrin-1, partial [Clostridium perfringens]"
WP_011436200.1	157	MULTISPECIES: rubrerythrin [Frankia]
WP_031006584.1	157	hypothetical protein [Streptomyces sp. NRRL WC-3773]
WP_050502351.1	157	hypothetical protein [Streptomyces monomycini]
WP_006785915.1	156	"rubrerythrin, partial [Turicibacter sanguinis]"
KKU54706.1	156	Rubrerythrin [Parcubacteria (Moranbacteria) bacterium GW2011_GWE2_47_10]
KJR74456.1	156	rubrerythrin [Thermoproteus sp. AZ2]
WP_012185133.1	156	rubrerythrin [Caldivirga maquilingensis]

WP_013294313.1 156   rubrerythrin [Gallionella capsiferriformans]
GAO35378.1   156   rubrerythrin [Sulfuricella sp. T08]
WP_051756320.1 156   hypothetical protein [Nocardia sp. NRRL WC-3656]
WP_013679694.1 156   rubrerythrin [Thermoproteus uzoniensis]
KER11151.1   156   rubrerythrin [Chlorobium sp. GBChlB]
EHG31504.1   156   "hypothetical protein HMPREF9467_02604, partial [, partial[[Clostridium] clostridioforme 2_1_49FAA]"
WP_027635568.1 156   "reverse rubrerythrin-1, partial [Clostridium butyricum]"
WP_011522081.1 156   bacterioferritin [Candidatus Koribacter versatilis]
WP_006784929.1 155   "rubrerythrin, partial [Turicibacter sanguinis]"
WP_042258237.1 155   "reverse rubrerythrin-1, partial [Clostridium perfringens]"
GAO32588.1   155   rubrerythrin [Ferriphaselus amnicola]
WP_043150422.1 155   rubrerythrin [Sphingobium sp. Ant17]
WP_012286452.1 155   rubrerythrin [Caulobacter sp. K31]
WP_042172505.1 155   rubrerythrin [Streptomyces sp. NBRC 110035]
EFL39891.1   155   rubrerythrin [Streptomyces griseoflavus Tu4000]
EAX48399.1   155   Rubredoxin-type Fe(Cys)4 protein [Thermosinus carboxydivorans Nor1]
AJF34516.1   155   rubrerythrin [Streptomyces sp. JCM 9888]
WP_009205171.1 154   rubrerythrin [Sulfuricella denitrificans]
WP_050790015.1 154   hypothetical protein [Mycobacterium parascrofulaceum]
WP_052955313.1 154   hypothetical protein [Mycobacterium nebraskense]
EQK72756.1   154   reverse rubrerythrin-1 [Clostridium difficile CD90]
EQK70397.1   154   reverse rubrerythrin-1 [Clostridium difficile CD90]
WP_021414809.1 154   "reverse rubrerythrin-1, partial [Peptoclostridium difficile]"
WP_051676974.1 153   rubrerythrin [Desulfovibrio frigidus]
ACN84478.1   153   rubrerythrin [Brachyspira hyodysenteriae WA1]
WP_055292553.1 153   "reverse rubrerythrin-1, partial [Clostridium disporicum]"
AAC28312.1   153   rubrerythrin/nigerythrin-like protein [Spirillum volutans]
EQH50765.1   153   reverse rubrerythrin-1 [Clostridium difficile DA00246]
EQH65514.1   153   reverse rubrerythrin-1 [Clostridium difficile DA00273]
WP_053784159.1 153   hypothetical protein [Paenibacillus sp. A59]
KON31042.1   152   rubrerythrin [miscellaneous Crenarchaeota group-1 archaeon SG8-32-3]
WP_055494260.1 152   hypothetical protein [Streptomyces sp. TP-

A0356]
KHF24740.1   151   rubrerythrin [Solemya velum gill symbiont]
AEE93928.1   151   Rubrerythrin [Acidianus hospitalis W1]
CUQ51039.1   151   NADH peroxidase [Prevotella copri]
WP_027747472.1 151   rubrerythrin [Streptomyces sp. CNH287]
KQC13660.1   150   rubrerythrin [Desulfuromonas sp. SDB]
WP_027302291.1 150   rubrerythrin [Rhodospirillales bacterium
URHD0088]
WP_034868608.1 150   hypothetical protein [Clostridium lundense]
WP_021418036.1 150   "reverse rubrerythrin-1, partial
[Peptoclostridium difficile]"
WP_021418035.1 150   "reverse rubrerythrin-1, partial
[Peptoclostridium difficile]"
WP_046058578.1 150   "reverse rubrerythrin-1, partial [Clostridium
sp.IBUN125C]"
KKC04101.1   150   "hypothetical protein WU83_15505, partial
[Mycobacterium nebraskense]"
WP_018955481.1 150   rubrerythrin [Streptomyces sp. CNB091]
WP_012378165.1 150   rubrerythrin [Streptomyces griseus]
WP_003964790.1 150   rubrerythrin [Streptomyces sp. ACT-1]
WP_057661269.1 150   rubrerythrin [Streptomyces anulatus]
WP_050357462.1 150   rubrerythrin [Streptomyces europaeiscabiei]
WP_056699138.1 150   MULTISPECIES: rubrerythrin [Streptomyces]
KRT72376.1   149   "rbr, rubrerythrin [bacterium CSP1-6]"
ETZ06992.1   149   rubrerythrin [Holospora obtusa F1]
EIB19227.1   149   non-heme iron protein [Campylobacter jejuni
subsp. jejuni 51494]
WP_032544991.1 149   "reverse rubrerythrin-1, partial
[Peptoclostridium difficile]"
WP_042263796.1 149   "reverse rubrerythrin-1, partial [Clostridium
butyricum]"
AKB27274.1   148   Rubrerythrin [Methanosarcina siciliae T4/M]
WP_040458481.1 148   rubrerythrin [Holospora obtusa]
EHG31578.1   148   "hypothetical protein HMPREF9467_02597,
partial [, partial[[Clostridium] clostridioforme 2_1_49FAA]"
WP_003524882.1 148   "hypothetical protein, partial
[[Clostridium]clostridioforme]"
EQG84553.1   147   reverse rubrerythrin-1 [Clostridium difficile
DA00183]
EQH17190.1   147   reverse rubrerythrin-1 [Clostridium difficile
DA00212]
EQH64168.1   147   reverse rubrerythrin-1 [Clostridium difficile
DA00275]
EQG79344.1   147   reverse rubrerythrin-1 [Clostridium difficile
DA00183]
EQH36677.1   147   reverse rubrerythrin-1 [Clostridium difficile
DA00232]
EQH23792.1   147   reverse rubrerythrin-1 [Clostridium difficile
DA00212]
EQH24718.1   147   reverse rubrerythrin-1 [Clostridium difficile

DA00211]
WP_009742898.1 146   rubrerythrin [Frankia sp. QA3]
WP_011437496.1 146   MULTISPECIES: rubrerythrin [Frankia]
WP_011606224.1 146   MULTISPECIES: rubrerythrin [Frankia]
WP_020459414.1 146   rubrerythrin [Frankia sp. EAN1pec]
WP_031933734.1 146   rubrerythrin [Candidatus Hepatobacter penaei]
WP_018501972.1 146   rubrerythrin [Frankia sp. BCU110501]
WP_026310774.1 146   rubrerythrin [Frankia sp. BMG5.12]
WP_054566699.1 146   rubrerythrin [Frankia sp. R43]
WP_006543966.1 146   rubrerythrin [Frankia sp. EUN1f]
EXY30933.1   146   "rubrerythrin family protein, partial [Bacteroides fragilis str. 3397 T10]"
WP_042274955.1 146   "reverse rubrerythrin-1, partial [Clostridium dakarense]"
WP_042274927.1 146   "reverse rubrerythrin-1, partial [Clostridium dakarense]"
KLU72979.1   146   hypothetical protein RHS_1221 [Robinsoniella sp. RHS]
WP_012309233.1 146   ferritin [Candidatus Korarchaeum cryptofilum]
CUS77722.1   145   Rubrerythrin [bacterium JGI-20]
CUS96407.1   145   Rubrerythrin [bacterium JGI-24]
WP_047133438.1 145   rubrerythrin [bacterium JGI-5]
WP_044887264.1 145   MULTISPECIES: rubrerythrin [Frankia]
WP_046329724.1 145   rubrerythrin [Polynucleobacter necessarius]
WP_007088410.1 145   MULTISPECIES: rubrerythrin [Thalassospira]
WP_021393665.1 145   "reverse rubrerythrin-1, partial [Peptoclostridium difficile]"
WP_009988550.1 144   rubrerythrin [Sulfolobus solfataricus]
WP_010980455.1 144   rubrerythrin [Sulfolobus tokodaii]
WP_012991978.1 144   rubrerythrin [Thermocrinis albus]
WP_012719271.1 144   rubrerythrin [Sulfolobus islandicus]
WP_012712582.1 144   rubrerythrin [Sulfolobus islandicus]
WP_015581689.1 144   rubrerythrin [Sulfolobus islandicus]
WP_011279060.1 144   rubrerythrin [Sulfolobus acidocaldarius]
WP_013874387.1 144   MULTISPECIES: rubrerythrin [Frankia]
WP_009072887.1 144   rubrerythrin [Metallosphaera yellowstonensis]
WP_015419492.1 144   MULTISPECIES: rubrerythrin [Hydrogenobaculum]
4DI0   144   "Chain A, The Structure Of Rubrerythrin From Burkholderia Pseudomallei"
WP_023492463.1 144   MULTISPECIES: rubrerythrin [Holospora]
WP_012513942.1 144   rubrerythrin [Hydrogenobaculum sp. Y04AAS1]
WP_023491195.1 144   rubrerythrin [Holospora undulata]
WP_045875567.1 144   rubrerythrin [Frankia sp. DC12]
WP_007516289.1 144   rubrerythrin [Frankia sp. CN3]
WP_047154773.1 144   rubrerythrin [Aneurinibacillus tyrosinisolvens]
WP_013422967.1 144   rubrerythrin [Frankia sp. EuI1c]
WP_027416763.1 144   rubrerythrin [Aneurinibacillus terranovensis]
WP_013683332.1 144   hypothetical protein [Archaeoglobus veneficus]
WP_008888879.1 144   rubrerythrin [Thalassospira profundimaris]

WP_044830049.1 144   rubrerythrin [Thalassospira sp. HJ]
EPZ58665.1   144   rubrerythrin-1 [[[Clostridium] sordellii ATCC 9714]
WP_032085513.1 144   bacterioferritin [Bacillus aquimaris]
WP_011921441.1 143   rubrerythrin [Metallosphaera sedula]
WP_048099261.1 143   rubrerythrin [Candidatus Acidianus copahuensis]
WP_025306238.1 143   rubrerythrin [Thermocrinis ruber]
WP_029551412.1 143   rubrerythrin [Thermocrinis sp. GBS]
WP_013738372.1 143   rubrerythrin [Metallosphaera cuprina]
WP_054836438.1 143   rubrerythrin [Metallosphaera hakonensis]
WP_054838853.1 143   rubrerythrin [Sulfolobus metallicus]
WP_054846390.1 143   rubrerythrin [Sulfolobus sp. JCM 16833]
ESZ86749.1   143   rubrerythrin [Blastomonas sp. CACIA14H2]
WP_048054906.1 143   rubrerythrin [Acidianus hospitalis]
WP_034210400.1 143   rubrerythrin [Arenimonas metalli]
WP_040824165.1 143   rubrerythrin [marine gamma proteobacterium HTCC2143]
WP_010548318.1 143   rubrerythrin [gamma proteobacterium HIMB30]
WP_025747942.1 143   hypothetical protein [Caldicoprobacter oshimai]
WP_049042481.1 143   hypothetical protein [Clostridium botulinum]
WP_003489320.1 143   MULTISPECIES: hypothetical protein [Clostridium]
WP_024933548.1 143   hypothetical protein [Clostridium botulinum]
EKN41266.1   143   rubrerythrin family protein [Clostridium botulinum CFSAN001627]
WP_021107503.1 143   rubrerythrin family protein [Clostridium botulinum]
WP_011948678.1 143   MULTISPECIES: ferritin [Clostridium]
WP_015957760.1 143   hypothetical protein [Clostridium botulinum]
WP_035152758.1 143   "reverse rubrerythrin-1, partial [Clostridium tetanomorphum]"
WP_045885682.1 143   hypothetical protein [Clostridium sporogenes]
WP_003356022.1 143   MULTISPECIES: hypothetical protein [Clostridium]
WP_012342694.1 143   hypothetical protein [Clostridium botulinum]
WP_021390835.1 143   reverse rubrerythrin-1 [Peptoclostridium difficile]
EQH64229.1   143   rubrerythrin-1 [Clostridium difficile DA00256]
WP_012062018.1 143   hypothetical protein [Alkaliphilus metalliredigens]
WP_050607928.1 143   hypothetical protein [Clostridium sp. mt5]
WP_012447566.1 143   hypothetical protein [Natranaerobius thermophilus]
WP_037029513.1 143   "hypothetical protein, partial [Psychrilyobacter atlanticus]"
CDI49983.1   143   rubrerythrin [Clostridium tetani 12124569]
KHO38660.1   143   hypothetical protein OR62_09655 [Clostridium tetani]

KRT72352.1   143   "Ferritin, Dps family protein, bacterioferritin[Deltaproteobacteria bacterium CSP1-8]"
WP_013250530.1 142   rubrerythrin [Nitrospira defluvii]
WP_020734163.1 142   rubrerythrin [Sorangium cellulosum]
KPK27352.1   142   rubrerythrin [Nitrospira bacterium SG8_3]
WP_012234388.1 142   rubrerythrin [Sorangium cellulosum]
WP_012963856.1 142   rubrerythrin [Hydrogenobacter thermophilus]
WP_015593555.1 142   rubrerythrin [Bacillus sp. 1NLA3E]
EKX80123.1   142   rubrerythrin [Clostridium botulinum CFSAN001628]
WP_048602010.1 142   rubrerythrin [Bacillaceae bacterium mt6]
KJR49294.1   142   Rubrerythrin [Desulfosporosinus sp. I2]
WP_033070232.1 142   rubrerythrin [Thalassospira australica]
WP_048096501.1 142   hypothetical protein [Geoglobus ahangari]
WP_048091177.1 142   hypothetical protein [Geoglobus acetivorans]
WP_026475716.1 142   hypothetical protein [Alkaliphilus transvaalensis]
WP_013276812.1 142   hypothetical protein [Thermosediminibacter oceani]
WP_049752544.1 142   hypothetical protein [Candidatus Desulforudis audaxviator]
WP_013866788.1 142   hypothetical protein [Methanothermococcus okinawensis]
WP_022733756.1 142   rubrerythrin [Thalassospira lucentensis]
WP_053963090.1 142   hypothetical protein [Clostridiales bacterium mt11]
EET59573.1   142   Rubrerythrin [Marvinbryantia formatexigens DSM 14469]
WP_039886787.1 142   reverse rubrerythrin-1 [[Clostridium]clostridioforme]
WP_006311269.1 142   rubrerythrin [Caldisalinibacter kiritimatiensis]
WP_013239529.1 142   MULTISPECIES: hypothetical protein [Clostridium]
WP_050355222.1 142   hypothetical protein [[Clostridium] purinilyticum]
WP_048061031.1 142   hypothetical protein [Methanothermobacter thermautotrophicus]
WP_048175889.1 142   hypothetical protein [Methanothermobacter sp. CaT2]
WP_023613972.1 142   MULTISPECIES: hypothetical protein [Bacillus]
CUS32522.1   141   Rubrerythrin [Nitrospira sp. 1 'RAS filter enrichment']
CUS37263.1   141   Rubrerythrin [Nitrospira sp. 2 'RAS filter enrichment']
WP_053381532.1 141   rubrerythrin [Nitrospira moscoviensis]
AKA48791.1   141   rubrerythrin [uncultured archaeon]
WP_020174695.1 141   rubrerythrin [Methyloferula stellata]
EQB66834.1   141   hypothetical protein AMDU2_EPLC00006G0391[Thermoplasmatales archaeon E-plasma]

WP_054133949.1 141   rubrerythrin [Blastomonas sp. AAP25]
WP_018718822.1 141   rubrerythrin [Arhodomonas aquaeolei]
WP_038093395.1 141   rubrerythrin [Tumebacillus flagellatus]
WP_033925658.1 141   rubrerythrin [Sphingomonas sp. 35-24ZXX]
EQB69900.1   141   rubrerythrin [Thermoplasmatales archaeon Gpl]
WP_053380434.1 141   rubrerythrin [Nitrospira moscoviensis]
WP_009150795.1 141   rubrerythrin [Thiorhodovibrio sp. 970]
WP_038466194.1 141   rubrerythrin [Candidatus Paracaedibacter acanthamoebae]
WP_041607851.1 141   hypothetical protein [Halobacteroides halobius]
Q58156.1   141   RecName: Full=Uncharacterized protein MJ0746
WP_012636492.1 141   hypothetical protein [Halothermothrix orenii]
WP_012981013.1 141   hypothetical protein [Methanocaldococcus sp.FS406-22]
WP_054858328.1 141   hypothetical protein [Methanobacterium formicicum]
WP_048081509.1 141   MULTISPECIES: hypothetical protein [Methanobacterium]
WP_015732525.1 141   hypothetical protein [Methanocaldococcus vulcanius]
WP_015791616.1 141   hypothetical protein [Methanocaldococcus fervens]
WP_048202141.1 141   hypothetical protein [Methanocaldococcus sp. JH146]
WP_018154037.1 141   hypothetical protein [Methanothermococcus thermolithotrophicus]
WP_027098464.1 141   hypothetical protein [Clostridium paraputrificum]
WP_055254081.1 141   hypothetical protein [Clostridium paraputrificum]
WP_007043629.1 141   hypothetical protein [Methanotorris formicicus]
WP_011343502.1 141   MULTISPECIES: hypothetical protein [Carboxydothermus]
WP_013798280.1 141   hypothetical protein [Methanotorris igneus]
WP_044822974.1 141   hypothetical protein [Clostridium aceticum]
CDB14411.1   141   rubrerythrin family protein [Clostridium sp. CAG:221]
WP_049179354.1 141   hypothetical protein [Clostridium botulinum]
WP_021392460.1 141   "rubrerythrin family protein, partial[Peptoclostridium difficile]"
CDB75770.1   141   rubrerythrin family protein [Clostridium sp. CAG:265]
WP_021392642.1 141   "rubrerythrin family protein, partial[Peptoclostridium difficile]"
WP_018868471.1 141   MULTISPECIES: bacterioferritin [Thioalkalivibrio]
KPK37994.1   140   rubrerythrin [Gammaproteobacteria bacterium SG8_47]

WP_052606700.1 140   rubrerythrin [Acidithrix ferrooxidans]
WP_031408378.1 140   rubrerythrin [Thiomonas sp. FB-Cd]
KPK52037.1   140   rubrerythrin [Gammaproteobacteria bacterium SG8_31]
WP_020373958.1 140   rubrerythrin [Sulfobacillus thermosulfidooxidans]
CUQ65510.1   140   Rubrerythrin [Nitrospira sp. ENR4]
WP_018914473.1 140   rubrerythrin [Thiomonas sp. FB-6]
WP_008612981.1 140   rubrerythrin [Magnetospirillum sp. SO-1]
WP_028202767.1 140   rubrerythrin [Burkholderia nodosa]
WP_012403411.1 140   rubrerythrin [Burkholderia phymatum]
WP_007677941.1 140   rubrerythrin [alpha proteobacterium BAL199]
WP_047213704.1 140   rubrerythrin [Pandoraea thiooxydans]
KPK59585.1   140   rubrerythrin [Gammaproteobacteria bacterium SG8_31]
WP_012112497.1 140   rubrerythrin [Parvibaculum lavamentivorans]
WP_027791020.1 140   MULTISPECIES: rubrerythrin [Burkholderia cepacia complex]
WP_054126129.1 140   rubrerythrin [beta proteobacterium AAP99]
WP_011355874.1 140   rubrerythrin [Burkholderia lata]
WP_043177896.1 140   rubrerythrin [Burkholderia cepacia]
WP_042268570.1 140   rubrerythrin [Burkholderia heleia]
WP_018440094.1 140   rubrerythrin [Burkholderia sp. JPY347]
WP_028209815.1 140   rubrerythrin [Burkholderia mimosarum]
WP_039014286.1 140   rubrerythrin [Cupriavidus sp. IDO]
WP_057676928.1 140   rubrerythrin [Curvibacter sp. PAE-UM]
WP_008943073.1 140   rubrerythrin [Oceanibaculum indicum]
WP_006479492.1 140   MULTISPECIES: rubrerythrin [Burkholderia]
WP_028363840.1 140   MULTISPECIES: rubrerythrin [Burkholderia]
WP_041352533.1 140   rubrerythrin [Nitrosospira multiformis]
AKV01752.1   140   Rubrerythrin [Labilithrix luteola]
WP_006497294.1 140   MULTISPECIES: rubrerythrin [Burkholderia]
WP_010110230.1 140   rubrerythrin [Burkholderia oklahomensis]
WP_007582958.1 140   MULTISPECIES: rubrerythrin [Burkholderia]
WP_011464660.1 140   rubrerythrin [Rhodoferax ferrireducens]
WP_026607172.1 140   rubrerythrin [Methylocapsa acidiphila]
WP_028218209.1 140   MULTISPECIES: rubrerythrin [Burkholderia]
WP_047897154.1 140   rubrerythrin [Burkholderia glathei]
WP_048250182.1 140   rubrerythrin [Burkholderia cepacia]
EWS62811.1   140   Rubrerythrin [Hydrogenophaga sp. T4]
WP_053170808.1 140   rubrerythrin [Limnohabitans planktonicus]
WP_019431318.1 140   rubrerythrin [Limnohabitans sp. Rim47]
WP_031399835.1 140   MULTISPECIES: rubrerythrin [Burkholderia]
WP_004190903.1 140   MULTISPECIES: rubrerythrin [Burkholderia]
WP_027796876.1 140   rubrerythrin [Burkholderia acidipaludis]
WP_043698586.1 140   rubrerythrin [Tepidimonas taiwanensis]
WP_009915736.1 140   MULTISPECIES: rubrerythrin [Burkholderia]
WP_043299034.1 140   rubrerythrin [Burkholderia sp. TSV202]
WP_038748009.1 140   rubrerythrin [Burkholderia pseudomallei]
ALK89563.1   140   Rubrerythrin [Limnohabitans sp. 63ED37-2]

WP_055899749.1	140	rubrerythrin [Ramlibacter sp. Leaf400]
WP_007867167.1	140	rubrerythrin [Polaromonas sp. CF318]
WP_019425834.1	140	rubrerythrin [Limnohabitans sp. Rim28]
WP_027821077.1	140	rubrerythrin [Burkholderia bannensis]
WP_034185149.1	140	rubrerythrin [Burkholderia pyrrocinia]
KPK49249.1	140	rubrerythrin [Thiotrichales bacterium SG8_50]
WP_010089086.1	140	MULTISPECIES: rubrerythrin [Burkholderia]
WP_035998670.1	140	MULTISPECIES: rubrerythrin [Burkholderia]
WP_040140994.1	140	MULTISPECIES: rubrerythrin [Burkholderia]
WP_006752513.1	140	rubrerythrin [Burkholderia ambifaria]
WP_038710402.1	140	rubrerythrin [Burkholderia sp. lig30]
WP_006757948.1	140	rubrerythrin [Burkholderia ambifaria]
WP_009694302.1	140	rubrerythrin [Burkholderia cenocepacia]
WP_011660451.1	140	rubrerythrin [Burkholderia ambifaria]
WP_012365587.1	140	rubrerythrin [Burkholderia ambifaria]
WP_028229454.1	140	rubrerythrin [Burkholderia ferrariae]
KRO78598.1	140	rubrerythrin [Methylophilales bacterium BACL14 MAG-120910-bin43]
WP_014900079.1	140	MULTISPECIES: rubrerythrin [Burkholderia]
WP_056269717.1	140	rubrerythrin [Hydrogenophaga sp. Root209]
WP_006765444.1	140	MULTISPECIES: rubrerythrin [Burkholderia cepacia complex]
WP_036259213.1	140	rubrerythrin [Methylocapsa aurea]
WP_006399380.1	140	rubrerythrin [Burkholderia multivorans]
WP_047901447.1	140	rubrerythrin [Burkholderia pyrrocinia]
KPK33907.1	140	rubrerythrin [Betaproteobacteria bacterium SG8_40]
WP_017774084.1	140	rubrerythrin [Burkholderia kururiensis]
WP_023473278.1	140	rubrerythrin [Betaproteobacteria bacterium MOLA814]
WP_011484607.1	140	rubrerythrin [Polaromonas sp. JS666]
WP_042300708.1	140	rubrerythrin [Burkholderia kururiensis]
WP_012827354.1	140	rubrerythrin [Haliangium ochraceum]
WP_028796255.1	140	rubrerythrin [Thalassobaculum salexigens]
WP_027287402.1	140	rubrerythrin [Rhodovibrio salinarum]
WP_011878784.1	140	hypothetical protein [Desulfotomaculum reducens]
ALK92838.1	140	Rubrerythrin [Limnohabitans sp. 103DPR2]
WP_008412913.1	140	hypothetical protein [Desulfotomaculum hydrothermale]
WP_015589891.1	140	rubrerythrin [Archaeoglobus sulfaticallidus]
WP_039376968.1	140	hypothetical protein [Methanobacterium sp. MB1]
WP_048073867.1	140	hypothetical protein [Methanobacterium formicicum]
WP_013180123.1	140	hypothetical protein [Methanococcus voltae]
WP_011973664.1	140	hypothetical protein [Methanococcus aeolicus]
KKM11973.1	140	hypothetical protein SY88_06220 [Clostridiales bacterium PH28_bin88]
WP_011393379.1	140	hypothetical protein [Moorella thermoacetica]

WP_054935301.1 140   hypothetical protein [Moorella glycerini]
WP_053094953.1 140   hypothetical protein [Moorella thermoacetica]
WP_055209409.1 140   "reverse rubrerythrin-1, partial [Clostridium
baratii]"
WP_014960187.1 139   MULTISPECIES: rubrerythrin [Leptospirillum]
KPK14747.1   139   rubrerythrin [Myxococcales bacterium SG8_38]
WP_006970644.1 139   rubrerythrin [Plesiocystis pacifica]
WP_038036680.1 139   rubrerythrin [Thermopetrobacter sp. TC1]
WP_011985128.1 139   rubrerythrin [Anaeromyxobacter sp. Fw109-5]
WP_028109453.1 139   rubrerythrin [Ferrimonas futtsuensis]
WP_009551232.1 139   rubrerythrin [Burkholderiales bacterium
JOSHI_001]
WP_045442038.1 139   rubrerythrin [Tepidicaulis marinus]
ESQ14505.1   139   rubrerythrin [uncultured Thiohalocapsa sp. PB-
PSB1]
WP_026852404.1 139   rubrerythrin [Geothrix fermentans]
WP_054673571.1 139   rubrerythrin [Photobacterium sp. JCM 19050]
WP_002535192.1 139   MULTISPECIES: rubrerythrin [Grimontia]
WP_054779508.1 139   rubrerythrin [Enterovibrio sp. JCM 19048]
WP_043117790.1 139   rubrerythrin [Solemya velum gill symbiont]
KPK55314.1   139   rubrerythrin [Gammaproteobacteria bacterium
SG8_31]
WP_013345268.1 139   rubrerythrin [Ferrimonas balearica]
WP_015006241.1 139   MULTISPECIES: rubrerythrin [Cycloclasticus]
WP_020682170.1 139   rubrerythrin [Marinobacterium rhizophilum]
WP_043107446.1 139   rubrerythrin [endosymbiont of unidentified
scaly snail isolate Monju]
WP_041068470.1 139   rubrerythrin [Thiolapillus brandeum]
WP_040842562.1 139   rubrerythrin [Nitrospirillum amazonense]
WP_044561892.1 139   rubrerythrin [Azospirillum sp. B4]
WP_009470998.1 139   rubrerythrin [gamma proteobacterium HIMB55]
WP_028114575.1 139   rubrerythrin [Ferrimonas kyonanensis]
WP_029641069.1 139   rubrerythrin [alpha proteobacterium Mf 1.05b.
01]
WP_012347695.1 139   rubrerythrin [Leptothrix cholodnii]
WP_020410400.1 139   rubrerythrin [Hahella ganghwensis]
EQB70410.1   139   rubrerythrin [Thermoplasmatales archaeon A-
plasma]
WP_052602919.1 139   rubrerythrin [Actinobacteria bacterium
IMCC26207]
WP_027298811.1 139   rubrerythrin [Rhodospirillales bacterium
URHD0088]
WP_008285772.1 139   "rubrerythrin, partial [Hydrogenivirga sp.
128-5-R1-1]"
WP_012524875.1 139   rubrerythrin [Anaeromyxobacter sp. K]
KJS06671.1   139   rubrerythrin [Gammaproteobacteria bacterium
BRH_c0]
WP_056464529.1 139   rubrerythrin [Rhizobacter sp. Root404]
WP_015260167.1 139   rubrerythrin [Thioalkalivibrio
nitratireducens]

WP_016919075.1 139   rubrerythrin [Methylocystis parvus]
KRP18022.1   139   rubrerythrin [SAR92 bacterium BACL16 MAG-120619-bin48]
KIG19520.1   139   Rubrerythrin [Enhygromyxa salina]
WP_011419823.1 139   rubrerythrin [Anaeromyxobacter dehalogenans]
WP_009574689.1 139   rubrerythrin [gamma proteobacterium IMCC3088]
WP_014238831.1 139   rubrerythrin [Azospirillum brasilense]
WP_036231269.1 139   MULTISPECIES: rubrerythrin [Methylibium]
WP_006746070.1 139   rubrerythrin [Thioalkalivibrio paradoxus]
WP_028099418.1 139   rubrerythrin [Dongia sp. URHE0060]
WP_056193371.1 139   rubrerythrin [Pelomonas sp. Root1237]
WP_036282154.1 139   rubrerythrin [Methylocystis sp. ATCC 49242]
AEJ39129.1   139   Rubrerythrin [Sulfobacillus acidophilus TPY]
WP_014889969.1 139   rubrerythrin [Methylocystis sp. SC2]
WP_022952757.1 139   rubrerythrin [Leucothrix mucor]
WP_041096251.1 139   rubrerythrin [Sulfuritalea hydrogenivorans]
KNZ33714.1   139   rubrerythrin [Methylibium sp. NZG]
WP_007233752.1 139   rubrerythrin [marine gamma proteobacterium HTCC2080]
WP_020558358.1 139   rubrerythrin [Thiothrix flexilis]
WP_020698696.1 139   rubrerythrin [Reyranella massiliensis]
ABB75142.1   139   Rubrerythrin [Nitrosospira multiformis ATCC 25196]
WP_026223003.1 139   MULTISPECIES: rubrerythrin [Methylocystis]
KGA10228.1   139   rubrerythrin [actinobacterium acAcidi]
WP_047484140.1 139   rubrerythrin [Methylibium sp. CF059]
GAP66008.1   139   rubrerythrin [Mizugakiibacter sediminis]
WP_003614530.1 139   rubrerythrin [Methylosinus trichosporium]
WP_002708504.1 139   rubrerythrin [Thiothrix nivea]
WP_020378051.1 139   rubrerythrin [Candidatus Microthrix parvicella]
WP_027948358.1 139   rubrerythrin [Haliea salexigens]
WP_043102137.1 139   rubrerythrin [Oleiagrimonas soli]
WP_045466129.1 139   rubrerythrin [Burkholderiales bacterium GJ-E10]
WP_035389184.1 139   rubrerythrin [Ferrimicrobium acidiphilum]
WP_028489320.1 139   rubrerythrin [Thiothrix lacustris]
GAO35509.1   139   rubrerythrin [Sulfuricella sp. T08]
WP_047510032.1 139   MULTISPECIES: rubrerythrin [Burkholderiales Genera incertae sedis]
WP_047584589.1 139   rubrerythrin [Methylibium sp. YR605]
WP_017940769.1 139   MULTISPECIES: rubrerythrin [Thioalkalivibrio]
WP_018264950.1 139   MULTISPECIES: rubrerythrin [Methylocystaceae]
WP_018953044.1 139   rubrerythrin [Thioalkalivibrio sulfidiphilus]
WP_008435973.1 139   rubrerythrin [Rhodanobacter thiooxydans]
WP_018935136.1 139   MULTISPECIES: rubrerythrin [Thioalkalivibrio]
WP_018946582.1 139   MULTISPECIES: rubrerythrin [Thioalkalivibrio]
WP_019624944.1 139   rubrerythrin [Thioalkalivibrio sp. ALJT]
WP_029010746.1 139   rubrerythrin [Azospirillum halopraeferens]
WP_009959939.1 139   "rubrerythrin, partial [Burkholderia

pseudomallei]"
KRO70450.1 139 rubrerythrin [OM182 bacterium BACL3 MAG-120507-bin80]
WP_018145308.1 139 MULTISPECIES: rubrerythrin [Thioalkalivibrio]
WP_018870615.1 139 MULTISPECIES: rubrerythrin [Thioalkalivibrio]
WP_020505926.1 139 rubrerythrin [Lamprocystis purpurea]
WP_012637414.1 139 rubrerythrin [Thioalkalivibrio sulfidiphilus]
WP_018860677.1 139 MULTISPECIES: rubrerythrin [Thioalkalivibrio]
WP_018971375.1 139 rubrerythrin [Rudaea cellulosilytica]
WP_043950397.1 139 rubrerythrin [Candidatus Phaeomarinobacter ectocarpi]
WP_007194770.1 139 rubrerythrin [Thiocapsa marina]
WP_018233937.1 139 rubrerythrin [Thioalkalivibrio thiocyanodenitrificans]
WP_018939955.1 139 rubrerythrin [Thioalkalivibrio sp. AKL11]
WP_056389959.1 139 rubrerythrin [Rhodanobacter sp. Soil772]
WP_043754752.1 139 rubrerythrin [Thiorhodococcus sp. AK35]
WP_012981831.1 139 MULTISPECIES: rubrerythrin [Thioalkalivibrio]
AJP49090.1 139 rubrerythrin [Rhodocyclaceae bacterium PG1-Ca6]
WP_054966958.1 139 rubrerythrin [Thiohalorhabdus denitrificans]
WP_011311618.1 139 rubrerythrin [Thiobacillus denitrificans]
WP_007507266.1 139 MULTISPECIES: rubrerythrin [Rhodanobacter]
WP_018508286.1 139 rubrerythrin [Thiobacillus thioparus]
WP_019916429.1 139 rubrerythrin [Methyloversatilis discipulorum]
WP_027493107.1 139 rubrerythrin [Rhodanobacter sp. OR444]
WP_014777060.1 139 rubrerythrin [Thiocystis violascens]
WP_018077402.1 139 rubrerythrin [Thiobacillus denitrificans]
AGO87990.1 139 rubrerythrin [uncultured bacterium FPPP_13C3]
WP_018137817.1 139 MULTISPECIES: rubrerythrin [Thioalkalivibrio]
WP_013966504.1 139 rubrerythrin [Nitrosomonas sp. Is79A3]
WP_014448660.1 139 rubrerythrin [Leptospirillum ferrooxidans]
WP_008212885.1 139 rubrerythrin [Rhodanobacter sp. 115]
WP_015434694.1 139 rubrerythrin/nigerythrin-like protein [Azoarcus sp.KH32C]
WP_025041542.1 139 rubrerythrin [Nitrosospira briensis]
WP_028460494.1 139 rubrerythrin [Nitrosomonas cryotolerans]
WP_020394522.1 139 rubrerythrin [Thiothrix disciformis]
WP_041514983.1 139 rubrerythrin [Nitrosospira sp. NpAV]
KRO37926.1 139 rubrerythrin [Acidimicrobium sp. BACL17 MAG-120924-bin0]
WP_004177887.1 139 rubrerythrin [Nitrosospira lacus]
WP_045774421.1 139 rubrerythrin [Elstera litoralis]
WP_015281464.1 139 rubrerythrin [Thioflavicoccus mobilis]
WP_013646950.1 139 rubrerythrin [Nitrosomonas sp. AL212]
WP_007040156.1 139 rubrerythrin [Thiorhodococcus drewsii]
WP_031598757.1 139 rubrerythrin [Ferrovum myxofaciens]
ALQ50049.1 139 rubrerythrin [Nitrosomonas ureae]
WP_018988805.1 139 rubrerythrin [Azoarcus toluclasticus]
KPK18652.1 139 rubrerythrin [Betaproteobacteria bacterium

SG8_41]
WP_018234323.1 139 rubrerythrin [Thioalkalivibrio thiocyanodenitrificans]
KRO46962.1 139 rubrerythrin [Acidimicrobiia bacterium BACL6 MAG-120924-bin43]
WP_008062917.1 139 rubrerythrin [Methyloversatilis universalis]
WP_020023575.1 139 rubrerythrin [Candidatus Thioglobus singularis]
WP_043801586.1 139 rubrerythrin [Arenimonas malthae]
WP_006786179.1 139 rubrerythrin [Thiorhodospira sibirica]
WP_005223844.1 139 rubrerythrin [Marichromatium purpuratum]
WP_003544283.1 139 MULTISPECIES: hypothetical protein [Desulfotomaculum]
AGB42181.1 139 rubrerythrin [Halobacteroides halobius DSM 5150]
WP_011171080.1 139 hypothetical protein [Methanococcus maripaludis]
WP_011972268.1 139 hypothetical protein [Methanococcus vannielii]
WP_048092848.1 139 hypothetical protein [Candidatus Methanoperedens nitroreducens]
EIA22164.1 139 "Rubredoxin/rubrerythrin, partial [Candidatus Arthromitus sp. SFB-2]"
WP_013119741.1 139 MULTISPECIES: hypothetical protein [Thermincola]
WP_011868219.1 139 hypothetical protein [Methanococcus maripaludis]
WP_011976791.1 139 hypothetical protein [Methanococcus maripaludis]
WP_012194264.1 139 hypothetical protein [Methanococcus maripaludis]
WP_012281502.1 139 hypothetical protein [Heliobacterium modesticaldum]
EQH53715.1 139 "reverse rubrerythrin-1, partial [Clostridium difficile DA00245]"
EEO29102.1 139 hypothetical protein OFBG_00130 [Oxalobacter formigenes OXCC13]
WP_018249480.1 139 hypothetical protein [Orenia marismortui]
WP_013100518.1 139 hypothetical protein [Methanocaldococcus infernus]
WP_011839058.1 139 ferritin [Staphylothermus marinus]
WP_012036145.1 139 ferritin [Methanocella arvoryzae]
KON28364.1 139 ferritin [miscellaneous Crenarchaeota group archaeon SMTZ-80]
KPL14442.1 139 ferritin [candidate division WOR_3 bacterium SM1_77]
WP_042684400.1 139 hypothetical protein [Methermicoccus shengliensis]
WP_025771539.1 138 rubrerythrin [Thioalkalivibrio sp. HK1]
WP_044428695.1 138 rubrerythrin [Skermanella aerolata]
KIE49910.1 138 rubrerythrin [marine actinobacterium MedAcidi-

G2B]
WP_002690947.1 138   rubrerythrin [Beggiatoa alba]
WP_021777682.1 138   acetylglutamate kinase [alpha proteobacterium RS24]
KPJ80931.1   138   rubrerythrin [Gammaproteobacteria bacterium SG8_30]
KOR29748.1   138   rubrerythrin [Achromatium sp. WMS1]
WP_025897501.1 138   rubrerythrin [Sneathiella glossodoripedis]
WP_008518902.1 138   rubrerythrin [alpha proteobacterium IMCC14465]
KOR31236.1   138   rubrerythrin [Achromatium sp. WMS2]
WP_027858953.1 138   rubrerythrin [Marinobacterium jannaschii]
WP_014953887.1 138   rubrerythrin [Candidatus Pelagibacter ubique]
WP_013028443.1 138   rubrerythrin [Sideroxydans lithotrophicus]
WP_006997509.1 138   rubrerythrin [Candidatus Pelagibacter ubique]
KPJ91164.1   138   rubrerythrin [Gammaproteobacteria bacterium SG8_15]
WP_005966510.1 138   MULTISPECIES: rubrerythrin [sulfur-oxidizing symbionts]
WP_028036147.1 138   rubrerythrin [Candidatus Pelagibacter ubique]
WP_029132750.1 138   rubrerythrin [Sedimenticola selenatireducens]
ALG68779.1   138   rubrerythrin [Beggiatoa leptomitiformis]
WP_057955360.1 138   rubrerythrin [endosymbiont of Ridgeia piscesae]
WP_023649160.1 138   rubrerythrin [Candidatus Pelagibacter ubique]
WP_046858125.1 138   rubrerythrin [Sedimenticola sp. SIP-G1]
KIE50874.1   138   rubrerythrin [marine actinobacterium MedAcidi-G1]
WP_022728492.1 138   MULTISPECIES: rubrerythrin [Fodinicurvata]
WP_004867815.1 138   rubrerythrin [Acidithiobacillus caldus]
WP_037451704.1 138   rubrerythrin [Skermanella stibiiresistens]
WP_031572415.1 138   rubrerythrin [Acidithiobacillus thiooxidans]
WP_038089562.1 138   rubrerythrin [Acidihalobacter prosperus]
KGA11434.1   138   rubrerythrin [actinobacterium acAcidi]
KGA05359.1   138   rubrerythrin [actinobacterium acAcidi]
KPQ43492.1   138   Rubrerythrin [Candidatus Methanoperedens sp. BLZ1]
CDG66052.1   138   rubrerythrin [Methanobacterium sp. MB1]
CEA13592.1   138   rubrerythrin [Methanobacterium formicicum]
WP_027339386.1 138   hypothetical protein [Halonatronum saccharophilum]
WP_013413504.1 138   hypothetical protein [Methanothermus fervidus]
WP_013643742.1 138   hypothetical protein [Methanobacterium lacus]
WP_013826855.1 138   hypothetical protein [Methanobacterium paludis]
WP_004029438.1 138   hypothetical protein [Methanobacterium formicicum]
WP_022582759.1 138   "rubrerythrin family protein, partial[Peptoclostridium difficile]"
EKQ53716.1   138   rubrerythrin [Methanobacterium sp. Maddingley MBC34]

WP_042693718.1 138   hypothetical protein [Methanobrevibacter oralis]
WP_021371436.1 138   "rubrerythrin family protein, partial[Peptoclostridium difficile]"
WP_015896643.1 138   rubrerythrin [Acidobacterium capsulatum]
WP_037329758.1 138   hypothetical protein [Anaerovibrio lipolyticus]
WP_039208791.1 138   hypothetical protein [Anaerovibrio lipolyticus]
WP_037325452.1 138   hypothetical protein [Anaerovibrio sp. RM50]
WP_009435316.1 138   rubrerythrin [Prevotella sp. oral taxon 306]
WP_004381747.1 138   rubrerythrin [Prevotella veroralis]
EQB63485.1   138   hypothetical protein RBG1_1C00001G1064 [candidate division Zixibacteria bacterium RBG-1]
WP_012547491.1 138   ferritin [Dictyoglomus thermophilum]
WP_012582438.1 138   ferritin [Dictyoglomus turgidum]
KPL03116.1   138   ferritin [candidate division Zixibacteria bacterium SM23_73_2]
WP_028844254.1 138   ferritin [Thermodesulfovibrio thiophilus]
KJR43106.1   138   "Ferritin, Dps family protein [Candidatus Magnetoovum chiemensis]"
WP_045581563.1 137   rubrerythrin [Azospirillum thiophilum]
WP_049724272.1 137   rubrerythrin [Wenzhouxiangella marina]
KGA10291.1   137   rubrerythrin [actinobacterium acAcidi]
CUQ33225.1   137   NADH peroxidase [Fusicatenibacter saccharivorans]
WP_055303962.1 137   rubrerythrin [Dorea longicatena]
CBK83764.1   137   Rubrerythrin [Coprococcus sp. ART55/1]
WP_035915262.1 137   "rubrerythrin, partial [Fusobacterium necrophorum]"
WP_013296519.1 137   hypothetical protein [Methanothermobacter marburgensis]
WP_012062207.1 137   rubrerythrin [Alkaliphilus metalliredigens]
WP_021378732.1 137   "rubrerythrin family protein, partial[Peptoclostridium difficile]"
WP_042702813.1 137   hypothetical protein [Methanobrevibacter arboriphilus]
WP_015051061.1 137   hypothetical protein [Thermacetogenium phaeum]
WP_042707192.1 137   hypothetical protein [Methanobrevibacter wolinii]
ACA13277.1   137   rubrerythrin [Methanobrevibacter arboriphilus]
CEO89731.1   137   Rubrerythrin (fragment) [Syntrophaceticus schinkii]
WP_019265232.1 137   hypothetical protein [Methanobrevibacter smithii]
WP_004032565.1 137   hypothetical protein [Methanobrevibacter smithii]
WP_021426297.1 137   "rubrerythrin family protein, partial[Peptoclostridium difficile]"
WP_021414358.1 137   "reverse rubrerythrin-1, partial

[Peptoclostridium difficile]"
WP_021423763.1 137   "rubrerythrin family protein,
partial[Peptoclostridium difficile]"
WP_021382197.1 137   "rubrerythrin family protein,
partial[Peptoclostridium difficile]"
WP_016359234.1 137   MULTISPECIES: rubrerythrin
[Methanobrevibacter]
KMW18566.1   137   "hypothetical protein HMPREF9471_03174,
partial[[Clostridium] clostridioforme WAL-7855]"
WP_005875589.1 137   rubrerythrin [Oxalobacter formigenes]
WP_036602459.1 137   rubrerythrin [Oxalobacter formigenes]
WP_021368987.1 137   "rubrerythrin family protein,
partial[Peptoclostridium difficile]"
WP_021414644.1 137   "rubrerythrin family protein,
partial[Peptoclostridium difficile]"
AAB85828.1   137   conserved protein [Methanothermobacter
thermautotrophicus str. Delta H]
BAM70473.1   137   rubrerythrin [Methanothermobacter sp. CaT2]
WP_049589410.1 137   "reverse rubrerythrin-1, partial
[[Clostridium]clostridioforme]"
WP_051569420.1 136   hypothetical protein [Alkaliphilus
transvaalensis]
WP_021422857.1 136   "rubrerythrin family protein,
partial[Peptoclostridium difficile]"
WP_021363463.1 136   "rubrerythrin family protein,
partial[Peptoclostridium difficile]"
WP_021361846.1 136   "reverse rubrerythrin-1, partial
[Peptoclostridium difficile]"
WP_046058712.1 136   "reverse rubrerythrin-1, partial [Clostridium
sp.IBUN13A]"
WP_052430932.1 136   hypothetical protein [Robinsoniella
peoriensis]
WP_052377700.1 136   hypothetical protein [Robinsoniella sp.
KNHs210]
CAC80732.1   135   rubrerythrin [Sulfolobus acidocaldarius]
WP_043014061.1 135   "rubrerythrin, partial [Clostridium
perfringens]"
WP_021411397.1 135   "rubrerythrin family protein,
partial[Peptoclostridium difficile]"
WP_021426298.1 135   "rubrerythrin family protein,
partial[Peptoclostridium difficile]"
WP_021426283.1 135   "rubrerythrin family protein,
partial[Peptoclostridium difficile]"
KKC04361.1   135   "hypothetical protein WU83_14230, partial
[Mycobacterium nebraskense]"
WP_021413441.1 135   "rubrerythrin family protein,
partial[Peptoclostridium difficile]"
WP_021413440.1 135   "rubrerythrin family protein,
partial[Peptoclostridium difficile]"
WP_021416190.1 135   "rubrerythrin family protein,

partial[Peptoclostridium difficile]"
WP_021426987.1 135   "rubrerythrin family protein,
partial[Peptoclostridium difficile]"
WP_019554264.1 135   hypothetical protein [Propionispira
raffinosivorans]
WP_052742776.1 134   hypothetical protein [Mycobacterium
nebraskense]
WP_021411568.1 134   "rubrerythrin family protein,
partial[Peptoclostridium difficile]"
WP_021370812.1 134   "rubrerythrin family protein,
partial[Peptoclostridium difficile]"
WP_021374033.1 134   "rubrerythrin family protein,
partial[Peptoclostridium difficile]"
WP_021416254.1 134   "rubrerythrin family protein,
partial[Peptoclostridium difficile]"
WP_026881496.1 134   hypothetical protein [Clostridium akagii]
WP_017209267.1 134   hypothetical protein [Clostridium
beijerinckii]
WP_013064066.1 134   rubrerythrin [Prevotella ruminicola]
WP_028907086.1 134   rubrerythrin [Prevotella ruminicola]
WP_028912454.1 134   rubrerythrin [Prevotella sp. MA2016]
WP_033151107.1 134   rubrerythrin [Prevotella sp. RM4]
WP_003524884.1 134   "hypothetical protein, partial
[[Clostridium]clostridioforme]"
WP_028903412.1 134   rubrerythrin [Prevotella sp. P6B4]
WP_033148836.1 134   rubrerythrin [Prevotella sp. P6B1]
WP_052661211.1 133   hypothetical protein [Clostridium aceticum]
EQI85294.1   133   "rubrerythrin family protein, partial
[Peptoclostridium difficile Y384]"
WP_021363532.1 133   "rubrerythrin family protein,
partial[Peptoclostridium difficile]"
WP_021427586.1 133   "rubrerythrin family protein,
partial[Peptoclostridium difficile]"
EQI85613.1   133   "rubrerythrin family protein, partial
[Peptoclostridium difficile Y384]"
EQH10382.1   133   "reverse rubrerythrin-1, partial [Clostridium
difficile DA00195]"
WP_021378864.1 133   "rubrerythrin family protein,
partial[Peptoclostridium difficile]"
WP_021375237.1 133   "rubrerythrin family protein,
partial[Peptoclostridium difficile]"
WP_023975507.1 133   MULTISPECIES: hypothetical protein
[Clostridium]
WP_012059259.1 133   rubrerythrin [Clostridium beijerinckii]
WP_026888942.1 133   rubrerythrin [Clostridium beijerinckii]
WP_041897503.1 133   rubrerythrin [Clostridium beijerinckii]
KKP85239.1   132   "Protein containing Rubrerythrin domain
protein, partial[candidate division CPR3 bacterium GW2011_GWE2_35_7]"
WP_021404181.1 132   "rubrerythrin family protein,
partial[Peptoclostridium difficile]"

GAE85727.1	132	rubrerythrin [Bacteroides reticulotermitis JCM 10512]
EHM93339.1	132	hypothetical protein HMPREF1021_00674 [Coprobacillus sp.3_3_56FAA]
WP_021372473.1	131	"rubrerythrin family protein, partial[Peptoclostridium difficile]"
WP_021427199.1	131	"rubrerythrin family protein, partial[Peptoclostridium difficile]"
WP_021403185.1	131	"rubrerythrin family protein, partial[Peptoclostridium difficile]"
WP_021406856.1	131	"reverse rubrerythrin-1, partial [Peptoclostridium difficile]"
WP_021364942.1	131	"rubrerythrin family protein, partial[Peptoclostridium difficile]"
WP_021365755.1	131	"rubrerythrin family protein, partial[Peptoclostridium difficile]"
WP_008479038.1	131	rubrerythrin [Nitrolancea hollandica]
WP_027365541.1	130	hypothetical protein [Desulfotomaculum alcoholivorax]
WP_021369014.1	130	"rubrerythrin family protein, partial[Peptoclostridium difficile]"
WP_021419031.1	130	"rubrerythrin family protein, partial[Peptoclostridium difficile]"
KMW18466.1	130	"hypothetical protein HMPREF9471_03181, partial[[Clostridium] clostridioforme WAL-7855]"
WP_021404923.1	130	"rubrerythrin family protein, partial[Peptoclostridium difficile]"
WP_021400898.1	130	"rubrerythrin family protein, partial[Peptoclostridium difficile]"
WP_024616014.1	130	"reverse rubrerythrin-1, partial [Clostridium sp.Ade.TY]"
WP_021406857.1	129	"rubrerythrin family protein, partial[Peptoclostridium difficile]"
WP_021413510.1	129	"rubrerythrin family protein, partial[Peptoclostridium difficile]"
WP_021406855.1	129	"rubrerythrin family protein, partial[Peptoclostridium difficile]"
WP_021419029.1	129	"reverse rubrerythrin-1, partial [Peptoclostridium difficile]"
WP_021411530.1	129	"rubrerythrin family protein, partial[Peptoclostridium difficile]"
WP_021404024.1	128	"rubrerythrin family protein, partial[Peptoclostridium difficile]"
WP_021386990.1	128	"rubrerythrin family protein, partial[Peptoclostridium difficile]"
WP_021413667.1	128	"rubrerythrin family protein, partial[Peptoclostridium difficile]"
WP_021411442.1	128	"rubrerythrin family protein, partial[Peptoclostridium difficile]"
WP_021413666.1	128	"rubrerythrin family protein,

partial[Peptoclostridium difficile]"
WP_021414146.1 128   "rubrerythrin family protein,
partial[Peptoclostridium difficile]"
WP_021406651.1 128   "rubrerythrin family protein,
partial[Peptoclostridium difficile]"
WP_021400902.1 128   "rubrerythrin family protein,
partial[Peptoclostridium difficile]"
WP_051628610.1 128   rubrerythrin [Acidobacteria bacterium KBS 146]
AAM01747.1   128   Rubrerythrin [Methanopyrus kandleri AV19]
WP_021390147.1 128   "rubrerythrin family protein,
partial[Peptoclostridium difficile]"
WP_021389727.1 128   "reverse rubrerythrin-1, partial
[Peptoclostridium difficile]"
WP_052742781.1 128   "hypothetical protein, partial [Mycobacterium
nebraskense]"
WP_021425477.1 127   "rubrerythrin family protein,
partial[Peptoclostridium difficile]"
WP_011020729.1 127   rubrerythrin [Methanosarcina acetivorans]
WP_048169953.1 127   hypothetical protein [Methanosarcina siciliae]
WP_013037098.1 127   rubrerythrin [Methanohalophilus mahii]
WP_048122158.1 127   hypothetical protein [Methanosarcina
vacuolata]
WP_048135681.1 127   MULTISPECIES: hypothetical protein
[Methanosarcina]
WP_048181061.1 127   hypothetical protein [Methanosarcina sp. MTP4]
WP_048157359.1 127   hypothetical protein [Methanosarcina sp.
Kolksee]
WP_048124520.1 127   hypothetical protein [Methanosarcina
lacustris]
WP_048204820.1 127   hypothetical protein [Methanococcoides
methylutens]
WP_048125708.1 127   MULTISPECIES: hypothetical protein
[Methanosarcina]
WP_048107947.1 127   hypothetical protein [Methanosarcina barkeri]
WP_021406701.1 127   "rubrerythrin family protein,
partial[Peptoclostridium difficile]"
WP_048193243.1 127   hypothetical protein [Methanococcoides
methylutens]
WP_011306588.1 127   rubrerythrin [Methanosarcina barkeri]
WP_052712916.1 127   hypothetical protein [Methanosarcina barkeri]
AAU83222.1   127   conserved hypothetical protein [uncultured
archaeon GZfos27A8]
WP_021373107.1 127   "rubrerythrin family protein,
partial[Peptoclostridium difficile]"
WP_048089361.1 127   hypothetical protein [Candidatus
Methanoperedens nitroreducens]
WP_021378209.1 127   "rubrerythrin family protein,
partial[Peptoclostridium difficile]"
WP_039679609.1 127   "reverse rubrerythrin-1, partial
[Terrisporobacter othiniensis]"

WP_009301085.1 127   "hypothetical protein, partial [Coprobacillus sp.3_3_56FAA]"
WP_021416147.1 126   "rubrerythrin family protein, partial[Peptoclostridium difficile]"
WP_021414593.1 126   "rubrerythrin family protein, partial[Peptoclostridium difficile]"
WP_021402040.1 126   "rubrerythrin family protein, partial[Peptoclostridium difficile]"
WP_021414643.1 126   "rubrerythrin family protein, partial[Peptoclostridium difficile]"
WP_021367735.1 126   "rubrerythrin family protein, partial[Peptoclostridium difficile]"
EQF72521.1   126   rubrerythrin family protein [Peptoclostridium difficile CD211]
WP_021364455.1 126   "rubrerythrin family protein, partial[Peptoclostridium difficile]"
WP_012200791.1 126   rubrerythrin [Lachnoclostridium phytofermentans]
WP_049589407.1 126   "reverse rubrerythrin-1, partial [[Clostridium]clostridioforme]"
AAB53660.1   126   unknown [Methanothermobacter thermautotrophicus]
WP_021397593.1 125   "rubrerythrin family protein, partial[Peptoclostridium difficile]"
WP_021400608.1 125   "rubrerythrin family protein, partial[Peptoclostridium difficile]"
WP_021414359.1 125   "rubrerythrin family protein, partial[Peptoclostridium difficile]"
AAM02462.1   125   Rubrerythrin [Methanopyrus kandleri AV19]
WP_021376773.1 125   "rubrerythrin family protein, partial[Peptoclostridium difficile]"
WP_021412252.1 125   "rubrerythrin family protein, partial[Peptoclostridium difficile]"
ESQ20704.1   124   Rubrerythrin [uncultured Acidilobus sp. CIS]
WP_032545214.1 124   "reverse rubrerythrin-1, partial [Peptoclostridium difficile]"
WP_021413167.1 124   "rubrerythrin family protein, partial[Peptoclostridium difficile]"
WP_021411634.1 124   "reverse rubrerythrin-1, partial [Peptoclostridium difficile]"
WP_021402469.1 124   "rubrerythrin family protein, partial[Peptoclostridium difficile]"
WP_021411632.1 124   "reverse rubrerythrin-1, partial [Peptoclostridium difficile]"
BAI60348.1   124   conserved hypothetical protein [Methanocella paludicola SANAE]
GAO30353.1   124   rubrerythrin [Geofilum rubicundum JCM 15548]
WP_021390425.1 124   "rubrerythrin family protein, partial[Peptoclostridium difficile]"
WP_021393289.1 124   "rubrerythrin family protein,

partial[Peptoclostridium difficile]"
WP_011500420.1 124 rubrerythrin [Methanococcoides burtonii]
WP_052219539.1 124 hypothetical protein [Clostridium tetani]
WP_045923706.1 124 hypothetical protein [Clostridium tetani]
WP_021406702.1 124 "reverse rubrerythrin-1, partial [Peptoclostridium difficile]"
WP_055305018.1 124 "reverse rubrerythrin-1, partial [Dorea longicatena]"
WP_021408066.1 123 "reverse rubrerythrin-1, partial [Peptoclostridium difficile]"
WP_032508496.1 123 "reverse rubrerythrin-1, partial [Peptoclostridium difficile]"
WP_012036825.1 123 rubrerythrin [Methanocella arvoryzae]
WP_021386993.1 123 "rubrerythrin family protein, partial[Peptoclostridium difficile]"
WP_032506858.1 123 "reverse rubrerythrin-1, partial [Peptoclostridium difficile]"
WP_049589409.1 123 reverse rubrerythrin-1 [[Clostridium]clostridioforme]
KJZ97793.1 123 hypothetical protein ClosIBUN13A_CONTIG108g01422[Clostridium sp. IBUN13A]
EQH71798.1 123 reverse rubrerythrin-1 [Clostridium difficile DA00306]
WP_021434820.1 123 "rubrerythrin family protein, partial[Peptoclostridium difficile]"
EQH71587.1 123 reverse rubrerythrin-1 [Clostridium difficile DA00306]
EKY24725.1 123 "rubredoxin, partial [Clostridium celatum DSM 1785]"
EHM38836.1 122 "Rubrerythrin, partial [Anaeroglobus geminatus F0357]"
WP_021406703.1 122 "rubrerythrin family protein, partial[Peptoclostridium difficile]"
EKY28180.1 122 rubredoxin [Clostridium celatum DSM 1785]
WP_009301087.1 122 "reverse rubrerythrin-1, partial [Coprobacillus sp.3_3_56FAA]"
EIA30199.1 122 "Rubrerythrin, partial [Candidatus Arthromitus sp. SFB-5]"
WP_031493686.1 122 hypothetical protein [Vibrio shilonii]
CCZ12522.1 121 rubrerythrin [Prevotella sp. CAG:1092]
WP_021403969.1 121 "rubrerythrin family protein, partial[Peptoclostridium difficile]"
CDB00710.1 120 putative uncharacterized protein [Lachnospiraceae bacterium CAG:215]
AGS05774.1 120 ferredoxin hydrogenase [Streptococcus lutetiensis 033]
EIA27813.1 120 "Rubrerythrin, partial [Candidatus Arthromitus sp. SFB-co]"
EIA31540.1 119 Rubredoxin/rubrerythrin [Candidatus Arthromitus sp.SFB-5]

EHM38617.1   119   Rubrerythrin [Anaeroglobus geminatus F0357]
WP_021382056.1 119   "reverse rubrerythrin-1, partial
[Peptoclostridium difficile]"
WP_054269956.1 119   "reverse rubrerythrin-1, partial
[Peptoclostridium difficile]"
WP_054276517.1 118   "reverse rubrerythrin-1, partial
[Peptoclostridium difficile]"
WP_054276425.1 118   "reverse rubrerythrin-1, partial
[Peptoclostridium difficile]"
EJG08417.1   117   "rubrerythrin, partial [Fusobacterium
nucleatum subsp.fusiforme ATCC 51190]"
KQC07090.1   116   "rubrerythrin, partial [Methanolinea sp. SDB]"
EEG51161.1   116   "Rubrerythrin, partial [, partial
[[Clostridium asparagiforme] DSM 15981]"
EEG51411.1   116   "Rubrerythrin, partial [, partial
[[Clostridium asparagiforme] DSM 15981]"
EQE00233.1   115   rubrerythrin family protein [Peptoclostridium
difficile CD8]
EHJ27642.1   115   "Rubrerythrin, partial [Peptoclostridium
difficile 002-P50-2011]"
EIA26882.1   114   "Rubrerythrin, partial [Candidatus Arthromitus
sp. SFB-5]"
EIA28480.1   112   Rubrerythrin [Candidatus Arthromitus sp.
SFB-4]
EKD67035.1   111   "rubrerythrin, partial [uncultured bacterium]"
CEO88395.1   110   Rubrerythrin (fragment) [Syntrophaceticus
schinkii]
CDB90289.1   109   putative uncharacterized protein [Clostridium
sp.CAG:253]
KJR40586.1   108   "rubrerythrin, partial [Candidatus Magnetoovum
chiemensis]"
GAE90246.1   108   rubrerythrin [[Clostridium] straminisolvens
JCM 21531]
EXZ93829.1   108   reverse rubrerythrin-1 [Bacteroides fragilis
str. Korea 419]
EQG84503.1   108   rubrerythrin family protein [Clostridium
difficile DA00183]
EQE58284.1   108   rubrerythrin family protein [Peptoclostridium
difficile CD43]
EQI59342.1   108   rubrerythrin family protein [Clostridium
difficile Y266]
ETI89703.1   104   "hypothetical protein Q612_NSC00131G0001,
partial[Negativicoccus succinicivorans DORA_17_25]"
CCK97857.1   104   Putative rubrerythrin (fragment)
[Peptoclostridium difficile E10]
EQE33744.1   104   rubrerythrin family protein [Peptoclostridium
difficile CD34]
EQI62955.1   104   rubrerythrin family protein [Clostridium
difficile Y312]
EQE33968.1   104   rubrerythrin family protein [Peptoclostridium

difficile CD34]
CEK32186.1   104   rubrerythrin [[[Clostridium] sordellii]
CEJ75096.1   104   hypothetical protein ATCC9714_29841
[[[Clostridium]sordellii]
CCY54826.1   103   reverse rubrerythrin-1 [Bacteroides eggerthii CAG:109]
ETI92262.1   103   "Rubrerythrin, partial [Veillonella sp. DORA_B_18_19_23]"
CDD98359.1   103   rubrerythrin [Roseburia sp. CAG:471]
EFC98151.1   102   "hypothetical protein CLOSTHATH_03668, partial[[Clostridium] hathewayi DSM 13479], partial [Hungatella hathewayi DSM 13479]"
CEK39923.1   102   "hypothetical protein JGS6382_32511, partial [, partial[[Clostridium] sordellii]"
WP_054353688.1 101   hypothetical protein [Clostridia bacterium UC5.1-1D4]
ERM27707.1   101   "reverse rubrerythrin-1, partial [Clostridium difficile P33]"
EQI38400.1   101   reverse rubrerythrin-1 [Clostridium difficile Y184]
EHM93268.1   101   reverse rubrerythrin-1 [Coprobacillus sp. 3_3_56FAA]
